# Convergent Evolution of Cysteine-Rich Keratins in Horny Teeth of Jawless Vertebrates and in Cornified Skin Appendages of Amniotes

**DOI:** 10.1093/molbev/msaf028

**Published:** 2025-02-01

**Authors:** Attila Placido Sachslehner, Leopold Eckhart

**Affiliations:** Department of Dermatology, Medical University of Vienna, Vienna, Austria; Department of Dermatology, Medical University of Vienna, Vienna, Austria

**Keywords:** protein evolution, gene family, intermediate filament, epithelia, cornification

## Abstract

Cornified skin appendages, such as claws and hair, of amniotes consist of keratins with high numbers of cysteine residues, which serve as sites of protein cross-linking through disulfide bonds. Here, we show by proteomic analysis that cysteine-rich keratins are also components of the horny teeth of the sea lamprey (*Petromyzon marinus*), a jawless vertebrate. The cysteine-rich keratins of the lamprey are conserved in hagfish, which diverged from lampreys around 460 million years ago. Phylogenetic analysis confirmed the orthology of the cysteine-rich keratins of lampreys and hagfish (cyclostomes) and showed that cysteine-rich keratins of amniotes belong to different clades of keratins. We conclude that keratins with elevated cysteine content evolved not only in amniotes but also, and much earlier, in jawless vertebrates. The convergent evolution of a high abundance of cysteine residues is in line with a critical role of intermolecular disulfide bonds in hard epithelial structures of vertebrates.

## Introduction

Keratins are the main cytoskeletal proteins of epithelia in vertebrates (Dodemont et al. 1990; Riemer et al. 1998; Zimek and Weber 2005; Bragulla and Homberger 2009; Kimura and Nikaido 2021). Type I and type II keratins dimerize and form intermediate filaments. In humans and other mammals, different epithelia and differentiation stages of cells within a particular epithelium, such as the epidermis of the skin, are characterized by the expression of specific pairs of type I and type II keratins (Moll et al. 2008; Bragulla and Homberger 2009; Bunick and Milstone 2017; Plowman 2018). Accordingly, changes of the keratin cytoskeleton have contributed to the differentiation and adaptation of epithelia during the evolution of vertebrates in different environments.

Epithelial cells of the skin and skin appendages, such as hair and nails, undergo cornification, which converts metabolically active keratinocytes into dead cell remnants, known as corneocytes (Fleckman et al. 2013; Harland and Plowman 2018; Alibardi 2022; Sukseree et al. 2024). The latter are largely filled with keratin intermediate filaments and remain connected to neighboring cells via desmosomes. The corneocytes of the mammalian epidermis contain mainly keratins KRT1, KRT2, and KRT10 which have a similar amino acid composition as other epithelial keratins except for enrichment of glycine residues in their head and tail domains (Strnad et al. 2011). By contrast, corneocytes of hair shafts and nail plates are filled with so-called hair keratins, which are characterized by a high content of cysteine residues (Langbein and Schweizer 2005; Strnad et al. 2011; Fraser and Parry 2018). These cysteine residues are used for intermolecular cross-linking via disulfide bonds to generate an extremely rigid cytoskeleton (Wang et al. 2000; Bornschlögl et al. 2016). Evolutionary precursors of hair keratins evolved in stem tetrapods with a likely function in the protection of toe tips (Carron et al. 2024). However, hair keratin homologs of amphibians contain only small numbers of cysteine residues (*n* < 5, which is markedly less than the minimum number of cysteines in hair keratins, *n* = 20) (Ehrlich et al. 2020), suggesting that the mechanism of epithelial hardening by disulfide bonds evolved in amniotes. Notably, convergent evolution led to high cysteine contents of distinct keratins in sauropsids (reptiles and birds), which were identified as components of scales and feathers (Ehrlich et al. 2020; Lachner et al. 2022). Additional keratins with more than 20 cysteine residues have been found in phylogenetically diverse vertebrates, but their roles have not been determined yet (Ehrlich et al. 2020).

**Fig. 1.**
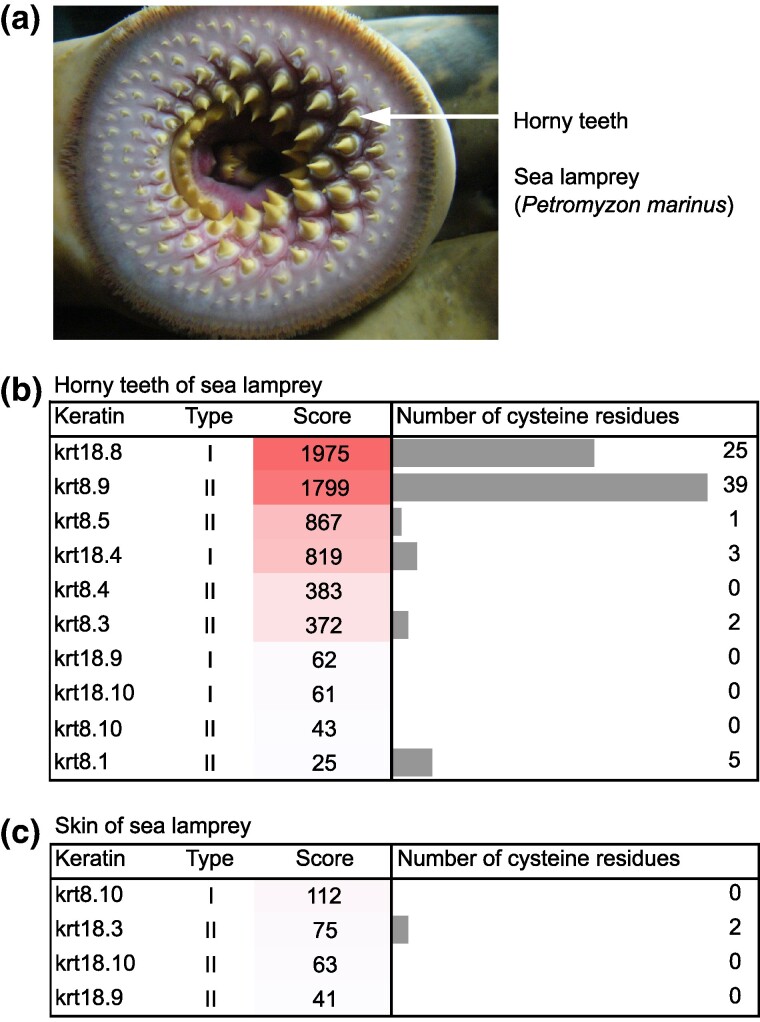
Horny teeth of the sea lamprey contain cysteine-rich keratins. The mouth of the sea lamprey (*Petromyzon marinus*) contains horny teeth a). Mass spectrometry-based detection of keratins in the horny teeth b) and the skin c) of the sea lamprey. The score of the mass spectrometric analysis equals sum of scores of all identified peptides corresponding to the keratin. The number of cysteine residues is indicated by bar charts. The photo in a) is reproduced from the following open access source: https://upload.wikimedia.org/wikipedia/commons/7/72/Petromyzon_marinus.002_-_Aquarium_Finisterrae.JPG, last accessed on August 23, 2024, photographer: Fernando Losada Rodríguez, License CC BY-SA 4.0 (https://creativecommons.org/licenses/by-sa/4.0/).

Lampreys and hagfishes, together forming the superclass Cyclostomata, are the only extant jawless vertebrates (Agnatha). According to recent analyses, they have diverged from the lineage leading to jawed vertebrates (Gnathostomata) around 520 million years ago (Marlétaz et al. 2024). Both lampreys (Fig. 1a) and hagfishes (Clark and Uyeno 2019) have a round mouth with hard conical epithelial structures, here referred to as horny teeth, that are used for grasping food. Being absent from jawed vertebrates with mineralized teeth, the horny teeth of cyclostomes are of similar physical properties as cornified claws in terrestrial vertebrates (Rice et al. 1994). Little is known about the molecular architecture and evolution of horny teeth of cyclostomes (Alibardi and Segalla 2011). Recently, we identified homologs of human transglutaminase 1, which cross-links epidermal proteins via isopeptide bonds, in the horny teeth of the sea lamprey (*Petromyzon marinus*) (Sachslehner et al. 2024).

Here, we investigated the evolution of keratins in cyclostomes and their contribution to horny teeth of lampreys. We provide evidence in support of the hypothesis that cysteine-rich keratins evolved as components of horny teeth, representing a striking example of convergent evolution with cysteine-rich keratins in amniotes.

## Results

### The Horny Teeth of the Sea Lamprey Contain Cysteine-Rich Keratins

To determine the distribution of keratins in a representative species of jawless vertebrates, we performed a mass spectrometry-based proteomic analysis of horny teeth and skin of the sea lamprey. The keratins with the highest sequence score in proteomics were krt18.8, a type I keratin, and krt8.9, type II keratin (Fig. 1b). Both of these keratins, but none of the other keratins detected in either horny teeth or skin, had more than 20 cysteine residues. Neither krt18.8 nor krt8.9 was detected in the skin of the sea lamprey (Fig. 1c).

### Identification of Cysteine-Rich Type I and Cysteine-Rich Type II Keratins in Jawless Vertebrates

Next, we identified keratin genes in the genome sequences of a second species of lamprey, the Far Eastern brook lamprey (*Lethenteron reissneri*), and two species of hagfish, the brown hagfish (*Eptatretus atami*) and the inshore hagfish (*Eptatretus burgeri*). We found that all cyclostomes investigated have 8 to 10 type I and 6 to 11 type II keratins, not including the so-called thread keratins, which are phylogenetically distinct fibrous components of epidermal and defensive slime (Schaffeld and Schultess 2006; Zeng et al. 2023) (supplementary tables S1 and S2, Supplementary Material online; supplementary figs. S1 to S3, Supplementary Material online). All species of lampreys and hagfish investigated have at least one cysteine-rich keratin, defined as containing 20 or more cysteine residues (Ehrlich et al. 2020), among type I and type II keratins. *Eptatretus atami* and *E. burgeri* have two cysteine-rich type I keratins (supplementary fig. S4, Supplementary Material online), and *L. reissneri* has two cysteine-rich type II keratins (supplementary fig. S5, Supplementary Material online).

**Fig. 2.**
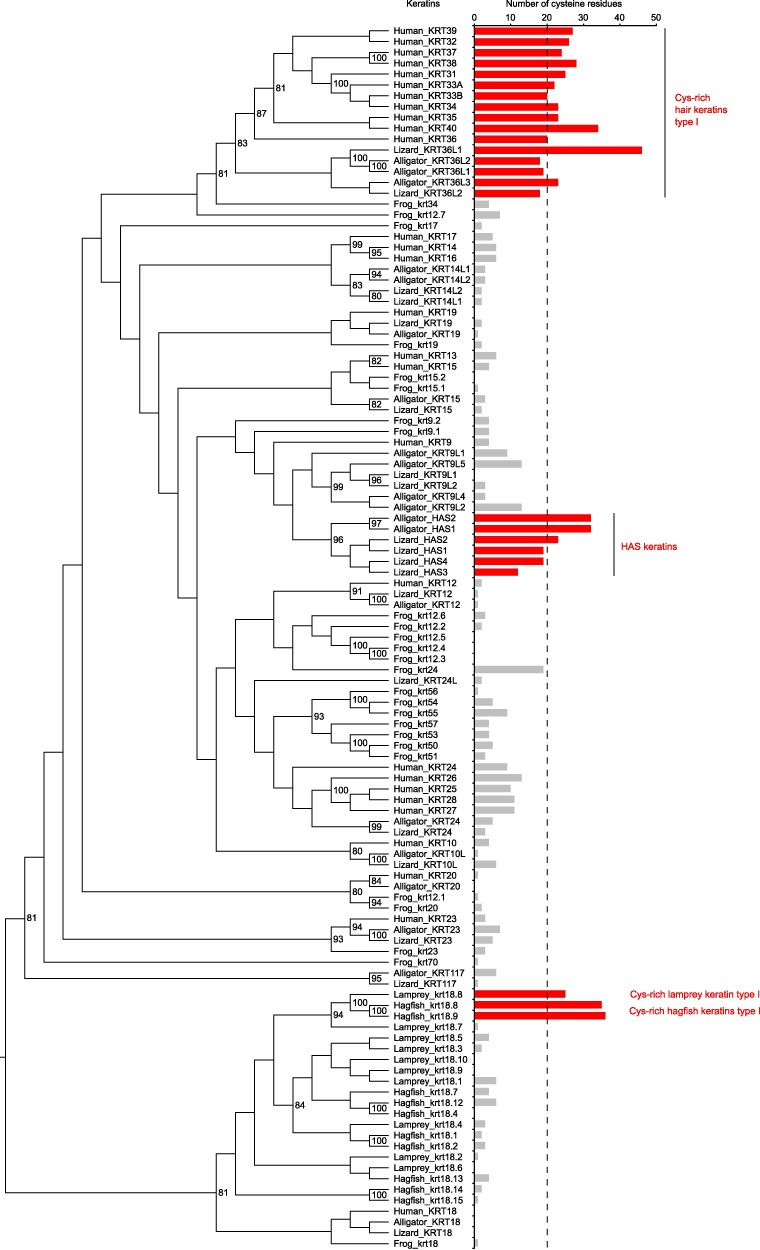
Phylogenetic analysis of type I keratins of cyclostomes and tetrapods. A phylogenetic tree of type I keratins was inferred using the maximum likelihood method. Bootstrap values ≥ 80 are shown. The number of cysteine residues of each keratin is indicated by a bar chart on the right. Hair keratins, hard acidic sauropsid-specific (HAS) keratins, and the cysteine (Cys)-rich type I keratins of cyclostomes are highlighted in red. The dashed line indicates the minimum number of cysteine residues (*n* = 20) in mammalian hair keratins, which is used the threshold for the definition of cysteine-rich keratins. Species: Alligator (*Alligator sinensis*), frog (*Xenopus tropicalis*), hagfish (*Eptatretus burgeri*), human (*Homo sapiens*), lamprey (*Petromyzon marinus*), lizard (*Anolis carolinensis*).

Phylogenetic analysis confirmed that the cysteine-rich keratins of lampreys and hagfish form monophyletic clades among type I (Fig. 2) and type II keratins (supplementary fig. S6, Supplementary Material online). Importantly, type I and type II hair/claw keratins of amniotes and HAS (cysteine-rich KRT9-like) and HBS (cysteine-rich KRT78-like) keratins of sauropsids formed distinct monophyletic clades (Fig. 2, supplementary figs. S7 to S12, Supplementary Material online).

The amino acid sequence alignments showed that the positions of cysteine residues were largely conserved in the keratins among lampreys and hagfish (supplementary figs. S4 and S5, Supplementary Material online). Strikingly, 22 cysteine residues of the cysteine-rich type I keratins are conserved in all species of cyclostomes investigated, and all of the 20 other cysteine positions are present in at least two species (supplementary fig. S4, Supplementary Material online). This high degree of conservation indicates that the cysteine-rich keratin sequences evolved in a common ancestor of lampreys and hagfish, which lived at least 460 million years ago (Marlétaz et al. 2024).

## Discussion

**Fig. 3.**
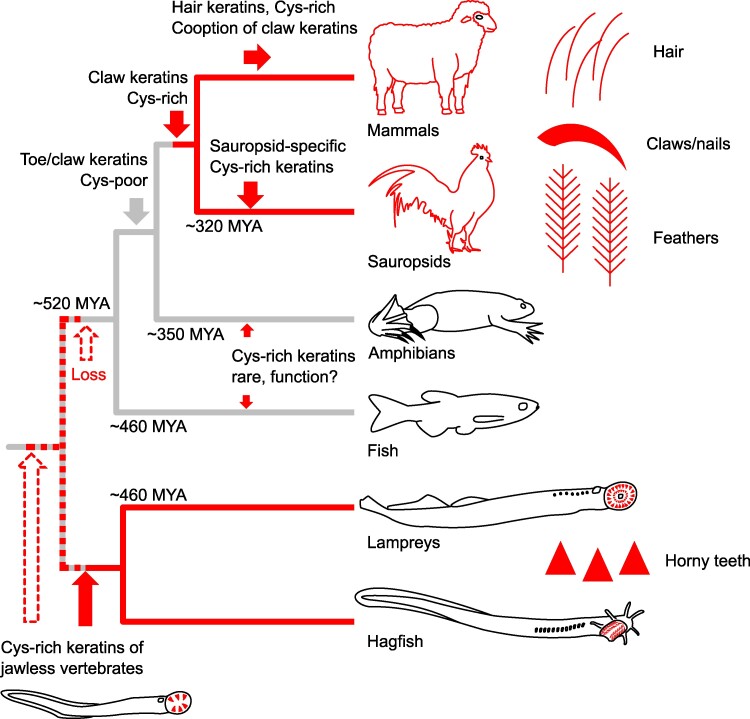
Evolution of cysteine-rich keratins and cornified skin appendages in vertebrates. Cysteine (Cys)-rich keratins evolved independently as components of hard cornified structures of cyclostomes and tetrapods. Red color indicates the presence of Cys-rich keratins. Note that some species of amphibians and jawed fish have functionally uncharacterized Cys-rich keratins not orthologous to the other Cys-rich keratins. Two possible scenarios for the evolutionary origin of horny teeth-associated keratins are indicated: (i) origin of cysteine-rich keratins in stem cyclostomes (red upward arrow), or (ii) origin in stem vertebrates (white upward arrow with a discontinuous frame) followed by loss of these keratins or decline of their cysteine content in stem gnathostomes. Toe/claw keratins orthologous to hair keratins originated in stem tetrapods, but their cysteine content increased only in amniotes after the divergence from the lineage leading to extant amphibians. Divergence times are based on recent literature (Kumar et al. 2022; Marlétaz et al. 2024). Cys, cysteine; MYA, million years ago.

The results of this study show that the cysteine content has not only increased in keratins that build the cytoskeleton of hard skin appendages in amniotes (Eckhart and Ehrlich 2018; Ehrlich et al. 2020) but also in specific keratins of jawless vertebrates. Evidence from proteomic analysis of sea lamprey tissues demonstrates that both the cysteine-rich type I keratin and the cysteine-rich type II keratin of this species are components of horny teeth, suggesting a function equivalent to hair keratins and sauropsid-specific cysteine-rich keratins (Ehrlich et al. 2020) as building blocks of hard cornified structures. As the phylogenetic analysis refuses the hypothesis that cysteine-rich keratins of jawless and jawed vertebrates are orthologous, we propose the following model of evolution of hard keratinized epithelial appendages of vertebrates (Fig. 3). The cysteine contents of originally claw-associated keratins, commonly referred to as hair keratins (Eckhart et al. 2008), and sauropsid-specific cysteine-rich keratins (Ehrlich et al. 2020) increased during the evolution from cysteine-poor ancestral keratins in amniotes. Independently, the cysteine contents of specific keratins increased in a common ancestor of extant cyclostomes, functioning in the hardening of horny teeth. Thus, cysteine-rich keratins of amniotes and cyclostomes are products of convergent evolution. Other cases of presumably convergent rises in cysteine content leading to more than 20 cysteine residues in single keratins of the zebrafish (Ehrlich et al. 2020) (supplementary figs. S13 to S15, Supplementary Material online) and the clawed frog (supplementary fig. S6, Supplementary Material online) remain to be investigated for possible associations with keratinized breeding tubercles (Fischer et al. 2014) or other cornified structures.

The principle of parsimony suggests that the cysteine contents of the type I and type II keratins increased during the evolution of horny teeth in the cyclostome lineage after its divergence from the gnathostome lineage. Gnathostomes have mineralized teeth, and cysteine-rich keratins are absent from phylogenetically basal gnathostomes, i.e. Chondrichthyes (cartilaginous fish) and basal clades of Osteichthyes (bony fish) (supplementary table S3, Supplementary Material online). In another scenario (indicated by white arrows with discontinuous frames in Fig. 3), horny teeth and cysteine-rich keratin components thereof originated already in stem vertebrates and were conserved in cyclostomes. According to this scenario, the horny teeth were replaced by complex mineralized teeth, in which the epithelium contributes to the extracellular enamel without a requirement for cornification, and the ancestral cysteine-rich keratins were either lost or evolved into keratins with low cysteine contents (Fig. 3). An interesting aspect of the second scenario is the possibility that mineralized teeth may have evolved by modifications of ancestral horny teeth.

Several taxa of amniotes have oral cornified epithelial structures that are functionally reminiscent of horny teeth of cyclostomes. Keratinized spines in the mouth and esophagus of the leatherback sea turtle and filiform papillae on the dorsal surface of the tongue of many mammals and birds support the grip of food (Noel and Hu 2018). Convergent evolution of oral cornification is likely but has not been studied yet comprehensively. The expression of hair keratins in lingual filiform papillae of mammals (Langbein et al. 2001; Brychtova et al. 2020) suggests that the evolution of these structures is linked to the evolution of claws and hair.

The identification of the cysteine-rich keratins follows our previous detection of transglutaminases in horny teeth of the sea lamprey (Sachslehner et al. 2024), suggesting that both disulfide-bonded keratins and proteins cross-linked by transglutaminase-dependent isopeptide bonds contribute to the stiffening of the epithelial teeth. These data are compatible with the results of biochemical analysis of hagfish (*Eptatretus stouti*) teeth (Rice et al. 1994), which showed that close to 90% of the horny tooth material could be solubilized by the reducing agent dithiothreitol, which breaks disulfide bonds. The insoluble remainder contained proteins that were cross-linked by transglutaminases. Similarly, hair shafts are predominantly hardened by disulfide bonds with minor contributions of transglutaminases (Bornschlögl et al. 2016; Harland and Plowman 2018; Sachslehner et al. 2023). Previous investigations have revealed histological features of cornification of horny teeth of cyclostomes (Uehara et al. 1983; Zaccone et al. 1995; Alibardi and Segalla 2011), but further studies are necessary to fully define the epithelial cell differentiation process and the biochemical basis for the hardening of horny teeth. In particular, transcriptome analyses and the in situ localization of mRNAs and proteins will help to characterize epithelial cornification in cyclostomes. These studies are important because, according to the results of this study (Fig. 3), cysteine-rich keratins as building blocks of hard cornified structures did not appear for the first time in evolution in fully terrestrial tetrapods (Eckhart et al. 2008), but in jawless fish.

## Materials and Methods

### Identification of Keratin Sequences

Keratin amino acid sequences of *P. marinus* (Timoshevskaya et al. 2023) and *L. reissneri* (Zhu et al. 2021) were downloaded from NCBI GenBank. Amino acid sequences of keratins of *E. burgeri* (Yu et al. 2024) and *E. atami* (Marlétaz et al. 2024) were downloaded from ENSEMBL and Zenodo, respectively. The sequences and accession numbers are provided in supplementary figs. S1 and S2, Supplementary Material online and supplementary tables S1 and S2, Supplementary Material online. Sequences of keratins of other species were downloaded from NCBI GenBank.

When sequences were incomplete or apparently predicted with errors, they were corrected via tBLASTn (Altschul et al. 1990) searches and de novo prediction from a transcriptome (supplementary fig. S3, Supplementary Material online, supplementary table S4, Supplementary Material online). The transcriptome assembly was performed as described previously (Sachslehner et al. 2024). Raw reads which derive from the head region of a juvenile Inshore hagfish (*E. burgeri*) were downloaded from NCBI GenBank (accession number: SRX2541845, run accession: SRR5234495) and converted to fastq files with sra-tools (version: 3.1.1, https://github.com/ncbi/sra-tools, last accessed on August 19, 2024). After quality control with FastQC (version: 0.12.1, https://www.bioinformatics.babraham.ac.uk/projects/fastqc/, last accessed on August 19, 2024), the reads were assembled with Trinity (Grabherr et al. 2011, version: v2.15.2), and coding regions were predicted with TransDecoder (version: 5.7.1, https://github.com/TransDecoder/TransDecoder, last accessed on August 19, 2024). Keratin sequences of *E. burgeri* were corrected using sequence hits obtained by BLASTp (Altschul et al. 1990, version: 2.14.0+) searches against the assembled transcriptome which is available under https://doi.org/10.5281/zenodo.13341147.

### Multiple Sequence Alignments and Molecular Phylogeny

AliView (Larsson 2014) was used to create the multiple sequence alignments for phylogeny. ProtTest (version 3.0) (Abascal et al. 2005; Darriba et al. 2011) was applied to determine the most fitting amino acid substitution model. The Akaike information criterion (Akaike 1998) was used to estimate the likelihood of the best model which was JTT (Jones et al. 1992) for the phylogenies shown in Fig. 2 and supplementary figs. S6 and S7, Supplementary Material online. LG (Le and Gascuel 2008) was the best fitting model for the phylogeny shown in supplementary fig. S8, Supplementary Material online. Phylogenetic analysis, based on maximum likelihood, was performed with PhyML (version: 3.3.20220408, https://github.com/stephaneguindon/phyml, last accessed on July 15, 2024) with optimized tree topology, branch length, and rate parameters (Guindon and Gascuel 2003). The obtained phylogenetic trees were visualized with FigTree (http://tree.bio.ed.ac.uk/software/figtree/, last accessed on July 15, 2024). The resulting images were edited with Inkscape (version: 1.0.0.0; https://inkscape.org/de/, accessed on July 15, 2024). Complete multiple sequence alignments for cysteine content comparison were created with MultAlin (Corpet 1988) with the settings: BLOSUM62 (Henikoff and Henikoff 1992), gap open: 12, gap extension: 2.

### Proteomic Analysis of Keratins of the Sea Lamprey

We analyzed our recently published datasets of sea lamprey tissue proteomes (Pride accession: PXD048873) (Sachslehner et al. 2024). Briefly, these samples were obtained from the cornified teeth and the skin of a sea lamprey specimen (inventory number: NMW-63577) kindly provided by Anja Palandačić from the Natural History Museum Vienna. Teeth and trunk skin were lysed in a buffer containing 30 mM Tris, 7 M urea (catalog number 0568, VWR), 2 M thiourea (catalog number T7875, Sigma-Aldrich), 4% CHAPSO (catalog number 28304, Pierce), and 0.2 M dithiothreitol. Samples were incubated at 70 °C for 3 h before they were homogenized (Precellys, VWR) and analyzed by mass spectrometry-based proteomics (Sachslehner et al. 2024). Proteins were identified using the Sea lamprey database “NCBI_Petromyzon_marinus_tx7757_230919.fasta” downloaded from https://www.thegpm.org/crap/ (last accessed on July 18, 2024).

## Supplementary Material

msaf028_Supplementary_Data

## Data Availability

The proteomic dataset for the lamprey tooth and trunk skin is available in the PRIDE database under the accession number: PXD048873. Genomes used for comparative genomics are available on NCBI GenBank (accession numbers for *Petromyzon marinus*: GCF_010993605.1, *Lethenteron reissneri*: GCF_015708825.1, *Eptatretus burgeri*: GCA_900186335.3) and Zenodo (*Eptatretus atami*: https://zenodo.org/records/10227719).
