# Supplementary material for: Convergent Evolution of Cysteine-Rich Keratins in Horny Teeth of Jawless Vertebrates and in Cornified Skin Appendages of Amniotes
Source: Mol Biol Evol. 2025 Feb 1;42(2):msaf028. doi: 10.1093/molbev/msaf028 (PMC11850651; doi:10.1093/molbev/msaf028)
Supplement: msaf028_Supplementary_Data [file msaf028_supplementary_data.pdf]

Supplementary Data: Supplementary Tables and Figures

Convergent evolution of cysteine-rich keratins in horny teeth of jawless vertebrates and in cornified skin appendages of amniotes

Attila Placido Sachslehner, Leopold Eckhart

Content

Supplementary Figures S1-S15

Supplementary Tables S1-S4

>Pm-krt18.1
MAHRRRSSLDFAYGGRPRETAARTFYPGLALGGGGGAGATAAALAAESLGANGAADEKAEMRGLNERLAEYIAKRPSGRVPASDPKPSPVTRGP
GASPGATAMNGAATLGTLKPLRQHPERPCPNLCKVHYLEGVNHELEVKIKELLKSGHGKIQGSSSRFTAAEELCAKIKSQIVDNARAGVKIENA
RLAADDFHYKLETERSARSCVEEDIARLYALLEEYGVAGAGAEAEIEALSEELHYIRKTHKQDTATLGARLEASSVSVEVASTKGSDLSEILAG
LRRQYEAMIVKTHEEMEFAYKQKMDTVNVTVSEKKQASQLVKDEISDMNHSTQSLQKELDTLWGLIRCLEEQLRSAEALNADSLTGYSSAIGAL
ESELEKLRADTHRQMHEYSLLLNEKMKLEQEISTYRTLLESGHSRLDDTSGAAAAAAAAAAAAASSGKDDGDGVGTTAAAARGGDEDIGRVEKKR
SAVIVVTQHVTDGRVTEESEQSCEH

>Pm-krt18.2
MSQSVRSSRGTHVSTSMWNRRGGSPPPPPRRATSVVSSSREVKISNAASVYGGAGGRGSRISRASSFSAVSTGGLAGNEKEELQGLNNRLSGYL
QRVRTLEQDNNKIELEIAELMSLRKGGPHDWAKWDGIFRALREEIFMLTMDTTKLELGIQNTDLARSDYYEKWETEHAIRMAVEADIQALRKMI
DDTNVARLNLESRVEALKEELSVLRYTHAEEVKKLLVEISSFEVGSVEVDSGTKDIDLNKALAEIRRQYEDLCRQNAVQVESSYLETITITNTK
VTEKMQALEKYRVELNELRRTTQVLFLDLENMRSKKLSLENANADMSARYEMEVSSLRDMVIMLDKILVDVNMDLERQRSEYEMLTNAKMRLEQ
EIAYYSSLLDQEGQISVDGRNFGSTTTTITHIKHTN

>Pm-krt18.3
MTYYSSSSRSVSGGYGGGIVLGRSGAASVYGGSSCQISSASFGRGNFAGGGAGGWGSSQFKMIVGGQGGGVSEKVEMQGLNERLAEYLDKVKYL
ESANQAIELKIKEMLQGKGSTAKDYSVYYSTIEDLREKIFVQILENAKISLEIDNARLAADDFRSKWETEAALRMSVETDIGNLRGLLDEYGMA
CMGLEGDIEALREELIFMKKNHENELAALRAQLSGANMSVEVDSTRGQDLHKILDDMRAQYEGLIAVNRANAEMAFNKQAESVVVQGAQQSQAA
LAAQSEMSETRHAMQSLMTELESLRGLIRSLEDQQYDTEDRNARDLASYTVHIQMLEGELGSVRVGINQQLKDYAELLNMKMKLEQEISTYRRL
LEGEDSRLKNISSESSHSMQSSFSFGAGHKGVTESSSSYQSSSTTGSSSGSGAASSSVSSATKSAGGSSTTGGASTAVTSNTTKTTSQTSSLSS
DGASSAASNSVVVENDASSSSSTTITSSSTTKEVVKEPEPKREEVKRTKTVIITQTIVDGKVVESTEEVAEKTLSDSTDA

>Pm-krt18.4
MTSTMKIRSGGVGSFSSRSAGGSVGTRRLVSSSSSVYGGGLRGGHGIIAGGCYGGGLGYQSHHGLGMGMGLGAGIIQGSDEKQELQGLNDRLAG
YIEKVRILEQANKEIEIKIKELLKGKGPTIKDYSMYYATIEDLRAKILAQTLENARVSLEIDNARLAADDFRSKWETELALRNSVEVDIANLKC
LLDEYNMARMGLEGEIEALREELIFMKKNHEQEVQALRAQIADTSMSVEVDNVKGADLARILADIRAQYEAMIARSRDEAEEAFRKQLETVKQV
SVQQNQVTITAKTELQETRRSMQGLQVELDSMRSMIRSLEDTLADTEDRNARDLSGYQNILARLEAELTSLRCDINRQLKDYADLLNMKMKLEA
EIATYRRLLDGGDTKLANITAGQAPTVLLPSGGSSSTANHAGFYTTGGSGGMGGSSSSTMTITRSSSGSQVN

>Pm-krt18.5
MRQQLPPHRFSPRHQHHAARGASCSYIRPCLFIQPCSAAAVAVVVVAAVVRLKMSSYTRGFSSMSLGGSRARGGLASSYSGARAAGVALPEPAG
RWPGTPLRDAHGEREEMQELNKRLAGYLHKVKELEAANATTEANIQELLRVRGAIVQDHGARFAAIADLRSKMLAQVLENARIGLDVDNARLAA
DDFRCKWETEVALRSSVEADIGNLHMLLDEYTGSRDAMASEAQSMHEELAYMKRNHRERLALSKAQVEGSSVSIQVDSAKGVDLTKMLGDMREQ
YESLIARSRGQAEEAFRKQLESVKVQSFQQDQAASAAKAELAEVRRTMQGLTVELESLMALLGSLEEQLRQTEHDNARELSSHGDQIGALQGRL
HSVHGATNAQLRDYSELLNMKMKLEQEIGTYRRLLEGEESSPVIPAEATKEEVKKRKKVVITQTIVDGEVINTSEEVSESNEKS

>Pm-krt18.6
MSFSSHSASGSSSPRTATTRSFSSSSRMASSRRMATIASASSMSSLPTLKGLHGDEKAEMQGLNGRLATYIERVRSLDEANRRTELQIKELVEK
RPSGAVELRQYHEAARELREQILKATMTNARLHVELDNGRLAAEDFRAKLESEVSIHTSVESDITNLRRAIDETNVTRMSLEGQVEQLEEQILH
MQKSHADEKSSLLKEIEESSISVEVDSVKGHNLNDIIAEIRTQYEALIKSNLQEMEVWYKSKVDAIHPRISQNSEELGTLRQQLSEQRRAMQAL
YAEAETLRSTVSSLNEAAQDVEAHSAEGLAGLAGSISRLLSELGSARGDIDRQLREHETLLNTKMRLEEEIETYRRLLEGQHSHYEERTTVRVT
KDEKEKPNKITKKMVKVVTQEIIDGRVVSERSETQDVQLTSSDSQ

>Pm-krt18.7
MSRAASTMSTSRKFSSHSQSGGGRSVARSSAASVYGGAGGIEVHSGGVFIYGAEPMLGPAKAAFSSSSFSSMSLGRGGGGGGGGAGGGGLLGGD
EKLEMQDLNTRLEEYMEKVRYLESVNKALEIKIKESRASNISTTNYDPLLVNIEALIEQITAAKLVNAQISLEIDNARLAADDFRTKWETEIVL
RQSVEGDIDNLRGLKLDYESESGLLHSQLQLLAEELLYLKKNHAEEIAALRAQCGAEMSVEVDSTPGVDLSKIIAQIRAQYEEMIRKNQEEAEA
AFKKQAEMVKATASQGQAALNTVKMESKEARQSLQTLMLELEMLRSTNKSLEDALADTENRYGHELQLLQAQLQKLEAEIAQVRTGDNAQLQEY
QTLLNAKMKLEMEIATYRRLLEGEDNRLGGAGGAGGAGGAFGAGGGSGGAGGAFGSGYGGGFSSSQTVSSSASKFNGQDDDDDDDDDGVDGTVA
GGVGVMQKRSKKKIVKIITQTIVDGKVVDQDENVQESEMVE

>Pm-krt18.8
MSCSVRSCLAPGRGFSSRSVGVTSRPFRSGFTAWSSAGGSALGGLGGARSAFCYPSRGAPCRPRMRAPTPCMPTVPCGPLFGPCGPLTAAAAPC
GVLLTNEKEALQDLNERLAGYLDKVRYLEQANQELECKIKEFRANSCIAEQQWEPCMGNIEELMGQVEVATLENTRLVLEIDNARLASEDFRVK
WEAESALRETVEADAHELRRLSAEYCACRNQLACEQNVLQGEIHTLKSHHRGEMESLRMDYSSSTTKVEMDNSPGADTAGIISEIRAQYETMIH
NNRHEAECMLQSKLEAAEASAVHSHGELAAAKNQAHHLRQQYQTMEVEMESLRSANATLEDNLAETEERFSVEVRSLAEVLSRLEAEYSDVRAN
VERQLHEYESLLNIKMGLEMEISTYKCLIEGEDSRLSSEVRCNPACCGSSRVCTSLPMPCGSDPCSLNVCSSGPVCTMPCLK

>Pm-krt18.9
MSRRSYSSASAYGSGSVSRISYSSVGSGYGGSGFGGASGGGFGGGSSSVGFSNFNVSGLSGGDEKLEMQGLNDRLAGYIEKVRFLEGANQELEL
KIKELLKGKGGSSKDYSAYYPIIEDLRAKILAQILENARISLEIDNARLAADDFRSKWETELALRSSVEADINNLRGLLDEYNMARMGLEGEIE
SLREELIFLKKNHEEELAALRAQLEGSSMSVEVDSAKGKDLSKILAEIRAQYEAMIARNRVDQEEAFKKQVDTVKVASVQQNQAAQSAKNEVVE
TRRSMQSLQAELDSLRGLIRSLEQQLQDTEERNARELSGYTNVIQRLEGELNNMRGDINRQLKDYSDLLNMKMKLEEEIATYRRLLEGEDSRLN
VSQSSSGGVKM

>Pm-krt18.10
MFSSRSTSSVNLGGNRRSYSASLHGGGGGGGYGGGSISRVSYSNVGSGYGGGFGGSFGGGGGYGGGGYGGGGSVGFSNFNVSGLSGGDEKLEMQ
GLNDRLAGYIEKVKFLEGANRELELKIKEMLKGKGGSSKDYSGYYKIMDDLRAKILVQILENARISLEIDNARLAADDFRTKWETELALRSSVE
ADINNLRGLLDEYNMARMGLEGEIESLREELHFMRKNHEEELAALRAQLEGSSMSVEVDSAKGVDLSKILAEIRAQYEAMIARNRVDQEEAFKK
QVETVKVSSVQQSQAANSAKSEVVETRRAMQSLQAELDSLRGLVRSLDDQLQDTEDRNARELSSYTIIIQRLEGELNNMRGDINRQLKDYSDLL
NMKMKLEAEIATYRRLLEGEDSRYGGGGGGVGG

>Lr-krt18.1
MSCSVRSCLAPGRGFSSRSVGVTSRPFRSGFTAWSSAGGSALGGLGGARSAFCYPSRGAPCRPRMRPPTPCMPTVPCGPLFGPCGPLTAAAAPC
GVLLTNEKEALQDLNERLAGYLDKVRYLEQANQELECKIKEFRANSCIAEQQWEPCMGNIEELMGQVEVATLENTRLVLEIDNARLASEDFRIK
WEAESALRETVEADAHELRRLSAEYCACRNQLACEQNVLQGEIHTLKDHHRGEMESLRMDYSSSTTKVEMDNSPGADTAGIISEIRAQYETMIH

NNRHEAECMLHSKLEAAEASAVHSHGELSAAKSQAHHLRQQFQTLEVEMESLRSANATLEDNLAETEERFSVEVRSLAEVLSRLEAEYSDVRAN
VERQLHEYESLLNIKMGLEMEISTYKCLIEGEDSRLSSEVRCNPACCGSSRVCSSLPMPCGKDPCSLNVCSSGPVCTMPCLK

>Lr-krt18.2
MSFSSRSIGGYGGMSTRLGRGSASVYEGGGSSGGYRISQSSMGGGGGYGGGYGGGGGYGGGCAISSSFQSFGAFGGGGAGGGGLSGGNEKVEMQ
GLNDRLAEYIEKVRFLENANQELELRIKELLKGKGPGNKDYSAYYTTMQELREKILAQIMENARVSLEIDNARLAADDFRSKWETELALRSSVE
ADINNLRGLLDEYSMARMGLEGEIESLREELIFMRKNHEEELAALRAQLEGSSMSVEVDSAKGKDLHKILAEIRAQYEGMIAKNRVDQEEAFNK
QAQSVQVVAVQHSQASQAAKVEVTETRRAMQSLQAELDSLRGLIRSLEDQLQDTEERNARDLSTYTMQIQRLEGELSNLRHGINQQLKEYADLL
NMKMKLEAEIATYRRLLEGEDSRMGNISANSTSGLITVSGGGGGGGGVSGGGGGGGGMSMTMTSSSSSSGGGGGGGGISSSGGGSVVKTTTTKE
SSSYSTGYRS

>Lr-krt18.3
MSRAGSTMSTARKFSSHSQSGGGGGHSVARSSAASMYGGGAGGIEVHSGGVFIYGAEPVLSSSKAAYSSSSFSSMSLGRGGGGGGAGGGGLLGG
DEKLEMQDLNTRLEEYMEKVRYLESVNKALEIKIKESRASNISTTNYDPLLVNIEALIEQITSAKLVNAQISLEIDNARLAADDFRTKWETEIV
LRQSVEGDIDNLRGLKLDYESESGLLHSQLQLLTEELLYLKKNHAEEMAALRAQCGAEMSVEVDSAPGVDLSKIIAQIRAQYEEMIRKNQEEAE
AAFKKQAEMVKATASQGQAALNTVKMESKEARQSLQTLMLELEMLRSTNKSLEDALADTEGRYGHELQLLQAQLQKLEGEIAQVRAGDNAQLQE
YQTLLNAKMKLEMEIATYRRLLEGEDNRLGGAGGAGGAGGAFGAGGGGGGGGGAFGAGGGGGGAGGGAFGSGYGAGYSSSQTVSSSASKFNGQD
DDDDDDDDGVDGTVAGGVGVIQKRSKKKIVKIITQTILDGKVIDQDENVQESEMIE

>Lr-krt18.4
MSFSSHSASGSSSPRTATTRSFSSSSRMAPGRRMATIASASSMSSLPTLKGLHGDEKAEMQGLNGRLATYIERVRSLDEANRRTELQIKELVEK
RPSGAVELRQYHEAARELRGQILKATMTNARLHVELDNGRLAAEDFRAKLESEVCIHTSVESDITNLRRAIDETNVTRMSLEGQVELLEEQLLH
MQKSHADEKSSLLKEIEESSISVEVDSVKGHNLNDIIAEIRAQYEALIKSNLQEMEVWHKSKVDEIHPRISQNSEELGTLRQQLSEQRRAMQAL
YAEAETLRCTVSSLNEAAQDVEAHSAEGLAGLAGSISRLLSELGSARGDIDRQLREHEALLNTKMRLEEEIETYRRLLEGQHSHCEEQTTVRVT
KDEKQKPSKITKKMVKVVTQEIIDGRVVSESSETQDVVLTSSDS

>Lr-krt18.5
MSFSSHSASGSSSPRTATTRSFSSSSRMAPGRRMATIASASSMSSLPTLKGLHGDEKAEMQGLNGRLATYIERVRSLDEANRRTELQIKELVEK
RPSGAVELRQYHEAARELRGQILKATMTNARLHVELDNGRLAAEDFRAKLESEVCIHTSVESDITNLRRAIDETNVTRMSLEGQVELLEEQLLH
MQKSHADEKSSLLKEIEESSISVEVDSVKGHNLNDIIAEIRAQYEALIKSNLQEMEVWHKSKVDEIHPRISQNSEELGTLRQQLSEQRRAMQAL
YAEAETLRCTVSSLNEAAQDVEAHSAEGLAGLAGSISRLLSELGSARGDIDRQLREHEALLNTKMRLEEEIETYRRLLEGQHSHCEEQTTVRVT
KDEKQKPSKITKKMVKVVTQEIIDGRVVSESSETQDVVLTSSDS

>Lr-krt18.6
MAYYSSSSRSVSGGGGGGIFLGRSGAASVYGGSSCRVSSASFGRSNSAGCGGGAWGSSQFKMIVGGQSGASEKVEMQGLNERLAEYLDKVKFLE
SANQAIELKIKEMLQGKGSIAKDYSVYYSTIEDLREKIFVQILENAKISLEIDNARLAADDFRSKWETEAALRMSVEADIGNLRGLLDEYGMAC
MGLEGDIEAMREELIFMKKNHEDELAALRAQLSGANMSVEVDSTRGQDLHKILDDMRAQYEGIIAVNRANAEMAFNKQAEIAMVQGAQQSQAAS
AAQSEVSETRHAMQSLAAELESLRALIRSLEDQLYDTEDRKARELASYTVHIQMLEGELGSVRMGINQQLKDYAELLNMKMKLEQEISTYRRLL
EGEDSRLRNISSESSHSMQSCFSFGAGSKGESSSSYQSSSTTGSGSGSGAASSSVSSATKSTGGASTAVTSSTTKNSSYQSSSTTGSGSGSGAA
SSSISSATKSAGGSSTTGGASTAMTSSTTKTTSQTSSLSSDGAFGAASNSVVVDNDASSSSSSSTTITNSSTTKEVVIEPEPKREEVKTTKTVI
ITQTIVDGQVVESTEEVAEKTLPN

>Lr-krt18.8
MAHGRRSSLDFSYGGRPRETAARTFYPGLPLGRGDGAGATAAALGADSLGANGAADEKAEMQGLNERLAEYIGKVHYLEGVNHELEVKIKELLK
SGHDKTQASGSRLAAAEELCAKIKSQTVDNARAGVKIENARLAADDFHYKLETERSARSCVEEDIARLHALLEEYGAAGAGAKAEIEALSEELH
YIRKTHQQDMATLGARLEASSVSVEVASAKGSDLSEILAGLRRQYEAMIVKTHEEMEFAYKQKMDTVNVTVFEKKQASQLVKDEISDMKHSSQS
LQKELDTLWGLIRCLDEQLRSAEALNADSLSGYSGAISTLESELAKQRADIHRQMHEYSLLLNEKMKLEQEISTYRALLESGHRRLDDTSTAAV
VAAAASSGTDDGDGVGTAAAAPARGRDEDIGRGEKKRSAVIVVTQHVTDGRVTEESEERCEH

>Lr-krt18.9
MTSTMKIRHGGVGGFSSRSAGGSVGTRRVVSSSSSTYGGGLRGGHSIIAGGCYGGGLGYQSNYGLGMGMGLGAGIIHGSDEKQELQGLNDRLAG
YIEKVRILEHANKEIEIKIKELLKGKGPSIKDYSVYYSAIEDLRAKILTQTLENARVSLEIDNARLAADDFRSKWETELALRNSVEVDIANLKC
LLDEYNMARMGLEGEIEALREELIFMKKNHEQEVLALRAQIADTSMSVEVDNVKGADLARILADIRSQYEGMIARSRDEAEEAFRKQLETVKQV
SVQQNQVTITVKTELQETRRSMQGLQVELDSMRSMIRSLEDTLADTEDRNARDLSGYQNILARLEAELTSLRCDINRQLKDYADLLNMKMKLEA
EIATYRRLLDGGDTKLANITAGQAPTVLLPSGGSSSTANHGGFYTTGSSAGMGGSSSSTMTITRSSSGSQVN

>Ea-krt18.1
MSCSAHSCSASQRNFSSRSCASLLGGKRAPRCTAFSLCRSTTGRPTRSSICNPCCNRPGICLPRPCLPPVPCGPCGSFPCGPLTSAAAPCGVLL
TNEKQTMQDLNDRLSGYLDKVRYLEATNLELECKIKEFRANKCIAEREWDPCLSGIESLMYQIEEASVENSQLVLAIDNARLTAEDFRTKWEIE
VSLREDLEIDIQDLRNLANEYCECRNTVACEINVLEDEICHLKNSHREEIEALRHEYANSTLNVDIDHTPGVDLAGIINDIRTQYETMISSTHY
EVECLIQNKLDAAQASAHQSACNLEAIKTEANNLRYEFQALEVEAESLRSANCILEENLHETEARLNLEISNLAQIISILEADYTDVRSNIERQ
IREYESLLNVKMQLEMEIATYQQLIDCEDSRLANVSCGRVCYGDAVRCTALPVPCGANPCSLNVCPSEPKCGGPCGVCGPCLPCA

>Ea-krt18.2
MYGGAGGSGTRGSYRVSSFGYGSGHGLGYGSGHGLGYGSSHGLGYGGSSLGSHLGGGLGYASGLGSSLLSGGGVIITNEKQEMQTLNDRLAEYL
DKVHFLEKANRELEIKIKEILKSKGVTLKDYSTYYSIIEDLRNKILLQILENARVSLEIDNARLAAEDFRNKWQMELNLRTSVENDIGGLRSML
DEYNMARLGLEGEIEAMREELVFMKKNHEQEVGALKAQIEDSTMSIEVDSTPGPDLSKTLTEIRAQYENMIAKNRDDAEVTFRKQVETHQTIIV
QQDQATQSIKVELQETRRNLQGLNVELESLKSMIRSLEDTLADTEDRNARDLAFYQNSISRLEAEYASMRCDINRQLKDYSDLLNVKMKLEKEI
ATYRSLLEGEEGRLSTVSSSQLGITTGGGSSTLILSGGGANSSSIGAGSTAGRSSTYEYSSSSTGTGGGSNLLSFPSIDRTSSSTSGGSTSG

>Ea-krt18.3
MSTWFRNNTSRSQGSVKIIHRPVSRPASVYAGAGGMGTRGNYRTSTIEFGSKQGLGFGGYFSPSSFRCARMGCRTPLAIGFGVKMNKEKQEMQS
LNDRLAEYLDNVRFLEKTNEDLEFKIKDILKLKGVTLKDCTAYYSNIEELRKKILTRAQDNAKITLEMDNAKLAAEDFKSKWQMELNLRTSVET
DIANLRSFLDEYNMERLGMEGEIEALQEELTFMKKNHQEEVAALNAQIENSSMSVEVDSIPGMDLSKTISEIRAQYENMIAKNREDAENTFRKQ
VDSHQAVPMQDDLTTQSKKLELQEMRRSMQGLQMELETLHNVVASLKDNLADTENHNVRELSGYASIVTNLESELASMHLDINRQLKDYSDLLN
VKMRLEQEINTYRRLLEGEEAWSSVKEEESNVVSTQQEKKMKRVIITQTIVDGKVVSENMEESEQSYND

>Ea-krt18.5
MSCSIRSCYSTPHRYSSRSCASLLGGKRAPRCTAFSVCRNPCGRPTRSSICNPCCNRPGICLPRPCLPPVPCGPCGSFPCGALTSAAVPCGVLL
TNEKQTMQDLNDRLSSYLDKVRYLEATNLELECKIKEFRANKCIAEREWDPCLSGIESLMYQIEEASVENSQLVLAIDNARLTAEDFRKKWEIE
VSLREELETDIQDLRNLANEYCECHNTVACEVNVIQDEIYYLKQSHREEIEGLRCEYANSTLNVDIDHSPNVDLSSIINDIRTQYETMIGNTHY
EVECLMNNKLEASQASAHQSACELEATKNEANNLRYEFQSLEVEAESLHTANCTLEENLHETEERLNLELSNLAEIISSLEAEYTDVRANIERQ
IREYESLLNVKMQLEMEIGTYQQLIEGEDCRMSNVVCGPVCCGSAMRCRALPVPCGANPCSLNVCPSEPKCGPCL

>Ea-krt18.6
MNAFRSYSSQSLVSSRPTFRVAHSGGSMSGSFGRLNSEKEAMQDLNDRLSNYMDKVHRLEVANGELEMKIDEMLKRHGPEISNNNVYFSTIEEL
KSKILAQVMENASLTLEIDNARLAADDFHTKWQTEVTLHSSVEGDIGSLGMLLDEYTLSRAGLETDVEFLQDELAYMRKNHEDEVTALRAQITS
SGMSVEVDSTPGIDLARALKDMRSQYEAVVAQNNADAEATFQKQAEKVKIVVEQRSQSSDAAKADVLESRRSMQTLQVELDTLRGQVNSLEFNL
TETEGRKAQELGSYEIVIQRLQQKLQSMKNDLNGKLGEYSELLNQKMLLEAEIATYRLLLDGNSSSSSISKYSTIENSETVSRITDQDLLSSER
STTMMNPQTKIAPVESPTTSQRKKTVIITEVIKDGRVVSSSQDVAERYMPH

>Ea-krt18.7
MTSCTYSSSPKGSRICNAIGSCSISDGRHSVSSLRLTESSTWSSWGQKEGPVQTTPNSRFASESFPGSVSAGKRILHGNEKEHLRQLNDRLAGY
MDKVKLLEQTNQNLENQIKEIFGKVNQTPDLSNFYNQVEELKGNAHRISVENTQLALAIDNGKEEAKEFRIKIESETAAMRAIEYDIGSLNQIV
EKIQTERKHLDEQLGSLQREMESIRSNHNEKLQGLRVAANDEGVHVKVDDVRGADLVQILADMRSEYENITQKSRADIEAIYQTQIEHANPNTA
QNLEAIELAKKQANDLRRQMQALTMDIQTQSNAISSLKSTLNHSEERYSYELNKFLPITGYLEEELSKIKGNLEHQRQEYDTALNAKMMLEMEI
ATYRQLLEAGGARDGEQTDDKIVYDVPDYDIPWKPSTDSNVNNASNKQDPCLDNRATAKPPGDGDSCAVNTDVRASSAPPFGVQGTGCLDDSSR
IGDRLCKDGISSLDSGLSLPGLLGSKSPISSQGSDSALYITKECKRESVLRT

>Ea-krt18.8
MQEATCTGDYKCASPGQGALRNSIFEQTAAEPADRRCTHIMSRSPSLRATGERRASGGGTTSRNFSATSWRGSSSSTFGTMHGSAPGGAVCRSN
LMSDLQYVNPKMEMQDLNTRLAEYVDKVRILEHSNEDLEVKIKQLLINRNAATNTDYTHYYDTVKDVQKKLLEKHLENESIVLKIDNAHLAADD
FKSKWDTECGVRNSVASDITNLRTLLDEYTLARTELETDVEAQKDELAYIKKSHAEDLAELRSHLETTLNVTEMKGDDLTDVLSNLRSQYETFI
DKIRDEAEENFRKLVSTVSPQEEQQTKVLSDVKQEQEELRRSYKIFQNDLESLRKTIEPLESEFANTQNRKERELANFLRVIGRLEGELNTAND
EHDRQLRAYSALLNEKMRLEQEIATYRRLLEDEMARIVVEVDDFTDGPSQSPIMGTPRGLSPSLTPGRAIARSPSPGMKEEPFLSASPSPTGHF
VLSDGEYKLEREDDDDQSDLTRKKQVRVITQMIVNDEVVSVKQDIVSGATKG

>Ea-krt18.9
MDQTERMLSNMSVSSMETSGIDRYRTSSLPRKHGFSSQSLTVGSRQQPSFSSRTSILGGGSRHSFQSIGKWVNGGFSSGSAHSASSAFSPGYGS
GASRFQSMVLAHSEKETLQQLNDRLDDYLKRVRSLESYNSKIEMEIKHLITSYGPEMIDWESQEKHLEELRVQLAELTLDNANLSMQAETNYLS
IKDFQMKLEIEQNLCHNIEMDINEMRKEIDSINLNVLELEGNNESLVASLMMMKKQHKERVKELKANIAVEDLSNVQVDSIQGMDLLAVLTSIR
EEYNRLSKKNEQDAEVWYREKIQNIKEVVGQGEAELNPLDEQLHSLRQEHHNLQLTINSSGTKKEFLRKSLTDVEIHFNSERNRMKGYMGQYES
TLAGLKEELEKQLGAYEQLMSTKIRLEQELAEYHNLLEHGDIQTRTSSC

>Ea-krt18.10
MAASSSAPPRRAMSIDGRARRHDMRISPGCTTFGNEQTQLKDLNERLGIYLERVSYLEKINGEMEASIKVELEQRAANVPDWTRYEQIIKDLRQ
KINDTINENTSLSLEIDNNRFAGSDFRQKWETENDLYQAVEGDIASLRSQTNDINLSCSELETHLECLRSDLESMRRQHKEEKEKLSKGLSDGM
SVEVDAVKGINLNEILNGVRNKYEEIVKKNQQEAEEEYQSKCKMAAPQMMKSCEALEASRSEVTELRSLLQTLSIEYQSLNSMVFSLEEMLQET
EDRKANELSRLQSSLLKAEANLEGAQKNLRGQMQEFDILLDAKMDLEREIAMYHKLINGEDKMVDYPHPTPEPSTPTKKKVVKVVTQEIVDGQV
VSEVSETQEALSL

>Eb-krt18.1
MTSSVSIRTTSKGGYSSRSQGNLGFGRRPIGSSASMYGGAGGSGTRGSYRVSSFGYGSGHGLGYGSGHGLGYGSCHGLGFGGSSLGSHLGGGLG
YASGLGSSLVSAGGVIITNEKQEMQTLNDRLAEYLDKVHFLEKANRELEIKIKDIIKSKGVTLKDYSTYYSIIEDLRNKILLQILENARVSLEI
DNARLAAEDFRNKWQMELNLRTSVENDIGGLRSMLDEYNMARLGLEGEIEAMREELVFMKKNHEQEVGALKAQIEDSTMSIEVDSTPGPDLSKT
LSEIRAQYENMIAKNRDDAEVTFRKQVETHQTIIVQQDQATQSIKVELQETRRNLQGLNVELESLKSMIRSLEDTLADTEDRNARDLAFYQNSI
SRLEAEYASMRCDINRQLKDYSDLLNMKMKLEKEIATYRSLLEGEEGRLSTVSSSQLGITTGGGSSTLVLSGGGANSSAIGAGSTAGRSSTYEY
SSSSTGTGGGSNLLSFPSIDRTSSSTSGGSTSGKSVTVITQTLVDGKVVSENQEVSRSGRR

>Eb-krt18.2
MSTWFRNNTSRSQGSVKIIHRPVSRPASVYAGAGGMGTRGNYRTSTIDFGSKQGLGFGGYFSPSSFRCARMGCRTPLAIGFGVKMNKEKQEMQS
LNDRLAEYLDNVRFLEKTNEDLEFKIKDILKLKGVTLKDCTAYYSNIEELRKKILTRAQDNAKITLEMDNAKLAAEDFKSKWQMELNLRTSVET
DIANLRSFLDEYNMERLGMEGEIEALQEELTFMKKNHQEEVAALKAQIEKSSMSVEVDSIPGMDLSKTISEIRAQYENMIAKNREDAENTFRKQ
VDSHQAVPMQDDLTTQSKKLELQEMRRSMQGLQMELETLHNVVASLKDNLADTENHNVRELSGYANIVTNLESELASMHLDINRQLKDYSDLLN
VKMRLEQEINTYRRLLEGEEAWSSVKEEESNVVSTQQGE

>Eb-krt18.4
MNTKQFSMRSLQSSGSNSSGRFSQHSRAFGSRSMAYGSGSMAYGSGGMAYGSGGMAYGSGGLGFNQSAMGLSSASMQMQGHEKQAMQNLNERLH
IYLQKVQSLENANAQLEIQIRELQKGKAPTSSDYESYYAIIVDLRAKIITQIMSNASISLEIDNARLAADDFKTKWDNEFALRTSVEADIDNLH
GLHEEYMMASSSMQGELELLQDELNFMKKNHAEEVAALKAQIAGSNMSIEVDAKSGPDIQQMLEDMRRKYEVIMEQNRVEAEQAFQQQVEQVQQ
VVVKQNQAAVSAKNEVTEVRQSMQSLQIELGTLQGQIASLENTLDDTKYRKQQELESYLIMLERVERDFENARMEINTKKHDYAKLLDEKMKLE
TEIETYRRLLNGGGSGILISSSGMGGASKSGGLALTDGMSSLSSNTSFISASQTSNLNAAAGNRSGYSSSTSYTSSGNTGGNSSSLLTSGGTTG
GIQSSSYKSGGTTGGIQSSSYISGGTSGGIQPSSYASGGFISRGSSSSTGTSSSMNGGMNSYSMEQSQFQSLQQGTQQPKLEKRKKVIILTTTY
VDGKEVTSEEEVSQSVLST

>Eb-krt18.7
NVMYLNFMNSFRSYSSQSLVSSRPTFRAAHSGGSMSGSFGRLNSEKEAMQDLNDRLSNYMDKVRRLEVTNGELEMKIDEMLKRHGPEISNNNVY
FSTIEELKSKILAQVMENASLTLEIDNARLAADDFHTKWQTEVTLHSSVEGDIGSLGMLLDEYTLSRAGLETDVEFLQDELAYMRKNHEDEVTA
LRAQITSSGMSVEVDATPGIDLARALKDMRNQYEAVVAQNNADAEATFQKQVTEKVKIVVEQRSQSSDAAKADVLESRRSMQTLQVELDTLRGQ
VNSLEFNLTETEGRKAQELGSYEIVIQRLQQKLQSMKNDLNGKLGEYSELLNQKMLLEAEIATYRLLLDGNSSRYWFNESLSCTNQHLHPLNQE
GVDGGPCVPCRNLRGRTKHGKSRRCKTRKRRW

>Ebkrt18.8
MSCSIRSCYSTPHRYSSRSCASLLGGKRAPRCTTFSVCRNPCGRPTRSSICNPCCNRPGICLPRPCLPPVPCGPCGSFPCGALTSAAVPCGVLL
TNEKQTMQDLNDRLSSYLDKVRYLEATNLELECKIKEFRANKCIAEREWDPCLSGIESLMYQIEEASVENSQLVLAIDNARLTAEDFRKKWEIE

VSLREELETDIQDLRNLANEYCECHNTVACEVNVIQDEIYYLKQSHREEIEGLRCEYANSTLNVDIDHSPNVDLSSIINDIRTQYETMIGNTHY
EVECLMNNKLEASQASAHQSACELEATKNEANNMRYEFQSLEVEAESLHTANCTLEENLRETEERLNLELSNLAEIISSLEAEYTDVRANIERQ
IREYESLLNVKMQLEMEIGTYQKLIEGEDCRMSNVVCGPVCCGSAMRCRALPVPCGANPCSLNVCPSEPKCGPCL

>Ebkrt18.9
MSCSAHSCSASQRNFSSRSCASLLGGKRAPRCTAFSLCRSTSGRPTRSSICNPCCNRPGICLPRPCLPPVPCGPCGSFPCGPLTSAAAPCGVLL
TNEKQTMQDLNDRLSGYLDKVRYLEATNLELECKIKEFRANKCIAEREWDPCLSGIESLMYQIEEASVENSQLVLAIDNARLTAEDFRTKWEIE
VSLREDLEIDIQDLRNLANEYCECRNTVACEINVLEDEICHLKNSHREEIEALRHEYANSTLNVDIDHTPGVDLAGIINDIRTQYETMIASTHY
EVECLIQNKLDAAQASAHQSACNLEAVKTEANNLRYEFQALEVEAESLRSANCILEENLHETEARLNLEISNLAQIISILEADYADVRSNIERQ
IREYESLLNVKMQLEMEIATYQQLIDCEDSRLANVSCGRVCYGDAVRCTALPVPCGANPCSLNVCPSEPKCGGPCGVCGPCLPCA

>Eb-krt18.12
MSLSLTTSFASSHRSNFGGGGYGGYCGSNFVGGGGGGSYSGGSIRMGSSSLQGNEKQAMQGLNDRLARYLEKVQSLERSNAEIELKIKELLQGR
GPENKDYNHYFEIITDLKNTIVQQIMENAKIGLEIDNARLAADDFKTKWENENTLRNSVEADIANLHSLHDEYTLARSDLECDIEGLQEELNFM
RKNHEEEVAALKAQIAGSNMSIEVDAKSGPDIQQMLEDMRRKYEVIMQQNRAEAEQAFQQQAEQVKAVVVKQDQACSTAKNEVVETRRSMQSIQ
TEMETLRGLCNSLEDQLADINDRKARELEQYLNAVACLENDIATSRSNINEQLCKYSALLDEKMKLEKEIETYRTLLSGGESMKLPDSSRVGLV
GI

>Eb-krt18.13
MAASSSAPPRRAMSIDGRARRHDMHISPGGTTFGNEQTQLKDLNERLGVYLERVSYLEKINGEMEASIKVELEQRAANVPDWTRYEQIIKDLRQ
KINDTINENTSLSLEIDNNRFAGSDFRQKWETENDLYQAVEGDIASLRSQTNDINLSCSELETHLECLRSDLESMRRQHKEEKEKLSKGLSDGM
SVEVDAVKGINLNEILNGVRNKYEEIVKKNQQEAEEEYQSKCKMAVPQMMKSCEALEASRSEVTELRSLLQTLSIEYQSLNSMVFSLEEMLQET
EDRKANELSRLQSSLLKAEANLEGAQKNLRGQMQEFDILLDAKMDLEREIAMYHKLINGEDKMVDYPDPTPEPSTPTKKKVVKVVTQEIVDGQV
VSEVSETQEALSM

>Eb-krt18.14
MDQTERMLSNMSVSSMETSGIDRYRTSSLPRKHGFSSQSLTVGSRQQSSFSSRTSILGGGSRHSFQSIGKWVNGGFSSGSAHSVSSAFSPGYGS
GASRFQSMVLAHSEKETLQQLNDRLDDYLKRVRSLESYNSKIEMEIKHLITSYGPEMIDWESQEKHLEELRVQLAELTLDNANLSMQAETNYLS
IKDFQIKLEIEQNLCHNIEMDINEMRKEIDSINLNILELEGNNESLVASLVMMKKQHKERVKELKANIAVEDLSNVQVDSIQGMDLLAVLTSIR
EEYNRLSKKNEQDAEVWYREKIQNIKEVVGQGEAELNPLNEQLHSLRQEHHNLQLTINSSGTKKEFLRKSLTDVEIHFNSERNRMKGYLGQYES
TLAGLKEAGKTARCL

>Eb-krt18.15
MDQTERMLSNMSVSSMETSGIDRYRTSSLPRKHGFSSQSLTVGSRQQSSFSSRTSILGGGSRHSFQSIGKWVNGGFSSGSAHSVSSAFSPGYGS
GASRFQSMVLAHSEKETLQQLNDRLDDYLKRVRSLESYNSKIEMEIKHLITSYGPEMIDWESQEKHLEELRVQLAELTLDNANLSMQAETNYLS
IKDFQIKLEIEQNLCHNIEMDINEMRKEIDSINLNILELEGNNESLVASLVMMKKQHKERVKELKANIAVEDLSNVQVDSIQGMDLLAVLTSIR
EEYNRLSKKNEQDAEVWYREKIQNIKEVVGQGEAELNPLNEQLHSLRQEHHNLQLTINSSGTKVSESQRITTSQSIRSS

Figure S1. Amino acid sequences of type I keratins of lampreys and hagfishes investigated in this study. Species abbreviations: Ea*, Eptatretus atami*; Eb, *Eptatretus burgeri*; Lr, *Lethenteron reissneri*; Pm, *Petromyzon marinus*.

>Pm-krt8.1
MSSLSASYVQHIGSSHSQHQRRRCLVNRSYGAGGGWCPSLQGPRTPVAFVPHGGTSRVRSWGVPPAPPPGIDRGVLPVRFQEKEQIKKVNDRFV
RFIEKVGILEQQNKVLEVQWLLLQDRGARGSKVEASVFFQSQIHGLQRQLDTLERDKQRRQGELRLTQGLVEDFKNKYEEDKSIRNKAENEFVA
VKQDFDDSCLVKAELEARLEGLKDEINFLRGIFEKELRDLETQIKDTLVSAGVDTSRSIDVQGMISDVHAQYELIAAKSQAEANEFYRKKYDLL
SLSADPTDQEIRSNRNEMNNLHRQIQRVRGESDALKKQRASLQAAIAGAVDRGGLSVCEAKERIARLEEELHKDKQQKVQRVREYQELMNVKLA
LDIEIATYGRLLEGEESRLGCVTSQKQHFSLGFGSDLVNKGHHGSSHATTNFDKAINLYKPPNKTVEIRTTETRNGRILYESSEFIHEVIDFPG
DAAVV

>Pm-krt8.2
MSYSSMMQSSSSSGVVSSLQSPMERMGLQDQRSPFAMTAAGGSGGGYSSTSTTTVQGSAYISSGMSSGNSAEVDKLQQVRQMEKNQLMGLNDRF
ANYIQNVRILERKNKELQVSLKRMRIHSGQDSQLDNLCAQYEVLLKRQIQALIEEKRRLANDQEQAHINMEQMKIKYETEIRERTQIENEFVVL
KQEADQVYMQKVELEARLTGLTDDIDFYRKVYEREIRELEARMVKVDVVVEVDSSPGLDLDTYLAEVREQYMKQAGRIREELKISFETKLDANR
NNEVKINDDMRLVTVEISEVRRNMQRYKAELEALKQQCLALERAIALAEEKGKESMRQLTEKRQLLEVTIMEQKNKLTGHHRSYQELMNVKLAL
DMEIIAYRKLLEGEEGRITKTTIGSTVSEYSPSPSPLETKPQFQGYSSQQVLIKQIETLDGRVVSESQDMQVVRK

>Pm-krt8.3
MASITISTAKKASVRSSVPRYSAGFSSRSLGGSGGWGRVSGVGGAGGLARRSALTSGYRAGSAASGFGYGYGVGGAGAAASAAALACAAPVDID
RSVLPTRTQEKEAIKHLNDRFANFIDKVRFLEQQNKVLEAQWYALQEKTTTGSSVDEMFEAYINGLRRQLDGLGHENGRANGDLGQMQAVVEDF
MSKYEAEITARIHKENEYVVIKKDVDAANVIKIELEAKMQGLVEDINFLREIFAQELSELEAQIKHTNVLVEIDNSRNLDVDGIIADVRHQYET
IAARSRAEADAFYQDKFDRLHADSGRNDEEMRVIRTEINDNNRHIHRMRAEIDALKKQRAKLEAAIAEAEGRGEADIREAKAAIAQLEEEIHKA
KQEMARHVREYQELMNVKLALDIEIVTYRKLLEGEESRLNVYSSQIGGNASSVTYKTCVDTTPYYYGGGGGGNYSGTSTVGLSASTGYSGGSTY
NYSVPTKAVETKQSKKMVVIKTIETKDGHVVSEQSEVIRD

>Pm-krt8.4
MSASIKVSTTKRTSTRSSAGGPSVVSGGFSSRSFGGGVGGSRFGAGGGSAFSRRSTALGGSSYRAGSAASSFGYGSGSGGYGYGVGGAGAAISA
AALASAAPVDIDRSVLPTRTQEKEAIKHLNDRFANFIDKVRFLEQQNKVLEAQWYALQEKTTTGSSVDEMFEAYINGLRRQLDGLGHENGRANG
DLGQMQAVVEDFRAKYEAEISLRTEREDEFVFAKKDVDGAYLSKVELEAKLDGLQEDINFMRSIFAQELSELEAQIKHTNVLVEIDNSRNLDVD
GIIADVRHQYETIAARSRAEADAFYQDKFQSLHSDSGRNDEELRVTRNEITELNRQMQRFRAEMEALKKQRAKLESAIAEAEGRGEADIREAKA
AIAQLEEEIHKAKQEMARHVREYQELMNVKLALDIEIVTYRKLLEGEESRLNVITQHLTYGGGQQMLSQGGGVYMAEGVTGAIVSHKTTSTVTM
SSGAYGGGVTYETL

>Pm-krt8.5
MSASIKVSTTKRTSTRSSAGGPRVVSGGFSSRSFGGGMGGSRFSAGGGSAFSRRSTALGGSSYRAGSAASSFGYGSGSGGYGYGVGGVGAAISA
AALASAAPVDIDRSVLPTRTQEKEAIKHLNDRFANFIDKVRFLEQQNKVLEAQWYALQEKTTTGSSIDEMFEAYINGLRRQLDGLGHDKGQLNG
NLGQMQAVVEDFRAKYEAEINNRNAAEGEFVVVKKDVDGAYLSKVELETRLQGLVDEINFLRSIFTQELSELEAQIKHTNVLVEIDNSRNLDVD
GIIADVRQQYETIAARSRAEADAFYQDKFQSLHSDSGRNDEELRMTRNEITELNRQMQRFRAEMEALKKQRAKLESAIAEAEGRGEADIREAKA
AIAQLEEEIHKAKQEMARHVREYQELMNVKLALDIEIVTYRKLLEGEESRLNVITQHITQSGGQLHSQGGSAFGGDMMCSIGGGGSYSGGGSYS
GGGSHSGGGNYSQMSSGSIKMSTHY

>Pm-krt8.6
MASTRSSTVRSSSTRISVGGGSGGGSRGFSSTSLSAGGRSGGRKGVLSSASFRSGGGGGGGFGGFSASSAAMLSGPPPEIDRSILPVRTQEKDQ
IKGLNDRFANFIDKVRFLEQQNKVLEAQWRALHERGTVVSGLDGLFDAYTRGLKQQLESLSNNKLGLQNELQAMQGHVEDFKTKYEEEINTRTE
RENEFVVVKKDVDDAFLNKVELEAKLEALQDEIEFLKKIFAEELRQLESQIQDTILTVEIDTSRNLDVDGLIADVRAQYEAIAAKSKAESDAFY
KSKFADLNAMSGKGDDELRQSRSEIADINRQVQRIKAEIEALKKQRAQLEAAIAEAEGRGELAIKEAKESIARLEEELHQAKQDMAKHVREYQE
LMNVKLALDIEIATYRKLLEGEESRLQNVTAGLANLGGMGGYGGYSVSTSYETSSKRSY

>Pm-krt8.7
MSLRNRGFSSKSVVTGGSLKGARSSVRYDGRASRSSAGVLFTGAPPPEIDRSVQDVRSQEKDQIRGLNDRFANFIDKVRSLEQQRSVLDAQWKV
LQAKGEDKSNLEDIYQEYIRGLRRQLEMLQENKEHLQSDVGHMQGVVEEFKNKYETELNNRNHAENEFVLIKKDFDDAHLNKVELEARLEGLTD
EIDFLRRIYEEELRELHAQMNNISLTVEVDTNRHFNMDDIVASVRSQYEALAQQSRQEAEDFYRVKFEDINASADKSNEDIRNSKQELNDLLRT
IKNLTSEVQRLKQQRGQAERAVAEAEDLGEQALKDAKKRIADLEQELADSRRQMAQHVRDYQELMNVKLALDIEIATYGKLLEGEEDRLSSLAG
ARGRNPRNYSYNFGDDDLTCHYESAVPRSSKTVIIKSIETQDGSVVSETTEVRN

>Pm-krt8.8
MSYNNLPSFATTAGFVRGVGPHHRGGRGGGVSGGVDGGGVSGGQFTSRSFAGLDSSQPRSERGTWSKSSVPGVVGRRSALLLGYRGGSGGGGGF
GSAYTGGYGRVSSSVRARPPVPVVEIDRSVLPIRAQEKEEIKMLNNRFANFIDKVRILEQQNKVLEAQWYVLQDKGKVDCNLDELFEVYIEELR
RQLQNLGHNGARMNSDLQMIKHAGKDFSSKYEVEVAAHKKKVEEHELVKKEAVEASFVKIELEAKFQGLIEDIHFLREAFAQELRELEAQIKNT
NVLVEIDNRRSLDMDGIISEVRKQYEFIMSQSQAEAEAIYKQKFDKLRSESDRSDEELRKAKAEINDIKRQIHQINSELDILSKQREKLEAAIS
EREEGGAKNIGEAKAAVAQLEEALAKAKHDMVKHVRDFTDLMNIKLALDTEILTYRKLLEGEEARLNIFASRGGGGVASSSLEPGGSVRERLGG
SCGQHIGGSQGKDSRSSSSNNNVYDSPDNRVCEASPRLNGTLGRSVTVASEPPEHESRKTVVIKTIETKNGRVVSESSAVILD

>Pm-krt8.9
MSYSKPCEPCVPNKYVSNFAPTPKLPKPCAPCHAPCPAPALCPRPCPPRTTVRCCTPGPEPCHDSGHGFSSLAPFIHGGAFYTGGAAHGSKPSC
SSYVPFTSAPCGPPPCGPVHHGSFHAPCGPAPCAPVPKCSKAVPVCPRPCPPPVCPTPCPRPSACIPRVSMEPLCPAPCVQIDRSGQNVRCQEK
NAISHLNDRFASFIDKVRYLEQENKVLEAQWGCLQARVCTSDLDSMFETYAQAIKRQLDCVLADRPRLETELHQTRALADEHKLKYEAEVTGRE
AAEANFVAIKREADDNYMGKVQMETRVGQLSDELHFIKELFACEMQEMEERIRDMNVTIDLDTGCNFDLSSLIAEVRANYEMVAARSREEVECW
YKSKMDDMAEASERHCIELRCTKNEILELSSMLQRLACEIDGLKTQRYQLESSIQEAETRGELNAHEARDATARVEMELSQAKADMARHVRDYQ
ELMNVKLALDIEIATYRKLLEGEESRLHCVPDRCGQPCMPCDPCGPSRRCL

>Pm-krt8.10
MSYSIKSSGVSSVSRSRSSGGSSIRIGGGGGGRSGGFSSHSQGFGSGGSRSRSSFKSGGSSYGGGGGGGFGGISASFSSQVAAGPPPEIDRSVL
PVRNQEKEQIKGLNDRFANFIEKVRFLEQQNKVLEAQWRALQERGSGGSNLDSLFEAYINGLKQHLDGLGNEKQRLQGELQHMQGAVEDFKAKY
EEEINTRTQRENEFVVVKKEVDDAFLNKVELEARLDGLNDEIEFLKKIFAEELRQLESQIKDTSLFVTVDTSRNLDVDGLIADVRAQYEAMARK
SQAEADDFYKKKISELSSSAGKGDDDVRNSRNEINELNRQMQRIKAEIEALKKQRAQLEAAISEAEGRGEMSIKEAKETIARLEEELHKAKQQM
AQHVREYQELMNVKLALDIEIATYRKLLEGEESRLNAVASGIANLAGAMSGGGGGGGGYGFSSGGGGGGGGGGYGFSSSGGGGGGSGFSYESSS
MSKRSY

>Lr-krt8.1
MASITISTARKASVRNSVPRHSAGFSSRSLGGGGGGWGRFSSVGGAGGFARRSALTSGYRAGSAASSYGSYGSYGYGGAGAAVSAAALACAAPV
DIDRSVLPTRTQEKEAIKHLNDRFANFIDKVRFLEQQNKVLEAQWYALQEKTTTGSSIEEMFEAYINGLRRQLDGLGHENGRANGELHQMQAVV
EDFMSKYEAEITARIHKENEYVVIKKDVDAAHVIKIELEAKMQGLVEDINFLREIFAQELSELEAQIKHTNVLVEIDNSRNLDIDGIIADVRHQ
YETIAVRSRAEADAFYQDKFDRLHSDSGRNDDELRIIRTEINDNNRHIHRMRAEIDALKKQRAKLEAAIAEAEGRGEADIREAKAAIAQLEEEI
HKAKQEMARHVREYQELMNVKLALDIEIVTYRKLLEGEESRLNVYSSQIGGNASSVTYNTCVDATPYYYGGGGSYSGTSTVGLSASTGYSGGST
YNYTVPTKVVETKQSKKMVVIKTIETKDGHVVSEQSEVIRD

>Lr-krt8.2
MSASIKVSTTKRTSTRSSAGGPRVVSGGFSSRSFGGGMGGSRFGSGGGSAFSRRSTGLGGSSYRAGSAASSFGYGSYGSGGYGGGVGAAVSAAA
MACAAPVDIDRSVLPTRTQEKEAIKHLNDRFANFIDKVRFLEQQNKVLEAQWYALQEKTTTGSSVEEMFEAYINGLRRQLDGLGHDKGQLNGNL
GQMQAVVEDFRAKYEAEISMRTEREGEFVFAKKDVDGAYLSKVELEAKLDGLQEDINFLRSMFAQELSELEAQIKHTNVLVEIDNSRNLDIDSI
IADVRHQYETIAARSRAEADAFYQDKFQSLHSDSGRNDEELRMTKNEITELNRQMQRFRAEMEALKKQRAKLESAIAEAEGRGEADIREAKAAI
AQLEEEIHKAKQEMARHVREYQELMNVKLALDIEIVTYRKLLEGEESRLNVITQHLTYGGGQQMLSQGGGVYMAEGVTGATITRKTTSTVTMSS
GGYGGGVTYETL

>Lr-krt8.3
MASTRSTTVRSSSTRISVGGSGGSRGFSSASLSAGGRSGGRKSGLSSASYRSGGGGGFGGFSASSAAMLSGPPPEIDRSVLPVRTQEKDQIKGL
NDRFANFIDKVRFLEQQNKVLEAQWRALHERGTVVSGLDGLFDAYIRGLKQQLESLSNNKLGLQNELQAMQGHVEDFKTKYEEEINTRTERENE
FVVVKKDVDDAFLNKVELEAKLEALQDEIEFLKKIFSEELRQLESQIQDTILTVEIDTSRNLDVDGLIADVRAQYEAIAAKSKAESDAFYRSKF
ADLNAMSGKGDDDLRQSRSEIADINRQVQRIKAEIEALKKQRAQLEAAIAEAEGRGELAIKEAKESIARLEEELHQAKQDMAKHVREYQELMNV
KLALDIEIATYRKLLEGEESRLQNVTAGMANLGGMGGYGGYSVSTSYETSSKRSY

>Lr-krt8.4
MSASIKVSTTKRTSTRSSAGGPRVVSGGFSSRSFGGGMGGSRFGSGGSSAFSRRSTGLSGSSYRAGSAASSFGYGSYGSGGYGGGVGAAMSAAA
MACAAPVDIDRSVLPTRTQEKEAIKHLNDRFANFIDKVRFLEQQNKVLEAQWYALQEKTTTGSSIDEMFEAYINGLRRQLDGLGHDKGQLNGNL
GQMQAVVEDFRAKYEAEINNRNAAEGEFVVVKKDVDGAYLSKVELETRLQGLVDEINFLRSIFTQELSELEAQIKHTNVLVEIDNSRNLDIDGI
IADVRHQYETIAARSRAEADAFYQDKFQSLHSDSGRNDEELRMTKTEITELNRQMQRFRAEMEALKKQRAKLESAIAEAEGRGEADIREAKAAI
AQLEEEIHKAKQEMARHVREYQELMNVKLALDIEIVTYRKLLEGEESRLNVITQHITQSGGQLHSQGGSSFGGDMMCSLGGGGSHSGSVSYSGG
GSYSQMSSGGSIKMSTHY

>Lr-krt8.5
MSLRNRGFSSKSVVTGGSLKGARSSIRYDGRASRSSAGVLFTGAPPPEIDRSVQDVRSQEKDQIRGLNDRFANFIDKVRSLEQQRSVLEAQWKV
LQAKGDDKSNLEDIYQEYIRGLRRQLEVLQENKEHLQSDVGHMQGVVEEFKNKYETELNNRNHAENEFVLIKKDFDDAHLNKVELEARLEGLTD
EIDFLRRIYEEELRELHAQMNNISLTVEVDTNRHFNMDDIVASVRSQYEALAQQSRQEAEDFYKVKFEDINASADKSNEDIRNAKQELNDLLRT
IKSLTSEVQRLKQQRGQAERAVAEAEDLGEQAVKDAKQRIADLEQELADSRRQMAQHVRDYQELMNVKLALDIEIATYGKLLEGEEDRLSSLAG
ARGRNSRNYSYNFGDDDLTSHYESAVPRSSSKTVIIKSIETQDGSVVSETTEVRN

>Lr-krt8.6
MSYSKPCEPCVPNKYVSNFAPTPKLNKPCAPCHAPCPAPALCPRPCPPRTTVRCCTPGPEPCHDSGHGFSSLAPFIHGGGFYTAGAAHGSKPSC
SSYVPFTSAPCGPAPCGPVHHTDFHAPCGPAPCAPKPKCTKAVPVCPRPCPPPVCPTPCPRPSACIPRISMEPLCPAPCVQIDRSGQNVRCQEK
NAISHLNDRFASFIDKVRYLEQENKVLEAQWGCLQARVCTSDLDSMFETYAQAIKRQLDCVLADRPRLETELHQTRALADEHKLKYEAEVTGRE
AAEANFVAIKREADDNYMGKVQMETRVGQLSDELHFIKELFACEMQEMEERIRDMNVTIDLDTGCNFDLSSLIAEVRANYEMVAARSREEVECW
YKSKMDDMAEASERHCIELRCTKNEILELSSMVQRLACEIDGLKTQRCQLESSIQEAEHHGEMNAHEARDATARVETELSQAKADMARHVRDYQ
ELMNVKLALDIEIATYRKLLEGEESRLHCVPDRCGQPCMPCDPCGPSRRCL

>Lr-krt8.7
MSYSKPCEPCVPNKYVSNFAPTPKLNKPCAPCHAPCPAPALCPRPCPPRTTVRCCTPGPEPCHDSGHGFSSLAPFIHGGGFYTAGAAHGSKPSC
SSYVPFTSAPCGPAPCGPVHHTDFHAPCGPAPCAPKPKCTKAVPVCPRPCPPPVCPTPCPRPSACIPRISMEPLCPAPCVQIDRSGQNVRCQEK
NAISHLNDRFASFIDKVRYLEQENKVLEAQWGCLQARVCTSDLDSMFETYAQAIKRQLDCVLADRPRLETELHQTRALADEHKLKYEAEVTGRE
AAEANFVAIKREADDNYMGKVQMETRVGQLSDELHFIKELFACEMQEMEERIRDMNVTIDLDTGCNFDLSSLIAEVRANYEMVAARSREEVECW
YKSKMDDMAEASERHCIELRCTKNEILELSSIVQRLACEIDGLKTQRCQLESSIQEAEHHGEMNAHEARDATARVETELSQAKADMARHVRDYQ
ELMNVKLALDIEIATYRKLLEGEESRLHCVPDRCGQPCMPCDPCGPSRRCL

>Lr-krt8.8
MSSLSASYVQHIGSSHSQRQRRRCLVNGSYAAGDGCPSLRGPRTPVAFVPHGGTSRARSWGCPPAPPPEIDRGVLPVRFQEKEQMKTLNDRFVS
FIEKVGILEQQNKVLRVQWLLLQDRGARGSKVEASVFFQSQIHGLQRQLDTLERDKQRRQGELRLAQGLVEDFKNKYEEDISIRNKAENEFVAV
KQDFDDSCLVKAELEARLEGLKDEINFLRGIFEKELRDLEPQIKDTLVSAGLDTSRSIDLQGMISDVRAQYELIAAKSQAEANEFYRKKYDVLS
LSADPTDQEIRSNRNEINNLHDQIQRVRGESNALKKQRASLQAAIAGAVDRGGLSVRVAKERIARLEEELHKDKRQMVQRVREYQELMNVKLAL
DIEIATYRRLLEGEESRLGCVKSQKQHFSLGFGSDLVNKGHRGSFQATTNFDKVINLYKPPKKTVEIRTTETRNGRILSASSEFIH

>Lr-krt8.9
MSYSIKSSGVSSVSRSRSSGGSSIRIGGGGGGGRSGGFSSHSQGFGGGGGSRSRSSYKSGGSSYGGGGGGFGGISASFSSQVAAGPPPEIDRSI
LPVRNQEKEQIKGLNDRFANFIDKVRFLEQQNKVLEAQWRALQERGSGGSNLDSLFEAYINGLKQHLDGLGNEKQRLQGELQHMQGAVEDFKAK
YEEEINTRTQRENEFVVVKKEVDDAFLNKVELEARLDGLNDEIEFLKKIFAEELRQLESQIKDTSLFVSVDTSRNLDVDGLIADVRAQYEAMAR
KSQAEADDFYKKKITELSSSAGKGDDDVRNSRNEINELNRQMQRIKAEIEALKKQRAQLEAAISEAEGRGEMSIKEAKETIARLEEELHKAKQQ
MAQHVREYQELMNVKLALDIEIATYRKLLEGEESRLNAVASGIANLAGAMSGGGGGGGYGFSSGGGGGGGGGSSYGFSSGGGGGGGGGSGFSYES
SSMSSKRSY

>Lr-krt8.10
MSYSSMMQSSSSSGAVSSLQSPMERMGLQDQRSPFAMTAAGGSGGGYSSTSTTTVQGSAYISSGMSSGISVEVDKLQQVRQMEKNQLMGLNDRF
ANYIQNVRILERKNKELQVSLKRMRIHSGQDSQLDNLCAQYEVLLKRQIQALIEEKRRLANDQEQAHNNMEQMKIKYETEIRERSQIENEFVVL
KQEADQVYMQKVELEARLTGLTDDIDFYRKVYEREIRELEARMVKVDVVVEVDSSPGLDLDAYLAEVREQYMKQAVRIREELKISFETKLDANR
SNEVKINDDMRLVTIEISEVRRNMQRYKAELEALKQQCLALERAIALAEEKGKDTIRQLTEKRQLLEVTIMEQRSKLTGHHRSYQELMNVKLAL
DMEIIAYRKLLEGEEGRITTTRIGSTATEYSPSPSPLETKPQFQGYASQQVLIKQIETLDGRVVSESQDMQVIRK

>Lr-krt8.11
MSSNFPNFATTAGFVRGVGPHQGGGSGRGGSGSGGQFTSRSFAGLDSSQPRSERGTWSKSSVPGVVGRRSALLLGYRGGGGGGGGSAYTGGSAY
TGGSAYTGGSAYTGGSAYTGGYGRGSSNVRARVPVAVVEIDRSVLPIRAQEKEEIKMLNNRFANFIDKVRILEQQNKVLEAQWYVLQDKGKVDC
NMDELFEVYIEELRRQLQSLGHNGARMNSDLQMIKHAGKDFSSKYQVEVATRKKKVEEHELVKKEAVEASFVKIELEAKIQGLIEDIHFLREAF
AQELRELEAQIKNTNVLVEIDNRRSLDMDGIISEVRKQYEFIMSQSQAEAEAIYKQKFDKLRSESDRSGEELRKAKTEINDIKRQIHQINSELD
ILKKQREKLEAAISEREEGGAKNIGEAKAAVAKLEEALATAKHDMVKHVRDFTDLMNIKLALDTEILTYRKLLEGEEARLNIFASRGGGGVASS
SPEPGGGVRERRGGSYGQHIGGRHGKDSKSNSSSSNNNNAYDSLDNGVFEASARMNGTLGRSVTVASEPPEQEFRKTVVIKTIETKNGRVVSES
SAVILD

>Ea-krt8.1
MSHMSIQPQRMLTDYADLLKICQVGWGSSVNSNFQVFPQILNGVQVWALAGPLKDFHALVLKPFQCCFGRVLGVIVLLERKSSPQSKHRVDSED
LRNTIVFREQEVKSSTRTSGRGPSSSIRLGTGVSRSGFSSLSMGTGNRMGLSSGRRSMIGSSYRSGSGYTGSLMGQGFGYGSGGNITSATAAML
AAASTQKIEIDRSVLPVRAQEKEQIKTLNNRFASLIDKVRYLEQHNKVLDAQWRTLQERSTATSNVDAMFEAYINGLKNQLEGLGTDKLRLNGE
LQQMQALVEDFKVKYEQEITNRTQVENEFVVVKKDVDDAYLAKVELEAKLEGLQDEINFLKEVFSEELRQLEAQIRDTSLYVEVDTRRNLDING
LIADVRAQYDTIAAKSRADAEDFYKVKFADLTSMTGKTDDEMRMMKGEMNDLNRQIQRINAEIAALKKQRAQLEAAIADAEGRGEVDIREAKET
IARLENDLQLAKQEMAKHVREYQELMNVKLALDIEIATYRKLLEGEECRLNNISIASSGFGVGGVGGVGGVGGGGGIGGAGLLGFSTGGGGMSY
STSGGSMSLASSAMQLESSSMKRSF

>Ea-krt8.2
MCCCPKCACNGHSDDAMMSPRSTPHVCHRRIQIDPSNPAPICIPKLEVDPIHPAPGIRLDRSGEFARSQEKDALKCLNDRFASFIDKVRYLEHQ
NRVLETQWQCLQRRNVTADADCILGAYTSKLKQEQEGILAERPRLQSELSQTVTLVDELKSKYQKENDLRIDLENEYMRTKSAVDESHISKVAM
ETRLNELNSQLEFLRHLFNAERHEMEQRVQDMTVTLELEDTGFNFNLESLVDEVRANYGLIASRSRQEVECWYKSKITDMNESTERNCRELRNA
KSEIEELRHRVQRFRCEMDTLQRQRKQMESAIQEAEERGQASVREGKSAITTLEDELAKAKNDMARHVRDYQELMNIKLALDVEIATYRKLLEG
EENRMVRVSQFSC

>Ea-krt8.3
MQVHGTPARSPAMHSSSAGMSSKNKRSSSRSSFSSRSHGTAGVRGYTPVGYGRASLRPGSVSAGTLFGGTLLFQGAAAKVDIDPTMLPTRTQEK
EAIKKLNDRFASFIDKVRFLEQQKTVLEAQWKALQERGSASLHLDNLFEPYVALLRQRFDELALGQPRLESELQQMQGAVEDLRCKYEGEITTH
SQMENEFVVVKKEVDESYLMKVELEGILDGLTEDISFYKEIFAEEMHQLEAQIRDTSLYIEVDTRRNLDIGGLIADVRAQYETIAAKSKAEAEE
FYREKFVSFNASAGKNEEELRLIKHDISESLRQLQRVKAEIEALKKQQAHLETAIAETEERGEMDIREAKSTMSHLENELQCTKQEMAKHVRDY
QELMNVKLALDIEIATYRKLLEGEECRLGVLASAFTSGGVTYGTSSHNPSGGYFSQEASHSNALAFHSSGNAFGSHGSSFAQAVQNPHSAQSKK
TVVIKTIETKDGQVVSESSEVVEQ

>Ea-krt8.4
MADRRSLSMRSKTSQYRSTSGSSGGGGGMRQSSRGGTSVRYGKGSSISGFPSRKGGSSMSMSGMGNNRASRSALKAAYCGGGAGMGGGITRSIG
ALNLHSTVPPNINMDVVPIRKNEKEQIKGLNDKFATFIGKVQQLEQQKQVLEAQWRALQQRGGASVNVDGMFQSYINSLKQQLEGLGQDKMRLH
GELQQMQGLVEDFKSKYEEEINNRTQKENEFVVVKKDVDDAYLAKVELEAKLDGLQDEIKFLKDIYAEEISQLEQQIKDTSLFVSVDTTRNLNV
ADIINESQRHYDDIAENSKREASRFYQEKLNEQVSCAPSGEDDIRKCRSEVNELNQSMQHMKAEIENLKKRRANLEAQIAEAEEQGDVQIAETK
VQITELEKDLHKCKDDMAAQLKEYHALMNVKLALDIEIATYRSLLEQEENRINNISYGGGGGGENYYLAVVLVEVVVLAVVLVEVVRHFPQVPR
AVQTMLEDNLMSRQSRDFRRDKTRK

>Ea-krt8.5
MSTRSCNIRPPAAASCRLNFPQPCAAPTVSIPKVTLDPTCNVPFVEIDKNAQALRNQEKDALKVLNDRFASFIERVRILEQQNKILQAQDNHFQ
TSGSDTNVGAMFEAFIRSLRKKLDCITGDKPKLESELQQMLAVVDELKVKYEKEYDLRTKAENEFVATKKDVDAAYLEKIGLETRAAQLLDEIN
FLREVFDTEICELQDRTKDTGVQVTISQGLNADLSTLIAEVRAQYEAIAAKSRQEVECWFKGKVDSLNESTERNNAEMRASKSEITEITRQIQR
IRAEMDVQKKQRTQIEQAIKEAEDRGSISVREGKEAIGRLEKELQQSRQEMARHVRDYQELMNVKLALDVEIATYRKLLEGEESRLSKVYEKRC
Y

>Ea-krt8.6
MASTGLALGLLLPLDAGFLEGITLDAGGVQEKGRRAQEARLQEQASLKSLNNQFAGFIDKVRHLEQQNKVLEAQLQALQHQESQGSRFYKLLEC
YSEDLRKNNDQVTKENERMQQENEEVQGLVENLRNKFESQRNSRIEVENRLQDIKRETDSAFVSKADVEANIELTSTDIQFMSQIFCEEEAQLQ
AKSKSVTVEVDTSRSLDLSNIIDDVREQYEANLMKMQQEAEIYYKNKLDDAATMARNISSELENTQGEIDNLNKQLEFVTATTEALKNERVELE
AVVIQAEENGENDVAAARSHCATLEEDIKASKQEMALQVQEHQELMNVKLALDVEIATYRKLLEGEENRLRKAQGICRVGSETVCNAKTSGCGD
GVTVHHTSSRISTPSMGRCMTKGSANERLRSPVHKCPSSCRTTSTGGNHPHSPTECGSTQSKKTIALGFTRTSTSKTGALDWSSNSNSIKASSY
TTSSNANGPPTYIYSRTTKTGGFLNSCKEPMLNKDDLTAKVSTSPRVKGITMSNLSEATFDTIVDESGEERVMEPNGIFGRANEILSKLKASSE
LTRDYLEAFEKGDAAVESYCKNRLQGEEDMFAVLNKQKTKTFISMNKSSKSKVKENEIMIKADRNLFQRLIIISGVKKFDIKKMLTHHHDSALP
LSLINADGFLNNVVKLNLRHVIETTVQQASVMEAIPRGSIWIMDGMAILQELRQKKIPAMFGLLAVYLLQAAAGHGEKCCQ

>Eb-krt8.1
MSVQVKSSTRTSGRGPSSSIRLGTGVGRSGFSSLSMGTANRMGLGSGRRSMIGSSYRSGSGYTGSLMGQGFGYGSGGNITSATAAMLAAASTQK
IEIDRSVLPVRAQEKEQIKTLNNRFASLIDKVRYLEQHNKVLDAQWRTLQERSTATSNVDAMFEAYINGLKNQLEGLGTDKLRLNGELQQMQAL
VEDFKVKYEQEITNRTQVENEFVVVKKDVDDAYLAKVELEAKLEGLQDEINFLKEVFSEELRQLEAQIRDTSLYVEVDTRRNLDINGLIADVRA
QYDAIAAKSRADAEDFYKVKFADLTSMTGKTDDEMRLMKGEMNDLNRQIQRINAEIAALKKQRAQLEAAIADAEGRGEVDIREAKETIARLEND
LQLAKQEMAKHVREYQELMNVKLALDIEIATYRKLLEGEECRLNNISIASSGLGVGGGGGGIGGAGLLGFSTGGGSMSYSTSGGSMSLASTGAM
QLESSSMKRSF

>Eb-krt8.3
MQVHGTPARSPAMHSSSAGMSSKNKRSASRSSFSSRSHGTAGVRGYTPVGYGRASLRPGSVSAGTLFGGTLLFQGGAAKVDIDPTMLPTRTQEK
EAIKKLNDRFASFIDKVRFLEQQKTVLEAQWKALQERGSASLHLDNLFEPYVALLRQRFDELALGQPRLESELQQMQGAVEDLRCKCDIRTCKI
FLYNLSLQEVDESYLMKVELEGILDGLTEDISFYKEIFEMHQLEAQIRDTSLYIEVDTRRNLDIGGLIADVRAQYETIAAKSKAEAEEFYREKF
VSFNASAGKNEEELRLIKHDISESLRQLQRVKAEIEALKKQQAHLETAIAETEERGEMDIREAKSTMSHLENELQCTKQEMAKHVRDYQELMNV
KLALDIEIATYRKLLEGEECSTSFSYGPEASKSYTY

>Eb-krt8.4
MGDRRSLSMRSKTSQYRSTGGSSGGGGGGGMRQSSRGGTSIRLGKGSSVSGFSNRRGASSMSMSGMSNHRASRSALKAAYCGGGGGGMAGGITR
SIGALNLHSTVPPNINMDVVPIRKNEKEQIKGLNDKFATFIGKVQQLEQQKQVLEAQWRALQQRGGASVNVDGMFQSYINSLKQQLEGLGQDKM
RLHGELQQMQGLVEDFKSKYEEEINNRTQKENEFVVVKKDVDDAYLAKVELEAKLDGLQDEIKFLKDIYAEEISQLEQQIKDTSLFVSVDTTRN
LNVADIINESQRHYDDIAESSKREASRFYQEKLNEQVSCAPSGEDDIRKCRSEINELNQSMQHMKAEIENLKKRRANLEAQIAEAEEQGDVQIA

```
ETKAQITELEKDLHKCKDDMAAQLKEYHALMNVKLALDIEIATYRSLLEQEENRINSISYGGGGGGGGGGLFGGIGGGIGGIGGLGGLGGGIGG
FGGSLGGSGSSISTNVQSSGSYVRR

>Eb-krt8.7
MALICSCGSGITRRRYLLYRVPSCNIRPPAATSCRLNFPQPCAAPTVSIPKVTLDPTCNVPFVEIDKNAQALRNQEKDALKVLNDRFASFIERV
RILEQQNKILQAQDNHFQTSGSDTNVGAMFEAFIRTLRKKLDCITGDKPKLESELQQMLAVVDELKVKYEKEYDLRTKAENEFVATKKDVDVAY
LEKIGLETRAAQLLDEINFLREEICELQDRTKDTGVQVTISQGLNADLSTLIAEVRAQYEAIAAKSRQEVECWFKGKVCGKRDKSEITEITRQI
QRIRAEMDVQKKQHTQIEQAIKEAEDRGSISVREGKEAIGRLEKELQQSRQEMARHVRDYQELMNVKLALDVEIATYRKLLEGEESSPHWTLSK
AGAYGFGLDFHVRGCVHVCVRIHISDMYRPILFELSTNTTHDDIPICISFFWHQIQDGRLGGF

>Eb-krt8.8
MSCNTCNPCCPAPCPEPCMPCCIPCPKPCPTPICCNPCPPPSVCIPTLDLDPICPAPCIDIDRSGQFMRSNEKEGMKCGNDRFASFIDKTRFLE
QQNKVLEAQWECLQNKTCTSKLDCMFEQFANRLKEQLECLVQDRPRLETEMNQTQSLANDYRSKYEEEIALRTQLENEFWALKQDVDCKYLEKV
RLETRLAQLNDEVLFLRTLFEQELQEMHERIRDMSVSVELDVAPNLDLTSLIAEVRCNYEAIAARSRQEVECWYKSKLDDINEASERNCCELRC
TKNEVADLARNIQRLRCEIETQARQRSQLEATIQDAEARGQASVLQGRETIARLENELQSAKQEMAKHVRDYQELMNVKLALDVEIATYKKLLE
GEECRLTCVPTGCGVPCRPCDPCGRF

>Eb-krt8.10
MRLYNCQRGGGKNVLAQRMAGSGGRKRVPLLRGHLLGRRCLSAGSQPLGAPSREVRLDSGVPELRTKEKDAIKHLNNRFANLIEKVSTLQQQNK
VLEAQWFALQQKVPADVGVEKGFQKYIDVLQQQLNTLHDKKSQLQSHLLQTRESVQANKGRYETEIDKRTQKDLGLLEIKKDVGIFRFKKTEKE
CEKKILVDEIDFLKMLFAKEIQDLELQIKDASLFVSVDTNHSLDFEGIISEVRAEYDAITAQSQANAQDFYRKKLLDISTSADNANNQLHEAKA
EMVKMSLQIKRMKAQLDSLKKQL
```

Figure S2. Amino acid sequences of type II keratins of lampreys and hagfishes investigated in this study. Species abbreviations: Ea*, Eptatretus atami*; Eb, *Eptatretus burgeri*; Lr, *Lethenteron reissneri*; Pm, *Petromyzon marinus*.

```
                        1                                                                                              100
Pm_krt18.8              MSCSVRSCLAPGRGFSSRSVGVTSRPFRSGFTAWSSAGGSALGGLGGARSAFCYPSRGAPCRPRMRAPTPCMPTVPCGPLFGPCGPLTAAAAPCGVLLTN
Lr_krt18.1              MSCSVRSCLAPGRGFSSRSVGVTSRPFRSGFTAWSSAGGSALGGLGGARSAFCYPSRGAPCRPRMRPPTPCMPTVPCGPLFGPCGPLTAAAAPCGVLLTN
Lr gDNA chr67           ----------------------------------------------------------------------------------------------------
SRR9964081.92223952.1   MSCSVRSCLAPGRGFSSRSVGVTSRPFRSGFTAWSSAGGSALGGL-------------------------------------------------------
SRR9964081.146725637.1  ------SCLAPGRGFSSRSVGVTSRPFRSGFTAWSSAGGSALGGLGGARSAFCYPS--------------------------------------------
SRR9964081.144473315.2  --------------------GVTSRPFRSGFTAWSSAGGSALGGLGGARSAFCYPSRGAPCRPRMRPPTP------------------------------
SRR9964081.94190217.2   ----------------------------------------------------------APCRPRMRPPTPCMPTVPCGPLFGPCGPLTAAAAPCGVLLTN

                        101                                                                                            200
Pm_krt18.8              EKEALQDLNERLAGYLDKVRYLEQANQELECKIKEFRANSCIAEQQWEPCMGNIEELMGQVEVATLENTRLVLEIDNARLASEDFRVKWEAESALRETVE
Lr_krt18.1              EKEALQDLNERLAGYLDKVRYLEQANQELECKIKEFRANSCIAEQQWEPCMGNIEELMGQVEVATLENTRLVLEIDNARLASEDFRIKWEAESALRETVE
Lr gDNA chr67           ------------------------------------------------------------VEVATLENTRLVLEIDNARLASEDFRIKWEAESALRETVE
SRR9964081.94190217.2   EKEALQD---------------------------------------------------------------------------------------------
SRR9964081.13621348.2   -----QDLNERLAGYLDKVRYLEQANQELECKIKEFRANSCIAEQQWEPCMGNIE---------------------------------------------
SRR9964081.96610813.1   ----------RLAGYLDKVRYLEQANQELECKIKEFRANSCIAEQQWEPCMGNIEELMGQ----------------------------------------
SRR9964081.45440897.2   ------------------------------------------------------------VEVATLENTRLVLEIDNARLASEDFRIK------------

                        201                                                                                            300
Pm_krt18.8              ADAHELRRLSAEYCACRNQLACEQNVLQGEIHTLKSHHRGEMESLRMDYSSSTTKVEMDNSPGADTAGIISEIRAQYETMIHNNRHEAECMLQSKLEAAE
Lr_krt18.1              ADAHELRRLSAEYCACRNQLACEQNVLQGEIHTLKDHHRGEMESLRMDYSSSTTKVEMDNSPGADTAGIISEIRAQYETMIHNNRHEAECMLHSKLEAAE
Lr gDNA chr67           ADAHELRRLSAEYCACRNQLACEQNVLQGEIHTLKDHHRGEMESLRMDYSSSTTKVEMDNSPGADTAGIISEIRAQYETMIHNNRHEAECMLHSKLEAAE

                        301                                                                                            400
Pm_krt18.8              ASAVHSHGELAAAKNQAHHLRQQYQTMEVEMESLRSANATLEDNLAETEERFSVEVRSLAEVLSRLEAEYSDVRANVERQLHEYESLLNIKMGLEMEIST
Lr_krt18.1              ASAVHSHGELSAAKSQAHHLRQQFQTLEVEMESLRSANATLEDNLAETEERFSVEVRSLAEVLSRLEAEYSDVRANVERQLHEYESLLNIKMGLEMEIST
Lr gDNA chr67          ASAVHSHGELSAAKSQAHHLRQQFQTLEVEMESLRSANATLEDNLAETEERFSVEVRSLAEVLSRLEAEYSDVRANVERQLHEYESLLNIKMGLEMEIST

                        401                                                       458
Pm_krt18.8              YKCLIEGEDSRLSSEVRCNPACCGSSRVCTSLPMPCGSDPCSLNVCSSGPVCTMPCLK
Lr_krt18.1              YKCLIEGEDSRLSSEVRCNPACCGSSRVCSSLPMPCGKDPCSLNVCSSGPVCTMPCLK
Lr gDNA chr67           YKCLIEGEDSRLSSEVRCNPACCGSSRVCSSLPMPCGKDPCSLNVCSSGPVCTMPCLK
```

Figure S3. Assembly of the amino acid sequence of krt18.1 of *Lethenteron reissneri*. krt18.8 of the sea lamprey (*Petromyzon marinus*, Pm) was submitted to tBLASTn against the transcriptomic sequence read archive (SRA) of an oral gland sample (run accession: SRR9964081) of *Lethenteron reissneri* (Lr). The amino acid sequences of BLAST hits were aligned and, together with the sequence of a partial krt18.1 prediction, used to assemble the complete amino acid sequence of the protein. The sequence encoding the partial *krt18.1* gene of *Lethenteron reissneri* is located on chromosome 67 (chr67), GenBank accession number NC_083845.1 (4793998..4810161, complement). Cysteine residues are highlighted in red. The intermediate filament domain is marked by a line above the sequences.

```
            1                                                                                                  100
Pm_krt18.8  MSCSVRSCLAPGRGFSSRSVGVTSRPFRSGFTAWSSAGGSALGGLGGARSAFCYPSRGAPCRPRMRAPTPCMPTVPCGPLFG-PCGPLTAAAAPCGVLLT
Lr_krt18.1  MSCSVRSCLAPGRGFSSRSVGVTSRPFRSGFTAWSSAGGSALGGLGGARSAFCYPSRGAPCRPRMRPPTPCMPTVPCGPLFG-PCGPLTAAAAPCGVLLT
Ea_krt18.1  MSCSAHSCSASQRNFSSRSCASLLGGKRAPRCTAFSLCRSTTGR--PTRSSICNPCCN---RPGICLPRPCLPPVPCGPCGSFPCGPLTSAAAPCGVLLT
Eb_krt18.9  MSCSAHSCSASQRNFSSRSCASLLGGKRAPRCTAFSLCRSTSGR--PTRSSICNPCCN---RPGICLPRPCLPPVPCGPCGSFPCGPLTSAAAPCGVLLT
Ea_krt18.5  MSCSIRSCYSTPHRYSSRSCASLLGGKRAPRCTAFSVCRNPCGR--PTRSSICNPCCN---RPGICLPRPCLPPVPCGPCGSFPCGALTSAAVPCGVLLT
Eb_krt18.8  MSCSIRSCYSTPHRYSSRSCASLLGGKRAPRCTTFSVCRNPCGR--PTRSSICNPCCN---RPGICLPRPCLPPVPCGPCGSFPCGALTSAAVPCGVLLT
            **** :** :  : :**** .      *: :                :**::* *.      ** :  * **:* *****    *** **:**.*******

            101                                                                                                200
Pm_krt18.8  NEKEALQDLNERLAGYLDKVRYLEQANQELECKIKEFRANSCIAEQQWEPCMGNIEELMGQVEVATLENTRLVLEIDNARLASEDFRVKWEAESALRETV
Lr_krt18.1  NEKEALQDLNERLAGYLDKVRYLEQANQELECKIKEFRANSCIAEQQWEPCMGNIEELMGQVEVATLENTRLVLEIDNARLASEDFRIKWEAESALRETV
Ea_krt18.1  NEKQTMQDLNDRLSGYLDKVRYLEATNLELECKIKEFRANKCIAEREWDPCLSGIESLMYQIEEASVENSQLVLAIDNARLTAEDFRTKWEIEVSLREDL
Eb_krt18.9  NEKQTMQDLNDRLSGYLDKVRYLEATNLELECKIKEFRANKCIAEREWDPCLSGIESLMYQIEEASVENSQLVLAIDNARLTAEDFRTKWEIEVSLREDL
Ea_krt18.5  NEKQTMQDLNDRLSSYLDKVRYLEATNLELECKIKEFRANKCIAEREWDPCLSGIESLMYQIEEASVENSQLVLAIDNARLTAEDFRKKWEIEVSLREEL
Eb_krt18.8  NEKQTMQDLNDRLSSYLDKVRYLEATNLELECKIKEFRANKCIAEREWDPCLSGIESLMYQIEEASVENSQLVLAIDNARLTAEDFRKKWEIEVSLREEL
            ***:::****:**:.*********  :* ************.****::*:**:..**.** *:* *::**::*** ******::**** *** * :*** :

            201                                                                                                300
Pm_krt18.8  EADAHELRRLSAEYCACRNQLACEQNVLQGEIHTLKSHHRGEMESLRMDYSSSTTKVEMDNSPGADTAGIISEIRAQYETMIHNNRHEAECMLQSKLEAA
Lr_krt18.1  EADAHELRRLSAEYCACRNQLACEQNVLQGEIHTLKDHHRGEMESLRMDYSSSTTKVEMDNSPGADTAGIISEIRAQYETMIHNNRHEAECMLHSKLEAA
Ea_krt18.1  EIDIQDLRNLANEYCECRNTVACEINVLEDEICHLKNSHREEIEALRHEYANSTLNVDIDHTPGVDLAGIINDIRTQYETMISSTHYEVECLIQNKLDAA
Eb_krt18.9  EIDIQDLRNLANEYCECRNTVACEINVLEDEICHLKNSHREEIEALRHEYANSTLNVDIDHTPGVDLAGIINDIRTQYETMIASTHYEVECLIQNKLDAA
Ea_krt18.5  ETDIQDLRNLANEYCECHNTVACEVNVIQDEIYYLKQSHREEIEGLRCEYANSTLNVDIDHSPNVDLSSIINDIRTQYETMIGNTHYEVECLMNNKLEAS
Eb_krt18.8  ETDIQDLRNLANEYCECHNTVACEVNVIQDEIYYLKQSHREEIEGLRCEYANSTLNVDIDHSPNVDLSSIINDIRTQYETMIGNTHYEVECLMNNKLEAS
            * * ::**.*: *** *:* :*** **::.**  **. ** *:*.** :*:.** :*::*::*..* :.**.:**:****** ..::*.**:::.**:*:

            301                                                                                                400
Pm_krt18.8  EASAVHSHGELAAAKNQAHHLRQQYQTMEVEMESLRSANATLEDNLAETEERFSVEVRSLAEVLSRLEAEYSDVRANVERQLHEYESLLNIKMGLEMEIS
Lr_krt18.1  EASAVHSHGELSAAKSQAHHLRQQFQTLEVEMESLRSANATLEDNLAETEERFSVEVRSLAEVLSRLEAEYSDVRANVERQLHEYESLLNIKMGLEMEIS
Ea_krt18.1  QASAHQSACNLEAIKTEANNLRYEFQALEVEAESLRSANCILEENLHETEARLNLEISNLAQIISILEADYTDVRSNIERQIREYESLLNVKMQLEMEIA
Eb_krt18.9  QASAHQSACNLEAVKTEANNLRYEFQALEVEAESLRSANCILEENLHETEARLNLEISNLAQIISILEADYADVRSNIERQIREYESLLNVKMQLEMEIA
Ea_krt18.5  QASAHQSACELEATKNEANNLRYEFQSLEVEAESLHTANCTLEENLHETEERLNLELSNLAEIISSLEAEYTDVRANIERQIREYESLLNVKMQLEMEIG
Eb_krt18.8  QASAHQSACELEATKNEANNMRYEFQSLEVEAESLHTANCTLEENLRETEERLNLELSNLAEIISSLEAEYTDVRANIERQIREYESLLNVKMQLEMEIG
            :*** :*  :* * *.:*:::* ::*::*** ***::**. **:** *** *:.:*: .**:::* ***:*:***:*:***::*******:** *****.

            401                                                    467
Pm_krt18.8  TYKCLIEGEDSRLSSEVRCNPACCGSSRVCTSLPMPCGSDPCSLNVCSSGPVCTMPCLK--------
Lr_krt18.1  TYKCLIEGEDSRLSSEVRCNPACCGSSRVCSSLPMPCGKDPCSLNVCSSGPVCTMPCLK--------
Ea_krt18.1  TYQQLIDCEDSRLAN-VSCGRVCYGDAVRCTALPVPCGANPCSLNVCPSEPKCGGPCGVCGPCLPCA
Eb_krt18.9  TYQQLIDCEDSRLAN-VSCGRVCYGDAVRCTALPVPCGANPCSLNVCPSEPKCGGPCGVCGPCLPCA
Ea_krt18.5  TYQQLIEGEDCRMSN-VVCGPVCCGSAMRCRALPVPCGANPCSLNVCPSEPKCG-PCL---------
Eb_krt18.8  TYQKLIEGEDCRMSN-VVCGPVCCGSAMRCRALPVPCGANPCSLNVCPSEPKCG-PCL---------
            **: **: **.*::  * *. .* *.:  * :**:*** :******* * * *  **
```

Figure S4. Conservation of cysteine-rich type I keratins in cyclostomes. Amino acid sequence alignments of cysteine-rich type I keratins. Cysteine residues are highlighted by red shading. “*”, “:” and “.” indicate identical, highly similar and weakly similar, respectively, amino acid residues in all sequences. Species abbreviations: Ea, *Eptatretus atami*; Eb, *Eptatretus burgeri*; Hs, *Homo sapiens*; Lr, *Lethenteron reissneri*; Pm, *Petromyzon marinus*.

```
           1                                                                                                100
Pm_krt8.9  MSYSKPCEPCVPNKYVSNFAPTPKLPKPCAPCHAPCPAPALCPRPCPPRTTVRCCTPGPEPCHDSGHGFSSLAPFIHGGAFYTGGAAHGSKPSCSSYVPF
Lr_krt8.6  MSYSKPCEPCVPNKYVSNFAPTPKLNKPCAPCHAPCPAPALCPRPCPPRTTVRCCTPGPEPCHDSGHGFSSLAPFIHGGGFYTAGAAHGSKPSCSSYVPF
Lr_krt8.7  MSYSKPCEPCVPNKYVSNFAPTPKLNKPCAPCHAPCPAPALCPRPCPPRTTVRCCTPGPEPCHDSGHGFSSLAPFIHGGGFYTAGAAHGSKPSCSSYVPF
Ea_krt8.2  MCC---CPKCACN-GHSDDA--------------------------------------------------------------------------------
Eb_krt8.8  MSCNT-CNPCCPAPCPEPCMP-------------------------------------------------------------------------------
           *.    *  *

           101                                                                                              200
Pm_krt8.9  TSAPCGPPPCGPVHHGSFHAPCGPAPCAPVPKCSKAVP-VCPRPCPPPVCPTPCPRPSACIPRVSMEPLCPAPCVQIDRSGQNVRCQEKNAISHLNDRFA
Lr_krt8.6  TSAPCGPAPCGPVHHTDFHAPCGPAPCAPKPKCTKAVP-VCPRPCPPPVCPTPCPRPSACIPRISMEPLCPAPCVQIDRSGQNVRCQEKNAISHLNDRFA
Lr_krt8.7  TSAPCGPAPCGPVHHTDFHAPCGPAPCAPKPKCTKAVP-VCPRPCPPPVCPTPCPRPSACIPRISMEPLCPAPCVQIDRSGQNVRCQEKNAISHLNDRFA
Ea_krt8.2  ----------------------------------MMSPRSTPHVCHRRIQIDPSNPAPICIPKLEVDPIHPAPGIRLDRSGEFARSQEKDALKCLNDRFA
Eb_krt8.8  ----------------------------------CCIP--CPKPCPTPICCNPCPPPSVCIPTLDLDPICPAPCIDIDRSGQFMRSNEKEGMKCGNDRFA
                                                 *   *: *   :   *.     *** :.::*: *** : :****:  *.:**:.:.  *****

           201                                                                                              300
Pm_krt8.9  SFIDKVRYLEQENKVLEAQWGCLQARVCTSDLDSMFETYAQAIKRQLDCVLADRPRLETELHQTRALADEHKLKYEAEVTGREAAEANFVAIKREADDNY
Lr_krt8.6  SFIDKVRYLEQENKVLEAQWGCLQARVCTSDLDSMFETYAQAIKRQLDCVLADRPRLETELHQTRALADEHKLKYEAEVTGREAAEANFVAIKREADDNY
Lr_krt8.7  SFIDKVRYLEQENKVLEAQWGCLQARVCTSDLDSMFETYAQAIKRQLDCVLADRPRLETELHQTRALADEHKLKYEAEVTGREAAEANFVAIKREADDNY
Ea_krt8.2  SFIDKVRYLEHQNRVLETQWQCLQRRNVTADADCILGAYTSKLKQEQEGILAERPRLQSELSQTVTLVDELKSKYQKENDLRIDLENEYMRTKSAVDESH
Eb_krt8.8  SFIDKTRFLEQQNKVLEAQWECLQNKTCTSKLDCMFEQFANRLKEQLECLVQDRPRLETEMNQTQSLANDYRSKYEEEIALRTQLENEFWALKQDVDCKY
           *****.*:**::*:***:** *** :  *:. *.::  ::. :*.: : :: :****::*: ** :*.:: : **: *   *   * ::   *  .* .:

           301                                                                                              400
Pm_krt8.9  MGKVQMETRVGQLSDELHFIKELFACEMQEMEERIRDMNVTIDL-DTGCNFDLSSLIAEVRANYEMVAARSREEVECWYKSKMDDMAEASERHCIELRCT
Lr_krt8.6  MGKVQMETRVGQLSDELHFIKELFACEMQEMEERIRDMNVTIDL-DTGCNFDLSSLIAEVRANYEMVAARSREEVECWYKSKMDDMAEASERHCIELRCT
Lr_krt8.7  MGKVQMETRVGQLSDELHFIKELFACEMQEMEERIRDMNVTIDL-DTGCNFDLSSLIAEVRANYEMVAARSREEVECWYKSKMDDMAEASERHCIELRCT
Ea_krt8.2  ISKVAMETRLNELNSQLEFLRHLFNAERHEMEQRVQDMTVTLELEDTGFNFNLESLVDEVRANYGLIASRSRQEVECWYKSKITDMNESTERNCRELRNA
Eb_krt8.8  LEKVRLETRLAQLNDEVLFLRTLFEQELQEMHERIRDMSVSVEL-DVAPNLDLTSLIAEVRCNYEAIAARSRQEVECWYKSKLDDINEASERNCCELRCT
           : ** :***: :*..:: *:: **  * :**.:*::**.*:::* *.. *::* **: ***.**  :*:***:*********: *: *::**:* *** :

           401                                                                                              500
Pm_krt8.9  KNEILELSSMLQRLACEIDGLKTQRYQLESSIQEAETRGELNAHEARDATARVEMELSQAKADMARHVRDYQELMNVKLALDIEIATYRKLLEGEESRLH
Lr_krt8.6  KNEILELSSMVQRLACEIDGLKTQRCQLESSIQEAEHHGEMNAHEARDATARVETELSQAKADMARHVRDYQELMNVKLALDIEIATYRKLLEGEESRLH
Lr_krt8.7  KNEILELSSIVQRLACEIDGLKTQRCQLESSIQEAEHHGEMNAHEARDATARVETELSQAKADMARHVRDYQELMNVKLALDIEIATYRKLLEGEESRLH
Ea_krt8.2  KSEIEELRHRVQRFRCEMDTLQRQRKQMESAIQEAEERGQASVREGKSAITTLEDELAKAKNDMARHVRDYQELMNIKLALDVEIATYRKLLEGEENRMV
Eb_krt8.8  KNEVADLARNIQRLRCEIETQARQRSQLEATIQDAEARGQASVLQGRETIARLENELQSAKQEMAKHVRDYQELMNVKLALDVEIATYKKLLEGEECRLT
           *.*: :*   :**: **::    ** *:*::**:** :*: .. :.:.: : :* ** .** :**:*********:*****:*****:*******  *:

           501                 523
Pm_krt8.9  CVPDRCGQPCMPCDPCGPSRRCL
Lr_krt8.6  CVPDRCGQPCMPCDPCGPSRRCL
Lr_krt8.7  CVPDRCGQPCMPCDPCGPSRRCL
Ea_krt8.2  RVSQFSC----------------
Eb_krt8.8  CVPTGCGVPCRPCDPCGRF----
            *   .
```

Figure S5. Conservation of cysteine-rich type II keratins in cyclostomes. Amino acid sequence alignments of cysteine-rich type II keratins. Cysteine residues are highlighted by red shading. “*”, “:” and “.” indicate identical, highly similar and weakly similar, respectively, amino acid residues in all sequences. Species abbreviations: Ea, *Eptatretus atami*; Eb, *Eptatretus burgeri*; Hs, *Homo sapiens*; Lr, *Lethenteron reissneri*; Pm, *Petromyzon marinus*.

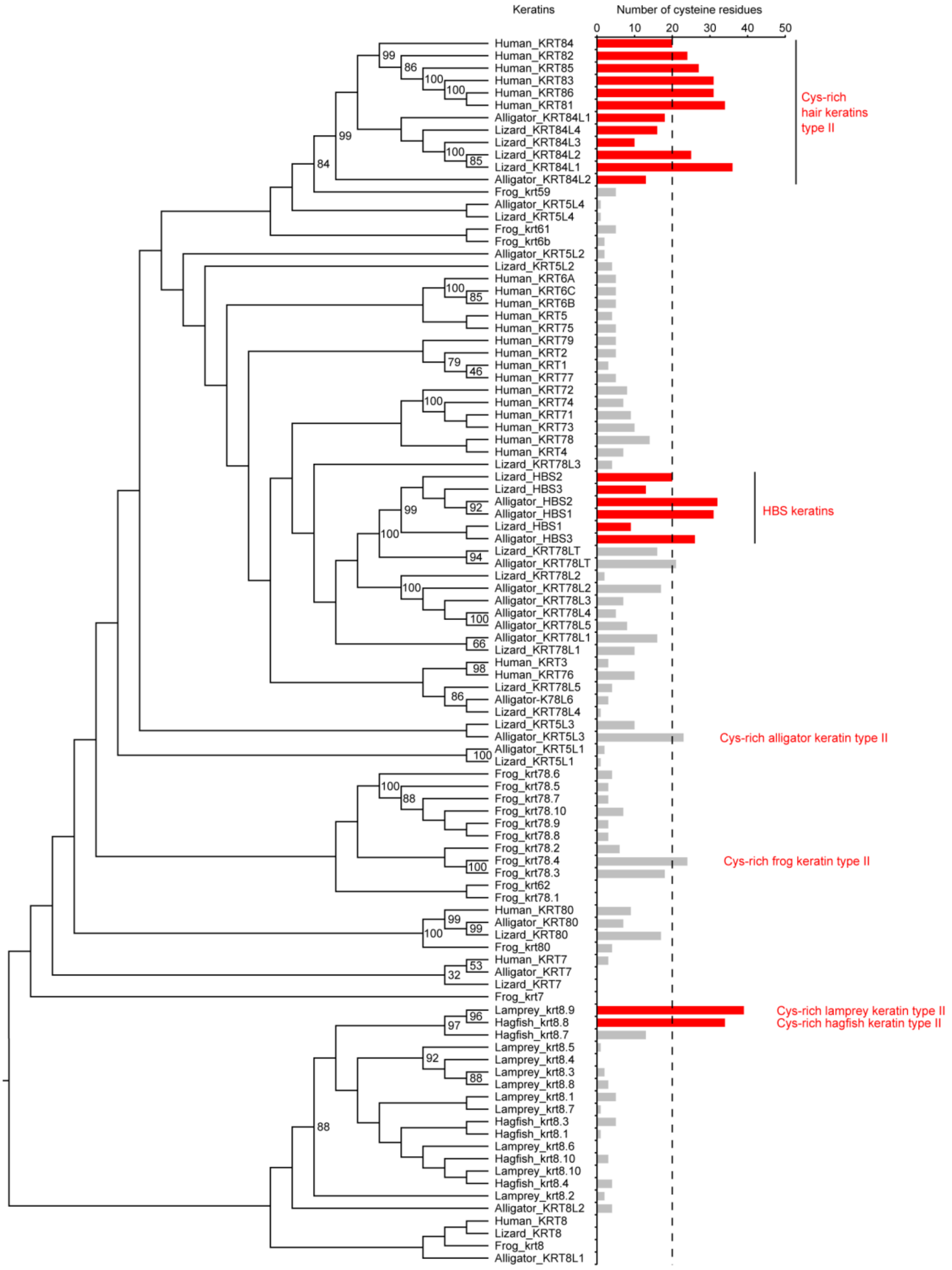

Figure S6. Phylogenetic analysis of type II keratins of cyclostomes and tetrapods. A phylogenetic tree of type II keratins was inferred using the maximum likelihood method. Bootstrap values ≥ 80 are shown. The number of cysteine residues of each keratin is indicated by a bar chart on the right. Hair keratis, hard basic sauropsid specific (HBS) keratins and the cysteine-rich type II keratins of cyclostomes are highlighted in red. The dashed line indicates the minimum number of cysteine residues (n=20) in mammalian hair keratins, which is used the threshold for the definition of cysteine-rich keratins. Species: Alligator (*Alligator sinensis*), frog (*Xenopus tropicalis*), hagfish (*Eptatretus burgeri*), human (*Homo sapiens*), lamprey (*Petromyzon marinus*), lizard (*Anolis carolinensis*).

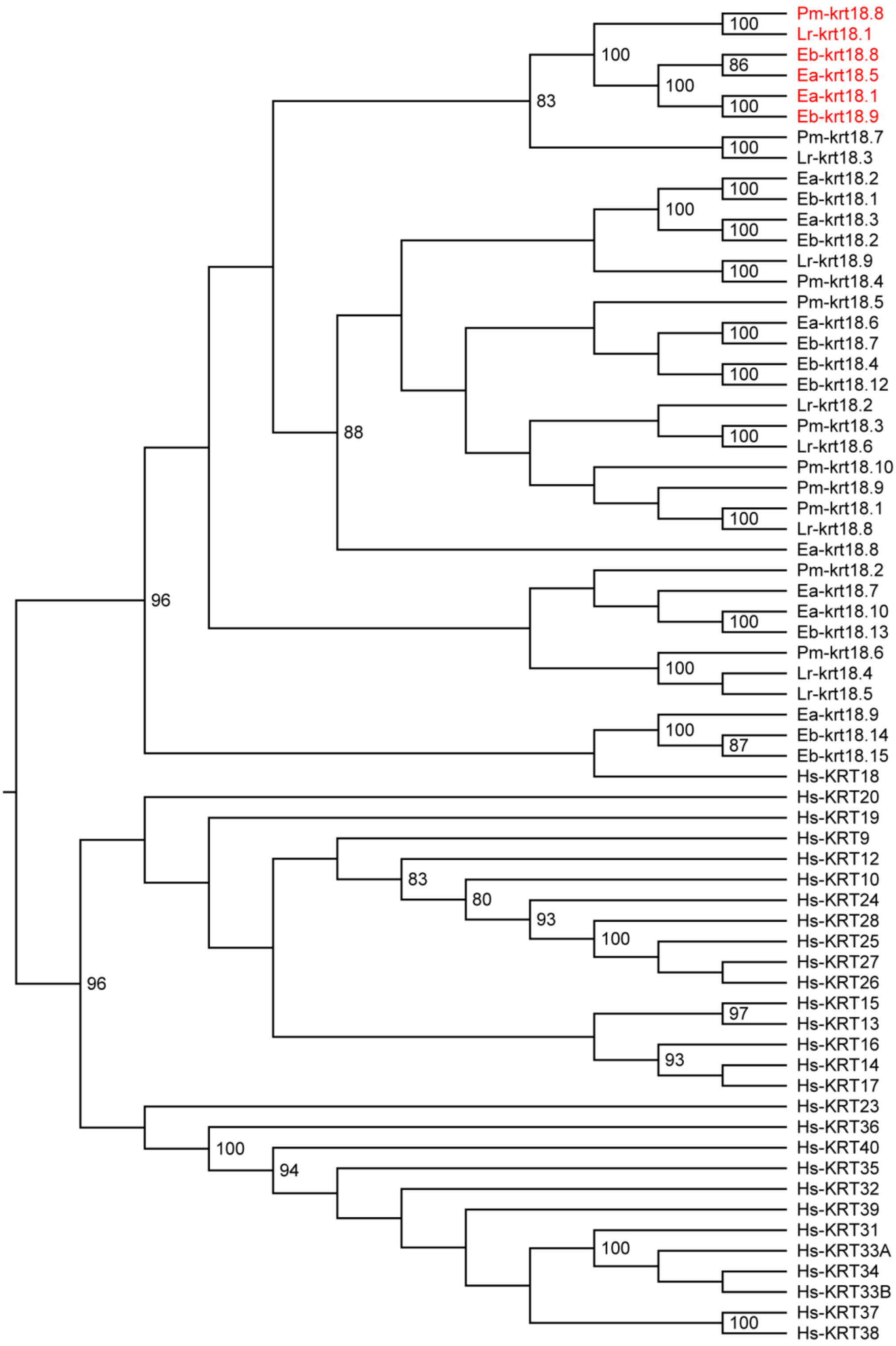

Figure S7. Phylogenetic analysis of type I keratins of four species of cyclostomes and humans. Bootstrap values ≥80 are shown. Species abbreviations: Ea*, Eptatretus atami*; Eb, *Eptatretus burgeri*; Hs, *Homo sapiens*; Lr, *Lethenteron reissneri*; Pm, *Petromyzon marinus*. Note that all cyclostome type I keratins cluster together with human KRT18.

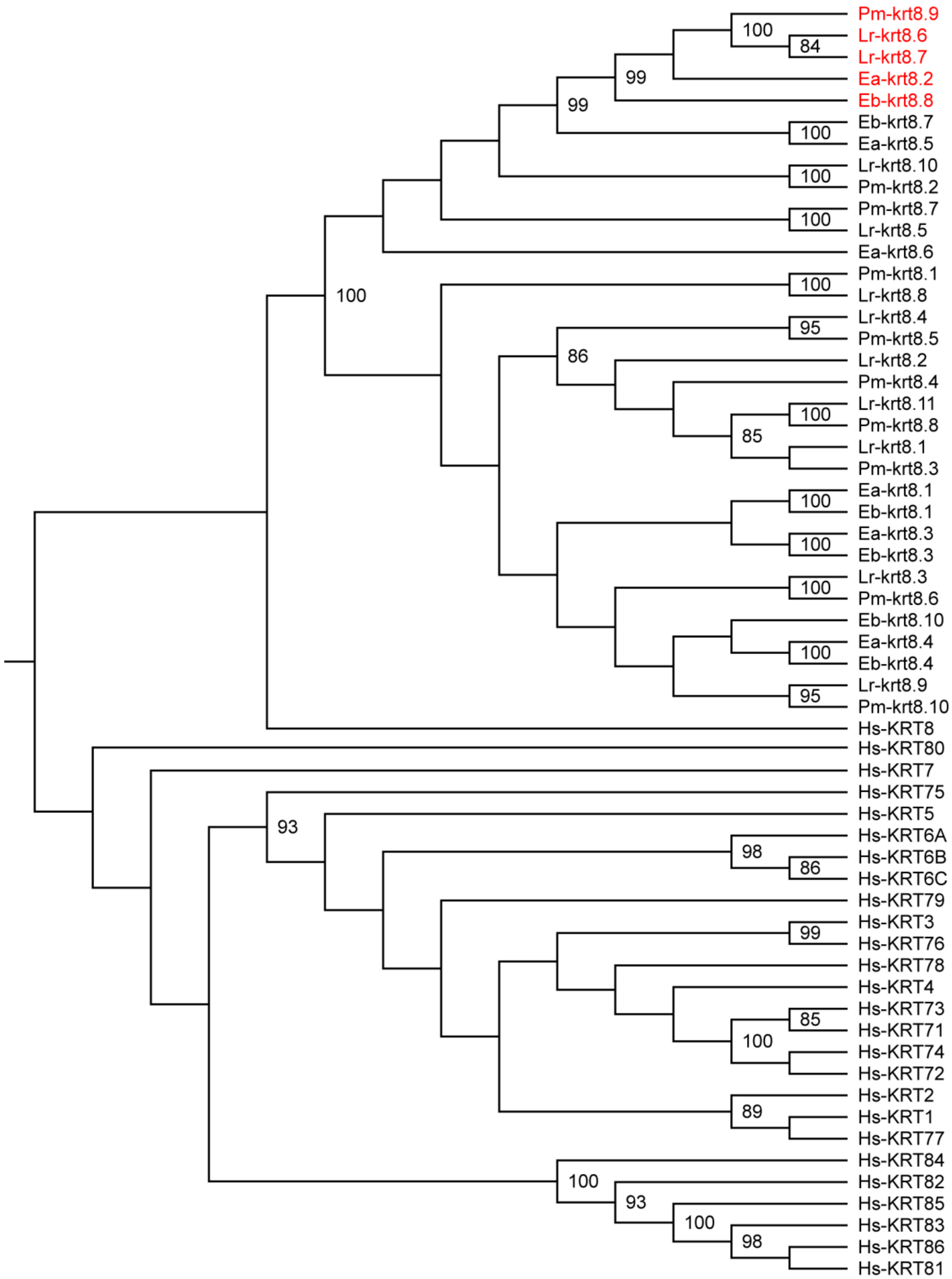

Figure S8. Phylogenetic analysis of type II keratins of four species of cyclostomes and humans. Bootstrap values ≥80 are shown. Species abbreviations: Ea*, Eptatretus atami*; Eb, *Eptatretus burgeri*; Hs, *Homo sapiens*; Lr, *Lethenteron reissneri*; Pm, *Petromyzon marinus*. Note that cyclostome type II keratins form a monophyletic clade.

```
>Hagfish_krt18.14
EKETLQQLNDRLDDYLKRVRSLESYNSKIEMEIKHLITSY-GPEMI------
DWESQEKHLEELRVQLAELTLDNANLSMQAETNYLSIKDFQIKLEIEQNLCHNIEMDINEMRKEIDSINLNILELEGNNESLVASLVMMKKQHK
ERVKELKANIAVEDLSNVQVDS-IQGMDLLAVLTSIREEYNRLSKKNEQDAEVWYREKI-
QNIKEVVGQGEAELNPLNEQLHSLRQEHHNLQLTINSSGTKKEFLRKSLTDVEIHFNSERNRMKGYLGQYESTLAGLKEAGK------------
---------TARCL----
>Hagfish_krt18.15
EKETLQQLNDRLDDYLKRVRSLESYNSKIEMEIKHLITSY-GPEMI------
DWESQEKHLEELRVQLAELTLDNANLSMQAETNYLSIKDFQIKLEIEQNLCHNIEMDINEMRKEIDSINLNILELEGNNESLVASLVMMKKQHK
ERVKELKANIAVEDLSNVQVDS-IQGMDLLAVLTSIREEYNRLSKKNEQDAEVWYREKI-
QNIKEVVGQGEAELNPLNEQLHSLRQEHHNLQLTINSS------------------------------------------------------
GTKVSESQRITTSQSIRSS-
>Frog_krt12.7
QKETLEQLNSRLAAYLEKVSQLEESNRHLEQKIQDLCERR-ARTSR------
DTSRYEKVIAEMEAQINRVKMENSELSLAVENTKLTANNFKAKYETELSLYQIITADIKEVKAADSHLEMETNCLKVELQIQTEELSSLKKDHQ
EDMAKLREDQSKCQV-KVEVDS-QPANDLTKTLEEMREHCKAIICNSNRKAE-PLVSKI-RHYTHQ--
NTIKPLQAEMVEVTLLRRTIQCLEAELETLHNMKASQRTTLIETEQNYATQLQRIQRIISRTEDELVKTRTDFKQMSADGGMLHYLKDLLQMEI
KTYGQLMDVE
>Hagfish_krt18.13
EQTQLKDLNERLGVYLERVSYLEKINGEMEASIKVELEQR-AANVP------
DWTRYEQIIKDLRQKINDTINENTSLSLEIDNNRFAGSDFRQKWETENDLYQAVEGDIASLRSQTNDINLSCSELETHLECLRSDLESMRRQHK
EEKEKLSKGLS-DGM-SVEVDA-VKGINLNEILNGVRNKYEEIVKKNQQEAEEEYQSKC-
KMAVPQMMKSCEALEASRSEVTELRSLLQTLSIEYQSLNSMVFSLEEMLQETEDRKANELSRLQSSLLKAEANLEGAQKNLRGQMQEFDILLDA
KMDLEREIAMYHKLINGE
>Lamprey_krt18.2
EKEELQGLNNRLSGYLQRVRTLEQDNNKIELEIAELMSLR-KGGPH------
DWAKWDGIFRALREEIFMLTMDTTKLELGIQNTDLARSDYYEKWETEHAIRMAVEADIQALRKMIDDTNVARLNLESRVEALKEELSVLRYTHA
EEVKKLLVEISSFEVGSVEVDSGTKDIDLNKALAEIRRQYEDLCRQNAVQVE-SYLETI-
TITNTKVTEKMQALEKYRVELNELRRTTQVLFLDLENMRSKKLSLENANADMSARYEMEVSSLRDMVIMLDKILVDVNMDLERQRSEYEMLTNA
KMRLEQEIAYYSSLLDQE
>Lamprey_krt18.8
EKEALQDLNERLAGYLDKVRYLEQANQELECKIKEFRANS-CIAEQ------
QWEPCMGNIEELMGQVEVATLENTRLVLEIDNARLASEDFRVKWEAESALRETVEADAHELRRLSAEYCACRNQLACEQNVLQGEIHTLKSHHR
GEMESLRMDYSSSTT-KVEMDN-SPGADTAGIISEIRAQYETMIHNNRHEAE-CLQSKL-
EAAEASAVHSHGELAAAKNQAHHLRQQYQTMEVEMESLRSANATLEDNLAETEERFSVEVRSLAEVLSRLEAEYSDVRANVERQLHEYESLLNI
KMGLEMEISTYKCLIEGE
>Hagfish_krt18.8
EKGAMQDLNDRLAGYLGKVRYLEGANRELECKIKD------------------------
GIESLMYQIEEASVENSQLVLAIDNARLTAEDFRKKWEIEVSLREELETDIQDLRNLANEYCECHNTVACEVNVIQDEIYYLKQSHR-
EIEGLRCEYANSTL-NVDIDH-SPNVDLSSIINDIRTQYETMIGNTHYEVECLMNNKA-----------------
TKNEANNMRYEFQSLEVEAESLHT-
NCTLEENLRETEERLNLELSNLAEIISSLEAEYTDVRANIERQIREYESLLNVKMQLEMEIGTYQKLIEGE
>Hagfish_krt18.9
EKQTMQDLNDRLSGYLDKVRYLEATNLELECKIKEFRANK-CIAER------
EWDPCLSGIESLMYQIEEASVENSQLVLAIDNARLTAEDFRTKWEIEVSLREDLEIDIQDLRNLANEYCECRNTVACEINVLEDEICHLKNSHR
E------VWYANSTL-NVDIDH-TPGVDLAGIINDIRTQYETMIASTHYELD---------
AAQASAHQSACNLEAVKTEANNLRYEFQALEVEAESLRS-
NCILEENLHETEARLNLEISNLAQIISILEADYADVRSNIERQIREYESLLNVKMQLEMEIGSQFEIFDPT
>Lamprey_krt18.6
EKAEMQGLNGRLATYIERVRSLDEANRRTELQIKELVEKR-PSGAV------
ELRQYHEAARELREQILKATMTNARLHVELDNGRLAAEDFRAKLESEVSIHTSVESDITNLRRAIDETNVTRMSLEGQVEQLEEQILHMQKSHA
DEKSSLLKEIEESSI-SVEVDS-VKGHNLNDIIAEIRTQYEALIKSNLQEME-VYKSKV-
DAIHPRISQNSEELGTLRQQLSEQRRAMQALYAEAETLRSTVSSLNEAAQDVEAHSAEGLAGLAGSISRLLSELGSARGDIDRQLREHETLLNT
KMRLEEEIETYRRLLEGQ
>Lamprey_krt18.1
EKAEMRGLNERLAEYIAKVHYLEGVNHELEVKIKELLKSG-HGKIQ------
GSSSRFTAAEELCAKIKSQIVDNARAGVKIENARLAADDFHYKLETERSARSCVEEDIARLYALLEEYGVAGAGAEAEIEALSEELHYIRKTHK
QDTATLGARLEASSV-SVEVAS-TKGSDLSEILAGLRRQYEAMIVKTHEEME-FYKQKM-
DTVNVTVSEKKQASQLVKDEISDMNHSTQSLQKELDTLWGLIRCLEEQLRSAEALNADSLTGYSSAIGALESELEKLRADTHRQMHEYSLLLNE
KMKLEQEISTYRTLLESG
>Lamprey_krt18.7
EKLEMQDLNTRLEEYMEKVRYLESVNKALEIKIKE-SRAS-NISTT------
NYDPLLVNIEALIEQITAAKLVNAQISLEIDNARLAADDFRTKWETEIVLRQSVEGDIDNLRGLKLDYESESGLLHSQLQLLAEELLYLKKNHA
EEIAALRAQCG-AEM-SVEVDS-TPGVDLSKIIAQIRAQYEEMIRKNQEEAE-AFKKQA-
EMVKATASQGQAALNTVKMESKEARQSLQTLMLELEMLRSTNKSLEDALADTENRYGHELQLLQAQLQKLEAEIAQVRTGDNAQLQEYQTLLNA
KMKLEMEIATYRRLLEGE
>Hagfish_krt18.7
EKEAMQDLNDRLSNYMDKVRRLEVTNGELEMKIDEMLKRH-GPEIS------
NNNVYFSTIEELKSKILAQVMENASLTLEIDNARLAADDFHTKWQTEVTLHSSVEGDIGSLGMLLDEYTLSRAGLETDVEFLQDELAYMRKNHE
DEVTALRAQITSSGM-SVEVDA-
TPGIDLARALKDMRNQYEAVVAQNNADAEATFQKQVTEKVKIVVEQRSQSSDAAKADVLESRRSMQTLQVELDTLRGQVNSLEFNLTETEGRKA
QELGSYEIVIQRLQQKLQSMKNDLNGKLGEYSELLNQKMLLEAEIATYRLLLDGN
>Hagfish_krt18.4
EKQAMQNLNERLHIYLQKVQSLENANAQLEIQIRELQKGK-APTSS------
DYESYYAIIVDLRAKIITQIMSNASISLEIDNARLAADDFKTKWDNEFALRTSVEADIDNLHGLHEEYMMASSSMQGELELLQDELNFMKKNHA
EEVAALKAQIAGSNM-SIEVDA-KSGPDIQQMLEDMRRKYEVIMEQNRVEAEQAFQQQV-
EQVQQVVVKQNQAAVSAKNEVTEVRQSMQSLQIELGTLQGQIASLENTLDDTKYRKQQELESYLIMLERVERDFENARMEINTKKHDYAKLLDE
KMKLETEIETYRRLLNGG
```

```
>Hagfish_krt18.12
EKQAMQGLNDRLARYLEKVQSLERSNAEIELKIKELLQGR-GPENK------
DYNHYFEIITDLKNTIVQQIMENAKIGLEIDNARLAADDFKTKWENENTLRNSVEADIANLHSLHDEYTLARSDLECDIEGLQEELNFMRKNHE
EEVAALKAQIAGSNM-SIEVDA-KSGPDIQQMLEDMRRKYEVIMQQNRAEAEQAFQQQA-
EQVKAVVVKQDQACSTAKNEVVETRRSMQSIQTEMETLRGLCNSLEDQLADINDRKARELEQYLNAVACLENDIATSRSNINEQLCKYSALLDE
KMKLEKEIETYRTLLSGG
>Lamprey_krt18.5
EREEMQELNKRLAGYLHKVKELEAANATTEANIQELLRVR-GAIVQ------
DHGARFAAIADLRSKMLAQVLENARIGLDVDNARLAADDFRCKWETEVALRSSVEADIGNLHMLLDEYTGSRDAMASEAQSMHEELAYMKRNHR
ERLALSKAQVEGSSV-SIQVDS-AKGVDLTKMLGDMREQYESLIARSRGQAE-EFRKQL-
ESVKVQSFQQDQAASAAKAELAEVRRTMQGLTVELESLMALLGSLEEQLRQTEHDNARELSSHGDQIGALQGRLHSVHGATNAQLRDYSELLNM
KMKLEQEIGTYRRLLEGE
>Hagfish_krt18.2
EKQEMQSLNDRLAEYLDNVRFLEKTNEDLEFKIKDILKLK-GVTLK------
DCTAYYSNIEELRKKILTRAQDNAKITLEMDNAKLAAEDFKSKWQMELNLRTSVETDIANLRSFLDEYNMERLGMEGEIEALQEELTFMKKNHQ
EEVAALKAQIEKSSM-SVEVDS-IPGMDLSKTISEIRAQYENMIAKNREDAENTFRKQV-
DSHQAVPMQDDLTTQSKKLELQEMRRSMQGLQMELETLHNVVASLKDNLADTENHNVRELSGYANIVTNLESELASMHLDINRQLKDYSDLLNV
KMRLEQEINTYRRLLEGE
>Hagfish_krt18.1
EKQEMQTLNDRLAEYLDKVHFLEKANRELEIKIKDIIKSK-GVTLK------
DYSTYYSIIEDLRNKILLQILENARVSLEIDNARLAAEDFRNKWQMELNLRTSVENDIGGLRSMLDEYNMARLGLEGEIEAMREELVFMKKNHE
QEVGALKAQIEDSTM-SIEVDS-TPGPDLSKTLSEIRAQYENMIAKNRDDAEVTFRKQV-
ETHQTIIVQQDQATQSIKVELQETRRNLQGLNVELESLKSMIRSLEDTLADTEDRNARDLAFYQNSISRLEAEYASMRCDINRQLKDYSDLLNM
KMKLEKEIATYRSLLEGE
>Lamprey_krt18.3
EKVEMQGLNERLAEYLDKVKYLESANQAIELKIKEMLQGK-GSTAK------
DYSVYYSTIEDLREKIFVQILENAKISLEIDNARLAADDFRSKWETEAALRMSVETDIGNLRGLLDEYGMACMGLEGDIEALREELIFMKKNHE
NELAALRAQLSGANM-SVEVDS-TRGQDLHKILDDMRAQYEGLIAVNRANAE-MFNKQA-
ESVVVQGAQQSQAALAAQSEMSETRHAMQSLMTELESLRGLIRSLEDQQYDTEDRNARDLASYTVHIQMLEGELGSVRVGINQQLKDYAELLNM
KMKLEQEISTYRRLLEGE
>Lamprey_krt18.4
EKQELQGLNDRLAGYIEKVRILEQANKEIEIKIKELLKGK-GPTIK------
DYSMYYATIEDLRAKILAQTLENARVSLEIDNARLAADDFRSKWETELALRNSVEVDIANLKCLLDEYNMARMGLEGEIEALREELIFMKKNHE
QEVQALRAQIADTSM-SVEVDN-VKGADLARILADIRAQYEAMIARSRDEAE-EFRKQL-
ETVKQVSVQQNQVTITAKTELQETRRSMQGLQVELDSMRSMIRSLEDTLADTEDRNARDLSGYQNILARLEAELTSLRCDINRQLKDYADLLNM
KMKLEAEIATYRRLLDGG
>Lamprey_krt18.9
EKLEMQGLNDRLAGYIEKVRFLEGANQELELKIKELLKGK-GGSSK------
DYSAYYPIIEDLRAKILAQILENARISLEIDNARLAADDFRSKWETELALRSSVEADINNLRGLLDEYNMARMGLEGEIESLREELIFLKKNHE
EELAALRAQLEGSSM-SVEVDS-AKGKDLSKILAEIRAQYEAMIARNRVDQE-EFKKQV-
DTVKVASVQQNQAAQSAKNEVVETRRSMQSLQAELDSLRGLIRSLEQQLQDTEERNARELSGYTNVIQRLEGELNNMRGDINRQLKDYSDLLNM
KMKLEEEIATYRRLLEGE
>Lamprey_krt18.10
EKLEMQGLNDRLAGYIEKVKFLEGANRELELKIKEMLKGK-GGSSK------
DYSGYYKIMDDLRAKILVQILENARISLEIDNARLAADDFRTKWETELALRSSVEADINNLRGLLDEYNMARMGLEGEIESLREELHFMRKNHE
EELAALRAQLEGSSM-SVEVDS-AKGVDLSKILAEIRAQYEAMIARNRVDQE-EFKKQV-
ETVKVSSVQQSQAANSAKSEVVETRRAMQSLQAELDSLRGLVRSLDDQLQDTEDRNARELSSYTIIIQRLEGELNNMRGDINRQLKDYSDLLNM
KMKLEAEIATYRRLLEGE
>Human_KRT23
GKATMQNLNDRLASYLEKVRALEEANMKLESRILKWHQQR-DPGSK-----
KDYSQYEENITHLQEQIVDGKMTNAQIILLIDNARMAVDDFNLKYENEHSFKKDLEIEVEGLRRTLDNLTIVTTDLEQEVEGMRKELILMKKHH
EQEMEKHHVPSD-FNV-NVKVDT-GPREDLIKVLEDMRQEYELIIKKKHRDLD-TYKEQS-AAMSQE-
AASPATVQSRQGDIHELKRTFQALEIDLQTQYSTKSALENMLSETQSRYSCKLQDMQEIISHYEEELTQLRHELERQNNEYQVLLGIKTHLEKE
ITTYRRLLEGE
>Lizard_KRT23
EKQTMQDLNDRLAAYLDKVHSLEEANTQLEGCIREWHERR-SQGMK-----
HDFTDYEQNIVDMHERIEAGRVTNAGIVLQIDNAKMATEDFRLKYESEKALRQSVQDDVENIRKELDNMTIIITDLEMEIEALREDHILKKKEH
EKDMEASGAFKD-VNV-NVKVNT-APQEDLAKILAGIRADYEAIIQKNHQSLD-AFQKQI-
ANDSSEEKPNQEEQQSNQKEISELNRTLQSLEIDLQTHLNKKCSLENNLDETRAYYAFQLQSIQNLISQCEEELSEFRHDIKCQNNQYKVLLGI
KTRLETEISTYRQLLDGK
>Alligator_KRT23
EKQMMQNLNDRLAAYLDKVRSLEAANAQLESCIVEWHKKR-SHGKR-----
YDFNQFEQSIADMQGQIQDGKISNASVLLQIDNAKLASEEFRLKYEAEKYQREGVQFDVENLRKELDNLTIVTTDLEMEIEGLREEHILRKKDH
EADMHEQRSTKD-VNV-NVKVKA-TPPEDLTKILAGIREDYEAIIEKNRRGLD-VYKQQT-
SAASLAVTNSPEQLQNNDKEIKNLKRNFQALEIELQAQLSKKHALENTLSETRAHYASRLQNIQEFISKCEEELSELRHDVRCQSNKYKVLLGI
KTRLEREISTYQLLLEGR
>Frog_krt12.1
EKETMRNLNNRLATYLEKVRSLEKANAELEGQIKEWYQQNSGFGKR------
DDAHYFQVIEELTKQIVNAKMENGKLLLQMDNATLALDDFKMKLEYELNLGAAVEKDLSELRKVVDDLTMTRTDLESQIESLKEEMVYLKKNHT
QDMVGLHKQE--GTI-DVEVDA-APSVDLGKTMEDMRSQYEQLVEKQRLEAK-YFDRKV-
EEWNEEVQTNTAELEKCRKDLSELKHKVQDLEIESQAELSKRKAAGNALDNVNSRYTMELADKQEKIARFEEHLQQTRRDVGFQIQEFTILFDL
KNRLEAEISTYRRLLDAE
>Frog_krt23
GKETMQNLNDRLASYLEKVRSLEEANTGLESKIQLWHRNR-AQNHK-----
RDYSVYEKTIGELQSQLVDGHMNGAKIKLQMENAKLAFDAFKRKYETEKTIRTALEGDLESLRRAMDNLTIVRTDLEMEVEGMRKELIYMRMGH
EEDIKLAQFQKKGASV-DVKIDA-PPSVDLAKTIAEIRKEYETLIEKNRREAS-EYSHQS-
SSVQQEVNSNTEALQNSRNQIKDLKRTLQTLEIELQGEIRRKHGLEGTLAETEANAAGQLQKIQATICRMEAELSNVRGELERQNIEYRALLDV
KTRLENEIATYRELLEGD
```

```
>Lizard_KRT117
QKGTMQNLNDRLAAYLGRVHSLEESNAQLEQNIREYYAKRASAGGP------
DLSGYFNTLSELKAKIEQETLNNAGLLLRIDNAKLAADDFKVKLESEVAIRLSLEGDVNGLRKVLDDVNASRASLQVQVDNLQEELVYLKRNHE
EEVASLQGRLG-GTV-NVEVDS-MPGVDLQKVLAEIRDQYEDVMEKNRQEAE-AHKAQC-
DAINQQVADSSEALQAAQLKITELRRLAQALEIELQSLRSTKIALEGTLVETESRYGMELSRLRELISARESELFQLRSEAQRQAEDYKRLMDI
KNKLEQEIATYRRLLEGL
>Alligator_KRT117
EKDTMQNLNDRLAAYLERVCSLEEANGQLEQRIQELQVKKTLARHY------
DPSSCFSTIAELRSQIQDESVRNSQLTLQLDNAKLAANDFRMKYEAELATRLGVESDIKGLRKVLDGLTEARASLQVQIESLKEELTYLQQNHG
EEVAALQGHLG-GSV-SVEVDA-SPGINLVKTLAEIRDQYEDIIEKNQREAE-AHKQQC-
ETFTQEVATSSEALQAARTKATELKRIVQSLEIEFQMLQSTKEALEGTLAETESHYGVEMAQLQNLVAGKEAELAQLRTDTQRQANEYQHLLDL
KTRLEVEIATYQHLLEGQ
>Frog_krt18
EKETMQDLNDRLASYLERVRSLEAANKKLEVQIRQHTEKK-GPS-K------
DWSPYYKTIEDLRKQVFDSTLENSQLVLQIDNARLAADDFRVKYEAEMAIRMSVEGDITGLRKLIDDTNVSRMNLENEIESLKEELIFLKKNHQ
DDVTELQAQVARSAV-TVEVDA-PKSQDLGKIMTELRAQYDGLAQKNRDDVE-KYQSKV-
DEHTVQVNLDTEALHSAKSSVTDLRRTVQSLEIELESLRNQKASLEGTLHDTEARYAMELEMLGGTAMAMEAELVQVRSDCQRQQQEYQALLNT
KMKLEAEIHTYRRLLEGD
>Human_KRT18
EKETMQSLNDRLASYLDRVRSLETENRRLESKIREHLEKK-GPQVR------
DWSHYFKIIEDLRAQIFANTVDNARIVLQIDNARLAADDFRVKYETELAMRQSVENDIHGLRKVIDDTNITRLQLETEIEALKEELLFMKKNHE
EEVKGLQAQIASSGL-TVEVDA-PKSQDLAKIMADIRAQYDELARKNREELD-KWSQQI-
EESTTVVTTQSAEVGAAETTLTELRRTVQSLEIDLDSMRNLKASLENSLREVEARYALQMEQLNGILLHLESELAQTRAEGQRQAQEYEALLNI
KVKLEAEIATYRRLLEDG
>Alligator_KRT18
EKETMQDLNDRLASYLEKVRSLETANHNLEVQIREFLDKK-GPSTQ------
DWTHYWDIIEDLRNKIYEESVDNARIVLQIDNARLAADDFRVKYEGELAIRQSVEADIAGLRKVIDDTNMSRLQLESEIENLKEELIYMKKNHE
EEAKSLQAQVSGSGL-TVEVDS-PKTHDLGKIMTDIRAQYEALAQKNREDLD-KWNQQI-
TESTIEITQSTKDMDTAHGTLTELRRTYQTLEIELESLRNLKGSLEANLMEVENRYGMQLEHLNGLLLRTEAELMQVRTEVQRQAEEYQALLNI
KVKLEAEIATYRRLLEGG
>Lizard_KRT18
EKETMQDLNDRLASYLEKVRSLEADNRKLEIQIREHLDKK-GPSAR------
DWSHYFELIEDLRNQIFDQTIDNSRIVLQIDNARLAADDFRVKFEAELAIRQSVENDINGLRRVIDDTNMMRLQLESEIEALKEELVFMKKNHQ
DEVNNLQAQIANSGL-TVEVDA-PKTQDLGKIMAEIRAQYDVLAQKNLEDLD-KWSQQI-
TESTVVITEHSKDIEKARANITDLRRSVQTHEIELESARNLRASLEANLHEVEMRYGLQMEHLNGIILRTEADLAQLRNDVQRQAEEYQILLNI
KDKLEAEITTYRHLLEGG
>Frog_krt20
EKQTMQNLNDRLATYLEKVRSLEKSNSKIELQIKEWHSKNAGVGEK------
DYQGYYKTIEALQNEILNSTMVNAEILLKIDNAKLAAEDFRLKFESERSLKIGVEKDIIGLRKLIDDLNLQRIDLEHQYESLQENLAYLRKNHE
EEASVLRNQIT-GHI-NVEVDA-APSVDLAKVLADMRAQCEAVVSKIKQDAK-QFETQI-
EEVNIQIGGNVTELEKHRSSVKEVKRHVQSLEIELQSEISKKDALNATLNNINAQYAAQLLHMQTAIKCIEEQLIQIRSDMSRQSQEYEILLNI
KLRLELEIETYRRLLEGE
>Lizard_HAS3
EKVLMQNLNDRLGNYIDKVRCLEKENAALESQISDFCLKH-GLTGE----
MKDHSHFYQQIEELKKQVFVANLENNKILLKIDNNHMDLEDLRKKYETEYVIRENVEADVNGLRPVLKELTGYKADLKAELNSLKEEICDLKKT
HEKDLNCRKKRSH-GS--NKEVSI-GPGTDLKQMLKEMRQKYEAKIESNRKEVV-QYEDKL-
KEMNLNVNNSSQDIEDGNHKVLDLKCKLPALEIDLQAQYNMRDTLKVCLAEIECHFQTQLGEMQEQISNMEHQLAELHLEMETQDQEYKGLVDV
KKRLEQEIQTYSSLLKEG
>Lizard_KRT9L2
EKSTMQNLNDRFASYLNKVKSLEAENNQLETLIREWYQKH-GQTSE----
PKDYSSYYTEIDQLVNELISASLESNKILLDVDNARMAAEDFKLKYETEYGLRQNVDADLQGLRPLLDKLTMDKSDLEMQYESLHEELVHLKKN
HDEVMKSQHHQSG-GDV-NVEVNS-SPGQDLTKTLNDLRQEYEEIIMKNRKEVQ-QFEAKM-
EEARQQQSPESQEGGSGRHQITELTREYQTLEIELQTQLSTIQALQNNLNNTEGGYNMQLQQLASQIEPVEAELAGIKGEIQNQTQEYQTLLGI
KTYLEQEINQYRQLLEEG
>Lizard_HAS1
KKMTMQNLNDRLASYLDNVRYLEEENAQLECKIRDWYDQQ-GSPND----
PKDYNYYYEQTEDLKKQLICATVDCRKLILDIHNNKLTADDFRMRYEAERVFRQDKETDIDSLRQVLDQLTLGRSDLEAQLESFQEELCCLKKN
HEEKVKLLTKQAQ-GNL-NVEVNS-NPSSELQKALDNLRRRYEAIIEKNVKEIE-TYESMT------
EAINMSKELKDDNNQVVDLKGQLQTLESNLQTQLSLRDTLQDSLAETQCLYNNSLAEIQNQICYMEQQLVELRVKTECLDQEYIRLSDAKCLLE
HEIETYCCLLGEE
>Lizard_HAS4
GKMTMQNLNDRLASYLDKVRCLEEENADLESRIREFYAKQ-GPLSE----
PKDYSHYHQQIEDLKNQLICTSVENNKLLLCIDNSKLTADDFRTKFETECCLRQNVEADINGLHQILDQLTACRADLDVQCDNLQDELNCLKKN
HAEEVACLKKQST-GDV-NVEVNT-CPGPDLKKILEEMRCKYETMIEGNRKEVE-AYESKI-
EEVNRDVCNSSQEIEESNNKVTELRRQLQALQIDYEAQCSLRDTLEASLGETELRYNGHLGDLQERISCLEQQLAELRSEMECQNHEYTELLDV
KSRLEQEIGTYRGLLEGG
>Lizard_HAS2
EKMTMQNLNDRLASYLDKVRCLEEENGQLECKIKEWYDRQ-GPVNE----
SKDYAAYYQQIEDLKNQIVCATVENSKLVLDIDNNRMTADDFRMKYETELALCQNVEADINGLRQVLDQLTLCRSDLEAQLESLREELCCLKKN
HEEESKHLKKQSS-GDV-SVEVNT-CPGPDLKKVLDELRCQYENMIENNRREVE-KYESKM-
EEVNLEVCNSSKEAEDCNNQIVDLKRQLQTLEIDLQAQLSLRDTLQDSLAETECRYNGHLAEIQNQISCVEQQLGELRAEMECQNREYRELLDV
KCRLEQEIQTYRCLLEGG
>Alligator_HAS1
EKITMQNLNDRLASYLDKVRCLEEENAELECRIREWYAKH-GPTCE----
PKDYSCYLREIEDLQNQIVCATIENNKIVLNIDNSRMAADDFRVKYETELSLLQSVEADINGLRQVLDQLTLCRSDLEAQLEALKEELCCLKKN
HEEEMNCLRKQST-GDV-SVEVNT-CPGPDLKQVLDEMRCQYEAIIAQNRKEVE-DYACKI-
EEVNREVISSGQEIECSNSQVCELRRQLQALEIDLQAQIRMRDNLQSSLAETECRYNNQLAELQNQIICVEQQLAELRAEIECQNQEYKVLLDV
KCRLEQEIHTYCCLLEGG
```

```
>Alligator_HAS2
EKITMQNLNDRLASYLDKVRCLEEENAELECRIRDWYAKH-GPTCE----
PKDYSCYIREIEDLQNQIVCATVDNNKIILDIDNSRMTGDDFRMKYETELALCQSVEADINGLRQVLDQLTLCRSDLEAQLEALKEELCCLKKN
HEEEINCLRNQST-GDV-NVEVNS-CPGPDLKQILDDMRCQYEAIIAQNRKEVE-DYACKI-
EEVNQEVISSSKEIECSNSQVSELRRELQALEIDLDSQLRMRDNLQSSLAETERRYNNQLAELQNQIICVEQQLAELRAEIECQNQEYKVLLDV
KCRLEQEIQTYCCLLEGG
>Lizard_KRT9L1
EKSTMQNLNDRLAAYMNKVKYLEDENAQLEVLIREWYQKH-GDVGT----
VRDYTHHYQEIDQLFNELVNETDDYNKILLNIDNTRMTAEDFQIKYQTEAGLRQNVEADINGLRPMLDQLTLARSDLEAQFESAKEELITLKKN
HQEALRELQSQRGHGDI-NVEVNA-APGPDVKQRLDQLRAEYEAIIDNNRREVE-QFESKM-
EEVRQQVSSSGLEISSGNQQVTDLRRQYQTLEIELQSALSMIQSLQSNLEDTERRYNLQLQQIQSMIQPVEGELASLRGEIESQSQEYQSLLGI
KTRLEQEIAQYRQLLDEG
>Alligator_KRT9L1
EKTTMQNLNDRLATYLDKVRRLEEENADLEERIREWYRKQ-GPSIS-----
KDYSPYYQTIEQLQNQIISATVDNNKLILDIDNSKMIADDFRMKYENELAIRQGVEADINGLRNFLDELVRVRSTLELEVETLKDELLTLKKNH
EEEMRLLQSQTG-GDV-NVEVNA-APGQDLTKILNDMRQEYEEIIAKNRREVE-QYEVKM-
EEVNQQVTSSGQEVQTSSHQLTELRRVFQSLEIELQTQLSMKASLENNLAETEARYGAQLQQIQAMINCVEEELAQLRCEMESQNQEYKMLLGI
KTRLEQEIAQYHGLLQEG
>Alligator_KRT9L5
EKMTMQNLNERLASYLDKVRCLEEDNLQLERLIREWYQTH-GPQGG-----
KDYSHYYGPIEDLLNQIVLATVANNKIILDLDNARMTAEDFRMKYETEYSLRQSVEGDINGLRPVLDNLTLAKSDLEMQFESLREELIQLKKNH
QEEMSSLQSESG-GDV-SVEVNA-APGEDLMGKLNEMRKEYEYIIQRNREEVE-RYESKM-
EEVRHEVHTSGQEIESGNQQVSELRREYQRLEIELQSQLSTLQSLQSNLEDTDYRYNMQLQQIQNMIIPVEEELASIRCEMESQNEEYKMLLGI
KTRLEQEICQYRELLDEG
>Alligator_KRT9L2
EKMTMQNLNERLASYLDKVRHLEEENANLERLIREWYQNH-GPQCG-----
RDYSQYYGPIEDLQNQIISATVDLNKVLLDIDNTRMTADDFRIKYETEYSLRQNVEGDINGLRPILDQLTLARSDLEAQYESLKEELISLKKNH
EEEMRGLQSQSG-GDV-NVEVNT-TPDSNLMQKLNEMRCEYEQIIENNRKEVE-RYETKM-
EEVNQEVHTSGQEIQSSNHQVSELRRDYQTLEIELQSQLSMVQSLQSNLEDTERRYNIQLQQIQNTITTVEEELANIRCEIESQNYEYKMLLDI
KCHLEQEIAQYRALLEEG
>Alligator_KRT9L4
EKMTMQNLNERLASYLDKVRRLEEENANLEQLIREWYQKQ-GPQGG-----
RDYSQYYKTIEDLQNQIISATVDLNKVLLDLDNTRMTVDDFRLKYETEYGLHQNVESDINGLRPMLDQLTLARSDLEAQYESLKEELISLKKNH
EEEMKGLQSQSS-GDV-SVEVNA-APGTNLTEKLNEMRGEYERLIENNRREVE-SFEAKM-
EEVNQQVHSSSQEMESSNQQVSELRREYQTLEIELQSLLSMLQSLQSNLEDTERRYNMQLQQIQAMIIPVEEELANIRCEMESQNEEYKMLLGI
KTRLEQEIAQYRALLEEG
>Human_KRT9
EKSTMQELNSRLASYLDKVQALEEANNDLENKIQDWYDKK-GPAAI----
QKNYSPYYNTIDDLKDQIVDLTVGNNKTLLDIDNTRMTLDDFRIKFEMEQNLRQGVDADINGLRQVLDNLTMEKSDLEMQYETLQEELMALKKN
HKEEMSQLTGQNS-GDV-NVEINV-APGKDLTKTLNDMRQEYEQLIAKNRKDIE-NYETQI-
TQIEHEVSSSGQEVQSSAKEVTQLRHGVQELEIELQSQLSKKAALEKSLEDTKNRYCGQLQMIQEQISNLEAQITDVRQEIECQNQEYSLLLSI
KMRLEKEIETYHNLLEGG
>Alligator_KRT36L1
GKETMQLLNDRLASYLEKVKSLEKENTDLERKIREWQECQTPLVCT------
DFSSCYSTTEELQQQICSARAENARLRLDIDIVKVASDDFSFKYEYECGLCQSAEADISSLHRFLDDLTLCKTDLETELESLTEELLCLRKNHE
EEASPLRHQLG-DCI-NVELDT-APSCDLNKVLEEIRCQYETLIEKNQCEVE-DYHTQM-
EELNKEVISDEEQLQGCQNEIIQLKHTVQALEIDLQTQHSLNTALESTLTDTEARYSHQLSELQCLISNMEAQLAELRSNIQHQNSEYKVLLDV
KSQLDCEISAYRRLLDEE
>Alligator_KRT36L2
EKETMQFLNDRLACYLEKVRFLEQENADLEYKIREWYECQTPYVYT------
DFNCYYKMIEELQQQICCAKADNARLCLDVDNLKVASDDYATKYEYEHGLCQSAEADVKSLRRILDDLTLCKADLETELESLTEELLCLRKNHE
EEASPLRRQLG-DRI-NVELDA-APSCDLNKVLEEIRYQYETLVDKNQCEVE-DYHTQM-
EELNKEVISSGEQLQGYQNEIIQLKHTVQALEIDLQTQHNLNTALENTLAETETHYSQQLSELQCLVSDVEVQLAELRSDIEHQDGEYKVLLDA
KCQLDCEISVYHSLLDVE
>Lizard_KRT36L1
EKETMEFLNNRLGNYLERVRCLEQENAELEAKIREWYEHENAYVCT------
DFKPFYCTIDELQQQICCTKADNAKLCLDIDNLKMASDDYCTKYDYELSLRQRDECDSSGLRKVLDNLTLCRADLEAQLESLTEEMMCLKKNHE
EESSALRSQLG-DRI-NVEVDA-APSCNLNKVLDEMRCQYEADLEKNRREVE-DYCAQM-
EELNKEVISSGEQLQCCQTEIIETRRCVQTLEIELQAQQSMKSALETTLQETESRYCTQLAQLQCLISNVEAQLTEIRSDIERQNCDYKALLDE
KARLDCEIAAYRNMLEGE
>Human_KRT37
EKETMKFLNDRLANYLEKVRQLEQENAELETTLLERSKCHESTVCP------
DYQSYFRTIEELQQKILCSKAENARLIVQIDNAKLAADDFRIKLESERSLHQLVEADKCGTQKLLDDATLAKADLEAQQESLKEEQLSLKSNHE
QEVKILRSQLG-EKF-RIELDI-EPTIDLNRVLGEMRAQYEAMVETNHQDVE-QFQAQS-
EGISLQAMSCSEELQCCQSEILELRCTVNALEVERQAQHTLKDCLQNSLCEAEDRYGTELAQMQSLISNLEEQLSEIRADLERQNQEYQVLLDV
KARLENEIATYRNLLESE
>Human_KRT38
EKETMQFLNDRLANYLEKVRQLEQENAELEATLLERSKCHESTVCP------
DYQSYFHTIEELQQKILCSKAENARLIVQIDNAKLAADDFRIKLESERSLRQLVEADKCGTQKLLDDATLAKADLEAQQESLKEEQLSLKSNHE
QEVKILRSQLG-EKL-RIELDI-EPTIDLNRVLGEMRAQYEAMLETNRQDVE-QFQAQS-
EGISLQDMSCSEELQCCQSEILELRCTVNALEVERQAQHTLKDCLQNSLCEAEDRFGTELAQMQSLISNVEEQLSEIRADLERQNQEYQVLLDV
KTRLENEIATYRNLLESE
>Human_KRT39
EKETMQILNERLANYLQKVRMLERENAELESKIQEESNKELPVLCP------
DYLSYYTTIEELQQKILCTKAENSRLVSQIDNTKLTADDLRAKYEAEVSLRQLVESDANGLKQILNVLTLGKADLEAQVQSLKEELLCLKNNHK
EEINSLQCQLG-ERL-DIEVTA-APSADLNQVLQEMRCQYEPIMETNRKDVE-QFNTQI-
EELNQQVVTSSQQQQCCQKEIIELRRSVNTLEVELQAQHRMRDSQECILTETEARYTALLTQIQSLIDNLEAQLAEIRCALERQNQEYEILLDV
KSRLECEITTYRSLLESS
```

```
>Human_KRT40
EKETMQFLNDRLASYLEKVRSLEETNAELESRIQEQCEQDIPMVCP------
DYQRYFNTIEDLQQKILCTKAENSRLAVQLDNCKLATDDFKSKYESELSLRQLLEADISSLHGILEELTLCKSDLEAHVESLKEDLLCLKKNHE
EEVNLLREQLG-DRL-SVELDT-APTLDLNRVLDEMRCQCETVLANNRREAE-ELAVQT-
EELNQQQLSSAEQLQGCQMEILELKRTASALEIELQAQQSLTESLECTVAETEAQYSSQLAQIQCLIDNLENQLAEIRCDLERQNQEYQVLLDV
KARLEGEINTYWGLLDSE
>Lizard_KRT36L2
EKELMQCLNDRLANYLERVRCLEQDNANLECKIREWHECQAPYEST------
DFHSHYKIIEELQHQILCAKTENARLVLEIDNARLAADDFRTKYETELALRQCVESDIHGLRKILDEMTLCRADLEAQLESHKEELLCLKKNHE
EEANALRSKLG-ARV-NVEVDA-APSCDLNKMLGEIRNQYECLAEKNRRDVE-TFSSKM-
EELNQQVLSSDEELQNCQSEIIELRHTMQALEIDLDAQQNMKNALECTLHETEARYSTQLAQLQCMISTIEGQLGDLRNDMERQSHEYKILLDV
KTRLEAEISTYRRLLEGE
>Alligator_KRT36L3
EKETMQFLNDRLACYLEKVRHLEQENADLECKIREWYECQVPYVCT------
DFQAFYKTLEELQQQILCAKTNNAKMVLQIDNARLAADDFRTKFESEQVIRQSVDADINGLRRIMDEFTLCRADLEMQLESLKEELLCLKKNHE
EEASVLRCQLG-ARV-NVEVDA-APSCDLNKVLNEIRCQYENLVENNRKDVE-AFSTKM-
EELNQQVISSGEQLQCCQTEITDLRRTIQALEIDLEAQTSMKSTLECTLHETENRYASQLAHLQCLITNVEAQLAELRCDIERQNQEYMVLLDV
KTRLETEIATYRRLLEGE
>Human_KRT32
EKETMQFLNDRLASYLTRVRQLEQENAELESRIQEASHSQVLTMTP------
DYQSHFRTIEELQQKILCTKAENARMVVNIDNAKLAADDFRAKYEAELAMRQLVEADINGLRRILDDLTLCKADLEAQVESLKEELMCLKKNHE
EEVGSLRCQLG-DRL-NIEVDA-APPVDLTRVLEEMRCQYEAMVEANRRDVE-EFNMQM-
EELNQQVATSSEQLQNYQSDIIDLRRTVNTLEIELQAQHSLRDSLENTLTESEARYSSQLAQMQCMITNVEAQLAEIRADLERQNQEYQVLLDV
RARLEGEINTYRSLLENE
>Human_KRT34
EKETMQFLNDRLASYLEKVRQLERDNAELEKLIQERSQQQEPLLCP------
SYQSYFKTIEELQQKILCAKAENARLVVNIDNAKLASDDFRSKYQTEQSLRLLVESDINSIRRILDELTLCKSDLESQVESLREELICLKKNHE
EEVNTLRSQLG-DRL-NVEVDT-APTVDLNQVLNETRSQYEALVEINRREVE-QFATQT-
EELNKQVVSSSEQLQSCQAEIIELRRTVNALEIELQAQHNLRDSLENTLTESEAHYSSQLSQVQSLITNVESQLAEIRCDLERQNQEYQVLLDV
RARLECEINTYRSLLESE
>Human_KRT33b
EKETMQFLNDRLASYLEKVRQLERDNAELENLIRERSQQQEPLLCP------
SYQSYFKTIEELQQKILCSKSENARLVVQIDNAKLAADDFRTKYQTEQSLRQLVESDINSLRRILDELTLCRSDLEAQMESLKEELLSLKQNHE
QEVNTLRCQLG-DRL-NVEVDA-APAVDLNQVLNETRNQYEALVETNRREVE-QFATQT-
EELNKQVVSSSEQLQSYQAEIIELRRTVNALEIELQAQHNLRYSLENTLTESEARYSSQLSQVQSLITNVESQLAEIRSDLERQNQEYQVLLDV
RARLECEINTYRSLLESE
>Human_KRT31
EKETMQFLNDRLASYLEKVRQLERDNAELENLIRERSQQQEPLLCP------
SYQSYFKTIEELQQKILCTKSENARLVVQIDNAKLAADDFRTKYQTELSLRQLVESDINGLRRILDELTLCKSDLEAQVESLKEELLCLKSNHE
QEVNTLRCQLG-DRL-NVEVDA-APTVDLNRVLNETRSQYEALVETNRREVE-QFTTQT-
EELNKQVVSSSEQLQSYQAEIIELRRTVNALEIELQAQHNLRDSLENTLTESEARYSSQLSQVQSLITNVESQLAEIRSDLERQNQEYQVLLDV
RARLECEINTYRSLLESE
>Human_KRT33a
EKETMQFLNDRLASYLEKVRQLERDNAELENLIRERSQQQEPLVCA------
SYQSYFKTIEELQQKILCSKSENARLVVQIDNAKLASDDFRTKYETELSLRQLVESDINGLRRILDELTLCRSDLEAQVESLKEELLCLKQNHE
QEVNTLRCQLG-DRL-NVEVDA-APTVDLNQVLNETRSQYEALVETNRREVE-QFATQT-
EELNKQVVSSSEQLQSYQAEIIELRRTVNALEIELQAQHNLRDSLENTLTESEARYSSQLSQVQRLITNVESQLAEIRSDLERQNQEYQVLLDV
RARLECEINTYRSLLESE
>Human_KRT35
EKETMQSLNDRLAGYLEKVRQLEQENASLESRIREWCEQQVPYMCP------
DYQSYFRTIEELQKKTLCSKAENARLVVEIDNAKLAADDFRTKYETEVSLRQLVESDINGLRRILDDLTLCKSDLEAQVESLKEELLCLKKNHE
EEVNSLRCQLG-DRL-NVEVDA-APPVDLNRVLEEMRCQYETLVENNRRDAE-DLDTQS-
EELNQQVVSSSEQLQSCQAEIIELRRTVNALEIELQAQHSMRDALESTLAETEARYSSQLAQMQCMITNVEAQLAEIRADLERQNQEYQVLLDV
RARLECEINTYRGLLESE
>Human_KRT36
EKETMQFLNDRLANYLEKVRQLERENAELESRIQEWYEFQIPYICP------
DYQSYFKTIEDFQQKILLTKSENARLVLQIDNAKLAADDFRTKYETELSLRQLVEADINGLRRILDELTLCKADLEAQVESLKEELMCLKKNHE
EEVSVLRCQLG-DRL-NVEVDA-APPVDLNKILEDMRCQYEALVENNRRDVE-AFNTQT-
EELNQQVVSSSEQLQCCQTEIIELRRTVNALEIELQAQHSMRNSLESTLAETEARYSSQLAQMQCLISNVEAQLSEIRCDLERQNQEYQVLLDV
KARLEGEIATYRHLLEGE
>Frog_krt56
KKETMQNLNDRLANYVDKVRVLEAANAELEWKINEWYDKQRSTSDG----
GKDYSKYATLIDDLKLKILSTTKDNRGITLQSDNARLAIDNFRLKYENEQILHHSMEAAINTLHRVIDDLTLSKSKLQSQLESLTEELAYLKKN
HDEETKGAPKATR-GQV-SVELKA-TPGTDLTKILNDMRAQHEALAQKNRREAE-DYEKLS-
ADLKQRISAGAGRVQTGKSEISELQRTLRALEIELQSQLAMKQSLEVTVGKTEGSYCRKLSRIQVTISSIEEHLAQLKADSESQHSQYQQLLGI
KTRLEQEIETYRRLLDGE
>Frog_krt54
KKETMQNLNDRLANYLDKVNALEAANAELERKIKEWYDKQ-PTTSD---
GGKDYSKYYSLIDELNLKITNATKDNAGITLQADNARLAADDFNNKYENERALYQMMDNDINGLRRVVDDFTLSKFDLESQFEGLFEEMVFIKK
KHDDEIKGTQNTKL-SQV-SVEMKA-QPGTDLTKILNDMRAQHEALAEKNRREAE-DYNTMT-
AELRQQISAGAGQMQASKTEISERKRTLQALETDLHSKLSMKQSLELMLAETEGIYCMKLSRIQVTISSIEEHLAQLKADSESQHSQYQQLLGI
KTRLEQEIETYRRLLDGE
>Frog_krt55
EKETMQNLNDRLANYLDKVNALEAANAELERKIKEWYDKQ-PTTSD---
GGKDYNKYYSLINELNQKILNAALDNAELALHCENAKLAAEDFRLKYESEWTLSQAVVSDINGLRKLMDDLTLSKSSLESQIESLSEELAYLNK
THDEEIKGTQNTKL-SQV-SVEMKA-QPGTDLTKILNDMRAQHEALAQKNRREAE-DYNKMS-
AELRQQISAGVGQVQTSKTETSELKKTLQALEIELQAQIAKKQSLELMLAETEGSYCMKLSRIQVTISSIEEHLAQLKGDSECQRSKYEQLLGI
KIRLEQEIEMYRKLLEGG
```

```
>Frog_krt57
EKQTMQNLNDRLANYLDKVHALELANGELERKIKEWYEKH-GPGTT-
TGEKKDYSKYYTIINDLKAQILVASVENAKLLLQCDNARLAADDFKMKFENEQYMRQTVEADINGLRRVMDDLTLAKSDYESQLESLNEEIAYL
KKNHEDEVKGMQVTQA-GQV-SVEMNA-APGTDLTKLLNDMRNQYEDLAQKNRAEAE-DFNRMS-
ADLKKSISQGVEQQKESKSELTELRRTLQALEIELQSQLAMKKSLESTLAETEGAFCVKLARIQEMIINIEEQIARLKGESECQTAEYQQLLDI
KARLENEIEIYRKLLDGD
>Frog_krt53
EKETMQNLNDRLASYLDKVNALEAANAELERKIKEWYDKQ-
KPSTTTGEGAKDYSKYYKDIEDLKAKIIAASKENAALLLQCDNARLAADDFKQKYESELHLRQTVEADINGLRRVMDELNLAKTDLDSQFQGLT
EELASLKKNHEDEVKGVQVTEA-GKV-KVEMNA-APSNDLTKRLNDMRAQYEDLAKKNRQELE-NFNAAT-
GPVKEKIKEDVKEDTTVKTQISDLKRSLQALEIELQSLLAMKKSLELQLAETEGRFCMKLSVLQENIASIEQHLEQLRAESECQKDEYDQLLEM
KTKLENEIKTYQSLLDKL
>Frog_krt50
EKQTMQNLNDRLASYLDKVHELEGKNAELERKIKDWYEKH-GPAPP--
EAPRDYSKYFKEIEALKAQIIAASKQNAVILLQCDNARLAADDFKQKFENEQAIRYSVEADINGLRRVMDDLTLSRAELEGQLESLTEELAYLK
KNHDDEVRSIKVTEA-GKV-SVEMNA-APSNDLTKRLNDMRAQYEDLARNNRQEAE-NFNRIS-
ADLKNQMHHVQITMDESRTEVTNLKKTLQSLQIELQSQLAQKNSLEILLAETEARFCMKLSHLQETIACVEQRLEQLRAESECQRTEYQQLLEM
KTKLEGEIATYRSLLDKL
>Frog_krt51
EKEAMQDLNGRLASYLDKVHELEAKNAELERKIKEWLDKH-GPAPA--
EAPKDYSKYYKEIEDLKVKIITASKDNAVILLQCDNARLAADDFKQKYESEHVLSQAVEADINGLHKVMDDLNMSKTDLQSQLDGLTEELTYLK
KNHDEEVKAIKAPVA-GTV-SVEMHA-APGSDLTKLLNDMRAQYEDLAQRNRKEAE-DFNKLS-
ADLKKQIDQGQTVVVESSTEVTNLKRALQSLEIELQSLLAQKKSLEMQLAETEGRFCTKLSHIQENIASIEQHLEQLRAESECQKDEYDQLLDL
KTKLEAEIKTYQSLLDKL
>Frog_krt9.2
EKMTMQNLNDRLANYLDKVHALEEANAELEKKIKDWYNNHRPASSK----
APDYGEYFKTIDDLQNKIIAAYANNHKAILAVDNTRLTIDDYRLKYENERALRQSAEADISGLRRVMDDLTLCKSDLEAQVESLTEELAYLKKN
HDEEVKAMQGQAT-GAV-NVEMNV-APSVDLSKSMADMRAEYEKLAERYRREVE-EFQAMT-
KDLQKQVVASVREVQTSKSEITDLRNVLQSLEIELQSQLSMKAALEASLAETEGQYCVKLSHIQELILRVQNNISDIWTQMETQSSEYKLLLDI
KSQLEQEIATYRELLDAQ
>Frog_krt15.1
EKSTMQNLNDRLSNYLEKVRCLEETNADLEKKIHGWHENQ-GSVTV---
HEQNYLPYFATIDELRQKIHAAFIDKNRVLLGLDNARLAAEDFKFKYENEHSMRLNVETDINGLRKLLDEMTLSRADLELQIENLKEELTYMKK
NHQEEMGAKSQQVI-GNV-NVEMDA-PPMNDLTKILSEMRENYEYLMSKNRRDLE-TYLSQS-ESLQNKVEYITE--
QTSKSEITDVRRNVQGLEAELQSQYSKKAAIEDALADTEERYAMQLSHIQSIIGGIEAQLYDLRYDLEHQNQEHKSLLAIKSHLEAEIATYHKL
LEGQ
>Frog_krt34
EKETMQQLNDRLANYLGRVRSLEEENGQFERRIREWYDKQVPYNFP------
DFNNFFRTIEELQNKILHACTGNANLILQIDNARLTSDDFRNKFESEAAFRMGVEGDMNGLRRVLEELNLGNNDQEIQRQNLSEELTYLKKNHA
EEVGSLRTQLG-ARV-NVEVNA-APAVDLNQVLGEIRDQYETIVERNRREAE-NFLNKS-
EELNQQMNSSADQLQTVHTEVIQLRNTVQGLEIDLQSQNSMRSALEGTLADTESRYGGQLSQLQFLINNVETQLGSLRSDLERQNFEYKALMDV
KTHLEMEIATYRRLLDGE
>Frog_krt70
GKETMQNLNDRLASYLERVRSLEQANHELELKIREYCEKKAAIGSP------
DVSGYYNIINLLRSQINDASIDNARLVLQIDNAKLAADDFKIKYESELAIRTGVESDIIGLRRVLDELTLNKTDLELEIESLKEELIYLKKNHE
EELAVARASAR-GQV-DVQVDS-APPVDLSQIMSDVRAQYEGMMEKNRQEVE-AYRGQS-
ENLNKEVATNTAALQTSKSQITDLKRTIQSLEIELQSLLSMKGALEGTLAETEARYGAELNHLQAMITQIELDLQNLRSDAERQSLEYKLLLDA
KTRLEMEISTYRRLLEGE
>Human_KRT20
EKMAMQNLNDRLASYLEKVRTLEQSNSKLEVQIKQWYETN-APRAG-----
RDYSAYYRQIEELRSQIKDAQLQNARCVLQIDNAKLAAEDFRLKYETERGIRLTVEADLQGLNKVFDDLTLHKTDLEIQIEELNKDLALLKKEH
QEEVDGLHKHLG-NTV-NVEVDA-APGLNLGVIMNEMRQKYEVMAQKNLQEAK-EFERQT-
AVLQQQVTVNTEELKGTEVQLTELRRTSQSLEIELQSHLSMKESLEHTLEETKARYSSQLANLQSLLSSLEAQLMQIRSNMERQNNEYHILLDI
KTRLEQEIATYRRLLEGE
>Frog_krt12.2
EKHTMQNLNDRLAAYLDKVRALETANHHLEVQIKEWYEANMHCYKE----
RNDYSKYMVVIEELRRQITNGTPANARILLQIDNAKLAADDFRMKYENELALRQSVESDVNGLRRVLDELTLARSDLELQLEGLNEELAFLKKN
HQEEISSIK--VGSGQV-TVEMDA-APAVDLTLLLNNMRADYEVVAEKNRKEVE-EFLKKS-
AALKQDILIEAQEVQSSHSEISELKRTQQTLEIELQALLSMKSSLEGTLSETEGRYCLSLSQIQMRISSIEEQLHQIRMDMSRQNEDYLRLLDV
KTRLEREIETYRILLEGE
>Frog_krt9.1
EKITMQNLNDRLATYLNKVKSLEEANTDLEKKIRNWYDTH-GPSPV---
QEKDYGPFYRTIEDLQKKILGATIDNGRIVLQIDNAKLAADDFRMKYENELILRQTVESDINGLKRLLDDLTLNRSDLESQLENLKEELAGLKK
NHEEEMKALKVQLV-GDV-TVDMKA-APGIDLQKVLGDLRHDYEQIMQKNQKDIE-FYQEKT-
AELRKNVSGSTQEIQTTNTQVTELRRTLQNLEIDLQAQLSTKAALEASLAETEGRYCLQLSQLQALIQNVEAELANIRCEMENQSHEYKILLDI
KTRLEQEINTYRNLLDGQ
>Frog_krt15.2
EKYTMQNLNDRLASYLAKVKTLEVANTDLEAKIRDWYSKQ-GSVAV---
SEQSFGTLYTSIGELRQKILAATINNSKLVLEIDNARLAADDFKLKYENEFSLRQTVENDISGLRHILDDLTMTRSDLELQIESLKEELIYLKA
NHEEEVSEKKQHAA-GTV-SVELDA-VPGVGLLNTLNDLREQYESIADRNRREAE-AFLSQV-
EGLQKEVVSSTQQIQSTKSESTELRRGLQSLEIELQSQLSAKAGLEASLAETEGRYAAQLFQIQNIISSMEAQLSDLRSDLERQNQEYRTLLDI
KSRLEQEIATYHQLLEGQ
>Alligator_KRT20
EKLTMQNLNDRLASYLEKVHSLEKTNSQLEKQIREWYANN-SSSTR-----
QDYSPYYNTIEELRNKIGAAQLENARLILQIDNAKLAADDFRMKYESELAIRQGAENDTAGLLKVIDDLTLTKADLESQVEGMTEELAFLKKNH
EEEVDRLRKQLG-GTV-NVEVDA-APSIDLAKIMENMRQQYESMAQKNRQEAK-DFDRQT-
EELNQVVAVNTEQLNSKKTEITDLKRTFQSLEIELQSQLSMKKALEDTLAETEARYGSLLAQIQAAIGNVEGQLVQVRADMERQNNEYNILLDV
KTRLEMEIATYRRLLEGE
```

```
>Lizard_KRT10L
EKETMQNLNDRLASYLAKVHALEKANSELELKIKNWYEKN-AFQAD--
GGFRDYSKYYASIDELRNKIVSASVNNSNIILQIDNARLAADDFRMKYENEVFFRQSIEADINGLHKVFDEVILSKSNLELQIESLIEELAFLK
KNHDEEIRGLRGPTS-GNV-SVEMNA-VPGPDLTILLNKMREEYEALAEQNRQDAE-TFVKKS-
NELNTTISMSAKETSTNKKEIAELRRTLQGLEIDLKTQLALRQSLETTLAETEDGYCSQLLQMQGLINNTEAQVKQVRDDMESQNADYRQLLDI
KIHLENEIETYRHLIDSE
>Alligator_KRT10L
EKETMQNLNDRLASYLGKVQALEEANAELEHKIKDWYETL-GSYTE--
GGSRDYSKYYTTIEDLRNQIIAATVENASIILQTDNARLAVDDFKVKYENELYLRQNVEADIRGLRKVLDEQTLAKSNLEIQIESLADEVAYLK
KNHEEEMNDLRSSAS-PDV-NVEINT-APGIDLTTRLNKMRTEYEALAEQNRKDVE-TFNEKS-
KELNVQINFSAEETNTNKSQITELKRTFQGLEIEMKSQLALKQSLEATLAETEARYCAQLSQIQVVISSVETQIQQIRDDMEWQNAEYEQLLDI
KIHLENEIETYRHLIDRE
>Human_KRT26
EKVTMQNLNDRLASYLDHVHALEEANADLEQKIKGWYEKC-EPGSS-
REHDHDYSRYFSVIEDLKRQIISATICNASIVLQNDNARLTADDFRLKYENELALHHSVEADTSGLRRVLDELTLCTTDLEIQCETLSEELTYL
KKSHEEEMEVLQYTAG-GNV-NVEMNA-TPGVDLTVLLNNMRAEYEDLAEQNRKDAE-AFNERS-
ATLQQQISDHEGAATAARNELTELKRNLQTLEIELQSLMAVKHSYECSLAETEGNYCNQLQQIQDQIGVMEEQLQQIRTETEGQKLEYEQLLDV
KIFLEKEIDIYCNLLDGE
>Human_KRT28
EKVTMQNLNDRLASYLDNVRALEEANAELERKIKGWYEKY-GPGSC-
RGLDHDYSRYHLTIEDLKNKIISSTTTNANVILQIDNARLAADDFRLKYENELTLHQNVEADINGLRRVLDELTLCRTDQELQYESLSEEMTYL
KKNHEEEMKALQCAAG-GNV-NVEMNA-APGVDLAVLLNNMRAEYEALAEQNRKDAE-AFNEKS-
ASLQQQISHDSGAATFARSQLTEMRRTLQTLEIQLQSLMATKHSLECSLTETESNYCTQLAQIQAQIGALEEQLHQVRTETEGQKLEYEHLLDV
KVHLEKEIETYCRLIDGD
>Human_KRT25
EKVTMQNLNDRLASYLDSVHALEEANADLEQKIKGWYEKF-GPGSC-
RGLDHDYSRYFPIIDDLKNQIIASTTSNANAVLQIDNARLTADDFRLKYENELALHQSVEADVNGLRRVLDEITLCRTDLEIQYETLSEEMTYL
KKNHKEEMQVLQCAAG-GNV-NVEMNA-APGVDLTVLLNNMRAEYEALAEQNRRDAE-AFNEKS-
ASLQQQISEDVGATTSARNELTEMKRTLQTLEIELQSLLATKHSLECSLTETESNYCAQLAQIQAQIGALEEQLHQVRTETEGQKLEYEQLLDI
KLHLEKEIETYCLLIGGD
>Human_KRT27
EKVTMQNLNDRLASYLENVRALEEANADLEQKIKGWYEKF-GPGSC-
RGLDHDYSRYFPIIDELKNQIISATTSNAHVVLQNDNARLTADDFRLKFENELALHQSVEADINGLRRVLDELTLCRTDLEIQLETLSEELAYL
KKNHEEEMKALQCAAG-GNV-NVEMNA-APGVDLTVLLNNMRAEYEALAEQNRRDAE-AFNEKS-
ASLQQQISDDAGATTSARNELIEMKRTLQTLEIELQSLLATKHSLECSLTETESNYCAQLAQIQAQIGALEEQLHQVRTETEGQKLEYEQLLDI
KVHLEKEIETYCLLIDGE
>Frog_krt19
EKETMQNLNDRLAMYLDKVRALEEANSGLELKIREWYEKQ-GPSPS-----
RDYSHYFAIIEDLRGKILDATVDNSKIVLQIDNARLAADDFRTKYENELALRQSVECDINGLRKVLDELTLCRTDLEHQIESLKEELAYLKKNH
EEEMNAMRGQVG-GQV-SVEVDA-APTVDMNKKLAEMREQYELLAEKNRRDAE-AFFSQS-
EELNKVVATHTEQIQTSKSEITDLRRTIQGLEIELQSQLSMKAALENTLSETEARYGMQLAQIQNVIGSIEAQLADLRSDMERQNHEYKLLMDI
KTRLEKEISTYRQLLEGH
>Frog_krt17
EKYTMQNLNDRLAAYLEKVRSLEKANGELEIKIRDWYQKQ-APGPA-----
ADYSHYYKTIEDLRNKILSATMDNAGLLLQIDNAKLAADDFRTKYETELALRMNVEADINGLRRVLDDLTINRSDLEIQIESLKEELAYLKKNH
EEEMIALRGQAT-GQV-NVEMDA-APSSDLTKTLAEMRIQYENLAEKNRRDVE-EFFKKT-
EELNKEVANSSVMIQSSKTEITDLKRTLQSLEIELQTQLSMKAALEGSLAETEGRYCVQLSQIQGLISSVEAQLGELRSDMERQSHEYKILMDV
KTRLEQEIATYRRLLEGE
>Human_KRT19
EKLTMQNLNDRLASYLDKVRALEAANGELEVKIRDWYQKQ-GPGPS-----
RDYSHYYTTIQDLRDKILGATIENSRIVLQIDNARLAADDFRTKFETEQALRMSVEADINGLRRVLDELTLARTDLEMQIEGLKEELAYLKKNH
EEEISTLRGQVG-GQV-SVEVDS-APGTDLAKILSDMRSQYEVMAEQNRKDAE-AFTSRT-
EELNREVAGHTEQLQMSRSEVTDLRRTLQGLEIELQSQLSMKAALEDTLAETEARFGAQLAHIQALISGIEAQLGDVRADSERQNQEYQRLMDI
KSRLEQEIATYRSLLEGQ
>Lizard_KRT19
EKITMQNLNDRLASYLDKVHALEESNADLEVKIREWYQKQ-GPTSP----
ARDYSHYYKTIEDLRDKILDATIDNNKIVLQIDNARLAADDFKTKFESEQALRMSVEGDINGLRRVLDELTLARSDLELQIETLKEELAYLKKN
HEEELGALSGHLG-GQV-SVEVDS-APGIDLTKILAEMRDQYELLADKNRRDAE-AFTTKT-
EELSREVAVNTEQLQSSKTEITDLRRTLQGLEIELQSQLSMKAALEGTLADTENRYSAQLCQIQNLISNIEAQLVEVRSDMERQNSDYKMLMDI
KSRLEQEIQTYRQLLDGH
>Alligator_KRT19
EKITMQNLNDRLASYLDKVRALEEANSELEIKIRDWYQKQ-GPGPS-----
RDYSPYYKTIEDLRDKILAATIDNSKIVLQIDNARLAADDFKTKFETEQGLRLSVEADINGLRRLLDELTLARTDLEMQIENLKEELAYLKKNH
EEEMNAHAGQAG-GQV-SVEVDS-APGIDLTKILAEMRDQYEFMAEKNRKDAE-GFNSKT-
EELNQEVAVNTEQLQTSRTEITDLRRTLQGLEIELQSQLSMKAALEGTLADTENRYGAQLSQIQCLISNIEAQLGDLRAEMERQNSEYKMLMDI
KTRLEQEIATYHQLLEGQ
>Lizard_KRT14L2
EKETMQNLNDRLATYLDKVRALEHANTELEVKIREWYQKR-GPGPQ-----
RDYSSYFRTIEDLRSKILAASVQNATILLQLDNAKLTADDFRTKYETEHALRTSVEADIHGLRKVMDELTLTRSDLEMQLENLKEELAYLHKNH
EEELKVLRSQMG-GEI-TVEMDA-APGVDLTKILAEMREQYEHLAEKNRKDAE-QFFSKT-
EELNQEVAINTEQLQSGKSEVIELRRTLQGLEIELQTQLSMKSSLEGTLAETESRYGLQLNQLQALITSVEEQLAELRCDMERQNSEYKILLDV
KTRLEQEISTYRRLLEGE
>Lizard_KRT14L1
EKETMQNLNDRLASYLDKVRALEEANTDLEVKIKEWYKKQ-GPSPE-----
RDYSPYFRTIEELRSKILAATVDNASIVLQIDNARLAADDFRNKFETEHALRVNVEADINGLRRVLDELTLARADLEMQIENFKEELVYLRKNH
EEEMSIFRSQSG-GEI-SVEMDA-APGVDLTKILSEMREQYESLAEKNRKDAE-QFFSKT-
EELTREVAINTEQLQSGKTEITELRRTYQNLEIELNAQLSMKAALESSLADTEARYGAQLSQIQVLITNVEEQLADLRCEMERQNHEYKMLLDV
KTRLEQEIATYRRLLEGE
```

```
>Alligator_KRT14L1
EKETMQNLNDRLAAYLDKVRALEEANSELEIKIRDWYKKQ-GPGPD-----
RDYSPYYRTIEDLRNKILAATVDNANLILQIDNARLAADDFRTKFETEQALRMSVEADINGLRRVLDELTLARADLEMQIENLKEELAYLKKNH
EEEMNALRGQVG-GEI-TVEMDA-APGVDLTKILTEMREQYEHLADKNRREAE-QFFKQT-
EELNQEVAINTEQLQSSKSEITELRRTIQGLEIELQSQLSTKAALEGTLADTEARYGSQLAQIQGLITSVEEQLAELRCDMERQNHEYKILLDV
KCRLEQEIATYRRLLEGE
>Alligator_KRT14L2
EKETMQNLNDRLAAYLDKVRALEEANSELEIKIRDWYKKQ-GPGPD-----
RDYSPYYRTIEDLRNKILSATVDNANLLLQIDNARLTADDFRTKFETEQALRMSVEGDINGLRRVLDELTLAKADLEMQLENLSEELAYLKKNH
EEEMNALRSQVG-GEI-TVEMDA-APGVDLTKVLAEMREQYEHLADKNRREAE-QFFKQT-
EELNKEVAINTEQLQSGKSEIMELRRTIQGLEIDLQSQLSMKAALEGTLADTEARYGSQLAQIQGLITSVEEQLAELRCDMERQNHEYKILLDV
KCRLEQEIATYRRLLEGE
>Human_KRT16
EKVTMQNLNDRLASYLDKVRALEEANADLEVKIRDWYQRQRPSEIK------
DYSPYFKTIEDLRNKIIAATIENAQPILQIDNARLAADDFRTKYEHELALRQTVEADVNGLRRVLDELTLARTDLEMQIEGLKEELAYLRKNHE
EEMLALRGQTG-GDV-NVEMDA-APGVDLSRILNEMRDQYEQMAEKNRRDAE-TFLSKT-
EELNKEVASNSELVQSSRSEVTELRRVLQGLEIELQSQLSMKASLENSLEETKGRYCMQLSQIQGLIGSVEEQLAQLRCEMEQQSQEYQILLDV
KTRLEQEIATYRRLLEGE
>Human_KRT14
EKVTMQNLNDRLASYLDKVRALEEANADLEVKIRDWYQRQRPAEIK------
DYSPYFKTIEDLRNKILTATVDNANVLLQIDNARLAADDFRTKYETELNLRMSVEADINGLRRVLDELTLARADLEMQIESLKEELAYLKKNHE
EEMNALRGQVG-GDV-NVEMDA-APGVDLSRILNEMRDQYEKMAEKNRKDAE-EFFTKT-
EELNREVATNSELVQSGKSEISELRRTMQNLEIELQSQLSMKASLENSLEETKGRYCMQLAQIQEMIGSVEEQLAQLRCEMEQQNQEYKILLDV
KTRLEQEIATYRRLLEGE
>Human_KRT17
EKATMQNLNDRLASYLDKVRALEEANTELEVKIRDWYQRQ-APGPA-----
RDYSQYYRTIEELQNKILTATVDNANILLQIDNARLAADDFRTKFETEQALRLSVEADINGLRRVLDELTLARADLEMQIENLKEELAYLKKNH
EEEMNALRGQVG-GEI-NVEMDA-APGVDLSRILNEMRDQYEKMAEKNRKDAE-DFFSKT-
EELNREVATNSELVQSGKSEISELRRTMQALEIELQSQLSMKASLEGNLAETENRYCVQLSQIQGLIGSVEEQLAQLRCEMEQQNQEYKILLDV
KTRLEQEIATYRRLLEGE
>Human_KRT13
EKITMQNLNDRLASYLEKVRALEEANADLEVKIRDWHLKQ-SPASP----
ERDYSPYYKTIEELRDKILTATIENNRVILEIDNARLAADDFRLKYENELALRQSVEADINGLRRVLDELTLSKTDLEMQIESLNEELAYMKKN
HEEEMKEFSNQVV-GQV-NVEMDA-TPGIDLTRVLAEMREQYEAMAERNRRDAE-EFHAKS-
AELNKEVSTNTAMIQTSKTEITELRRTLQGLEIELQSQLSMKAGLENTVAETECRYALQLQQIQGLISSIEAQLSELRSEMECQNQEYKMLLDI
KTRLEQEIATYRSLLEGQ
>Human_KRT15
EKITMQNLNDRLASYLDKVRALEEANADLEVKIHDWYQKQ-TPTSP----
ECDYSQYFKTIEELRDKIMATTIDNSRVILEIDNARLAADDFRLKYENELALRQGVEADINGLRRVLDELTLARTDLEMQIEGLNEELAYLKKN
HEEEMKEFSSQLA-GQV-NVEMDA-APGVDLTRVLAEMREQYEAMAEKNRRDVE-AFFSKT-
EELNKEVASNTEMIQTSKTEITDLRRTMQELEIELQSQLSMKAGLENSLAETECRYATQLQQIQGLIGGLEAQLSELRCEMEAQNQEYKMLLDI
KTRLEQEIATYRSLLEGQ
>Lizard_KRT15
EKVTMQNLNDRLATYLDKVRALEEANTELEVKIRDWHQKQ-APKSP----
SRDYSQYYKIIEDLRDKILGTTIDNSRVILEIDNARLAADDFRLKYENELHLRQSVEADINGLRRVLDELTMSKSDLEMQIEGLKEDLAYLNKN
HEELMKEFSTQLT-GKV-SVEMDA-APGIDLTKILADMREQYEHLAEQNRKDAE-TFFSKT-
EELNREVATNTEQIQTSKSEISELRRTIQGLEIELQSQLSMKAGLESNLAETEGRYCAQLAQIQNLITGVEEQLAELRNDMERQNQEYKMLMDI
KTRLEQEIATYRNLLEGQ
>Alligator_KRT15
EKITMQNLNDRLASYLDKVRALEEANADLEVKIRDWYQKQ-GPTSP----
ARDYSPYYKTIEDLRDKILAATIDNSRIILEIDNARLAADDFRMKYENELYLRQSVEADINGLRRVLDELTLARADLEMQIENLKEELAYLKKN
HEEEMREYSNQMT-GKV-SVEMDA-APGVDLTKVLAEMREQYEALADKNRREAE-AFFTKT-
EELNREVATSTEQIQTSKSEMTELRRTLQGLEIELQSQLSMKAGLEANLADTESRYCAQLAQLQAMITSIEEQLAELRCDMERQNQEYKMLLDI
KTRLEQEIATYRRLLDGQ
>Lizard_KRT24L
EKHTMQNLNDRLANYLGKVHALEEANAELELKIKDWYAKCSIPDTS-----
RDYTKYFNIIEDLRGKILRQTIENAGIVLQIDNARLAADDFKLKYENELFLHQNVENDINGLRRVLDDLTMHRSDLELQIEGLNEELAYLKKNH
EEELKSFRGASS-GDV-SVEMDA-APGVDLTKLLNSMRSQYEELAEKNRQEAE-DFNKQV-
GALRQEISANVDQLSTGKTEITDLRRTLQGLELELQAQLAMKQSLEGTLAETERNYCDQLAQIQSQIAGLEEQLLQLRADMENQNSDYQHLLGI
KTRLEQEIETYRRLLDGE
>Frog_krt12.6
EKHTMQNLNDRLANYLDKVKALETDNNDLEKKIREWYEKLRPESGG--
VGTVDYSKYLPIIEDLRKKIMESTLENAKILLQTDNARLAADDFRLKYENELALRQSVEADINGLRRVLDELTLCKADLELQIESLTEELAYLK
KNHKEELDALRGGPA-GQL-TVEMNA-APAVDLTKLLNDMREQYETLAEKNRRETE-AFNEQS-
KDIKKEILAGVQQVQSNTTEISDLKRSLQGLEIELQSQLAMKQSLEKTLAETEGRFCSQLGQLQNLITSVEEQLFQLRNDMELQSNEYKQLLDI
KTRLEQEIETYRKLLEGE
>Frog_krt24
EKETMQNLNDRLAAYLNKVRALEEANSDLECKIREWYEKHRNACMP----
DRDYSKYYHIIEDLKKQIQCASVENARVILQIDNARLAADDFKLKYENELCLRHGVEADINGLRKVLDELNLAKCDLESQIESLTDEIACLKKN
HEEEMKGYQGAT--GHL-SVEMKA-APGNDLTKHLNDMRAEYEELAEKNRKEAE-AFNEAS-
RALKQEISCGAEQIQCSKNEITDLKRNLQALEIDLQAALAMKKTLECSLAETEGNYCVQLSKIQAKISALEQQLCEVRTDMERQSIEYEQLLDI
KTRLEMEIETYRRLLDGE
>Alligator_KRT24
EKETMQNLNDRLANYLDKVHALEEANADLEYKIHQWYEKH-GPAAA--
ELTRDYSKYYQIIEDLRNQIISATIGNASIVLQIDNSQLAAEDFRLKYENELFLHRSVEADSNGLRRVLDDLTIAKSNLEAQLESLTEELVYLK
KSHEEEMKSLKSTST-GDV-NVEINA-APGIDLTKLLNDMRAQYESLAEQNRREAE-EFNKMS-
QPLQKQIYDDAGAVSSATNELTEMKRTIQGLEIELQTVMAQKCSLEGTLARTEENYSAQLSQRQLHISNLEEQLQQIRSEMECQNAEYEQLLGI
KIRLEMEIETYKRLLDGE
```

```
>Lizard_KRT24
EKETMQNLNNRLANYLEKVRSLEEANAELEVKIRHWYEKN-GPGAP--
GVVRDYTKYHQIIEDLRNKIVNTTIDNANVVLQIDNARLAADDFRLKFENELFLRQSVESDINGLRRVLDELTMNRSDLEAQLENLTEELAYLK
KNHEEEINMLKSNST-GDV-TVEMNA-APGIDLTKLLNDMRAQYEDLAEQNRREAE-EFHKMS-
QPLQQQIYDDAGAVSSARNELIELKRSFQSLEIELQSLLAKKASLEGTLTETEANYSSQLHQLQLQVSNLEEQLQQIRSETECQNSEYQQLLGI
KTRLEMEIETYRRLLDGE
>Human_KRT24
EKQTMQNLNDRLANYLDKVRALEEANTDLENKIKEWYDKY-
GPGSGDGGSGRDYSKYYSIIEDLRNQIIAATVENAGIILHIDNARLAADDFRLKYENELCLRQSVEADINGLRKVLDDLTMTRSDLEMQIESFT
EELAYLRKNHEEEMKNMQGSSG-GEV-TVEMNA-APGTDLTKLLNDMRAQYEELAEQNRREAE-EFNKQS-
ASLQAQISTDAGAATSAKNEITELKRTLQALEIELQSQLAMKSSLEGTLADTEAGYVAQLSEIQTQISALEEEICQIWGETKCQNAEYKQLLDI
KTRLEVEIETYRRLLDGE
>Frog_krt12.5
DKQTMQNLNDRLAAYLEKVRALEAANADLELKIREWYEKQRTSGIG--
AGAKDFSKYFDIITDLRSKILFATIDNSRVVLQIDNAKLAADDFRLKFENELALRQSVEADINGLRRVLDELTLARGDLEMQIESLTEELAYLK
KNHEEEMSIAKSSAA-GQV-TVEMDA-APGTDLTNILNNMRADYETLAEKNRRDAE-LFNQKS-
GELKKEILTGVELVQSSKSEISDLRRSLQSLEIELQSQLAMKKSLEDSLAETDGRYGAQLQNIQLSIRSLEEQLLQIRSDMERQNMEYKQLLDI
KTRLEMEIETYRRLLEGE
>Frog_krt12.4
EKHAMQNLNDRLASYLDKVKALEASNVTLEGKIHDWYDSKVDAGIG--
AGSKDYSKYFEIINELRSKIGAATVGNATVTLQIDNARLAADDFRLKFENELALRQSVEADIAGLRRVLDELTLARGDLEMQIESLTEELAYLK
KNHEEEMSHAKSQSA-GKV-SVEMDA-APGVDLTNILNNMRADYEILAEKNRRDAE-LFNQKS-
GELKKEISVGVEQVQASKSEITELKRSLQSLEIELQSQLAMKQSVEGNLSEVQGFYSAQLLQIQNTISSLEEQLLQIRSDMEHQNMEYKQLLDI
KTRLEMEIETYRRLLEGE
>Frog_krt12.3
EKHAMQNLNDRLASYLEKVRALEASNADLEGKIRTWHEKQTGTGLG--
AGSKDYSKYFEIISELRGKIHGATVDNATVTLQIDNARLAADDFRLKFENELALRQSVEADIAGLRRVLDELTLARGDLEMQIESLTEELAYLK
KNHEEEMMHAKSQSA-GKV-SVEMDA-APGVDLTNILNNMRADYEILAEKNRRDAE-LFTQKS-
GELKKEISVGVEQVQASKSEITELKRSLQSLEIELQSQLAMKQSVEGNLNELQGFYSAQLLQIQNTISSLEEQLLQIRSDMEHQNMEYKQLLDI
KTRLEMEIETYRRLLDGE
>Human_KRT10
EKVTMQNLNDRLASYLDKVRALEESNYELEGKIKEWYEKH-GNSHQ--
GEPRDYSKYYKTIDDLKNQILNLTTDNANILLQIDNARLAADDFRLKYENEVALRQSVEADINGLRRVLDELTLTKADLEMQIESLTEELAYLK
KNHEEEMKDLRNVST-GDV-NVEMNA-APGVDLTQLLNNMRSQYEQLAEQNRKDAE-AFNEKS-
KELTTEIDNNIEQISSYKSEITELRRNVQALEIELQSQLALKQSLEASLAETEGRYCVQLSQIQAQISALEEQLQQIRAETECQNTEYQQLLDI
KIRLENEIQTYRSLLEGE
>Human_KRT12
EKETMQNLNDRLASYLDKVRALEEANTELENKIREWYETR-GTGTA-
DASQSDYSKYYPLIEDLRNKIISASIGNAQLLLQIDNARLAAEDFRMKYENELALRQGVEADINGLRRVLDELTLTRTDLEMQIESLNEELAYM
KKNHEDELQSFRVGGP-GEV-SVEMDA-APGVDLTRLLNDMRAQYETIAEQNRKDAE-AFIEKS-
GELRKEISTNTEQLQSSKSEVTDLRRAFQNLEIELQSQLAMKKSLEDSLAEAEGDYCAQLSQVQQLISNLEAQLLQVRADAERQNVDHQRLLNV
KARLELEIETYRRLLDGE
>Lizard_KRT12
EKETMQNLNDRLAAYLDRVHSLEEANTELERKIREWYEKN-
GPGTGALGAGRDYSKYYPVIEDLRNKIINATIENARIVLQIDNARLAADDFRLKFENEVALRQSVEADINGLRRVLDELTLTRADLEMQIETLN
EELAYLKKNHEEELIGFQSNAT-GQV-SVEMDA-APGVDLTKLLNEMRAQYESIAEQHRKEAE-AFNEKS-
GELKREISTHSEQLQYGKSELTDLKRTLQSLEIELQSQLALKKSLEDTLAETEAGYCTQLAQMQLQIGNVESQLFQVRADMERQNAEYQNLMDI
KTRLEMEIETYRRLLDGE
>Alligator_KRT12
KKETMQNLNDRLAAYLDKVRSLEEANTELEHKIREWYEKN-
GPGAGSLGAGNDYSKYYPIIEDLRNKIINATIDNARIVLQIDNARLAADDFRLKYENEVALRQSVEADINGLRRVLDELTLTRADLEMQIESLN
EELAYLKKNHEEELQGFQGNAT-GQV-SVEMDA-APGIDLTKLLNDMRGQYEAIAEQNRKDAE-AFNEKS-
GELKKEISTNTEQLQSGRSEITDLKRTLQSLEIELQSQLAMKKSLEDTLAETEAGYCTQLSQMQLQIGNLENQLFQVRADMERQNAEYQQLLDI
KTRLEMEIETYRRLLDGE
```

Figure S9. Multiple sequence alignment used for the type I keratin phylogeny of amniote species and sea lamprey in Figure 2. The trimmed amino acid sequences are provided in fast_align format. Species: alligator (*Alligator sinensis*), frog (*Xenopus tropicalis*), hagfish (*Eptatretus burgeri*), human (*Homo sapiens*), lamprey (*Petromyzon marinus*), lizard (*Anolis carolinensis*).

>Frog_krt80
EEAETK-LRAALATFNDKIRYLEKQREILESRWSMIQTE------ETTQ-TDLEP--
QYLSYISRLLGEVKNVTQNNHQTQRQLLDMMDSVNDMKDKFEDELFRRTDVEYSFVELKKDADGRSLEQTELETRKQDIKEMIDLMKTVYKQEL
KELMEDSG--DISVLVNMEQSC-PLNLDKVVEEVKAQYERIASWSRDEAQA-
SKNKLAEGVQRVGRYELELKSSRSEITHLNSKIQRLRSEIQSIQKQCSKLEQDVSLAQTNSNMAIVDAN-
KVTEIQDVLLKAKQDMAKQLRDYQELLHVKMALDVEIAAYNLLLEGE
>Hagfish_krt8.10
EKDAIKHLNNRFANLIEKVSTLQQQNKVLEAQWFALQQK------VPAD-VGVEK--
GFQKYIDVLQQQLNTLHDKKSQLQSHLLQTRESVQANKGRYETEIDKRTQKDLGLLEIKKDVGIFRFKKTEKECEKKILVDEIDFLKMLFAKEI
QDLELQIK--DASLFVSVDTNH-
SLDFEGIISEVRAEYDAITAQSQANAQDFYRKKLLDISTSADNANNQLHEAKAEMVKMSLQIKRMKAQLDSLKKQL------------------
----
>Lamprey_krt8.2
EKNQLMGLNDRFANYIQNVRILERKNKELQVSLKRMRIH------SGQD-SQLDN--
LCAQYEVLLKRQIQALIEEKRRLANDQEQAHINMEQMKIKYETEIRERTQIENEFVVLKQEADQVYMQKVELEARLTGLTDDIDFYRKVYEREI
RELEARMV--KVDVVVEVDSSP-GLDLDTYLAEVREQYMKQAGRIREELKI-
FETKLDANRNNEVKINDDMRLVTVEISEVRRNMQRYKAELEALKQQCLALERAIALAEEKGKESMRQLTEKRQLLEVTIMEQKNKLTGHHRSYQ
ELMNVKLALDMEIIAYRKLLEGE
>Human_KRT80
EKEEMKALNDKFASLIGKVQALEQRNQLLETRWSFLQGQ------DSAI-FDLGH--
LYEEYQGRLQEELRKVSQERGQLEANLLQVLEKVEEFRIRYEDEISKRTDMEFTFVQLKKDLDAECLHRTELETKLKSLESFVELMKTIYEQEL
KDLAAQVK--DVSVTVGMDSRC-HIDLSGIVEEVKAQYDAVAARSLEEAEA-
SRSQLEEQAARSAEYGSSLQSSRSEIADLNVRIQKLRSQILSVKSHCLKLEENIKTAEEQGELAFQDAK-
KLAQLEAALQQAKQDMARQLRKYQELMNVKLALDIEIATYRKLVEGE
>Lizard_KRT80
EKEEIKTLNNQFASLIGKVQNLEQHNQVLLTRWNFLREQ------DNSL-
SDLDIKLLYDQYMNRLRLEIRSIDNEKEQLDAELDEVLDAMDNVRNSYEEEINKRTGMEFTFTALKKDLDNGFLHKTELEAKLSGLHAWVELMK
NIQEQELEEVMSQVK--DVSVVLGIDNNRYNPDPHRIVEDVRAQYEALAIRSWEELEA-
TRSKLNEREVLSVKYGDHLLHDRRAIAELNIQIQKMRSCILSLKSQCLRLEDNIKDVGLQGETALNDAKAKLAKLEEALHNAKQDLAQLVKQYQ
ELMNIKLALDIEILTYRKLMEGE
>Alligator_KRT80
EKEEIKVLNNQFVALIKQVQSLELQNKMLETRWDFLKDQ------DNSS-
SEVDIKCIYDEYMGRMEQEMKSIDQEKEELESELTKVLDSMDDFRAKYEGEMQLRNSLEYTFLELKKDLDAGTLHRTELEVKLNGLQEHMELKK
SVYEQELQELLAEVK--DISVVLGIDTRC-NLDLHSIVEEVRAQYEALAVWSWKEAEA-
IWSKLNEGVPRSAMYGNHLFNSRREIADLNIQIQKLRSCIVSLKSQCLSLEESIKEAGEQGEFALQDASAKLASLEEALQKGKEDVTHLVKEHQ
QLMNTKLALDVEILTYRKLVEGE
>Alligator_KRT8L2
EKEELKTLNNQFVHLIERVCSLEQHKKVLETKCSILKSQ------KATP-ARMEN--
LLKTYISQLKKHLADQGQMKGKLHLELHEIQDIMDENKQKYEEEFNLRNEKENDFLLMKKEMDEAYISQNELEGKLDRLRDELEFLKNFYDQEM
HELQTEVHMYKESVVISMPSQP-SLEVDSIVSEAQSQYEKMAERSCKEAEK-
YRDRHNKLKDSMGRHGEELQTMKTEINDLTRKSQHIQLEIQNLKEQCNKLKEDIANADDRYKFPIEEAKNKLHMLENAIQKARQDMAHHLREYQ
ELLNTKLGLDIEIATYRKLLEGE
>Lamprey_krt8.9
EKNAISHLNDRFASFIDKVRYLEQENKVLEAQWGCLQAR------VCT--SDLDS--
MFETYAQAIKRQLDCVLADRPRLETELHQTRALADEHKLKYEAEVTGREAAEANFVAIKREADDNYMGKVQMETRVGQLSDELHFIKELFACEM
QEMEERIR--DMNVTIDLDTGC-NFDLSSLIAEVRANYEMVAARSREEVEC-
YKSKMDDMAEASERHCIELRCTKNEILELSSMLQRLACEIDGLKTQRYQLESSIQEAETRGELNAHEARDATARVEMELSQAKADMARHVRDYQ
ELMNVKLALDIEIATYRKLLEGE
>Hagfish_krt8.8
EKEGMKCGNDRFASFIDKTRFLEQQNKVLEAQWECLQNK------TCT--SKLDC--
MFEQFANRLKEQLECLVQDRPRLETEMNQTQSLANDYRSKYEEEIALRTQLENEFWALKQDVDCKYLEKVRLETRLAQLNDEVLFLRTLFEQEL
QEMHERIR--DMSVSVELDVAP-
NLDLTSLIAEVRCNYEAIAARSRQEVECWYKSKLDDINEASERNCCELRCTKNEVADLARNIQRLRCEIETQARQRSQLEATIQDAEARGQASV
LQGRETIARLENELQSAKQEMAKHVRDYQELMNVKLALDVEIATYKKLLEGE
>Hagfish_krt8.7
EKDALKVLNDRFASFIERVRILEQQNKILQAQDNHFQTS------GSD--TNVGA--
MFEAFIRTLRKKLDCITGDKPKLESELQQMLAVVDELKVKYEKEYDLRTKAENEFVATKKDVDVAYLEKIGLETRAAQLLDEINFLR----
EEICELQDRTK--DTGVQVTISQGL-NADLSTLIAEVRAQYEAIAAKSRQEVECWFKGKV------CGKRD------
KSEITEITRQIQRIRAEMDVQKKQHTQIEQAIKEAEDRGSISVREGKEAIGRLEKELQQSRQEMARHVRDYQELMNVKLALDVEIATYRKLLEG
E
>Lamprey_krt8.7
EKDQIRGLNDRFANFIDKVRSLEQQRSVLDAQWKVLQAK------GEDK-SNLED--
IYQEYIRGLRRQLEMLQENKEHLQSDVGHMQGVVEEFKNKYETELNNRNHAENEFVLIKKDFDDAHLNKVELEARLEGLTDEIDFLRRIYEEEL
RELHAQMN--NISLTVEVDTNR-HFNMDDIVASVRSQYEALAQQSRQEAED-
YRVKFEDINASADKSNEDIRNSKQELNDLLRTIKNLTSEVQRLKQQRGQAERAVAEAEDLGEQALKDAKKRIADLEQELADSRRQMAQHVRDYQ
ELMNVKLALDIEIATYGKLLEGE
>Hagfish_krt8.3
EKEAIKKLNDRFASFIDKVRFLEQQKTVLEAQWKALQER------GSAS-LHLDN--
LFEPYVALLRQRFDELALGQPRLESELQQMQGAVEDLRCKCD----IRTCKIFLYNLSLQEVDESYLMKVELEGILDGLTEDISFYKEIF--
EMHQLEAQIR--DTSLYIEVDTRR-
NLDIGGLIADVRAQYETIAAKSKAEAEEFYREKFVSFNASAGKNEEELRLIKHDISESLRQLQRVKAEIEALKKQQAHLETAIAETEERGEMDI
REAKSTMSHLENELQCTKQEMAKHVRDYQELMNVKLALDIEIATYRKLLEGE
>Lamprey_krt8.8
EKEEIKMLNNRFANFIDKVRILEQQNKVLEAQWYVLQDK------GKVD-CNLDE--
LFEVYIEELRRQLQNLGHNGARMNSDLQMIKHAGKDFSSKYEVEVAAHKKKVEEHELVKKEAVEASFVKIELEAKFQGLIEDIHFLREAFAQEL
RELEAQIK--NTNVLVEIDNRR-SLDMDGIISEVRKQYEFIMSQSQAEAEA-
YKQKFDKLRSESDRSDEELRKAKAEINDIKRQIHQINSELDILSKQREKLEAAISEREEGGAKNIGEAKAAVAQLEEALAKAKHDMVKHVRDFT
DLMNIKLALDTEILTYRKLLEGE

```
>Lamprey_krt8.1
EKEQIKKVNDRFVRFIEKVGILEQQNKVLEVQWLLLQDR------GARG-
SKVEASVFFQSQIHGLQRQLDTLERDKQRRQGELRLTQGLVEDFKNKYEEDKSIRNKAENEFVAVKQDFDDSCLVKAELEARLEGLKDEINFLR
GIFEKELRDLETQIK--DTLVSAGVDTSR-SIDVQGMISDVHAQYELIAAKSQAEANE-
YRKKYDLLSLSADPTDQEIRSNRNEMNNLHRQIQRVRGESDALKKQRASLQAAIAGAVDRGGLSVCEAKERIARLEEELHKDKQQKVQRVREYQ
ELMNVKLALDIEIATYGRLLEGE
>Hagfish_krt8.4
EKEQIKGLNDKFATFIGKVQQLEQQKQVLEAQWRALQQR------GGAS-VNVDG--
MFQSYINSLKQQLEGLGQDKMRLHGELQQMQGLVEDFKSKYEEEINNRTQKENEFVVVKKDVDDAYLAKVELEAKLDGLQDEIKFLKDIYAEEI
SQLEQQIK--DTSLFVSVDTTR-
NLNVADIINESQRHYDDIAESSKREASRFYQEKLNEQVSCAPSGEDDIRKCRSEINELNQSMQHMKAEIENLKKRRANLEAQIAEAEEQGDVQI
AETKAQITELEKDLHKCKDDMAAQLKEYHALMNVKLALDIEIATYRSLLEQE
>Lamprey_krt8.3
EKEAIKHLNDRFANFIDKVRFLEQQNKVLEAQWYALQEK------TTTG-SSVDE--
MFEAYINGLRRQLDGLGHENGRANGDLGQMQAVVEDFMSKYEAEITARIHKENEYVVIKKDVDAANVIKIELEAKMQGLVEDINFLREIFAQEL
SELEAQIK--HTNVLVEIDNSR-NLDVDGIIADVRHQYETIAARSRAEADA-
YQDKFDRLHADSGRNDEEMRVIRTEINDNNRHIHRMRAEIDALKKQRAKLEAAIAEAEGRGEADIREAKAAIAQLEEEIHKAKQEMARHVREYQ
ELMNVKLALDIEIVTYRKLLEGE
>Lamprey_krt8.4
EKEAIKHLNDRFANFIDKVRFLEQQNKVLEAQWYALQEK------TTTG-SSVDE--
MFEAYINGLRRQLDGLGHENGRANGDLGQMQAVVEDFRAKYEAEISLRTEREDEFVFAKKDVDGAYLSKVELEAKLDGLQEDINFMRSIFAQEL
SELEAQIK--HTNVLVEIDNSR-NLDVDGIIADVRHQYETIAARSRAEADA-
YQDKFQSLHSDSGRNDEELRVTRNEITELNRQMQRFRAEMEALKKQRAKLESAIAEAEGRGEADIREAKAAIAQLEEEIHKAKQEMARHVREYQ
ELMNVKLALDIEIVTYRKLLEGE
>Lamprey_krt8.5
EKEAIKHLNDRFANFIDKVRFLEQQNKVLEAQWYALQEK------TTTG-SSIDE--
MFEAYINGLRRQLDGLGHDKGQLNGNLGQMQAVVEDFRAKYEAEINNRNAAEGEFVVVKKDVDGAYLSKVELETRLQGLVDEINFLRSIFTQEL
SELEAQIK--HTNVLVEIDNSR-NLDVDGIIADVRQQYETIAARSRAEADA-
YQDKFQSLHSDSGRNDEELRMTRNEITELNRQMQRFRAEMEALKKQRAKLESAIAEAEGRGEADIREAKAAIAQLEEEIHKAKQEMARHVREYQ
ELMNVKLALDIEIVTYRKLLEGE
>Hagfish_krt8.1
EKEQIKTLNNRFASLIDKVRYLEQHNKVLDAQWRTLQER------STAT-SNVDA--
MFEAYINGLKNQLEGLGTDKLRLNGELQQMQALVEDFKVKYEQEITNRTQVENEFVVVKKDVDDAYLAKVELEAKLEGLQDEINFLKEVFSEEL
RQLEAQIR--DTSLYVEVDTRR-
NLDINGLIADVRAQYDAIAAKSRADAEDFYKVKFADLTSMTGKTDDEMRLMKGEMNDLNRQIQRINAEIAALKKQRAQLEAAIADAEGRGEVDI
REAKETIARLENDLQLAKQEMAKHVREYQELMNVKLALDIEIATYRKLLEGE
>Lamprey_krt8.10
EKEQIKGLNDRFANFIEKVRFLEQQNKVLEAQWRALQER------GSGG-SNLDS--
LFEAYINGLKQHLDGLGNEKQRLQGELQHMQGAVEDFKAKYEEEINTRTQRENEFVVVKKEVDDAFLNKVELEARLDGLNDEIEFLKKIFAEEL
RQLESQIK--DTSLFVTVDTSR-NLDVDGLIADVRAQYEAMARKSQAEADD-
YKKKISELSSSAGKGDDDVRNSRNEINELNRQMQRIKAEIEALKKQRAQLEAAISEAEGRGEMSIKEAKETIARLEEELHKAKQQMAQHVREYQ
ELMNVKLALDIEIATYRKLLEGE
>Lamprey_krt8.6
EKDQIKGLNDRFANFIDKVRFLEQQNKVLEAQWRALHER------GTVV-SGLDG--
LFDAYTRGLKQQLESLSNNKLGLQNELQAMQGHVEDFKTKYEEEINTRTERENEFVVVKKDVDDAFLNKVELEAKLEALQDEIEFLKKIFAEEL
RQLESQIQ--DTILTVEIDTSR-NLDVDGLIADVRAQYEAIAAKSKAESDA-
YKSKFADLNAMSGKGDDELRQSRSEIADINRQVQRIKAEIEALKKQRAQLEAAIAEAEGRGELAIKEAKESIARLEEELHQAKQDMAKHVREYQ
ELMNVKLALDIEIATYRKLLEGE
>Lizard_HBS1
EKEQIKDLNNQFACFIDKVRYLEERNQALATKWELLKSQ------NPAVKKDLSP--
LCENYITSMRRKLDNLLCEKNQLQQEHKTMLDLIEANRCKYEEEVKRRTNAENEFIVLKQDVDGIFKKQKELEQKKEVLEENIGFLTNFFSKER
AILESQLN--NTSVVVNMDNSR-CLDMDGLIQEIESWYQGIVQKSKGEANL-
YWNQMEDLQSKRSQFYEKLRQNNADIAELNRYVQIMRCQVGTEKQKVASLHAALRETKELGDHALQDAESKYKDLQNRVQTSKDRLVALVRDYQ
DLMNTKLALDIEIATYKTLLEGE
>Frog_krt6b
ETDQIKSLNNKFASVIDKVQLLEQHNCVLRTKWNILQAQ------GIKH-
ENSVPQLPFKDYINALQRQLDQLGNDKLHSDSKLRQIEKIVNDFRRKYEEEVNKHIALKDTFAELKRDADAGYIDKAELEAKIQFLTHEINTLR
VVYEEELTQMQAQTS--DIVVSLSIDNNR-HLDLDSVIALVKAQFEEIISFSHKE-ES-
YQTKYEKLQGQSRTDGDELRKSKVESSELNHMIKRLNSEINILKNQCAKLQMTIAETEQHGEVTLKGAKQQSQDLKAALQKAKQDMARQMMELQ
ELINAKLALDIEIATYRKLLEVE
>Lizard_KRT84L3
TKNELQSLNSKLASFINKVQLLEQHNLMLKTKWDFVQEM------KQHR-SDMEP--
LFDDHTSRLKKELECLEREKKEMQIEHDSSAQTLEKNKSRYEEELNKRATAENGFVLLKKDLDFAFADKAELEAKVEKLAKHISFLKHIYAQEI
SELQNCIS--ETCVMVQLDNRR-ALDMNRTIEEFRRQHEYIASRTRAEAEA-
LQHQYQELKTTAAKNNDNLNAVKEEIQALTRTAHQLESQITSIKTQRCRLEDEVVEAKEHGETAVKDARCKLSDLEEALRKAKQDMTCQLREYQ
SLMNVKMAMNIEIATYRKLIEGE
>Lizard_KRT84L1
EKNELQCLNSKFASFIDKVQFLEQQNLMLKTKWDFLQER------KCCK-SNMEP--
MFKEYIANLKKEQECMECERAQLQAEMKNWREALEVNKKKFEEECHRRTCTENEYVSIKKEVDCVFMDKSEKEAKVQALMKDLFFYKPTFYQEI
HELQSCIS--DTCVTVQMDNSR-GLNMDCVMEVYRRQFEEISSRSRAEAEA-
CRCQYQELKTTAAKHCDNLRHVKEELSEITRVVHRLESEVSNVKAQCTKLEEEVAAAEERGGIAVKDARCKLVELEDALHKAKQDMACQLREYQ
DLMNMKMGLDIEITTYKKLLDGE
>Lizard_KRT84L2
EKNQLQCLNSKFACFIDKVRFLEQHNLMLKTKWDFLKEK------KCQK-SNMDP--
MFHEYISRLRKELERLQWERNQLQVEVNNWRDTMEGNKKKYEEEYNRRASAENEYVTLKKEVDYIFMEKSEKEAKVETLMQDIFFYKTTFEEEI
CEMQSSIS--DTCVTVKMDNSR-DLDVACIIEEFRCRYEDIASRSRAEAEA-
CQCQYQELKTTAAKHSDNLRNFKDELEELTRTVNRLQAEIANVKAQRCKLEEEVAGAEERGEMAVKDAKCKLFDLEDALHKAKQDMACQLREYQ
ELMHLKLALDIEIATYRKLLEGE
```

```
>Human_KRT78
ETQEIRTLNNQFASFIDKVRFLEQQNKVLETKWHLLQQQ-GLS--GSQ--QGLEP--
VFEACLDQLRKQLEQLQGERGALDAELKACRDQEEEYKSKYEEEAHRRATLENDFVVLKKDVDGVFLSKMELEGKLEALREYLYFLKHLNEEEL
GQLQTQAS--DTSVVLSMDNNR-YLDFSSIITEVRARYEEIARSSKAEAEA-
YQTKYQELQVSAQLHGDRMQETKVQISQLHQEIQRLQSQTENLKKQNASLQAAITDAEQRGELALKDAQAKVDELEAALRMAKQNLARLLCEYQ
ELTSTKLSLDVEIATYRRLLEGE
>Frog_krt78.1
EREKIKLLNNKFASFIDKVRFLEQQNKVLETKWSLLQKQ-QVS--KSRS-SEIES--
IFNTLITSLRSQLDSLVKDKGLLGGELQVMKDHAENFKNKFELEFKKRTADENDFVSLKKDVDATYLIQVQLETKQKALDDELAFLRNLYKEEL
DGIKQQTA--GTSVVLSMDNSR-MLDLGDIIADVKAQYEDTTRRSKEEAEA-
YQSKYQQLQLAAGQQGEDLKSSKKEISEVNRSVQKLRNEIESVKKQIASLQVSIQDSERRGDIALRDAQEKLVNLERALQKAKQEMAQQLREYQ
TLLNVKLALDVEIATYRKLLEGE
>Frog_krt78.3
ERDQIKGLNNKFAGFIDKVRFLEQQNQILGTKWNFLQEK-SQKLCSRR--DTIKP--
LFDAYITNLQRQLEGLRSEKCRLDGELKNMQDVVEEFKSKYEEEINKRTCTENEFVALKKDVDILYMKKAELEAKEESLGNEINFLRNIFDAEL
AELQDCLS--DINVVLSMDNNR-DLDLNGLIAECKAQYEEIAARSKTEAEA-
YSKKFQQLKEAAGQHGDNLRSSKTEIQDLNTMIKRLQSEIECVKKQIAGLQAAICDAESRGEATLKDANCKMSELEAVLQKAKEDLAIQLREYQ
ELLRVKLALDVEIATYRTLLEGE
>Frog_krt78.4
ERDQIKCLNNKFASFIDKVRFLEQQNQILGTKWNFLQDK-SQKLCSRR--DTIKP--
LFDAYITNLQRQLEGLRSEKCRLDGELKNMQDVVEEFKCKYEEEINKRTCAENQFVGLKKDVDVLYMKKAELEAKEETLANEISFLRSLFDAEL
AELQDCLS--DINVVLCMDNNR-ELDLNGLIAECKAQYEEIAARSKTEAEA-
YARKFQQLKETAGLHGDNLKSSKTEIQDLNTMIKRLQSEIECVRKQIAGLQAAICDAESRGEATLKDARCKMSELEAVLQKAKEDLAIQLKEYQ
ELLRVKLALDVEIATYRTLLEGE
>Frog_krt78.6
EREQIKTLNNKFAAYIDKVRFLEQQNKVLETKWKLLQEQ-GTKHSTKR--ANLEP--
LFEKYIGDLRRYLDTLINERGRLEHELKNQQDLVEEYKKKYEDEINKRTKAENDFVLLKKDVDAAFMAKTDLEARVDGLTSEINFLRTLYAAEL
SQVHEQVT--DTSVILTMDNNR-DLNLSGIIQEVKCQYEQIAQRSKMEAEA-
YDNKYKELQKTVEGHGHSIKNSKAEIAELNRNIQRLRAEIENLKKQIAGLNQSIAGAEEKGNLALKDAEKKLHDLEAAEKKLKEDMARQLKEYQ
ELLAAKISLDVEISTYGLMLGGE
>Frog_krt78.5
EGEQIKTLNNKFAAYIDKVRFLEQNNKVLETKWKLLQEQ-GTKHSTKR--ANLDP--
LFEKYIGDLKRYSNTLINERGHLVHERKNQQGFAEEYKKKYEDEINKRTKAENDFVVLKKDVDTAYMAKTELEAKVDAVTSEINFLRTLYAAEL
SQVHDSVT--NTSVVLTMDNNR-DLNLEGIIQDVKAQLELCAQRSKMEAEA-
FDNTYKELQKTAEGHGDSIKNSKAEIAELNRRIQRLKAEIENLKKQIAGLNQSIAGAEEKGNLALKDAEKKLQDLEAAEKKLKEDMARQLKDYQ
ELLAAKIALDVEIGTYGLMLGGE
>Frog_krt78.7
EREQIKTLNNKFAAYIDKVRFLEQQNKVLETKWKLLQEQ-GTKGSTKR--ASLEP--
LFEKYIGDLRRYLDTLTNEKARLLQELKNLQDLVEEYKKKYEDEINKRTKAENDFVVLKKDVDAAYMIKTELEAKVDAVTSEINFLRTLFAAEL
SQVHDSVT--DTSVVLTMDNNR-DFNLEGIIQDVKAQLELAAQRSKMEAEA-
YDNKYKELQRTAEGHGDSIKNSKTEIAELNRRIQRLRAEIENIKKQIAGLNQSIAGAEEKGNLALKDAEKKLQDLEAAEKKLKEDMARQLKEYQ
ELLAAKISLDVEISTYGLMLGGE
>Frog_krt78.9
EREQIKTLNNKFAAYIDKVRFLEQQNKVLETKWKLLQEQ-GTKGSTKR--ANLEP--
LFEKYIGDLNKYLSTLINEKDRLTQELKNLQVLVEDYKKKYEDEITKRTKAENDFVLLKKDVDTVYMAKTELEAKVDAVTSEINFLRTLYAAEL
SQVHDTVT--DTSVVLTMDNNR-DLNLEGIIKDVKAQLELCAQRSKMEAEA-
FDNKYKELQKTAEGHGDSIKNSKSEIAELNRRIQRLKAEIENIKKQIAGLNQSIAGAEEKGNLALKDAEKKLQDLEAAEKKLKEDMARQLKEYQ
ELLAAKIALDVEIGTYGLMLGGE
>Frog_krt78.10
EREQIKTLNNKFAAYIDKVRFLEQQNKVLETKWKLLQEQ-GTKGSTKR--ANLEP--
LFEKYIGDLNKYLSTLITKKDRLNQELKNLQDLVEDYKKKYEDEINKRTKAENDFVVLKKDVDAAYMVKTELEAKVDAVTSEINFLRTLYAAEL
SQVHDSVT--DTFVVLTMDNNR-DLNLEGIIQDVKAQLELCAQRSKMEAEA-
FDNKYKELQKTAEGHGDSIKNSKSEIAELNRRIQRLKAEIESTKKQIAGLNQSIAGAEEKGNLALKDAEKKLQDLEAAEKKLKEDMARQLKEYQ
ELLAAKISLDVEISTYGLMLGGE
>Frog_krt78.8
EREQIKTLNNKFAAYIDKVRFLEQQNKVLETKWKLLQEQ-GTKGSTKR--ASLEP--
LFEKYIGDLNKYLSTLINEKDRLNQELKNLQVLVEDYKKKYEDEINKRTKAENDFVLLKKDVDAAYMIKTELEAKVDAVTSEINFLRTLYAAEL
SQVHDSVT--DTSVVLTMDNNR-DLNLEGIIQEVKAQLELCAQRSKMEAEA-
FDNKYKELQKTAEGHGDSIKNSKSEIAELNRRIQRLKAEIESTKKQIAGLNQSIAGAEEKGNLALKDAEKKLQDLEAAEKKLKEDMARQLKEYQ
ELLAAKIALDVEIGTYGLMLGGE
>Lizard_KRT78L3
EREQIKCLNDKFASFIDKVRFLEQQNKILETKLNLLQQQ-PMP--HLK--RKADH--
TFEAYIHGLRKQLESLSTERGRLGSEWKNMQDLVEDFKRRYEEEINKRTTAENEFVLLKKDVDLAYMNKMELKTRTASLDKEVDFLRSLYEEEQ
AQMQSQVT--DTSIILSMDNSR-DLDMDVIIAEVKAQYEDMANRSRAEVDA-
YQSKFQELQLTAGKHDDALKNSKTEILELNRLIHRLRAETGTVKKQCAFLQSSIAEAEERGETALKDAREKLTKLENATQKSNEELVRLLRDYQ
ELLNVKISLDIEIATYKTLLEGE
>Lizard_KRT78L2
EKEQIKLLNNQFANFIDKVRFLEQQNKVLQTKWQILQQQ-TQG--RGPM-QNLDG--
EYQLFLNGLRNQIDQIRSQKQQLMSELQNMQQVVEDSKNKYEQEINQRTTSENEFVVLKKDVDSAYMTKVDLDAKVGGIVEQINFFRRIFDIEL
SQIPIGGK--DTSVVVNMDDSR-DLNLEGMIDEVRAQYEEVARSSRAEAET-
YRSQYEALQNAAGRHGENLQNTRQEIQELTRNIQRLRTEIEHAKKQAAQMQSAIAEAEERGEMALRDAKQKLDELEGSLTKDKEVLARLLKEYQ
DLLNVKLALDMEIAMYRKLLEGE
>Human_KRT82
EKEQIKCLNNRFASFINKVRFLEQKNKLLETKWNFMQQQ------RCCQ-TNIEP--
IFEGYISALRRQLDCVSGDRVRLESELCSLQAALEGYKKKYEEELSLRPCVENEFVALKKDVDTAFLMKADLETNAEALVQEIDFLKSLYEEEI
CLLQSQIS--ETSVIVKMDNSR-ELDVDGIIAEIKAQYDDIASRSKAEAEA-
YQCRYEELRVTAGNHCDNLRNRKNEILEMNKLIQRLQQETENVKAQRCKLEGAIAEAEQQGEAALNDAKCKLAGLEEALQKAKQDMACLLKEYQ
EVMNSKLGLDIEIATYRRLLEGE
```

```
>Alligator_KRT84L1
EKEQIKTLNNKFASFIDKVRFLEQQNKVLETKWSFLQNQ------QCRR-NTIMP--
TLDAYICNLRKQLEALGCEGAQLEADLKAAHEVMETNKKMYEEECSRRTCAENEFVVLKKEVDCVFLNKAELEARVESLKDEIIFLKSLYEEEI
HMLHSHIS--DTSVIVQMDNSR-NLDLDGIITEVKAQYEDIASRSRAEAEA-
YESKFEELRVTAGRHADNLRDTKNEIAELTRIIQRLRGEVGTAKDQRCKLEAAVAEAEQQGELAIKDARCKLTDLETALQQAKADLAHQLREYQ
ELMNVKLALDIEIATYRKLLEGE
>Alligator_KRT84L2
EKEQIKTLNNKFASFIDKVRFLEQQNKVLETKWRFLQEQ------KRYK-SNIEP--
MFEAYIYNLRRQLEVLAGDRAKLETELSNMQETLEDFKEKYEEETSRRACAENEFVMLKKDVDCAYMNKAELEAKVESLIEEITFLKNLHEEEI
AQLQASIS--DTSVIVQMDNSR-ALDMDDIIAEVKAQYEDIANRSRADAES-
YYSKYEELRETAGKHSDKLRNTKNEIMELSRVIQRLNGELENAKAQRGKLEAAIAEAEERGEMALKDAKCKLTDLEAALQKAKQDMACQLREYQ
ELMNVKLALDIEIATYRKLLEGE
>Lizard_KRT84L4
EKEQLKTLNNKFASFIDKVRFLEQQNKVLETKWSLLQEQ------KRVK-SNIEP--
LFDGLIHKLKKQLESLGCDRNKLESDLTSSRDILEDYRKRYEDECNRRTSAENEFVTLKKDVDCIYMHKADLEGKVESLVEEVNFLRCLHDMEL
QELHSCIS--DTSVIVQMDNSR-ELDLDGIIAEVKAQYEDIANKSRYEAES-
YQCKYEEMRATAGKHNDHLRDTKNEIMELNRVIQRLKAEIDTAKGQRSKLEAAVAEAEERGEMALKDAKCKLTELEDALIKAKADMARQLREYQ
ELMNVKLALDIEIATYRKMLEGE
>Human_KRT84
EKEQIKTLNNKFASFIDKVRFLEQQNKLLETKWSFLQEQ------KCIR-SNLEP--
LFESYITNLRRQLEVLVSDQARLQAERNHLQDVLEGFKKKYEEEVVCRANAENEFVALKKDVDAAFMNKSDLEANVDTLTQEIDFLKTLYMEEI
QLLQSHIS--ETSVIVKMDNSR-DLNLDGIIAEVKAQYEEVARRSRADAEA-
YQTKYEEMQVTAGQHCDNLRNIRNEINELTRLIQRLKAEIEHAKAQRAKLEAAVAEAEQQGEATLSDAKCKLADLECALQQAKQDMARQLCEYQ
ELMNAKLGLDIEIATYRRLLEGE
>Human_KRT85
EKEQIKSLNSRFAAFIDKVRFLEQQNKLLETKWQFYQNQ------RCCE-SNLEP--
LFSGYIETLRREAECVEADSGRLASELNHVQEVLEGYKKKYEEEVALRATAENEFVVLKKDVDCAYLRKSDLEANVEALVEESSFLRRLYEEEI
RVLQAHIS--DTSVIVKMDNSR-DLNMDCIIAEIKAQYDDVASRSRAEAES-
YRSKCEEMKATVIRHGETLRRTKEEINELNRMIQRLTAEIENAKCQRAKLEAAVAEAEQQGEAALSDARCKLAELEGALQKAKQDMACLLKEYQ
EVMNSKLGLDIEIATYRRLLEGE
>Human_KRT83
EKEQIKSLNSRFAAFIDKVRFLEQQNKLLETKLQFYQNR------ECCQ-SNLEP--
LFAGYIETLRREAECVEADSGRLASELNHVQEVLEGYKKKYEEEVALRATAENEFVALKKDVDCAYLRKSDLEANVEALIQEIDFLRRLYEEEI
RILQSHIS--DTSVVVKLDNSR-DLNMDCIVAEIKAQYDDIATRSRAEAES-
YRSKCEEMKATVIRHGETLRRTKEEINELNRMIQRLTAEVENAKCQNSKLEAAVAQSEQQGEAALSDARCKLAELEGALQKAKQDMACLIREYQ
EVMNSKLGLDIEIATYRRLLEGE
>Human_KRT81
EKEQIKSLNSRFAAFIDKVRFLEQQNKLLETKLQFYQNR------ECCQ-SNLEP--
LFEGYIETLRREAECVEADSGRLASELNHVQEVLEGYKKKYEEEVSLRATAENEFVALKKDVDCAYLRKSDLEANVEALIQEIDFLRRLYEEEI
LILQSHIS--DTSVVVKLDNSR-DLNMDCIIAEIKAQYDDIVTRSRAEAES-
YRSKCEEMKATVIRHGETLRRTKEEINELNRMIQRLTAEVENAKCQNSKLEAAVAQSEQQGEAALSDARCKLAELEGALQKAKQDMACLIREYQ
EVMNSKLGLDIEIATYRRLLEGE
>Human_KRT86
EKEQIKSLNSRFAAFIDKVRFLEQQNKLLETKLQFYQNR------ECCQ-SNLEP--
LFEGYIETLRREAECVEADSGRLASELNHVQEVLEGYKKKYEEEVSLRATAENEFVALKKDVDCAYLRKSDLEANVEALIQEIDFLRRLYEEEI
RVLQSHIS--DTSVVVKLDNSR-DLNMDCIIAEIKAQYDDIVTRSRAEAES-
YRSKCEEMKATVIRHGETLRRTKEEINELNRMIQRLTAEVENAKCQNSKLEAAVAQSEQQGEAALSDARCKLAELEGALQKAKQDMACLIREYQ
EVMNSKLGLDIEIATYRRLLEGE
>Human_KRT77
EREQIMVLNNKFASFIDKVRFLEQQNQVLQTKWELLQQV-NTS--TGT--NNLEP--
LLENYIGDLRRQVDLLSAEQMRQNAEVRSMQDVVEDYKSKYEDEINKRTGSENDFVVLKKDVDAAYVSKVDLESRVDTLTGEVNFLKYLFLTEL
SQVQTHIS--DTNVILSMDNNR-SLDLDSIIDAVRTQYELIAQRSKDEAEA-
YQTKYQELQITAGRHGDDLKNSKMEIAELNRTVQRLQAEISNVKKQIEQMQSLISDAEERGEQALQDAWQKLQDLEEALQQSKEELARLLRDYQ
AMLGVKLSLDVEIATYRQLLEGE
>Lizard_KRT78L5
EREQIKTLNNKFATFIDKVRFLEQQNKVLETKWDLLYQQ-GTP--ALR--DNLEP--
LYEAYIDTLRRQFESLTNDKNHLDIELKNTQELVEDFKSKYEDEINKRTACENDFVVLKKDVDGSYMHKIDLGSKADSLTQHIQFLRALFETEL
SQVQSQVR--DTNVVLQMDNNR-DLDLNGIIAEVRAQYEDIANRSRAEAET-
YQSKYQELQTTAQKHCDELKTTKDDIAEMSRVVHKLRAEIEIVKKAVNGLQSSIAESEQRGEIALKDAQKKLLDLQSALTSSKDELARLLRDYQ
ELMNVKLALDVEIATYRTLLEGE
>Alligator_KRT78L2
EKEQIKTLNNKFATFIDKVRFLEQQNKVLSTKWELLQQQ-GPS--GPR--RDLTA--
LYENYIENLRRQLDNVLSQRGPLESQLHDMQGYVEDYKNKYEEEINRRTSAENEFVVLKKDVDCAYMTKVDLEAKVQALIDEINFLRYVFEEEL
SQLQTISR--DLSVVVSMDNSR-HLNLDSIIDEIRAQYEQIAQSSRAEAEA-
YQSRYEELQSTAGQHGESLRNTRQEIQELTRCIQKLRSEIENVRKQCEQLQAAIAEAEERGEMALKDARCKLEELECALHKDKEELARLLKEYQ
ELLNVKIALDIEIAMYRKLLEGE
>Alligator_KRT78L3
EKEQIKTLNNKFATFIDKVRFLEQQNKVLSTKWELLQQQ-GHS--GPR--KDLSS--
LFENYIQNLRRQLDSIHGQRGQLESELQNMQNYVEDYKNKYEDEINRRTAAENEFVVLKKDVDCAYMTKVDLEAKVQALIDEINFLRYVYEEEL
SQMQTISR--DLSVVVSMDNSR-HLDMDSMIDEVRRQYEDIARSSRAEAEA-
YQSRYEELQTTAGRHGDNLRNTKQEIQELTRCIQRLRSEIENVKKQ------------------------------------------------------
-----------------------
>Alligator_KRT78L5
EKEQIKTLNNQFATFIDKVRFLEQQNKVLSTKWELLQQQ-GHS--GPR--KDLSS--
LFESYIQNLKRQLDSILHQREQLESDLQNMQPHVEEYKKKYEEEINRRATAENEFVVLKKDVDNAYMNKVQLEAKVQALMDEIDFLRHVFEEEL
SQMHTISR--DLSVVVSMDNTR-HLDMDSIIDEVRRQYEDIARSSRAEAEE-
YQSRYEELQNTAGRHGDNLRNTKQEIQELTRCIQRLRSEIENVKKQVRALQAAIAEAEDRGEMALKDARSKLQELEAALQKDKEELARLLKEYQ
ELLNIKIALDIEIAMYRKLLEGE
```

```
>Alligator_KRT78L4
EKEQIKTLNNQFATFIDKVRFLEQQNKVLSTKWELLQQQ-GHS--GPS--KDLSS--
LFEIYIQNLKRQLDSILDERGQLVSELQKMQGHLEDYKTKYEEEINRRTTAENEFVVLKKDVDNAYMSKVELEAKVQALTDEINFLRYVYEEEM
SQMHTISR--DLSVVVSMDNTR-HLDMDSIIDEVRRQYEDIARSSRAEAEE-
YQSRYEELQNTAGRHGDNLRNTKQEIQELTRCIQRLRSEIENVKKQHQQLQAAIAEAENRGEMALKDARSKLQELEAALQKDKEELARLLKEYQ
ELLNIKIALDIEIAMYRKLLEGE
>Human_KRT74
EREQIKVLNDKFASFIDKVRFLEQQNQVLETKWELLQQL-DLN--NCK--KNLEP--
ILEGYISNLRKQLETLSGDRVRLDSELRSMRDLVEDYKKRYEVEINRRTTAENEFVVLKKDADAAYAVKVELQAKVDSLDKEIKFLKCLYDAEI
AQIQTHAS--ETSVILSMDNNR-DLDLDSIIAEVRMHYEEIALKSKAEAEA-
YQTKIQELQLAASRHGDDLKHTRSEMVELNRLIQRIRCEIGNVKKQRASLETAIADAEQRGDNALKDAQAKLDELEGALHQAKEELARMLREYQ
ELMSLKLALDMEIATYRKLLEGE
>Human_KRT72
EREQIKALNNKFASFIDKVRFLEQQNQVLETKWNLLQQL-DLN--NCR--KNLEP--
IYEGYISNLQKQLEMLSGDGVRLDSELRNMQDLVEDYKKRYEVEINRRTAAENEFVVLKKDVDAAYMNKVELQAKVDSLTDEIKFFKCLYEGEI
TQIQSHIS--DTSIVLSMDNNR-DLDLDSIIAEVRAQYEEIALKSKAEAET-
YQTKIQELQVTAGQHGDDLKLTKAEISELNRLIQRIRSEIGNVKKQCADLETAIADAEQRGDCALKDARAKLDELEGALHQAKEELARMLREYQ
ELVSLKLALDMEIATYRKLLESE
>Human_KRT71
EREQIKALNNKFASFIDKVRFLEQQNQVLETKWELLQQL-DLN--NCK--NNLEP--
ILEGYISNLRKQLETLSGDRVRLDSELRNVRDVVEDYKKRYEEEINKRTAAENEFVLLKKDVDAAYANKVELQAKVESMDQEIKFFRCLFEAEI
TQIQSHIS--DMSVILSMDNNR-NLDLDSIIDEVRTQYEEIALKSKAEAEA-
YQTKFQELQLAAGRHGDDLKNTKNEISELTRLIQRIRSEIENVKKQASNLETAIADAEQRGDNALKDARAKLDELEGALHQAKEELARMLREYQ
ELMSLKLALDMEIATYRKLLESE
>Human_KRT73
EREQIKVLNNKFASFIDKVRFLEQQNQVLETKWELLQQL-DLN--NCK--NNLEP--
ILEGYISNLRKQLETLSGDRVRLDSELRSVREVVEDYKKRYEEEINKRTTAENEFVVLKKDVDAAYTSKVELQAKVDALDGEIKFFKCLYEGET
AQIQSHIS--DTSIILSMDNNR-NLDLDSIIAEVRAQYEEIARKSKAEAEA-
YQTKFQELQLAAGRHGDDLKHTKNEISELTRLIQRLRSEIESVKKQCANLETAIADAEQRGDCALKDARAKLDELEGALQQAKEELARMLREYQ
ELLSVKLSLDIEIATYRKLLEGE
>Lizard_KRT78L1
EKEQMKTLNNKFASFIDKVRFLEQQNKVLETKYKLLQEQ----GPGAIQ-KHLDP--
MYEAYINNLRRQLECLQNEKRGLDSELKNFQDMVEDYKTKYEEEINKRTQAENDFVILKKDVDAAYMNKVELQAKCDALIDEINFMKCLYEAEL
SQMQQTVS--DTSVILQMDNNR-CLDMDSIIAEVKARYEEIAQMGRIDAES-
YQSKYEELQASVGKHGDNLQKTKAEISEHNRMIQRIRAEIDNVKKQCEKLKSSIAEAEERGELALKDARNKLTELEEALQKAKEEMTRLLKEYQ
ELMSCKLALDVEIATYKKLLEGE
>Human_KRT4
EREQIKLLNNKFASFIDKVQFLEQQNKVLETKWNLLQQQ-----TTTTSSKNLEP--
LFETYLSVLRKQLDTLGNDKGRLQSELKTMQDSVEDFKTKYEEEINKRTAAENDFVVLKKDVDAAYLNKVELEAKVDSLNDEINFLKVLYDAEL
SQMQTHVS--DTSVVLSMDNNR-NLDLDSIIAEVRAQYEEIAQRSKAEAEA-
YQTKVQQLQISVDQHGDNLKNTKSEIAELNRMIQRLRAEIENIKKQCQTLQVSVADAEQRGENALKDAHSKRVELEAALQQAKEELARMLREYQ
ELMSVKLALDIEIATYRKLLEGE
>Alligator_KRT78L1
EREQMKTLNDKFASFIDKVRFLEQQNKVLETKWKLLQEQ-GQGTGPHS--RSLDQ--
LYEVYINSLRKQLDGLTSEKSQLQLELKNCEDLVEDFKTKYDEEINKRTAAENDFVLLKKDVDAAYMNKVELQAKLDSLADELNFLRCVYDAEL
SQMQQTVS--DTSVVLSMDNNR-NLDLDSIIAEVKAQYEEIAQKSRAEAEA-
YQCKYQELQVTAGKHGDSLKDTKAEISEMNRMIQRLRAEIENVKKQCENLQTSVADAEQRGELALKDARVKLSELEAALQKAKTELTRQLRDYQ
ELMNVKLALDIEIATYRKLLEGE
>Frog_krt78.2
EREQIKTLNNKFASFIDKTRFLEQQNQVLKTKWELLQEH-GQKGVTKR--SNIVP--
LFEAYISNLRRSLDSLLNDKHRLESDLRSTQDLVEDYKNKYEDEINKRTTAENEFVVLKKDVDGAYMNKVELDCKVDALTDEINFLRTLYEAEV
QDLQGQLS--DISVVLSMDNNR-ALDLDGIIAEVKCQYEEIANKSKAEAEA-
CAMKFQQLQATAGQHGDSLKSSKTEIADLNRMIQRLKAEIESVKKQCANLQTSITEAEERGELAVKDAQKKLDELQEALRKAKEEMAKQLHDYQ
RLMSIKLALDIEIATYKKLLEGE
>Human_KRT2
EREQIKTLNNKFASFIDKVRFLEQQNQVLQTKWELLQQM-NV---GTRP-INLEP--
IFQGYIDSLKRYLDGLTAERTSQNSELNNMQDLVEDYKKKYEDEINKRTAAENDFVTLKKDVDNAYMIKVELQSKVDLLNQEIEFLKVLYDAEI
SQIHQSVT--DTNVILSMDNSR-NLDLDSIIAEVKAQYEEIAQRSKEEAEA-
YHSKYEELQVTVGRHGDSLKEIKIEISELNRVIQRLQGEIAHVKKQCKNVQDAIADAEQRGEHALKDARNKLNDLEEALQQAKEDLARLLRDYQ
ELMNVKLALDVEIATYRKLLEGE
>Lizard_KRT78L4
EREQIKTLNNKFASFIDKVRFLEQQNKVLETKLNLLQQQ-GST--ATT--IHIDP--
MFEAYINNLRNQLDGLVGDRGRLDGELRNTQDLVEDYRKRYEDEINKRTTAENEFVDIKKNVDGGFMHKIELGAKADGLTDEINFLRALHEAEL
SQIQGRIS--DTNIVLQMDNNR-DIDLNSIISEVKAQYEDIANRSRAEAEA-
YQSKFQELQSTAVNHGDDLKNTKNEIAELSRIIQRMKAEIDNVKKQIAGLQTAIADAEQRGELALKDARVKLTDLQHALQSAKDELARLLRDYQ
ELMNVKLALDVEIATYRTLLEGE
>Alligator-K78L6
EREQIKTLNNKFASFIDKVRFLEQQNKVLETKWDLLQGT------TTTR-VSLEP--
MFEAYINNLQCQLENLLGNRGQLEGELRNLQDLVEDFKKKYEEEINRRTAIENEFVMSKKDADHSFMNKVELQVKVDSLIDEISFLRAFFKAEL
DQMQGQIT--DTNVILQMDNNR-DLDLNSIISEVKAQYEDIANRSRAEAEA-
YQSKFQELQTTAMTHGDDLKNTRNEISELTRIIQRLKAEIDNVKKQIAGLQTAIADAEQRGEMALKDARAKLSDLQNALQTAKDELAHLLRDYQ
ELMNVKLALDIEIATYRTLLEGE
>Human_KRT1
EREQIKSLNNQFASFIDKVRFLEQQNQVLQTKWELLQQV-DT---STRT-HNLEP--
YFESFINNLRRRVDQLKSDQSRLDSELKNMQDMVEDYRNKYEDEINKRTNAENEFVTIKKDVDGAYMTKVDLQAKLDNLQQEIDFLTALYQAEL
SQMQTQIS--ETNVILSMDNNR-SLDLDSIIAEVKAQYEDIAQKSKAEAES-
YQSKYEELQITAGRHGDSVRNSKIEISELNRVIQRLRSEIDNVKKQISNLQQSISDAEQRGENALKDAKNKLNDLEDALQQAKEDLARLLRDYQ
ELMNTKLALDLEIATYRTLLEGE
```

```
>Human_KRT79
EREQIKTLNNKFASFIDKVRFLEQQNKVLETKWALLQEQ-GQNLGVTR--NNLEP--
LFEAYLGSMRSTLDRLQSERGRLDSELRNVQDLVEDFKNKYEDEINKHTAAENEFVVLKKDVDAAYMGRMDLHGKVGTLTQEIDFLQQLYEMEL
SQVQTHVS--NTNVVLSMDNNR-NLDLDSIIAEVKAQYELIAQRSRAEAEA-
YQTKYEELQVTAGKHGDNLRDTKNEIAELTRTIQRLQGEADAAKKQCQQLQTAIAEAEQRGELALKDAQKKLGDLDVALHQAKEDLTRLLRDYQ
ELMNVKLALDVEIATYRKLLESE
>Frog_krt59
EKEQIKTLNNKFASFIDKVRFLEQQNKMLETKWAILQEQ------RTAR-SHIEP--
LFEAYIGNLRRQLDCLLGEKSRLEGERKNMEDIVEDFKRKYEEEVNRRTTAENEFVVLKKDVDAAFLHKAELQTRVESQTDEVNFLRTMFEAEI
AQLQGQIS--DTSVIVSMDNNR-NLDLDGIIAEVKAQYEEIANRSRAEAES-
YQSKYEELRLTAGRNGDDLRNTKNEVADLNRMINRLRGEIETVNAQRGKLEGAITEAEERGEMATKDAKKKLADLEEGLQKAKQDMARQLREYQ
ELMNVKLALDIEIATYRKLLEGE
>Alligator_KRT5L1
EKEQIKTLNNKFASFIDKVRFLEQQNKVLETKWALLNDQ-GQKSSLSK--NNMDP--
LFEAYISNLKKQLNSLLNERGRLDGELKNMQDLVEDFKNKYEDEINRRTTAENEFVVLKKDVDAAYMNKVELEAKVDALMDELNFLRALYDAEL
AQLSAQMS--DTSVILSMDNNR-DLDLSSIITEVKAQYEDIANRSRAEAEA-
YQTKFEELQATAGKHGDDLRNTKGEISELTRLIQRIRAEIENARNQCANLQTAIADSEERGELALKDAKLKLAELEDALQKAKADMTRQLREYQ
ELMNVKLALDIEIATYRKLLEGE
>Lizard_KRT5L1
EKEQIKTLNNKFASFIDKVRFLEQQNKVLETKWTLLQDQ-GFKTGANK--GNLDP--
LFEAYINNLRKQLNGLINEKGRLDGELRNMQDIVEDFKTKYEEEINRRTSAENEFVMLKKDVDAAYMNKVELEAKVDSLTDELNFLRALYEAEL
AQLQAQMS--DTAVILSMDNNR-DLDLNGIVSEVKAQYEDIANRSRAEAEA-
YQTKFEELQATAGRHGDDLRNTKGEISELNRLIQRIRAEIENARNQCANMQTAIADAEDRGELALKDARAKLIELEEALQKAKEEMTRLLREYQ
ELMNVKLALDIEIATYRKLLEGE
>Alligator_KRT5L3
EKEQIQTLNNKFASFIDKVRFLEQQNKVLETKWMLLQEQ-GQK--TVR--NNIEP--
LFETYINNLRIQLNGLVGDRGRLEGELDNTQNLLEDFKNRYEEETNRRHDAENDFVTLKKDVDGAFMNKVELQAKVDSLIDEINFLRAIYEAEL
SQMQTQIT--DTSVVLSMDNNR-SLDMDSIIAEVKAQYEDIANRSRVEAES-
YQVKYEELQATAGRHGDDLRNTKHEISEINRHIQRLRADIESVKRQCASLQAAIAEAEERGEMALKDARDKLAQLEDALQQAKADLARQLREYQ
ELMNVKLALDIEIATYRKLLEGE
>Lizard_KRT5L3
EREQIKTLNNKFASFIDKVRFLEQQNKVLETKLTLLQEQ-GQK--TCK--SNIEP--
LFEMYINDLRKKLNNLENDRGRLLGELNNIQNLVEDCKNKYEDEINKRATAENDFVTLKKDVDAAYLNKVELESKADSLNCEVNFLRALYEEEL
AQMHTQIS--DTSVVLSMDNNR-NLDMDSIIAEVKAQYEDIANKSRAESET-
YQCKYEELQTAAGRHGDDLRNTKQEISEINRHIHRLRAEIDNVKKQCANLQAAIAEAEERGELALKDARQKLAELEDALQKAKQDMARQLKEYQ
DLMNVKLALDIEIATYRKLLEGE
>Frog_krt61
EREQIKTLNNKFASFIDKVRFLEQQNKVLETKWNLLQEQ-GVK--SVK--CNIDP--
LFEAYISALRRQLDSLLSEKVRLDSDLRQMQDAVEDYKKKYEDEINKRTSVENEFVVLKKDVDAAYMNKVELEAKLNALTDEINFLRAVYEAEI
AQMQTQIS--DTSVVLSMDNNR-TLDLNGIIAEVKAQYEDIANRSRAEAES-
YQTKYEELQQSAGRHGDDLRSTKTEISELNRMINRLRSEIDSVKKQCAKLQAAITEAEERGELALKDAKKKLADLEEALQKCKQEMARQLREYQ
ELMNVKLSLDVEIATYRKLLEGE
>Lizard_KRT5L2
EREQIKTLNNKFASFIDKVRFLEQQNKVLETKWDLLQQQ-GTT--CQQ--STMEP--
LFDVYITNLRRQLEALTNERGRLDSELRNMQDMVEDYKNKYEDEINKRTGSENEFVVLKKDVDGAYMNKIELAGKFESLNDEIEFLKAMYDAEL
SQMQQHVS--DTSVVLSMDNNR-CLDLNSIIAEVKAQYEDIAQRSRTEAES-
YQTKYEELQVSAGRHGDDLRNTKTEISELNRMIQRLRSEIDSFKKQCANLQAAIAEAEDRGEMALKDAKHKLSELEEALQRTKQDLARQLKEYQ
ELMNVKLALDIEIATYKKLLEGE
>Human_KRT3
EREQIKTLNNKFASFIDKVRFLEQQNKVLETKWNLLQQQ-GTSSISGT--NNLEP--
LFENHINYLRSYLDNILGERGRLDSELKNMEDLVEDFKKKYEDEINKRTAAENEFVTLKKDVDSAYMNKVELQAKVDALIDEIDFLRTLYDAEL
SQMQSHIS--DTSVVLSMDNNR-SLDLDSIIAEVRAQYEDIAQRSKAEAEA-
YQTKLGELQTTAGRHGDDLRNTKSEIIELNRMIQRLRAEIEGVKKQNANLQTAIAEAEQHGEMALKDANAKLQELQAALQQAKDDLARLLRDYQ
ELMNVKLALDVEIATYRKLLEGE
>Human_KRT76
EREQIKTLNNKFASFIDKVRFLEQQNKVLETKWELLQQQ-TTG--SGP--SSLEP--
CFESYISFLCKQLDSLLGERGNLEGELKSMQDLVEDFKKKYEDEINKRTAAENEFVGLKKDVDAAFMNKVELQAKVDSLTDEVSFLRTLYEMEL
SQMQSHAS--DTSVVLSMDNNR-CLDLGSIIAEVRAQYEEIAQRSKSEAEA-
YQTKLGELQTTAGRHGDDLRNTKSEIMELNRMIQRLRAEIENVKKQNANLQTAIAEAEQRGEMALKDANAKLQDLQTALQKAKDDLARLLRDYQ
ELMNVKLALDVEIATYRKLLEGE
>Alligator_KRT5L2
EREQIKTLNNKFASFIDKVRFLEQQNKVLETKWTLLQEQ-GQT--VTR--KTLEP--
LFEAYINNLRRQLDSLLGDGPRLDSELRNMQDLVEDFKNKYEDEINRRTAAENEFVMLKKDVDNAYMNKVELQAKADSLADEINFLRALYDAEL
SQVHQQVS--DTSVILSMDNNR-SLNLDSIISDVKAQYEDIANRSRAEAES-
YQSKYEELQVSAGRHGEDLRSTKMEITEINRMIQRLRNEIDNVKKQCANLQAAIAEAEERGEMALKDARAKLADLEEALQKAKADLARQLREYQ
ELMNVKLALDIEIATYRKLLEGE
>Human_KRT75
EREQIKTLNNKFASFIDKVRFLEQQNKVLETKWALLQEQ-GSR--TVR--QNLEP--
LFDSYTSELRRQLESITTERGRLEAELRNMQDVVEDFKVRYEDEINKRTAAENEFVALKKDVDAAYMNKVELEAKVKSLPEEINFIHSVFDAEL
SQLQTQVG--DTSVVLSMDNNR-NLDLDSIIAEVKAQYEDIANRSRAEAES-
YQTKYEELQVTAGRHGDDLRNTKQEISEMNRMIQRLRAEIDSVKKQCSSLQTAIADAEQRGELALKDARAKLVDLEEALQKAKQDMARLLREYQ
ELMNIKLALDVEIATYRKLLEGE
>Human_KRT5
EREQIKTLNNKFASFIDKVRFLEQQNKVLDTKWTLLQEQ-GTK--TVR--QNLEP--
LFEQYINNLRRQLDSIVGERGRLDSELRNMQDLVEDFKNKYEDEINKRTTAENEFVMLKKDVDAAYMNKVELEAKVDALMDEINFMKMFFDAEL
SQMQTHVS--DTSVVLSMDNNR-NLDLDSIIAEVKAQYEEIANRSRTEAES-
YQTKYEELQQTAGRHGDDLRNTKHEISEMNRMIQRLRAEIDNVKKQCANLQNAIADAEQRGELALKDARNKLAELEEALQKAKQDMARLLREYQ
ELMNTKLALDVEIATYRKLLEGE
```

```
>Human_KRT6A
EREQIKTLNNKFASFIDKVRFLEQQNKVLETKWTLLQEQ-GTK--TVR--QNLEP--
LFEQYINNLRRQLDSIVGERGRLDSELRGMQDLVEDFKNKYEDEINKRTAAENEFVTLKKDVDAAYMNKVELQAKADTLTDEINFLRALYDAEL
SQMQTHIS--DTSVVLSMDNNR-NLDLDSIIAEVKAQYEEIAQRSRAEAES-
YQTKYEELQVTAGRHGDDLRNTKQEIAEINRMIQRLRSEIDHVKKQCANLQAAIADAEQRGEMALKDAKNKLEGLEDALQKAKQDLARLLKEYQ
ELMNVKLALDVEIATYRKLLEGE
>Human_KRT6B
EREQIKTLNNKFASFIDKVRFLEQQNKVLDTKWTLLQEQ-GTK--TVR--QNLEP--
LFEQYINNLRRQLDNIVGERGRLDSELRNMQDLVEDLKNKYEDEINKRTAAENEFVTLKKDVDAAYMNKVELQAKADTLTDEINFLRALYDAEL
SQMQTHIS--DTSVVLSMDNNR-NLDLDSIIAEVKAQYEEIAQRSRAEAES-
YQTKYEELQITAGRHGDDLRNTKQEIAEINRMIQRLRSEIDHVKKQCANLQAAIADAEQRGEMALKDAKNKLEGLEDALQKAKQDLARLLKEYQ
ELMNVKLALDVEIATYRKLLEGE
>Human_KRT6C
EREQIKTLNNKFASFIDKVRFLEQQNKVLDTKWTLLQEQ-GTK--TVR--QNLEP--
LFEQYINNLRRQLDSIVGERGRLDSELRNMQDLVEDLKNKYEDEINKRTAAENEFVTLKKDVDAAYMNKVELQAKADTLTDEINFLRALYDAEL
SQMQTHIS--DTSVVLSMDNNR-NLDLDSIIAEVKAQYEEIAQRSRAEAES-
YQTKYEELQVTAGRHGDDLRNTKQEIAEINRMIQRLRSEIDHVKKQCASLQAAIADAEQRGEMALKDAKNKLEGLEDALQKAKQDLARLLKEYQ
ELMNVKLALDVEIATYRKLLEGE
>Lizard_KRT5L4
EKEQIKTLNNKFASFIDKVRFLEQQNKVLETKWSLLQDQ------KITR-HNFDA--
MFEAYITNLRRQLEGILGERPRLESELKNMQDTVEDFRIRYEDEINKRTAAENEFVVLKKDVDAAFMNKVELEARVDSINDEATFLKSFYDAEL
AQMHAQVS--DTSVILQMDNSR-NLDLDSIIAEVKAQYEDIANRSRAEAES-
YQSKYEELQLSAGRHGDDLHNTKIEISELNRMIQRLHSEIDNVKKQCAGLQASIADAEQRGELALKDARNKLAGLEEALTKAKADMARQLREYQ
ELMNVKLALDIEIATYRKLLEGE
>Alligator_KRT5L4
EKEQIKTLNNKFASFIDKVRFLEQQNKVLETKWSLLQEH------KTAR-SNIEP--
MFEAYISNLRRQVEVLGGERIRLDSELKGMQDVVEDFRIRYEDEIHKRTAAENEFVVLKKDVDAAYMNKVELGAKVDGLTDEINFLRALYEAEL
AQMQSQIS--ETSVVLSMDNNR-NLDLDSIIEEVKAQYEDIANRSRAEAES-
YQSKYEELQLTAGRHGDDLRNTKVEITEMSRMIHRLHSEIDSVKKQCASLQTAIADAEQRGELALKDARVKLAELEDALQKAKQDMARQLREYQ
ELMNVKLALDIEIATYRKLLEGE
>Human_KRT7
ESEQIKTLNNKFASFIDKVRFLEQQNKLLETKWTLLQEQ------KSAKSSRLPD--
IFEAQIAGLRGQLEALQVDGGRLEAELRSMQDVVEDFKNKYEDEINHRTAAENEFVVLKKDVDAAYMSKVELEAKVDALNDEINFLRTLNETEL
TELQSQIS--DTSVVLSMDNSR-SLDLDGIIAEVKAQYEEMAKCSRAEAEA-
YQTKFETLQAQAGKHGDDLRNTRNEISEMNRAIQRLQAEIDNIKNQRAKLEAAIAEAEERGELALKDARAKQEELEAALQRGKQDMARQLREYQ
ELMSVKLALDIEIATYRKLLEGE
>Frog_krt62
EREQIKTLNNKFASFIDKVRFLEQQNKVLETKWSLLQEQ-GGQVKGSRK-NNIDP--
IFDAYINSLKRQLDALQNDKLRLDGELRNMQDLVDDFKNKYEDEINKRTSAENEFVVIKKDVDAAYMGKVELEAKVDALTDELNFLRTFFEAEL
GELQAQIS--DTSVVLSMDNNR-ALDLESIIAEVKSQYEDIAKKSRAEAES-
YQGKVQELQASAGEQGDVLRNTKSEISELNRKSQRLKAEIENVKKQIAKLQASITEAEERGDLVLKDAQSKLAELEAALQKVKQDMARQLREYQ
ELMNVKLALDVEIATYRKLLEGE
>Alligator_KRT8L1
EKEQIKSLNNKFASFIDKVRFLEQQNQVLETKWSLLQGQ------RTTR-SNMTG--
MFEAYITNLRRQLEGLDQDKLRLESELGNVQGLVEEFRSKYEDEINHRTEKENEFVLLKKDVDEAYMNKVELESRLEGLTDEISFLRQLYEEEL
HQLQSQVS--NTSVVLSMDNNR-SLDMDSIIADVRNQYEDIANQSRAEAEG-
YKIKYEELRNTASKHGDDLRNTKAEISELTRTIQRMQAEIDAVKGQRANLEAAIAEAEERGEMAVRDARGKLAELEAALQKAKQDMARQLREYQ
ELMNVKLALDIEIATYRKLLEGE
>Frog_krt8
EKEQIKTLNNKFASFIDKVRFLEQQNKMLETKWSLLQNQ------KATR-SNMDA--
MFEAYIGNLRRQLDGLGQDKMRLESELGNMQGLVEDFKNKYEDEINKRTELENEFVLLKKDVDEAYMNKVQLEARLEALTDEINFLRQLYEEEL
RELQSQIS--DTSVVLSMDNNR-SLDLDGIIAEVRAQYEDIANKSRLEVES-
YQVKYQELQASAGRHGDDLKNTKAEISELTRYTTRLQSEIDALKAQRSNLEAQIAEAEERGELALKDARTKLAELEAALKNAKQDMARQLREYQ
ELMNVKLALDIEIATYRKLLEGE
>Lizard_KRT8
EKEEIKSLNNKFAGFIDKVRFLEQQNKMLDTKWSLLQNQ------KITR-SNMDS--
MFEAYINNLRRQLDGLGQERVRLDSELGNMQGLVEEFKSKYEDEINHRTEKENDFVLLKKDVDEAYMNKIELESRLESLTDELNFLRQLHEEEL
RVLQSQIS--DTSVVLSMDNNR-SLDLDGIIAEVKAQYEEVAARSRAEAES-
YKSKYEELQSVAGKHGDDLRITKNEISELNRLITRIQSEIDGLKGQRANLESAIAEAEERGELAVKDATAKLSELEAALQRAKQDMARQLREYQ
ELMNVKLALDIEIATYRKLLEGE
>Human_KRT8
EKEQIKTLNNKFASFIDKVRFLEQQNKMLETKWSLLQQQ------KTAR-SNMDN--
MFESYINNLRRQLETLGQEKLKLEAELGNMQGLVEDFKNKYEDEINKRTEMENEFVLIKKDVDEAYMNKVELESRLEGLTDEINFLRQLYEEEI
RELQSQIS--DTSVVLSMDNSR-SLDMDSIIAEVKAQYEDIANRSRAEAES-
YQIKYEELQSLAGKHGDDLRRTKTEISEMNRNISRLQAEIEGLKGQRASLEAAIADAEQRGELAIKDANAKLSELEAALQRAKQDMARQLREYQ
ELMNVKLALDIEIATYRKLLEGE
>Lizard_KRT7
EKEQIKTLNNKFASFIDKVRFLEQQNKMLETKYSLLQEQ------KTTK-SNLAP--
IFETYISNLRRQLDSLMNDKGRLEGELRNMQDLVEDFKSKYEDEINKRTTAENDFVVLKKDVDASYMNKVELESKVDGLTDEVNFLREFYEAEL
RELHNQIS--DTSVVLQMDNNR-NLDLDSIIAEVKAQYEEIANKSRAEAEA-
YQTKYEALQVTAGKHGDDLRNTKTEISDLNRTIQRMQAEIENIKNQRGKLEAAITEAEERGELAVKDARAKLQELENALNKAKQEMARQLREYQ
ELMNVKMSLDIEIATYRKLLEGE
>Frog_krt7
EREQIKTLNNKFASFIDKVRFLEQQNKVLETKWELLQNQ------KSAKASNVGP--
LFDAYIANLRRQLDGLTGDKGKLEGELRNMQDLVEDFKNKYEDEINKRTSAENEFVVLKKDVDAAYMNKVELEAKMEALTDEINFLRALYEAEL
RELQEQIS--DTSVVLSMDNNR-ALDMDSIIAEVKAQYEDIANKSRANAEA-
YQNKFQELQATAGRHGDDLRTTKTEISELNRAMQRLQAEIESVKAQRAKLEAQIAEAEERGELALKDARTKLAELEAALQKAKQDMARQLREYQ
ELMNVKLALDIEIATYRKLLEGE
```

```
>Alligator_KRT7
EKEQIKTLNNKFASFIDKVRFLEQQNKMLETKWSLLQDQ------KTAR-SNIAP--
LFEAYISNLRRQLDGLMNDKGRLEGELKNMQDLVEDFKNKYEDEINRRTTAENEFVVLKKDVDGAYMNKVELEARVDALTDEINFLRSLYEAEL
QELQTQIS--DTSVVLSMDNSR-NLDLDSIIAEVKAQYEDIANRSRAEAES-
YQSKFEVLQATAGKHGDDLRSTKNEIAETNRMIMRLQSEIDNVKNQRAKLEAAIAEAEERGEMAIKDARAKQDELEAALQKAKQDMARQLREYQ
DLMNVKLALDIEIATYRKLLEGE
>Alligator_KRT78LT
EKEQIKSLNDKFACFIDKVRCLEQENQVLLTKWELLQQQGGQT--GVH--RDIEP--
FFQSYICHLRRQRDTLQSQKEELNLEDCKMLQIVNEYKASYEKEIDRRLCTEEEFVNLKKNLDCIYMGKMEVGVKVNILREEIEFLKCLYAMEL
SDVQRIAQ--DTNVVVSMDNCR-DLDVEGIIAEIRREYEGLAQKSQAEVNA-
YQSKYQDLHNTWGRHCDRLRTRQHEIQELTRLIQKLRLDIGDVKKKNACLQEEIADVERRGCCAVSDAQEKLRELRCSLLKAKDNLAHLLRDYQ
ELLNVKMALDIEIATYRALLEGE
>Lizard_HBS3
EREEMKNLNNQFACFIEKVRILEQENKALETKWNLLQQY------AMVPRRNLEP--
YYENFISNMKYQIDCLLHTREHLANDKNAIQELVEDFKSKYEEEFKRRTTAENEFVLLKKDVDSISLSKTELEGKMNLLRWELEFRKSVYMEEL
MQLSGQIS--DMNILVQMDNNR-DLDMDSIIKNVEAWYQSIAHRSKEEVNA-
YADRFQELQEQRGKYLNDLKINQYEIEELTRAVHTLQSDHSNVKKQVDALQASICDVDQRGDQALKDAQEKHSDLLSALQKAKDDLAGMLKDYH
DLLNVKLALDIEIATYRTLLEGE
>Lizard_HBS2
EKEEMKTLNNQFACFIDKVRSLEQQNKVLGTKWELLNQC------VQPAKKNLEG--
YYENFIATLKKQLECLLSERGKLEHEQKNMQELVEEYRSKYEEEVNRRTTAENEFVVLKKDVDCVFLTKEELESKVDVLAQELEVLRCVFAEEL
TQLDSQIL--DTSVILKMDNTR-DLHMDLILQNVENWYQNIAHNSKQEAEA-
YHNKIAEIQNNRSKFNEELKCNQHEIAELNRVTQRLRTDLGNAKKQVSNLQSAICDAEQRGDIALKDARNKHAELQNAYQQSKDKLASLLRDYQ
DLLNTKMALDIEIATYKMMLEGE
>Lizard_KRT78LT
EREQIKTLNDQFACFIDKVRHLEQQNKLLETKWSCLQQQ------EPMEKQNTEH--
LYQNYIVTLKKQLDLLLDEREQLKLEQVKFQDVVEEYKSRYEGVINRRLGAENEFVVLKKDVDCAYTSKVELEVKVDSLRQEQDFHRCVYDTEL
ESL-ATAS--DTNVVVSMDNSR-DLDMEGIIESVRCQYEEIAQRSKDEVNA-
YETKYKELQTTWGHHCNSLSSSRHEIQELTRLIQRYKADVENGKKQVEALQVAVADHEQQGDCTLKDAKEKLAEVEKALQSTKDELARLLRDYQ
ELLNVKVALDIEIAMYKSLLEGE
>Alligator_HBS3
EREQIKKLNNQFACFIDKVRCLEQQNKVLGTKWNLLQEQ------VLPCRKNLQH--
LFENYLCSLQRQLDCLLSERGQLEPELQNGQKLVEELKCKYEQEINRRTASENEFILLKKDVDCSYMSKVELEAKVDTLRQEIEFLRCVYAQEL
VQMEGNLC--DTSVVVKMDNNR-DLDMEGIIREIECWYEEIAQKSKAEVDA-
YRTRFQELQETKGRYCNDLKCNHHEITELSRVVQRLQHNLDNAKKEVACLQTSICETEQRGDCALKDAQGKYGELQNALQKAKDELASMLRDYQ
ELLNIKLALDIEIAMYKTLLEGE
>Alligator_HBS1
EKEQIKNLNNKFACFIDKVRLLEQQNKVLTTKWDLLQQC-IVP--GTR--RNLEP--
LYESFICNLKKQLEHLLCDRDKLMCEEKAANHLVDEFKCKYEEQINRRTAAENEFVVLKKDVDCLHLTKEELEIRVALLRQQLEFLTCIFAEER
AQMDCQLC--DTSVIVEMDNNR-GLDMDSIINSVKCCYEEIAHKSKAEVEA-
YQTRLEELHTNRGKYCDDLRISQCEIADLKRGIQKLQGELDGVKKQIGCLETAICDAEHHGDCTLKDAREKHINLQTALQQAKDKLACLLRDYQ
ELLNVKLALDIEIATYRSLLEGE
>Alligator_HBS2
EKEQMKCLNNQFACFIDKVQCLEQQNKALETKWNLLQQH-TMP--AAR--KTLEA--
YFENYICKLKKQLECLLGEREQLSNKECVAKKLVDEFRCKYEEVINKRMTAEHGFVLAKKDVDYAFLNTEEQEVKVDLLKCQLELLNHVFTEER
AQMDCQLC--DTSVMVKMDNRR-DLDMETIIKNVECCYKEIAQKSKEEVDT-
YQTRFQELQEQRGRFCDDLESNKREIAQLTQLIQELQCELDNVKKQVGCLQTDIRDAEQHGDFTLQDGRAKHVELQNALQKAKDELASMLRDYQ
ELLNVKLALDIEIAMYKTLLEGE
```

Figure S10. Multiple sequence alignment used for the type II keratin phylogeny of amniote species and sea lamprey in Figure S6. The trimmed amino acid sequences are provided in fast_align format. Species: alligator (*Alligator sinensis*), frog (*Xenopus tropicalis*), hagfish (*Eptatretus burgeri*), human (*Homo sapiens*), lamprey (*Petromyzon marinus*), lizard (*Anolis carolinensis*).

>Eb-krt18.15
SEKETLQQLNDRLDDYLKRVRSLESYNSKIEMEIKHLITSYGPEMIDWESQEKHLEELRVQLAELTLDNANLSMQAETNYLSIKDFQIKLEIEQ
NLCHNIEMDINEMRKEIDSINLNILELEGNNESLVASLVMMKKQHKERVKELKANIAVEDLNVQVDSIQGMDLLAVLTSIREEYNRLSKKNEQD
AEVWYREKIQNIKEVVGQGEAELNPLNEQLHSLRQEHHNLQLTINSSGTKVSESQRITTSQSIRSS----------------------------
----
>Ea-krt18.9
SEKETLQQLNDRLDDYLKRVRSLESYNSKIEMEIKHLITSYGPEMIDWESQEKHLEELRVQLAELTLDNANLSMQAETNYLSIKDFQMKLEIEQ
NLCHNIEMDINEMRKEIDSINLNVLELEGNNESLVASLMMMKKQHKERVKELKANIAVEDLNVQVDSIQGMDLLAVLTSIREEYNRLSKKNEQD
AEVWYREKIQNIKEVVGQGEAELNPLDEQLHSLRQEHHNLQLTINSSGTKKEFLRKSLTDVEIHFNSERNRMKGYMGQYESTLAGLKEELEKQL
GAYEQLMSTKIRLEQELAEYHNLLEHG
>Eb-krt18.14
SEKETLQQLNDRLDDYLKRVRSLESYNSKIEMEIKHLITSYGPEMIDWESQEKHLEELRVQLAELTLDNANLSMQAETNYLSIKDFQIKLEIEQ
NLCHNIEMDINEMRKEIDSINLNILELEGNNESLVASLVMMKKQHKERVKELKANIAVEDLNVQVDSIQGMDLLAVLTSIREEYNRLSKKNEQD
AEVWYREKIQNIKEVVGQGEAELNPLNEQLHSLRQEHHNLQLTINSSGTKKEFLRKSLTDVEIHFNSERNRMKGYLGQYESTLAGLKEAGK---
------------------TARCL----
>Ea-krt18.7
NEKEHLRQLNDRLAGYMDKVKLLEQTNQNLENQIKEIF-
GKVNQTPDLSNFYNQVEELKGNAHRISVENTQLALAIDNGKEEAKEFRIKIESETAAMRAIEYDIGSLNQIVEKIQTERKHLDEQLGSLQREME
SIRSNHNEKLQGLRVAANDEGVHVKVDDVRGADLVQILADMRSEYENITQKSRADIEAIYQTQIEHANPNTAQNLEAIELAKKQANDLRRQMQA
LTMDIQTQSNAISSLKSTLNHSEERYSYELNKFLPITGYLEEELSKIKGNLEHQRQEYDTALNAKMMLEMEIATYRQLLEAG
>Ea-krt18.10
NEQTQLKDLNERLGIYLERVSYLEKINGEMEASIKVELEQRAANVPDWTRYEQIIKDLRQKINDTINENTSLSLEIDNNRFAGSDFRQKWETEN
DLYQAVEGDIASLRSQTNDINLSCSELETHLECLRSDLESMRRQHKEEKEKLSKGLSDG-
MSVEVDAVKGINLNEILNGVRNKYEEIVKKNQQEAEEEYQSKCKMAAPQMMKSCEALEASRSEVTELRSLLQTLSIEYQSLNSMVFSLEEMLQE
TEDRKANELSRLQSSLLKAEANLEGAQKNLRGQMQEFDILLDAKMDLEREIAMYHKLINGE
>Eb-krt18.13
NEQTQLKDLNERLGVYLERVSYLEKINGEMEASIKVELEQRAANVPDWTRYEQIIKDLRQKINDTINENTSLSLEIDNNRFAGSDFRQKWETEN
DLYQAVEGDIASLRSQTNDINLSCSELETHLECLRSDLESMRRQHKEEKEKLSKGLSDG-
MSVEVDAVKGINLNEILNGVRNKYEEIVKKNQQEAEEEYQSKCKMAVPQMMKSCEALEASRSEVTELRSLLQTLSIEYQSLNSMVFSLEEMLQE
TEDRKANELSRLQSSLLKAEANLEGAQKNLRGQMQEFDILLDAKMDLEREIAMYHKLINGE
>Pm-krt18.2
NEKEELQGLNNRLSGYLQRVRTLEQDNNKIELEIAELMSLRKGGPHDWAKWDGIFRALREEIFMLTMDTTKLELGIQNTDLARSDYYEKWETEH
AIRMAVEADIQALRKMIDDTNVARLNLESRVEALKEELSVLRYTHAEEVKKLLVEISSFEVSVEVDSTKDIDLNKALAEIRRQYEDLCRQNAVQ
VESSYLETITITNTKVTEKMQALEKYRVELNELRRTTQVLFLDLENMRSKKLSLENANADMSARYEMEVSSLRDMVIMLDKILVDVNMDLERQR
SEYEMLTNAKMRLEQEIAYYSSLLDQE
>Pm-krt18.8
NEKEALQDLNERLAGYLDKVRYLEQANQELECKIKEFRANSCIAEQQWEPCMGNIEELMGQVEVATLENTRLVLEIDNARLASEDFRVKWEAES
ALRETVEADAHELRRLSAEYCACRNQLACEQNVLQGEIHTLKSHHRGEMESLRMDYSSSTTKVEMDNSPGADTAGIISEIRAQYETMIHNNRHE
AECMLQSKLEAAEASAVHSHGELAAAKNQAHHLRQQYQTMEVEMESLRSANATLEDNLAETEERFSVEVRSLAEVLSRLEAEYSDVRANVERQL
HEYESLLNIKMGLEMEISTYKCLIEGE
>Lr-krt18.1
NEKEALQDLNERLAGYLDKVRYLEQANQELECKIKEFRANSCIAEQQWEPCMGNIEELMGQVEVATLENTRLVLEIDNARLASEDFRIKWEAES
ALRETVEADAHELRRLSAEYCACRNQLACEQNVLQGEIHTLKDHHRGEMESLRMDYSSSTTKVEMDNSPGADTAGIISEIRAQYETMIHNNRHE
AECMLHSKLEAAEASAVHSHGELSAAKSQAHHLRQQFQTLEVEMESLRSANATLEDNLAETEERFSVEVRSLAEVLSRLEAEYSDVRANVERQL
HEYESLLNIKMGLEMEISTYKCLIEGE
>Ea-krt18.1
NEKQTMQDLNDRLSGYLDKVRYLEATNLELECKIKEFRANKCIAEREWDPCLSGIESLMYQIEEASVENSQLVLAIDNARLTAEDFRTKWEIEV
SLREDLEIDIQDLRNLANEYCECRNTVACEINVLEDEICHLKNSHREEIEALRHEYANSTLNVDIDHTPGVDLAGIINDIRTQYETMISSTHYE
VECLIQNKLDAAQASAHQSACNLEAIKTEANNLRYEFQALEVEAESLRSANCILEENLHETEARLNLEISNLAQIISILEADYTDVRSNIERQI
REYESLLNVKMQLEMEIATYQQLIDCE
>Eb-krt18.9
NEKQTMQDLNDRLSGYLDKVRYLEATNLELECKIKEFRANKCIAEREWDPCLSGIESLMYQIEEASVENSQLVLAIDNARLTAEDFRTKWEIEV
SLREDLEIDIQDLRNLANEYCECRNTVACEINVLEDEICHLKNSHRE------
VWYANSTLNVDIDHTPGVDLAGIINDIRTQYETMIASTHYE--------LDAAQASAHQSACNLEAVKTEANNLRYEFQALEVEAESLRS-
NCILEENLHETEARLNLEISNLAQIISILEADYADVRSNIERQIREYESLLNVKMQLEMEIC-----WGGG
>Eb-krt18.8
DEKGAMQDLNDRLAGYLGKVRYLEGANRELECKIKD-----------------
GIESLMYQIEEASVENSQLVLAIDNARLTAEDFRKKWEIEVSLREELETDIQDLRNLANEYCECHNTVACEVNVIQDEIYYLKQSHR-
EIEGLRCEYANSTLNVDIDHSPNVDLSSIINDIRTQYETMIGNTHYEVECLMNNK----------------
ATKNEANNMRYEFQSLEVEAESLHT-
NCTLEENLRETEERLNLELSNLAEIISSLEAEYTDVRANIERQIREYESLLNVKMQLEMEIGTYQKLIEGE
>Ea-krt18.5
NEKQTMQDLNDRLSSYLDKVRYLEATNLELECKIKEFRANKCIAEREWDPCLSGIESLMYQIEEASVENSQLVLAIDNARLTAEDFRKKWEIEV
SLREELETDIQDLRNLANEYCECHNTVACEVNVIQDEIYYLKQSHREEIEGLRCEYANSTLNVDIDHSPNVDLSSIINDIRTQYETMIGNTHYE
VECLMNNKLEASQASAHQSACELEATKNEANNLRYEFQSLEVEAESLHTANCTLEENLHETEERLNLELSNLAEIISSLEAEYTDVRANIERQI
REYESLLNVKMQLEMEIGTYQQLIEGE
>Pm-krt18.6
DEKAEMQGLNGRLATYIERVRSLDEANRRTELQIKELVEKRPSGAVELRQYHEAARELREQILKATMTNARLHVELDNGRLAAEDFRAKLESEV
SIHTSVESDITNLRRAIDETNVTRMSLEGQVEQLEEQILHMQKSHADEKSSLLKEIEESSISVEVDSVKGHNLNDIIAEIRTQYEALIKSNLQE
MEVWYKSKVDAIHPRISQNSEELGTLRQQLSEQRRAMQALYAEAETLRSTVSSLNEAAQDVEAHSAEGLAGLAGSISRLLSELGSARGDIDRQL
REHETLLNTKMRLEEEIETYRRLLEGQ
>Lr-krt18.4
DEKAEMQGLNGRLATYIERVRSLDEANRRTELQIKELVEKRPSGAVELRQYHEAARELRGQILKATMTNARLHVELDNGRLAAEDFRAKLESEV
CIHTSVESDITNLRRAIDETNVTRMSLEGQVELLEEQLLHMQKSHADEKSSLLKEIEESSISVEVDSVKGHNLNDIIAEIRAQYEALIKSNLQE
MEVWHKSKVDEIHPRISQNSEELGTLRQQLSEQRRAMQALYAEAETLRCTVSSLNEAAQDVEAHSAEGLAGLAGSISRLLSELGSARGDIDRQL
REHEALLNTKMRLEEEIETYRRLLEGQ
>Lr-krt18.5

DEKAEMQGLNGRLATYIERVRSLDEANRRTELQIKELVEKRPSGAVELRQYHEAARELRGQILKATMTNARLHVELDNGRLAAEDFRAKLESEV
CIHTSVESDITNLRRAIDETNVTRMSLEGQVELLEEQLLHMQKSHADEKSSLLKEIEESSISVEVDSVKGHNLNDIIAEIRAQYEALIKSNLQE
MEVWHKSKVDEIHPRISQNSEELGTLRQQLSEQRRAMQALYAEAETLRCTVSSLNEAAQDVEAHSAEGLAGLAGSISRLLSELGSARGDIDRQL
REHEALLNTKMRLEEEIETYRRLLEGQ
>Hs-KRT23
NGKATMQNLNDRLASYLEKVRALEEANMKLESRILKWHQQRDPGSKDYSQYEENITHLQEQIVDGKMTNAQIILLIDNARMAVDDFNLKYENEH
SFKKDLEIEVEGLRRTLDNLTIVTTDLEQEVEGMRKELILMKKHHEQEMEKHHVPSDFN-
VNVKVDTGPREDLIKVLEDMRQEYELIIKKKHRDLDTWYKEQSAAMSQEAA-
SPATVQSRQGDIHELKRTFQALEIDLQTQYSTKSALENMLSETQSRYSCKLQDMQEIISHYEEELTQLRHELERQNNEYQVLLGIKTHLEKEIT
TYRRLLEGE
>Hs-KRT18
NEKETMQSLNDRLASYLDRVRSLETENRRLESKIREHLEKKGPQVRDWSHYFKIIEDLRAQIFANTVDNARIVLQIDNARLAADDFRVKYETEL
AMRQSVENDIHGLRKVIDDTNITRLQLETEIEALKEELLFMKKNHEEEVKGLQAQIASSGLTVEVDAPKSQDLAKIMADIRAQYDELARKNREE
LDKYWSQQIEESTTVVTTQSAEVGAAETTLTELRRTVQSLEIDLDSMRNLKASLENSLREVEARYALQMEQLNGILLHLESELAQTRAEGQRQA
QEYEALLNIKVKLEAEIATYRRLLEDG
>Hs-KRT20
NEKMAMQNLNDRLASYLEKVRTLEQSNSKLEVQIKQWYETNAPRAGDYSAYYRQIEELRSQIKDAQLQNARCVLQIDNAKLAAEDFRLKYETER
GIRLTVEADLQGLNKVFDDLTLHKTDLEIQIEELNKDLALLKKEHQEEVDGLHKHLGNT-
VNVEVDAAPGLNLGVIMNEMRQKYEVMAQKNLQEAKEQFERQTAVLQQQVTVNTEELKGTEVQLTELRRTSQSLEIELQSHLSMKESLEHTLEE
TKARYSSQLANLQSLLSSLEAQLMQIRSNMERQNNEYHILLDIKTRLEQEIATYRRLLEGE
>Hs-KRT9
NEKSTMQELNSRLASYLDKVQALEEANNDLENKIQDWYDKKGPAAINYSPYYNTIDDLKDQIVDLTVGNNKTLLDIDNTRMTLDDFRIKFEMEQ
NLRQGVDADINGLRQVLDNLTMEKSDLEMQYETLQEELMALKKNHKEEMSQLTGQNSGD-
VNVEINVAPGKDLTKTLNDMRQEYEQLIAKNRKDIENQYETQITQIEHEVSSSGQEVQSSAKEVTQLRHGVQELEIELQSQLSKKAALEKSLED
TKNRYCGQLQMIQEQISNLEAQITDVRQEIECQNQEYSLLLSIKMRLEKEIETYHNLLEGG
>Hs-KRT26
NEKVTMQNLNDRLASYLDHVHALEEANADLEQKIKGWYEKCEPGSSDYSRYFSVIEDLKRQIISATICNASIVLQNDNARLTADDFRLKYENEL
ALHHSVEADTSGLRRVLDELTLCTTDLEIQCETLSEELTYLKKSHEEEMEVLQYTAGGN-
VNVEMNATPGVDLTVLLNNMRAEYEDLAEQNRKDAEAWFNERSATLQQQISDHEGAATAARNELTELKRNLQTLEIELQSLMAVKHSYECSLAE
TEGNYCNQLQQIQDQIGVMEEQLQQIRTETEGQKLEYEQLLDVKIFLEKEIDIYCNLLDGE
>Hs-KRT28
NEKVTMQNLNDRLASYLDNVRALEEANAELERKIKGWYEKYGPGSCDYSRYHLTIEDLKNKIISSTTTNANVILQIDNARLAADDFRLKYENEL
TLHQNVEADINGLRRVLDELTLCRTDQELQYESLSEEMTYLKKNHEEEMKALQCAAGGN-
VNVEMNAAPGVDLAVLLNNMRAEYEALAEQNRKDAEAWFNEKSASLQQQISHDSGAATFARSQLTEMRRTLQTLEIQLQSLMATKHSLECSLTE
TESNYCTQLAQIQAQIGALEEQLHQVRTETEGQKLEYEHLLDVKVHLEKEIETYCRLIDGD
>Hs-KRT25
NEKVTMQNLNDRLASYLDSVHALEEANADLEQKIKGWYEKFGPGSCDYSRYFPIIDDLKNQIIASTTSNANAVLQIDNARLTADDFRLKYENEL
ALHQSVEADVNGLRRVLDEITLCRTDLEIQYETLSEEMTYLKKNHKEEMQVLQCAAGGN-
VNVEMNAAPGVDLTVLLNNMRAEYEALAEQNRRDAEAWFNEKSASLQQQISEDVGATTSARNELTEMKRTLQTLEIELQSLLATKHSLECSLTE
TESNYCAQLAQIQAQIGALEEQLHQVRTETEGQKLEYEQLLDIKLHLEKEIETYCLLIGGD
>Hs-KRT27
NEKVTMQNLNDRLASYLENVRALEEANADLEQKIKGWYEKFGPGSCDYSRYFPIIDELKNQIISATTSNAHVVLQNDNARLTADDFRLKFENEL
ALHQSVEADINGLRRVLDELTLCRTDLEIQLETLSEELAYLKKNHEEEMKALQCAAGGN-
VNVEMNAAPGVDLTVLLNNMRAEYEALAEQNRRDAEAWFNEKSASLQQQISDDAGATTSARNELIEMKRTLQTLEIELQSLLATKHSLECSLTE
TESNYCAQLAQIQAQIGALEEQLHQVRTETEGQKLEYEQLLDIKVHLEKEIETYCLLIDGE
>Hs-KRT24
GEKQTMQNLNDRLANYLDKVRALEEANTDLENKIKEWYDKYGPGSGDYSKYYSIIEDLRNQIIAATVENAGIILHIDNARLAADDFRLKYENEL
CLRQSVEADINGLRKVLDDLTMTRSDLEMQIESFTEELAYLRKNHEEEMKNMQGSSGGE-
VTVEMNAAPGTDLTKLLNDMRAQYEELAEQNRREAEERFNKQSASLQAQISTDAGAATSAKNEITELKRTLQALEIELQSQLAMKSSLEGTLAD
TEAGYVAQLSEIQTQISALEEEICQIWGETKCQNAEYKQLLDIKTRLEVEIETYRRLLDGE
>Hs-KRT19
NEKLTMQNLNDRLASYLDKVRALEAANGELEVKIRDWYQKQGPGPSDYSHYYTTIQDLRDKILGATIENSRIVLQIDNARLAADDFRTKFETEQ
ALRMSVEADINGLRRVLDELTLARTDLEMQIEGLKEELAYLKKNHEEEISTLRGQVGGQ-
VSVEVDSAPGTDLAKILSDMRSQYEVMAEQNRKDAEAWFTSRTEELNREVAGHTEQLQMSRSEVTDLRRTLQGLEIELQSQLSMKAALEDTLAE
TEARFGAQLAHIQALISGIEAQLGDVRADSERQNQEYQRLMDIKSRLEQEIATYRSLLEGQ
>Hs-KRT16
SEKVTMQNLNDRLASYLDKVRALEEANADLEVKIRDWYQRQRPSEIDYSPYFKTIEDLRNKIIAATIENAQPILQIDNARLAADDFRTKYEHEL
ALRQTVEADVNGLRRVLDELTLARTDLEMQIEGLKEELAYLRKNHEEEMLALRGQTGGD-
VNVEMDAAPGVDLSRILNEMRDQYEQMAEKNRRDAETWFLSKTEELNKEVASNSELVQSSRSEVTELRRVLQGLEIELQSQLSMKASLENSLEE
TKGRYCMQLSQIQGLIGSVEEQLAQLRCEMEQQSQEYQILLDVKTRLEQEIATYRRLLEGE
>Hs-KRT17
GEKATMQNLNDRLASYLDKVRALEEANTELEVKIRDWYQRQAPGPADYSQYYRTIEELQNKILTATVDNANILLQIDNARLAADDFRTKFETEQ
ALRLSVEADINGLRRVLDELTLARADLEMQIENLKEELAYLKKNHEEEMNALRGQVGGE-
INVEMDAAPGVDLSRILNEMRDQYEKMAEKNRKDAEDWFFSKTEELNREVATNSELVQSGKSEISELRRTMQALEIELQSQLSMKASLEGNLAE
TENRYCVQLSQIQGLIGSVEEQLAQLRCEMEQQNQEYKILLDVKTRLEQEIATYRRLLEGE
>Hs-KRT14
SEKVTMQNLNDRLASYLDKVRALEEANADLEVKIRDWYQRQRPAEIDYSPYFKTIEDLRNKILTATVDNANVLLQIDNARLAADDFRTKYETEL
NLRMSVEADINGLRRVLDELTLARADLEMQIESLKEELAYLKKNHEEEMNALRGQVGGD-
VNVEMDAAPGVDLSRILNEMRDQYEKMAEKNRKDAEEWFFTKTEELNREVATNSELVQSGKSEISELRRTMQNLEIELQSQLSMKASLENSLEE
TKGRYCMQLAQIQEMIGSVEEQLAQLRCEMEQQNQEYKILLDVKTRLEQEIATYRRLLEGE
>Hs-KRT13
NEKITMQNLNDRLASYLEKVRALEEANADLEVKIRDWHLKQSPASPDYSPYYKTIEELRDKILTATIENNRVILEIDNARLAADDFRLKYENEL
ALRQSVEADINGLRRVLDELTLSKTDLEMQIESLNEELAYMKKNHEEEMKEFSNQVVGQ-
VNVEMDATPGIDLTRVLAEMREQYEAMAERNRRDAEEWFHTKSAELNKEVSTNTAMIQTSKTEITELRRTLQGLEIELQSQLSMKAGLENTVAE
TECRYALQLQQIQGLISSIEAQLSELRSEMECQNQEYKMLLDIKTRLEQEIATYRSLLEGQ
>Hs-KRT15
NEKITMQNLNDRLASYLDKVRALEEANADLEVKIHDWYQKQTPTSPDYSQYFKTIEELRDKIMATTIDNSRVILEIDNARLAADDFRLKYENEL
ALRQGVEADINGLRRVLDELTLARTDLEMQIEGLNEELAYLKKNHEEEMKEFSSQLAGQ-

```
VNVEMDAAPGVDLTRVLAEMREQYEAMAEKNRRDVEAWFFSKTEELNKEVASNTEMIQTSKTEITDLRRTMQELEIELQSQLSMKAGLENSLAE
TECRYATQLQQIQGLIGGLEAQLSELRCEMEAQNQEYKMLLDIKTRLEQEIATYRSLLEGQ
>Hs-KRT12
SEKETMQNLNDRLASYLDKVRALEEANTELENKIREWYETRGTGTADYSKYYPLIEDLRNKIISASIGNAQLLLQIDNARLAAEDFRMKYENEL
ALRQGVEADINGLRRVLDELTLTRTDLEMQIESLNEELAYMKKNHEDELQSFRVGGPGE-
VSVEMDAAPGVDLTRLLNDMRAQYETIAEQNRKDAEAWFIEKSGELRKEISTNTEQLQSSKSEVTDLRRAFQNLEIELQSQLAMKKSLEDSLAE
AEGDYCAQLSQVQQLISNLEAQLLQVRADAERQNVDHQRLLNVKARLELEIETYRRLLDGE
>Hs-KRT10
NEKVTMQNLNDRLASYLDKVRALEESNYELEGKIKEWYEKHGNSHQDYSKYYKTIDDLKNQILNLTTDNANILLQIDNARLAADDFRLKYENEV
ALRQSVEADINGLRRVLDELTLTKADLEMQIESLTEELAYLKKNHEEEMKDLRNVSTGD-
VNVEMNAAPGVDLTQLLNNMRSQYEQLAEQNRKDAEAWFNEKSKELTTEIDNNIEQISSYKSEITELRRNVQALEIELQSQLALKQSLEASLAE
TEGRYCVQLSQIQAQISALEEQLQQIRAETECQNTEYQQLLDIKIRLENEIQTYRSLLEGE
>Hs-KRT37
HEKETMKFLNDRLANYLEKVRQLEQENAELETTLLERSKCHESTVCDYQSYFRTIEELQQKILCSKAENARLIVQIDNAKLAADDFRIKLESER
SLHQLVEADKCGTQKLLDDATLAKADLEAQQESLKEEQLSLKSNHEQEVKILRSQLGEK-
FRIELDIEPTIDLNRVLGEMRAQYEAMVETNHQDVEQWFQAQSEGISLQAMSCSEELQCCQSEILELRCTVNALEVERQAQHTLKDCLQNSLCE
AEDRYGTELAQMQSLISNLEEQLSEIRADLERQNQEYQVLLDVKARLENEIATYRNLLESE
>Hs-KRT38
HEKETMQFLNDRLANYLEKVRQLEQENAELEATLLERSKCHESTVCDYQSYFHTIEELQQKILCSKAENARLIVQIDNAKLAADDFRIKLESER
SLRQLVEADKCGTQKLLDDATLAKADLEAQQESLKEEQLSLKSNHEQEVKILRSQLGEK-
LRIELDIEPTIDLNRVLGEMRAQYEAMLETNRQDVEQWFQAQSEGISLQDMSCSEELQCCQSEILELRCTVNALEVERQAQHTLKDCLQNSLCE
AEDRFGTELAQMQSLISNVEEQLSEIRADLERQNQEYQVLLDVKTRLENEIATYRNLLESE
>Hs-KRT39
NEKETMQILNERLANYLQKVRMLERENAELESKIQEESNKELPVLCDYLSYYTTIEELQQKILCTKAENSRLVSQIDNTKLTADDLRAKYEAEV
SLRQLVESDANGLKQILNVLTLGKADLEAQVQSLKEELLCLKNNHKEEINSLQCQLGER-
LDIEVTAAPSADLNQVLQEMRCQYEPIMETNRKDVEQWFNTQIEELNQQVVTSSQQQQCCQKEIIELRRSVNTLEVELQAQHRMRDSQECILTE
TEARYTALLTQIQSLIDNLEAQLAEIRCALERQNQEYEILLDVKSRLECEITTYRSLLESS
>Hs-KRT40
NEKETMQFLNDRLASYLEKVRSLEETNAELESRIQEQCEQDIPMVCDYQRYFNTIEDLQQKILCTKAENSRLAVQLDNCKLATDDFKSKYESEL
SLRQLLEADISSLHGILEELTLCKSDLEAHVESLKEDLLCLKKNHEEEVNLLREQLGDR-
LSVELDTAPTLDLNRVLDEMRCQCETVLANNRREAEEWLAVQTEELNQQQLSSAEQLQGCQMEILELKRTASALEIELQAQQSLTESLECTVAE
TEAQYSSQLAQIQCLIDNLENQLAEIRCDLERQNQEYQVLLDVKARLEGEINTYWGLLDSE
>Hs-KRT32
NEKETMQFLNDRLASYLTRVRQLEQENAELESRIQEASHSQVLTMTDYQSHFRTIEELQQKILCTKAENARMVVNIDNAKLAADDFRAKYEAEL
AMRQLVEADINGLRRILDDLTLCKADLEAQVESLKEELMCLKKNHEEEVGSLRCQLGDR-
LNIEVDAAPPVDLTRVLEEMRCQYEAMVEANRRDVEEWFNMQMEELNQQVATSSEQLQNYQSDIIDLRRTVNTLEIELQAQHSLRDSLENTLTE
SEARYSSQLAQMQCMITNVEAQLAEIRADLERQNQEYQVLLDVRARLEGEINTYRSLLENE
>Hs-KRT34
SEKETMQFLNDRLASYLEKVRQLERDNAELEKLIQERSQQQEPLLCSYQSYFKTIEELQQKILCAKAENARLVVNIDNAKLASDDFRSKYQTEQ
SLRLLVESDINSIRRILDELTLCKSDLESQVESLREELICLKKNHEEEVNTLRSQLGDR-
LNVEVDTAPTVDLNQVLNETRSQYEALVEINRREVEQWFATQTEELNKQVVSSSEQLQSCQAEIIELRRTVNALEIELQAQHNLRDSLENTLTE
SEAHYSSQLSQVQSLITNVESQLAEIRCDLERQNQEYQVLLDVRARLECEINTYRSLLESE
>Hs-KRT33B
SEKETMQFLNDRLASYLEKVRQLERDNAELENLIRERSQQQEPLLCSYQSYFKTIEELQQKILCSKSENARLVVQIDNAKLAADDFRTKYQTEQ
SLRQLVESDINSLRRILDELTLCRSDLEAQMESLKEELLSLKQNHEQEVNTLRCQLGDR-
LNVEVDAAPAVDLNQVLNETRNQYEALVETNRREVEQWFATQTEELNKQVVSSSEQLQSYQAEIIELRRTVNALEIELQAQHNLRYSLENTLTE
SEARYSSQLSQVQSLITNVESQLAEIRSDLERQNQEYQVLLDVRARLECEINTYRSLLESE
>Hs-KRT33A
SEKETMQFLNDRLASYLEKVRQLERDNAELENLIRERSQQQEPLVCSYQSYFKTIEELQQKILCSKSENARLVVQIDNAKLASDDFRTKYETEL
SLRQLVESDINGLRRILDELTLCRSDLEAQVESLKEELLCLKQNHEQEVNTLRCQLGDR-
LNVEVDAAPTVDLNQVLNETRSQYEALVETNRREVEQWFATQTEELNKQVVSSSEQLQSYQAEIIELRRTVNALEIELQAQHNLRDSLENTLTE
SEARYSSQLSQVQRLITNVESQLAEIRSDLERQNQEYQVLLDVRARLECEINTYRSLLESE
>Hs-KRT31
SEKETMQFLNDRLASYLEKVRQLERDNAELENLIRERSQQQEPLLCSYQSYFKTIEELQQKILCTKSENARLVVQIDNAKLAADDFRTKYQTEL
SLRQLVESDINGLRRILDELTLCKSDLEAQVESLKEELLCLKSNHEQEVNTLRCQLGDR-
LNVEVDAAPTVDLNRVLNETRSQYEALVETNRREVEQWFTTQTEELNKQVVSSSEQLQSYQAEIIELRRTVNALEIELQAQHNLRDSLENTLTE
SEARYSSQLSQVQSLITNVESQLAEIRSDLERQNQEYQVLLDVRARLECEINTYRSLLESE
>Hs-KRT36
SEKETMQFLNDRLANYLEKVRQLERENAELESRIQEWYEFQIPYICDYQSYFKTIEDFQQKILLTKSENARLVLQIDNAKLAADDFRTKYETEL
SLRQLVEADINGLRRILDELTLCKADLEAQVESLKEELMCLKKNHEEEVSVLRCQLGDR-
LNVEVDAAPPVDLNKILEDMRCQYEALVENNRRDVEAWFNTQTEELNQQVVSSSEQLQCCQTEIIELRRTVNALEIELQAQHSMRNSLESTLAE
TEARYSSQLAQMQCLISNVEAQLSEIRCDLERQNQEYQVLLDVKARLEGEIATYRHLLEGE
>Hs-KRT35
NEKETMQSLNDRLAGYLEKVRQLEQENASLESRIREWCEQQVPYMCDYQSYFRTIEELQKKTLCSKAENARLVVEIDNAKLAADDFRTKYETEV
SLRQLVESDINGLRRILDDLTLCKSDLEAQVESLKEELLCLKKNHEEEVNSLRCQLGDR-
LNVEVDAAPPVDLNRVLEEMRCQYETLVENNRRDAEDWLDTQSEELNQQVVSSSEQLQSCQAEIIELRRTVNALEIELQAQHSMRDALESTLAE
TEARYSSQLAQMQCMITNVEAQLAEIRADLERQNQEYQVLLDVRARLECEINTYRGLLESE
>Ea-krt18.8
NPKMEMQDLNTRLAEYVDKVRILEHSNEDLEVKIKQLLINRNAATNDYTHYYDTVKDVQKKLLEKHLENESIVLKIDNAHLAADDFKSKWDTEC
GVRNSVASDITNLRTLLDEYTLARTELETDVEAQKDELAYIKKSHAEDLAELRSHLE---
TTLNVTEMKGDDLTDVLSNLRSQYETFIDKIRDEAEENFRKLVSTVSPQEEQQTKVLSDVKQEQEELRRSYKIFQNDLESLRKTIEPLESEFAN
TQNRKERELANFLRVIGRLEGELNTANDEHDRQLRAYSALLNEKMRLEQEIATYRRLLEDE
>Pm-krt18.1
DEKAEMRGLNERLAEYIAKVHYLEGVNHELEVKIKELLKSGHGKIQGSSSRFTAAEELCAKIKSQIVDNARAGVKIENARLAADDFHYKLETER
SARSCVEEDIARLYALLEEYGVAGAGAEAEIEALSEELHYIRKTHKQDTATLGARLEASSVSVEVASTKGSDLSEILAGLRRQYEAMIVKTHEE
MEFAYKQKMDTVNVTVSEKKQASQLVKDEISDMNHSTQSLQKELDTLWGLIRCLEEQLRSAEALNADSLTGYSSAIGALESELEKLRADTHRQM
HEYSLLLNEKMKLEQEISTYRTLLESG
>Lr-krt18.8
```

DEKAEMQGLNERLAEYIGKVHYLEGVNHELEVKIKELLKSGHDKTQASGSRLAAAEELCAKIKSQTVDNARAGVKIENARLAADDFHYKLETER
SARSCVEEDIARLHALLEEYGAAGAGAKAEIEALSEELHYIRKTHQQDMATLGARLEASSVSVEVASAKGSDLSEILAGLRRQYEAMIVKTHEE
MEFAYKQKMDTVNVTVFEKKQASQLVKDEISDMKHSSQSLQKELDTLWGLIRCLDEQLRSAEALNADSLSGYSGAISTLESELAKQRADIHRQM
HEYSLLLNEKMKLEQEISTYRALLESG
>Ea-krt18.6
SEKEAMQDLNDRLSNYMDKVHRLEVANGELEMKIDEMLKRHGPEISNNNVYFSTIEELKSKILAQVMENASLTLEIDNARLAADDFHTKWQTEV
TLHSSVEGDIGSLGMLLDEYTLSRAGLETDVEFLQDELAYMRKNHEDEVTALRAQITSSGMSVEVDSTPGIDLARALKDMRSQYEAVVAQNNAD
AEATFQKQAEKVKIVVEQRSQSSDAAKADVLESRRSMQTLQVELDTLRGQVNSLEFNLTETEGRKAQELGSYEIVIQRLQQKLQSMKNDLNGKL
GEYSELLNQKMLLEAEIATYRLLLDGN
>Eb-krt18.7
SEKEAMQDLNDRLSNYMDKVRRLEVTNGELEMKIDEMLKRHGPEISNNNVYFSTIEELKSKILAQVMENASLTLEIDNARLAADDFHTKWQTEV
TLHSSVEGDIGSLGMLLDEYTLSRAGLETDVEFLQDELAYMRKNHEDEVTALRAQITSSGMSVEVDATPGIDLARALKDMRNQYEAVVAQNNAD
AEATFQKQVEKVKIVVEQRSQSSDAAKADVLESRRSMQTLQVELDTLRGQVNSLEFNLTETEGRKAQELGSYEIVIQRLQQKLQSMKNDLNGKL
GEYSELLNQKMLLEAEIATYRLLLDGN
>Pm-krt18.7
DEKLEMQDLNTRLEEYMEKVRYLESVNKALEIKIKESRASN-
ISTTNYDPLLVNIEALIEQITAAKLVNAQISLEIDNARLAADDFRTKWETEIVLRQSVEGDIDNLRGLKLDYESESGLLHSQLQLLAEELLYLK
KNHAEEIAALRAQCGAE-
MSVEVDSTPGVDLSKIIAQIRAQYEEMIRKNQEEAEAAFKKQAEMVKATASQGQAALNTVKMESKEARQSLQTLMLELEMLRSTNKSLEDALAD
TENRYGHELQLLQAQLQKLEAEIAQVRTGDNAQLQEYQTLLNAKMKLEMEIATYRRLLEGE
>Lr-krt18.3
DEKLEMQDLNTRLEEYMEKVRYLESVNKALEIKIKESRASN-
ISTTNYDPLLVNIEALIEQITSAKLVNAQISLEIDNARLAADDFRTKWETEIVLRQSVEGDIDNLRGLKLDYESESGLLHSQLQLLTEELLYLK
KNHAEEMAALRAQCGAE-
MSVEVDSAPGVDLSKIIAQIRAQYEEMIRKNQEEAEAAFKKQAEMVKATASQGQAALNTVKMESKEARQSLQTLMLELEMLRSTNKSLEDALAD
TEGRYGHELQLLQAQLQKLEGEIAQVRAGDNAQLQEYQTLLNAKMKLEMEIATYRRLLEGE
>Eb-krt18.4
HEKQAMQNLNERLHIYLQKVQSLENANAQLEIQIRELQKGKAPTSSDYESYYAIIVDLRAKIITQIMSNASISLEIDNARLAADDFKTKWDNEF
ALRTSVEADIDNLHGLHEEYMMASSSMQGELELLQDELNFMKKNHAEEVAALKAQIAGSNMSIEVDAKSGPDIQQMLEDMRRKYEVIMEQNRVE
AEQAFQQQVEQVQQVVVKQNQAAVSAKNEVTEVRQSMQSLQIELGTLQGQIASLENTLDDTKYRKQQELESYLIMLERVERDFENARMEINTKK
HDYAKLLDEKMKLETEIETYRRLLNGG
>Eb-krt18.12
NEKQAMQGLNDRLARYLEKVQSLERSNAEIELKIKELLQGRGPENKDYNHYFEIITDLKNTIVQQIMENAKIGLEIDNARLAADDFKTKWENEN
TLRNSVEADIANLHSLHDEYTLARSDLECDIEGLQEELNFMRKNHEEEVAALKAQIAGSNMSIEVDAKSGPDIQQMLEDMRRKYEVIMQQNRAE
AEQAFQQQAEQVKAVVVKQDQACSTAKNEVVETRRSMQSIQTEMETLRGLCNSLEDQLADINDRKARELEQYLNAVACLENDIATSRSNINEQL
CKYSALLDEKMKLEKEIETYRTLLSGG
>Pm-krt18.5
GEREEMQELNKRLAGYLHKVKELEAANATTEANIQELLRVRGAIVQDHGARFAAIADLRSKMLAQVLENARIGLDVDNARLAADDFRCKWETEV
ALRSSVEADIGNLHMLLDEYTGSRDAMASEAQSMHEELAYMKRNHRERLALSKAQVEGSSVSIQVDSAKGVDLTKMLGDMREQYESLIARSRGQ
AEEAFRKQLESVKVQSFQQDQAASAAKAELAEVRRTMQGLTVELESLMALLGSLEEQLRQTEHDNARELSSHGDQIGALQGRLHSVHGATNAQL
RDYSELLNMKMKLEQEIGTYRRLLEGE
>Ea-krt18.3
KEKQEMQSLNDRLAEYLDNVRFLEKTNEDLEFKIKDILKLKGVTLKDCTAYYSNIEELRKKILTRAQDNAKITLEMDNAKLAAEDFKSKWQMEL
NLRTSVETDIANLRSFLDEYNMERLGMEGEIEALQEELTFMKKNHQEEVAALNAQIENSSMSVEVDSIPGMDLSKTISEIRAQYENMIAKNRED
AENTFRKQVDSHQAVPMQDDLTTQSKKLELQEMRRSMQGLQMELETLHNVVASLKDNLADTENHNVRELSGYASIVTNLESELASMHLDINRQL
KDYSDLLNVKMRLEQEINTYRRLLEGE
>Eb-krt18.2
KEKQEMQSLNDRLAEYLDNVRFLEKTNEDLEFKIKDILKLKGVTLKDCTAYYSNIEELRKKILTRAQDNAKITLEMDNAKLAAEDFKSKWQMEL
NLRTSVETDIANLRSFLDEYNMERLGMEGEIEALQEELTFMKKNHQEEVAALKAQIEKSSMSVEVDSIPGMDLSKTISEIRAQYENMIAKNRED
AENTFRKQVDSHQAVPMQDDLTTQSKKLELQEMRRSMQGLQMELETLHNVVASLKDNLADTENHNVRELSGYANIVTNLESELASMHLDINRQL
KDYSDLLNVKMRLEQEINTYRRLLEGE
>Ea-krt18.2
NEKQEMQTLNDRLAEYLDKVHFLEKANRELEIKIKEILKSKGVTLKDYSTYYSIIEDLRNKILLQILENARVSLEIDNARLAAEDFRNKWQMEL
NLRTSVENDIGGLRSMLDEYNMARLGLEGEIEAMREELVFMKKNHEQEVGALKAQIEDSTMSIEVDSTPGPDLSKTLTEIRAQYENMIAKNRDD
AEVTFRKQVETHQTIIVQQDQATQSIKVELQETRRNLQGLNVELESLKSMIRSLEDTLADTEDRNARDLAFYQNSISRLEAEYASMRCDINRQL
KDYSDLLNVKMKLEKEIATYRSLLEGE
>Eb-krt18.1
NEKQEMQTLNDRLAEYLDKVHFLEKANRELEIKIKDIIKSKGVTLKDYSTYYSIIEDLRNKILLQILENARVSLEIDNARLAAEDFRNKWQMEL
NLRTSVENDIGGLRSMLDEYNMARLGLEGEIEAMREELVFMKKNHEQEVGALKAQIEDSTMSIEVDSTPGPDLSKTLSEIRAQYENMIAKNRDD
AEVTFRKQVETHQTIIVQQDQATQSIKVELQETRRNLQGLNVELESLKSMIRSLEDTLADTEDRNARDLAFYQNSISRLEAEYASMRCDINRQL
KDYSDLLNMKMKLEKEIATYRSLLEGE
>Pm-krt18.3
SEKVEMQGLNERLAEYLDKVKYLESANQAIELKIKEMLQGKGSTAKDYSVYYSTIEDLREKIFVQILENAKISLEIDNARLAADDFRSKWETEA
ALRMSVETDIGNLRGLLDEYGMACMGLEGDIEALREELIFMKKNHENELAALRAQLSGANMSVEVDSTRGQDLHKILDDMRAQYEGLIAVNRAN
AEMAFNKQAESVVVQGAQQSQAALAAQSEMSETRHAMQSLMTELESLRGLIRSLEDQQYDTEDRNARDLASYTVHIQMLEGELGSVRVGINQQL
KDYAELLNMKMKLEQEISTYRRLLEGE
>Lr-krt18.6
SEKVEMQGLNERLAEYLDKVKFLESANQAIELKIKEMLQGKGSIAKDYSVYYSTIEDLREKIFVQILENAKISLEIDNARLAADDFRSKWETEA
ALRMSVEADIGNLRGLLDEYGMACMGLEGDIEAMREELIFMKKNHEDELAALRAQLSGANMSVEVDSTRGQDLHKILDDMRAQYEGIIAVNRAN
AEMAFNKQAEIAMVQGAQQSQAASAAQSEVSETRHAMQSLAAELESLRALIRSLEDQLYDTEDRKARELASYTVHIQMLEGELGSVRMGINQQL
KDYAELLNMKMKLEQEISTYRRLLEGE
>Pm-krt18.4
DEKQELQGLNDRLAGYIEKVRILEQANKEIEIKIKELLKGKGPTIKDYSMYYATIEDLRAKILAQTLENARVSLEIDNARLAADDFRSKWETEL
ALRNSVEVDIANLKCLLDEYNMARMGLEGEIEALREELIFMKKNHEQEVQALRAQIADTSMSVEVDNVKGADLARILADIRAQYEAMIARSRDE
AEEAFRKQLETVKQVSVQQNQVTITAKTELQETRRSMQGLQVELDSMRSMIRSLEDTLADTEDRNARDLSGYQNILARLEAELTSLRCDINRQL
KDYADLLNMKMKLEAEIATYRRLLDGG
>Lr-krt18.9

```
DEKQELQGLNDRLAGYIEKVRILEHANKEIEIKIKELLKGKGPSIKDYSVYYSAIEDLRAKILTQTLENARVSLEIDNARLAADDFRSKWETEL
ALRNSVEVDIANLKCLLDEYNMARMGLEGEIEALREELIFMKKNHEQEVLALRAQIADTSMSVEVDNVKGADLARILADIRSQYEGMIARSRDE
AEEAFRKQLETVKQVSVQQNQVTITVKTELQETRRSMQGLQVELDSMRSMIRSLEDTLADTEDRNARDLSGYQNILARLEAELTSLRCDINRQL
KDYADLLNMKMKLEAEIATYRRLLDGG
>Lr-krt18.2
NEKVEMQGLNDRLAEYIEKVRFLENANQELELRIKELLKGKGPGNKDYSAYYTTMQELREKILAQIMENARVSLEIDNARLAADDFRSKWETEL
ALRSSVEADINNLRGLLDEYSMARMGLEGEIESLREELIFMRKNHEEELAALRAQLEGSSMSVEVDSAKGKDLHKILAEIRAQYEGMIAKNRVD
QEEAFNKQAQSVQVVAVQHSQASQAAKVEVTETRRAMQSLQAELDSLRGLIRSLEDQLQDTEERNARDLSTYTMQIQRLEGELSNLRHGINQQL
KEYADLLNMKMKLEAEIATYRRLLEGE
>Pm-krt18.9
DEKLEMQGLNDRLAGYIEKVRFLEGANQELELKIKELLKGKGGSSKDYSAYYPIIEDLRAKILAQILENARISLEIDNARLAADDFRSKWETEL
ALRSSVEADINNLRGLLDEYNMARMGLEGEIESLREELIFLKKNHEEELAALRAQLEGSSMSVEVDSAKGKDLSKILAEIRAQYEAMIARNRVD
QEEAFKKQVDTVKVASVQQNQAAQSAKNEVVETRRSMQSLQAELDSLRGLIRSLEQQLQDTEERNARELSGYTNVIQRLEGELNNMRGDINRQL
KDYSDLLNMKMKLEEEIATYRRLLEGE
>Pm-krt18.10
DEKLEMQGLNDRLAGYIEKVKFLEGANRELELKIKEMLKGKGGSSKDYSGYYKIMDDLRAKILVQILENARISLEIDNARLAADDFRTKWETEL
ALRSSVEADINNLRGLLDEYNMARMGLEGEIESLREELHFMRKNHEEELAALRAQLEGSSMSVEVDSAKGVDLSKILAEIRAQYEAMIARNRVD
QEEAFKKQVETVKVSSVQQSQAANSAKSEVVETRRAMQSLQAELDSLRGLVRSLDDQLQDTEDRNARELSSYTIIIQRLEGELNNMRGDINRQL
KDYSDLLNMKMKLEAEIATYRRLLEGE
```

Figure S11. Multiple sequence alignment used for the phylogeny of type I keratins of four species of cyclostomes and human in Figure S7. The trimmed amino acid sequences are provided in fast_align format. Species abbreviations: Ea, *Eptatretus atami*; Eb, *Eptatretus burgeri*; Hs, *Homo sapiens*; Lr, *Lethenteron reissneri*; Pm, *Petromyzon marinus*.

>Pm-krt8.2
MEKNQLMGLNDRFANYIQNVRILERKNKELQVSLKRMRIHSGQDSQLDNLCAQYEVLLKRQIQALIEEKRRLANDQEQAHINMEQMKIKYETEI
RERTQIENEFVVLKQEADQVYMQKVELEARLTGLTDDIDFYRKVYEREIRELEARMVKVDVVVEVDSSPGLDLDTYLAEVREQYMKQAGRIREE
LKISFETKLDANRNNEVKINDDMRLVTVEISEVRRNMQRYKAELEALKQQCLALERAIALAEEKGKESMRQLTEKRQLLEVTIMEQKNKLTGHH
RSYQELMNVKLALDMEIIAYRKLLEGEE
>Lr-krt8.10
MEKNQLMGLNDRFANYIQNVRILERKNKELQVSLKRMRIHSGQDSQLDNLCAQYEVLLKRQIQALIEEKRRLANDQEQAHNNMEQMKIKYETEI
RERSQIENEFVVLKQEADQVYMQKVELEARLTGLTDDIDFYRKVYEREIRELEARMVKVDVVVEVDSSPGLDLDAYLAEVREQYMKQAVRIREE
LKISFETKLDANRSNEVKINDDMRLVTIEISEVRRNMQRYKAELEALKQQCLALERAIALAEEKGKDTIRQLTEKRQLLEVTIMEQRSKLTGHH
RSYQELMNVKLALDMEIIAYRKLLEGEE
>Eb-krt8.10
KEKDAIKHLNNRFANLIEKVSTLQQQNKVLEAQWFALQQKVPADVGVEKGFQKYIDVLQQQLNTLHDKKSQLQSHLLQTRESVQANKGRYETEI
DKRTQKDLGLLEIKKDVGIFRFKKTEKECEKKILVDEIDFLKMLFAKEIQDLELQIKDASLFVSVDTNHSLDFEGIISEVRAEYDAITAQSQAN
AQDFYRKKLLDISTSADNANNQLHEAKAEMVKMSLQIKRMKAQLDSLKKQL--

>Ea-krt8.6
QEQASLKSLNNQFAGFIDKVRHLEQQNKVLEAQLQALQHQESQGSRFYKLLECYSEDLRKNNDQVTKENERMQQENEEVQGLVENLRNKFESQR
NSRIEVENRLQDIKRETDSAFVSKADVEANIELTSTDIQFMSQIFCEEEAQLQAKSK--
SVTVEVDTSRSLDLSNIIDDVREQYEANLMKMQQEAEIYYKNKLDDAATMARNISSELENTQGEIDNLNKQLEFVTATTEALKNERVELEAVVI
QAEENGENDVAAARSHCATLEEDIKASKQEMALQVQEHQELMNVKLALDVEIATYRKLLEGEE
>Hs-KRT80
QEKEEMKALNDKFASLIGKVQALEQRNQLLETRWSFLQGQDSAIFDLGHLYEEYQGRLQEELRKVSQERGQLEANLLQVLEKVEEFRIRYEDEI
SKRTDMEFTFVQLKKDLDAECLHRTELETKLKSLESFVELMKTIYEQELKDLAAQVKDVSVTVGMDSRCHIDLSGIVEEVKAQYDAVAARSLEE
AEAYSRSQLEEQAARSAEYGSSLQSSRSEIADLNVRIQKLRSQILSVKSHCLKLEENIKTAEEQGELAFQDAKTKLAQLEAALQQAKQDMARQL
RKYQELMNVKLALDIEIATYRKLVEGEE
>Ea-krt8.2
QEKDALKCLNDRFASFIDKVRYLEHQNRVLETQWQCLQRR-
NVTADADCILGAYTSKLKQEQEGILAERPRLQSELSQTVTLVDELKSKYQKENDLRIDLENEYMRTKSAVDESHISKVAMETRLNELNSQLEFL
RHLFNAERHEMEQRVQDMTVTLELDTGFNFNLESLVDEVRANYGLIASRSRQEVECWYKSKITDMNESTERNCRELRNAKSEIEELRHRVQRFR
CEMDTLQRQRKQMESAIQEAEERGQASVREGKSAITTLEDELAKAKNDMARHVRDYQELMNIKLALDVEIATYRKLLEGEE
>Eb-krt8.8
NEKEGMKCGNDRFASFIDKTRFLEQQNKVLEAQWECLQNK-
TCTSKLDCMFEQFANRLKEQLECLVQDRPRLETEMNQTQSLANDYRSKYEEEIALRTQLENEFWALKQDVDCKYLEKVRLETRLAQLNDEVLFL
RTLFEQELQEMHERIRDMSVSVELDVAPNLDLTSLIAEVRCNYEAIAARSRQEVECWYKSKLDDINEASERNCCELRCTKNEVADLARNIQRLR
CEIETQARQRSQLEATIQDAEARGQASVLQGRETIARLENELQSAKQEMAKHVRDYQELMNVKLALDVEIATYKKLLEGEE
>Pm-krt8.9
QEKNAISHLNDRFASFIDKVRYLEQENKVLEAQWGCLQAR-
VCTSDLDSMFETYAQAIKRQLDCVLADRPRLETELHQTRALADEHKLKYEAEVTGREAAEANFVAIKREADDNYMGKVQMETRVGQLSDELHFI
KELFACEMQEMEERIRDMNVTIDLDTGCNFDLSSLIAEVRANYEMVAARSREEVECWYKSKMDDMAEASERHCIELRCTKNEILELSSMLQRLA
CEIDGLKTQRYQLESSIQEAETRGELNAHEARDATARVEMELSQAKADMARHVRDYQELMNVKLALDIEIATYRKLLEGEE
>Lr-krt8.6
QEKNAISHLNDRFASFIDKVRYLEQENKVLEAQWGCLQAR-
VCTSDLDSMFETYAQAIKRQLDCVLADRPRLETELHQTRALADEHKLKYEAEVTGREAAEANFVAIKREADDNYMGKVQMETRVGQLSDELHFI
KELFACEMQEMEERIRDMNVTIDLDTGCNFDLSSLIAEVRANYEMVAARSREEVECWYKSKMDDMAEASERHCIELRCTKNEILELSSMVQRLA
CEIDGLKTQRCQLESSIQEAEHHGEMNAHEARDATARVETELSQAKADMARHVRDYQELMNVKLALDIEIATYRKLLEGEE
>Lr-krt8.7
QEKNAISHLNDRFASFIDKVRYLEQENKVLEAQWGCLQAR-
VCTSDLDSMFETYAQAIKRQLDCVLADRPRLETELHQTRALADEHKLKYEAEVTGREAAEANFVAIKREADDNYMGKVQMETRVGQLSDELHFI
KELFACEMQEMEERIRDMNVTIDLDTGCNFDLSSLIAEVRANYEMVAARSREEVECWYKSKMDDMAEASERHCIELRCTKNEILELSSIVQRLA
CEIDGLKTQRCQLESSIQEAEHHGEMNAHEARDATARVETELSQAKADMARHVRDYQELMNVKLALDIEIATYRKLLEGEE
>Ea-krt8.5
QEKDALKVLNDRFASFIERVRILEQQNKILQAQDNHFQTSGS-
DTNVGAMFEAFIRSLRKKLDCITGDKPKLESELQQMLAVVDELKVKYEKEYDLRTKAENEFVATKKDVDAAYLEKIGLETRAAQLLDEINFLRE
VFDTEICELQDRTKDTGVQVTISQGLNADLSTLIAEVRAQYEAIAAKSRQEVECWFKGKVDSLNESTERNNAEMRASKSEITEITRQIQRIRAE
MDVQKKQRTQIEQAIKEAEDRGSISVREGKEAIGRLEKELQQSRQEMARHVRDYQELMNVKLALDVEIATYRKLLEGEE
>Eb-krt8.7
QEKDALKVLNDRFASFIERVRILEQQNKILQAQDNHFQTSGS-
DTNVGAMFEAFIRTLRKKLDCITGDKPKLESELQQMLAVVDELKVKYEKEYDLRTKAENEFVATKKDVDVAYLEKIGLETRAAQLLDEINFLR-
---EEICELQDRTKDTGVQVTISQGLNADLSTLIAEVRAQYEAIAAKSRQEVECWFKGKVCG------------
KRDKSEITEITRQIQRIRAEMDVQKKQHTQIEQAIKEAEDRGSISVREGKEAIGRLEKELQQSRQEMARHVRDYQELMNVKLALDVEIATYRKL
LEGEE
>Pm-krt8.7
QEKDQIRGLNDRFANFIDKVRSLEQQRSVLDAQWKVLQAKGEDKSNLEDIYQEYIRGLRRQLEMLQENKEHLQSDVGHMQGVVEEFKNKYETEL
NNRNHAENEFVLIKKDFDDAHLNKVELEARLEGLTDEIDFLRRIYEEELRELHAQMNNISLTVEVDTNRHFNMDDIVASVRSQYEALAQQSRQE
AEDFYRVKFEDINASADKSNEDIRNSKQELNDLLRTIKNLTSEVQRLKQQRGQAERAVAEAEDLGEQALKDAKKRIADLEQELADSRRQMAQHV
RDYQELMNVKLALDIEIATYGKLLEGEE
>Lr-krt8.5
QEKDQIRGLNDRFANFIDKVRSLEQQRSVLEAQWKVLQAKGDDKSNLEDIYQEYIRGLRRQLEVLQENKEHLQSDVGHMQGVVEEFKNKYETEL
NNRNHAENEFVLIKKDFDDAHLNKVELEARLEGLTDEIDFLRRIYEEELRELHAQMNNISLTVEVDTNRHFNMDDIVASVRSQYEALAQQSRQE
AEDFYKVKFEDINASADKSNEDIRNAKQELNDLLRTIKSLTSEVQRLKQQRGQAERAVAEAEDLGEQAVKDAKQRIADLEQELADSRRQMAQHV
RDYQELMNVKLALDIEIATYGKLLEGEE
>Hs-KRT78
QETQEIRTLNNQFASFIDKVRFLEQQNKVLETKWHLLQQQGGSQQGLEPVFEACLDQLRKQLEQLQGERGALDAELKACRDQEEEYKSKYEEEA
HRRATLENDFVVLKK--------------------------------
ELGQLQTQASDTSVVLSMDNNRYLDFSSIITEVRARYEEIARSSKAEAEALYQTKYQELQVSAQLHGDRMQETKVQISQLHQEIQRLQSQTENL
KKQNASLQAAITDAEQRGELALKDAQAKVDELEAALRMAKQNLARLLCEYQELTSTKLSLDVEIATYRRLLEGEE
>Hs-KRT82

DEKEQIKCLNNRFASFINKVRFLEQKNKLLETKWNFMQQQRCCQTNIEPIFEGYISALRRQLDCVSGDRVRLESELCSLQAALEGYKKKYEEEL
SLRPCVENEFVALKKDVDTAFLMKADLETNAEALVQEIDFLKSLYEEEICLLQSQISETSVIVKMDNSRELDVDGIIAEIKAQYDDIASRSKAE
AEAWYQCRYEELRVTAGNHCDNLRNRKNEILEMNKLIQRLQQETENVKAQRCKLEGAIAEAEQQGEAALNDAKCKLAGLEEALQKAKQDMACLL
KEYQEVMNSKLGLDIEIATYRRLLEGEE
>Hs-KRT84
DEKEQIKTLNNKFASFIDKVRFLEQQNKLLETKWSFLQEQKCIRSNLEPLFESYITNLRRQLEVLVSDQARLQAERNHLQDVLEGFKKKYEEEV
VCRANAENEFVALKKDVDAAFMNKSDLEANVDTLTQEIDFLKTLYMEEIQLLQSHISETSVIVKMDNSRDLNLDGIIAEVKAQYEEVARRSRAD
AEAWYQTKYEEMQVTAGQHCDNLRNIRNEINELTRLIQRLKAEIEHAKAQRAKLEAAVAEAEQQGEATLSDAKCKLADLECALQQAKQDMARQL
CEYQELMNAKLGLDIEIATYRRLLEGEE
>Hs-KRT85
EEKEQIKSLNSRFAAFIDKVRFLEQQNKLLETKWQFYQNQRCCESNLEPLFSGYIETLRREAECVEADSGRLASELNHVQEVLEGYKKKYEEEV
ALRATAENEFVVLKKDVDCAYLRKSDLEANVEALVEESSFLRRLYEEEIRVLQAHISDTSVIVKMDNSRDLNMDCIIAEIKAQYDDVASRSRAE
AESWYRSKCEEMKATVIRHGETLRRTKEEINELNRMIQRLTAEIENAKCQRAKLEAAVAEAEQQGEAALSDARCKLAELEGALQKAKQDMACLL
KEYQEVMNSKLGLDIEIATYRRLLEGEE
>Hs-KRT83
EEKEQIKSLNSRFAAFIDKVRFLEQQNKLLETKLQFYQNRECCQSNLEPLFAGYIETLRREAECVEADSGRLASELNHVQEVLEGYKKKYEEEV
ALRATAENEFVALKKDVDCAYLRKSDLEANVEALIQEIDFLRRLYEEEIRILQSHISDTSVVVKLDNSRDLNMDCIVAEIKAQYDDIATRSRAE
AESWYRSKCEEMKATVIRHGETLRRTKEEINELNRMIQRLTAEVENAKCQNSKLEAAVAQSEQQGEAALSDARCKLAELEGALQKAKQDMACLI
REYQEVMNSKLGLDIEIATYRRLLEGEE
>Hs-KRT86
EEKEQIKSLNSRFAAFIDKVRFLEQQNKLLETKLQFYQNRECCQSNLEPLFEGYIETLRREAECVEADSGRLASELNHVQEVLEGYKKKYEEEV
SLRATAENEFVALKKDVDCAYLRKSDLEANVEALIQEIDFLRRLYEEEIRVLQSHISDTSVVVKLDNSRDLNMDCIIAEIKAQYDDIVTRSRAE
AESWYRSKCEEMKATVIRHGETLRRTKEEINELNRMIQRLTAEVENAKCQNSKLEAAVAQSEQQGEAALSDARCKLAELEGALQKAKQDMACLI
REYQEVMNSKLGLDIEIATYRRLLEGEE
>Hs-KRT81
EEKEQIKSLNSRFAAFIDKVRFLEQQNKLLETKLQFYQNRECCQSNLEPLFEGYIETLRREAECVEADSGRLASELNHVQEVLEGYKKKYEEEV
SLRATAENEFVALKKDVDCAYLRKSDLEANVEALIQEIDFLRRLYEEEILILQSHISDTSVVVKLDNSRDLNMDCIIAEIKAQYDDIVTRSRAE
AESWYRSKCEEMKATVIRHGETLRRTKEEINELNRMIQRLTAEVENAKCQNSKLEAAVAQSEQQGEAALSDARCKLAELEGALQKAKQDMACLI
REYQEVMNSKLGLDIEIATYRRLLEGEE
>Hs-KRT77
QEREQIMVLNNKFASFIDKVRFLEQQNQVLQTKWELLQQVNTGTNNLEPLLENYIGDLRRQVDLLSAEQMRQNAEVRSMQDVVEDYKSKYEDEI
NKRTGSENDFVVLKKDVDAAYVSKVDLESRVDTLTGEVNFLKYLFLTELSQVQTHISDTNVILSMDNNRSLDLDSIIDAVRTQYELIAQRSKDE
AEALYQTKYQELQITAGRHGDDLKNSKMEIAELNRTVQRLQAEISNVKKQIEQMQSLISDAEERGEQALQDAWQKLQDLEEALQQSKEELARLL
RDYQAMLGVKLSLDVEIATYRQLLEGEE
>Hs-KRT8
QEKEQIKTLNNKFASFIDKVRFLEQQNKMLETKWSLLQQQKTARSNMDNMFESYINNLRRQLETLGQEKLKLEAELGNMQGLVEDFKNKYEDEI
NKRTEMENEFVLIKKDVDEAYMNKVELESRLEGLTDEINFLRQLYEEEIRELQSQISDTSVVLSMDNSRSLDMDSIIAEVKAQYEDIANRSRAE
AESMYQIKYEELQSLAGKHGDDLRRTKTEISEMNRNISRLQAEIEGLKGQRASLEAAIADAEQRGELAIKDANAKLSELEAALQRAKQDMARQL
REYQELMNVKLALDIEIATYRKLLEGEE
>Hs-KRT7
EESEQIKTLNNKFASFIDKVRFLEQQNKLLETKWTLLQEQKAKSSRLPDIFEAQIAGLRGQLEALQVDGGRLEAELRSMQDVVEDFKNKYEDEI
NHRTAAENEFVVLKKDVDAAYMSKVELEAKVDALNDEINFLRTLNETELTELQSQISDTSVVLSMDNSRSLDLDGIIAEVKAQYEEMAKCSRAE
AEAWYQTKFETLQAQAGKHGDDLRNTRNEISEMNRAIQRLQAEIDNIKNQRAKLEAAIAEAEERGELALKDARAKQEELEAALQRGKQDMARQL
REYQELMSVKLALDIEIATYRKLLEGEE
>Hs-KRT2
QEREQIKTLNNKFASFIDKVRFLEQQNQVLQTKWELLQQMNTRPINLEPIFQGYIDSLKRYLDGLTAERTSQNSELNNMQDLVEDYKKKYEDEI
NKRTAAENDFVTLKKDVDNAYMIKVELQSKVDLLNQEIEFLKVLYDAEISQIHQSVTDTNVILSMDNSRNLDLDSIIAEVKAQYEEIAQRSKEE
AEALYHSKYEELQVTVGRHGDSLKEIKIEISELNRVIQRLQGEIAHVKKQCKNVQDAIADAEQRGEHALKDARNKLNDLEEALQQAKEDLARLL
RDYQELMNVKLALDVEIATYRKLLEGEE
>Hs-KRT4
EEREQIKLLNNKFASFIDKVQFLEQQNKVLETKWNLLQQQTTSSKNLEPLFETYLSVLRKQLDTLGNDKGRLQSELKTMQDSVEDFKTKYEEEI
NKRTAAENDFVVLKKDVDAAYLNKVELEAKVDSLNDEINFLKVLYDAELSQMQTHVSDTSVVLSMDNNRNLDLDSIIAEVRAQYEEIAQRSKAE
AEALYQTKVQQLQISVDQHGDNLKNTKSEIAELNRMIQRLRAEIENIKKQCQTLQVSVADAEQRGENALKDAHSKRVELEAALQQAKEELARML
REYQELMSVKLALDIEIATYRKLLEGEE
>Hs-KRT1
REREQIKSLNNQFASFIDKVRFLEQQNQVLQTKWELLQQVDTRTHNLEPYFESFINNLRRRVDQLKSDQSRLDSELKNMQDMVEDYRNKYEDEI
NKRTNAENEFVTIKKDVDGAYMTKVDLQAKLDNLQQEIDFLTALYQAELSQMQTQISETNVILSMDNNRSLDLDSIIAEVKAQYEDIAQKSKAE
AESLYQSKYEELQITAGRHGDSVRNSKIEISELNRVIQRLRSEIDNVKKQISNLQQSISDAEQRGENALKDAKNKLNDLEDALQQAKEDLARLL
RDYQELMNTKLALDLEIATYRTLLEGEE
>Hs-KRT79
QEREQIKTLNNKFASFIDKVRFLEQQNKVLETKWALLQEQGVTRNNLEPLFEAYLGSMRSTLDRLQSERGRLDSELRNVQDLVEDFKNKYEDEI
NKHTAAENEFVVLKKDVDAAYMGRMDLHGKVGTLTQEIDFLQQLYEMELSQVQTHVSNTNVVLSMDNNRNLDLDSIIAEVKAQYELIAQRSRAE
AEAWYQTKYEELQVTAGKHGDNLRDTKNEIAELTRTIQRLQGEADAAKKQCQQLQTAIAEAEQRGELALKDAQKKLGDLDVALHQAKEDLTRLL
RDYQELMNVKLALDVEIATYRKLLESEE
>Hs-KRT75
EEREQIKTLNNKFASFIDKVRFLEQQNKVLETKWALLQEQGTVRQNLEPLFDSYTSELRRQLESITTERGRLEAELRNMQDVVEDFKVRYEDEI
NKRTAAENEFVALKKDVDAAYMNKVELEAKVKSLPEEINFIHSVFDAELSQLQTQVGDTSVVLSMDNNRNLDLDSIIAEVKAQYEDIANRSRAE
AESWYQTKYEELQVTAGRHGDDLRNTKQEISEMNRMIQRLRAEIDSVKKQCSSLQTAIADAEQRGELALKDARAKLVDLEEALQKAKQDMARLL
REYQELMNIKLALDVEIATYRKLLEGEE
>Hs-KRT5
EEREQIKTLNNKFASFIDKVRFLEQQNKVLDTKWTLLQEQGTVRQNLEPLFEQYINNLRRQLDSIVGERGRLDSELRNMQDLVEDFKNKYEDEI
NKRTTAENEFVMLKKDVDAAYMNKVELEAKVDALMDEINFMKMFFDAELSQMQTHVSDTSVVLSMDNNRNLDLDSIIAEVKAQYEEIANRSRTE
AESWYQTKYEELQQTAGRHGDDLRNTKHEISEMNRMIQRLRAEIDNVKKQCANLQNAIADAEQRGELALKDARNKLAELEEALQKAKQDMARLL
REYQELMNTKLALDVEIATYRKLLEGEE
>Hs-KRT6A
EEREQIKTLNNKFASFIDKVRFLEQQNKVLETKWTLLQEQGTVRQNLEPLFEQYINNLRRQLDSIVGERGRLDSELRGMQDLVEDFKNKYEDEI
NKRTAAENEFVTLKKDVDAAYMNKVELQAKADTLTDEINFLRALYDAELSQMQTHISDTSVVLSMDNNRNLDLDSIIAEVKAQYEEIAQRSRAE

AESWYQTKYEELQVTAGRHGDDLRNTKQEIAEINRMIQRLRSEIDHVKKQCANLQAAIADAEQRGEMALKDAKNKLEGLEDALQKAKQDLARLL
KEYQELMNVKLALDVEIATYRKLLEGEE
>Hs-KRT6B
EEREQIKTLNNKFASFIDKVRFLEQQNKVLDTKWTLLQEQGTVRQNLEPLFEQYINNLRRQLDNIVGERGRLDSELRNMQDLVEDLKNKYEDEI
NKRTAAENEFVTLKKDVDAAYMNKVELQAKADTLTDEINFLRALYDAELSQMQTHISDTSVVLSMDNNRNLDLDSIIAEVKAQYEEIAQRSRAE
AESWYQTKYEELQITAGRHGDDLRNTKQEIAEINRMIQRLRSEIDHVKKQCANLQAAIADAEQRGEMALKDAKNKLEGLEDALQKAKQDLARLL
KEYQELMNVKLALDVEIATYRKLLEGEE
>Hs-KRT6C
EEREQIKTLNNKFASFIDKVRFLEQQNKVLDTKWTLLQEQGTVRQNLEPLFEQYINNLRRQLDSIVGERGRLDSELRNMQDLVEDLKNKYEDEI
NKRTAAENEFVTLKKDVDAAYMNKVELQAKADTLTDEINFLRALYDAELSQMQTHISDTSVVLSMDNNRNLDLDSIIAEVKAQYEEIAQRSRAE
AESWYQTKYEELQVTAGRHGDDLRNTKQEIAEINRMIQRLRSEIDHVKKQCASLQAAIADAEQRGEMALKDAKNKLEGLEDALQKAKQDLARLL
KEYQELMNVKLALDVEIATYRKLLEGEE
>Hs-KRT76
QEREQIKTLNNKFASFIDKVRFLEQQNKVLETKWELLQQQTSGPSSLEPCFESYISFLCKQLDSLLGERGNLEGELKSMQDLVEDFKKKYEDEI
NKRTAAENEFVGLKKDVDAAFMNKVELQAKVDSLTDEVSFLRTLYEMELSQMQSHASDTSVVLSMDNNRCLDLGSIIAEVRAQYEEIAQRSKSE
AEALYQTKLGELQTTAGRHGDDLRNTKSEIMELNRMIQRLRAEIENVKKQNANLQTAIAEAEQRGEMALKDANAKLQDLQTALQKAKDDLARLL
RDYQELMNVKLALDVEIATYRKLLEGEE
>Hs-KRT3
QEREQIKTLNNKFASFIDKVRFLEQQNKVLETKWNLLQQQGSGTNNLEPLFENHINYLRSYLDNILGERGRLDSELKNMEDLVEDFKKKYEDEI
NKRTAAENEFVTLKKDVDSAYMNKVELQAKVDALIDEIDFLRTLYDAELSQMQSHISDTSVVLSMDNNRSLDLDSIIAEVRAQYEDIAQRSKAE
AEALYQTKLGELQTTAGRHGDDLRNTKSEIIELNRMIQRLRAEIEGVKKQNANLQTAIAEAEQHGEMALKDANAKLQELQAALQQAKDDLARLL
RDYQELMNVKLALDVEIATYRKLLEGEE
>Hs-KRT71
QEREQIKALNNKFASFIDKVRFLEQQNQVLETKWELLQQLDNCKNNLEPILEGYISNLRKQLETLSGDRVRLDSELRNVRDVVEDYKKRYEEEI
NKRTAAENEFVLLKKDVDAAYANKVELQAKVESMDQEIKFFRCLFEAEITQIQSHISDMSVILSMDNNRNLDLDSIIDEVRTQYEEIALKSKAE
AEALYQTKFQELQLAAGRHGDDLKNTKGEIPPCTHPINSLAVVPQLHHQPRSWLPAFLLCPASSAVANYS------
AGLPGSLPKAPHTLGPSI----------------VPAYARSW----
>Hs-KRT72
GAKQQVRLLHRQFLVLEIQVRFLEQQNQVLETKWNLLQQLDNCRKNLEPIYEGYISNLQKQLEMLSGDGVRLDSELRNMQDLVEDYKKRYEVEI
NRRTAAENEFVVLKKDVDAAYMNKVELQAKVDSLTDEIKFFKCLYEGEITQIQSHISDTSIVLSMDNNRDLDLDSIIAEVRAQYEEIALKSKAE
AETLYQTKIQELQVTAGQHGDDLKLTKAEISELNRLIQRIRSEIGNVKKQCADLETAIADAEQRGDCALKDARAKLDELEGALHQAKEELARML
REYQELVSLKLALDMEIATYRKLLESEE
>Hs-KRT74
QEREQIKVLNDKFASFIDKVRFLEQQNQVLETKWELLQQLDNCKKNLEPILEGYISNLRKQLETLSGDRVRLDSELRSMRDLVEDYKKRYEVEI
NRRTTAENEFVVLKKDADAAYAVKVELQAKVDSLDKEIKFLKCLYDAEIAQIQTHASETSVILSMDNNRDLDLDSIIAEVRMHYEEIALKSKAE
AEALYQTKIQELQLAASRHGDDLKHTRSEMVELNRLIQRIRCEIGNVKKQRASLETAIADAEQRGDNALKDAQAKLDELEGALHQAKEELARML
REYQELMSLKLALDMEIATYRKLLEGEE
>Hs-KRT73
QEREQIKVLNNKFASFIDKVRFLEQQNQVLETKWELLQQLDNCKNNLEPILEGYISNLRKQLETLSGDRVRLDSELRSVREVVEDYKKRYEEEI
NKRTTAENEFVVLKKDVDAAYTSKVELQAKVDALDGEIKFFKCLYEGETAQIQSHISDTSIILSMDNNRNLDLDSIIAEVRAQYEEIARKSKAE
AEALYQTKFQELQLAAGRHGDDLKHTKNEISELTRLIQRLRSEIESVKKQCANLETAIADAEQRGDCALKDARAKLDELEGALQQAKEELARML
REYQELLSVKLSLDIEIATYRKLLEGEE
>Pm-krt8.8
QEKEEIKMLNNRFANFIDKVRILEQQNKVLEAQWYVLQDKGKVDCNLDELFEVYIEELRRQLQNLGHNGARMNSDLQMIKHAGKDFSSKYEVEV
AAHKKKVEEHELVKKEAVEASFVKIELEAKFQGLIEDIHFLREAFAQELRELEAQIKNTNVLVEIDNRRSLDMDGIISEVRKQYEFIMSQSQAE
AEAIYKQKFDKLRSESDRSDEELRKAKAEINDIKRQIHQINSELDILSKQREKLEAAISEREEGGAKNIGEAKAAVAQLEEALAKAKHDMVKHV
RDFTDLMNIKLALDTEILTYRKLLEGEE
>Lr-krt8.11
QEKEEIKMLNNRFANFIDKVRILEQQNKVLEAQWYVLQDKGKVDCNMDELFEVYIEELRRQLQSLGHNGARMNSDLQMIKHAGKDFSSKYQVEV
ATRKKKVEEHELVKKEAVEASFVKIELEAKIQGLIEDIHFLREAFAQELRELEAQIKNTNVLVEIDNRRSLDMDGIISEVRKQYEFIMSQSQAE
AEAIYKQKFDKLRSESDRSGEELRKAKTEINDIKRQIHQINSELDILKKQREKLEAAISEREEGGAKNIGEAKAAVAKLEEALATAKHDMVKHV
RDFTDLMNIKLALDTEILTYRKLLEGEE
>Pm-krt8.1
QEKEQIKKVNDRFVRFIEKVGILEQQNKVLEVQWLLLQDRGARGSKVEASFQSQIHGLQRQLDTLERDKQRRQGELRLTQGLVEDFKNKYEEDK
SIRNKAENEFVAVKQDFDDSCLVKAELEARLEGLKDEINFLRGIFEKELRDLETQIKDTLVSAGVDTSRSIDVQGMISDVHAQYELIAAKSQAE
ANEFYRKKYDLLSLSADPTDQEIRSNRNEMNNLHRQIQRVRGESDALKKQRASLQAAIAGAVDRGGLSVCEAKERIARLEEELHKDKQQKVQRV
REYQELMNVKLALDIEIATYGRLLEGEE
>Lr-krt8.8
QEKEQMKTLNDRFVSFIEKVGILEQQNKVLRVQWLLLQDRGARGSKVEASFQSQIHGLQRQLDTLERDKQRRQGELRLAQGLVEDFKNKYEEDI
SIRNKAENEFVAVKQDFDDSCLVKAELEARLEGLKDEINFLRGIFEKELRDLEPQIKDTLVSAGLDTSRSIDLQGMISDVRAQYELIAAKSQAE
ANEFYRKKYDVLSLSADPTDQEIRSNRNEINNLHDQIQRVRGESNALKKQRASLQAAIAGAVDRGGLSVRVAKERIARLEEELHKDKRQMVQRV
REYQELMNVKLALDIEIATYRRLLEGEE
>Ea-krt8.4
NEKEQIKGLNDKFATFIGKVQQLEQQKQVLEAQWRALQQRGGASVNVDGMFQSYINSLKQQLEGLGQDKMRLHGELQQMQGLVEDFKSKYEEEI
NNRTQKENEFVVVKKDVDDAYLAKVELEAKLDGLQDEIKFLKDIYAEEISQLEQQIKDTSLFVSVDTTRNLNVADIINESQRHYDDIAENSKRE
ASRFYQEKLNEQVSCAPSGEDDIRKCRSEVNELNQSMQHMKAEIENLKKRRANLEAQIAEAEEQGDVQIAETKVQITELEKDLHKCKDDMAAQL
KEYHALMNVKLALDIEIATYRSLLEQEE
>Eb-krt8.4
NEKEQIKGLNDKFATFIGKVQQLEQQKQVLEAQWRALQQRGGASVNVDGMFQSYINSLKQQLEGLGQDKMRLHGELQQMQGLVEDFKSKYEEEI
NNRTQKENEFVVVKKDVDDAYLAKVELEAKLDGLQDEIKFLKDIYAEEISQLEQQIKDTSLFVSVDTTRNLNVADIINESQRHYDDIAESSKRE
ASRFYQEKLNEQVSCAPSGEDDIRKCRSEINELNQSMQHMKAEIENLKKRRANLEAQIAEAEEQGDVQIAETKAQITELEKDLHKCKDDMAAQL
KEYHALMNVKLALDIEIATYRSLLEQEE
>Ea-krt8.3
QEKEAIKKLNDRFASFIDKVRFLEQQKTVLEAQWKALQERGSASLHLDNLFEPYVALLRQRFDELALGQPRLESELQQMQGAVEDLRCKYEGEI
TTHSQMENEFVVVKKEVDESYLMKVELEGILDGLTEDISFYKEIFAEEMHQLEAQIRDTSLYIEVDTRRNLDIGGLIADVRAQYETIAAKSKAE
AEEFYREKFVSFNASAGKNEEELRLIKHDISESLRQLQRVKAEIEALKKQQAHLETAIAETEERGEMDIREAKSTMSHLENELQCTKQEMAKHV
RDYQELMNVKLALDIEIATYRKLLEGEE
>Eb-krt8.3

```
QEKEAIKKLNDRFASFIDKVRFLEQQKTVLEAQWKALQERGSASLHLDNLFEPYVALLRQRFDELALGQPRLESELQQMQGAVEDLRCKCD---
-IRTCKIFLYNLSLQEVDESYLMKVELEGILDGLTEDISFYKEIF--
EMHQLEAQIRDTSLYIEVDTRRNLDIGGLIADVRAQYETIAAKSKAEAEEFYREKFVSFNASAGKNEEELRLIKHDISESLRQLQRVKAEIEAL
KKQQAHLETAIAETEERGEMDIREAKSTMSHLENELQCTKQEMAKHVRDYQELMNVKLALDIEIATYRKLLEGEE
>Pm-krt8.3
QEKEAIKHLNDRFANFIDKVRFLEQQNKVLEAQWYALQEKTTTGSSVDEMFEAYINGLRRQLDGLGHENGRANGDLGQMQAVVEDFMSKYEAEI
TARIHKENEYVVIKKDVDAANVIKIELEAKMQGLVEDINFLREIFAQELSELEAQIKHTNVLVEIDNSRNLDVDGIIADVRHQYETIAARSRAE
ADAFYQDKFDRLHADSGRNDEEMRVIRTEINDNNRHIHRMRAEIDALKKQRAKLEAAIAEAEGRGEADIREAKAAIAQLEEEIHKAKQEMARHV
REYQELMNVKLALDIEIVTYRKLLEGEE
>Lr-krt8.1
QEKEAIKHLNDRFANFIDKVRFLEQQNKVLEAQWYALQEKTTTGSSIEEMFEAYINGLRRQLDGLGHENGRANGELHQMQAVVEDFMSKYEAEI
TARIHKENEYVVIKKDVDAAHVIKIELEAKMQGLVEDINFLREIFAQELSELEAQIKHTNVLVEIDNSRNLDIDGIIADVRHQYETIAVRSRAE
ADAFYQDKFDRLHSDSGRNDDELRIIRTEINDNNRHIHRMRAEIDALKKQRAKLEAAIAEAEGRGEADIREAKAAIAQLEEEIHKAKQEMARHV
REYQELMNVKLALDIEIVTYRKLLEGEE
>Pm-krt8.5
QEKEAIKHLNDRFANFIDKVRFLEQQNKVLEAQWYALQEKTTTGSSIDEMFEAYINGLRRQLDGLGHDKGQLNGNLGQMQAVVEDFRAKYEAEI
NNRNAAEGEFVVVKKDVDGAYLSKVELETRLQGLVDEINFLRSIFTQELSELEAQIKHTNVLVEIDNSRNLDVDGIIADVRQQYETIAARSRAE
ADAFYQDKFQSLHSDSGRNDEELRMTRNEITELNRQMQRFRAEMEALKKQRAKLESAIAEAEGRGEADIREAKAAIAQLEEEIHKAKQEMARHV
REYQELMNVKLALDIEIVTYRKLLEGEE
>Lr-krt8.4
QEKEAIKHLNDRFANFIDKVRFLEQQNKVLEAQWYALQEKTTTGSSIDEMFEAYINGLRRQLDGLGHDKGQLNGNLGQMQAVVEDFRAKYEAEI
NNRNAAEGEFVVVKKDVDGAYLSKVELETRLQGLVDEINFLRSIFTQELSELEAQIKHTNVLVEIDNSRNLDIDGIIADVRHQYETIAARSRAE
ADAFYQDKFQSLHSDSGRNDEELRMTKTEITELNRQMQRFRAEMEALKKQRAKLESAIAEAEGRGEADIREAKAAIAQLEEEIHKAKQEMARHV
REYQELMNVKLALDIEIVTYRKLLEGEE
>Pm-krt8.4
QEKEAIKHLNDRFANFIDKVRFLEQQNKVLEAQWYALQEKTTTGSSVDEMFEAYINGLRRQLDGLGHENGRANGDLGQMQAVVEDFRAKYEAEI
SLRTEREDEFVFAKKDVDGAYLSKVELEAKLDGLQEDINFMRSIFAQELSELEAQIKHTNVLVEIDNSRNLDVDGIIADVRHQYETIAARSRAE
ADAFYQDKFQSLHSDSGRNDEELRVTRNEITELNRQMQRFRAEMEALKKQRAKLESAIAEAEGRGEADIREAKAAIAQLEEEIHKAKQEMARHV
REYQELMNVKLALDIEIVTYRKLLEGEE
>Lr-krt8.2
QEKEAIKHLNDRFANFIDKVRFLEQQNKVLEAQWYALQEKTTTGSSVEEMFEAYINGLRRQLDGLGHDKGQLNGNLGQMQAVVEDFRAKYEAEI
SMRTEREGEFVFAKKDVDGAYLSKVELEAKLDGLQEDINFLRSMFAQELSELEAQIKHTNVLVEIDNSRNLDIDSIIADVRHQYETIAARSRAE
ADAFYQDKFQSLHSDSGRNDEELRMTKNEITELNRQMQRFRAEMEALKKQRAKLESAIAEAEGRGEADIREAKAAIAQLEEEIHKAKQEMARHV
REYQELMNVKLALDIEIVTYRKLLEGEE
>Ea-krt8.1
QEKEQIKTLNNRFASLIDKVRYLEQHNKVLDAQWRTLQERSTATSNVDAMFEAYINGLKNQLEGLGTDKLRLNGELQQMQALVEDFKVKYEQEI
TNRTQVENEFVVVKKDVDDAYLAKVELEAKLEGLQDEINFLKEVFSEELRQLEAQIRDTSLYVEVDTRRNLDINGLIADVRAQYDTIAAKSRAD
AEDFYKVKFADLTSMTGKTDDEMRMMKGEMNDLNRQIQRINAEIAALKKQRAQLEAAIADAEGRGEVDIREAKETIARLENDLQLAKQEMAKHV
REYQELMNVKLALDIEIATYRKLLEGEE
>Eb-krt8.1
QEKEQIKTLNNRFASLIDKVRYLEQHNKVLDAQWRTLQERSTATSNVDAMFEAYINGLKNQLEGLGTDKLRLNGELQQMQALVEDFKVKYEQEI
TNRTQVENEFVVVKKDVDDAYLAKVELEAKLEGLQDEINFLKEVFSEELRQLEAQIRDTSLYVEVDTRRNLDINGLIADVRAQYDAIAAKSRAD
AEDFYKVKFADLTSMTGKTDDEMRLMKGEMNDLNRQIQRINAEIAALKKQRAQLEAAIADAEGRGEVDIREAKETIARLENDLQLAKQEMAKHV
REYQELMNVKLALDIEIATYRKLLEGEE
>Pm-krt8.10
QEKEQIKGLNDRFANFIEKVRFLEQQNKVLEAQWRALQERGSGGSNLDSLFEAYINGLKQHLDGLGNEKQRLQGELQHMQGAVEDFKAKYEEEI
NTRTQRENEFVVVKKEVDDAFLNKVELEARLDGLNDEIEFLKKIFAEELRQLESQIKDTSLFVTVDTSRNLDVDGLIADVRAQYEAMARKSQAE
ADDFYKKKISELSSSAGKGDDDVRNSRNEINELNRQMQRIKAEIEALKKQRAQLEAAISEAEGRGEMSIKEAKETIARLEEELHKAKQQMAQHV
REYQELMNVKLALDIEIATYRKLLEGEE
>Lr-krt8.9
QEKEQIKGLNDRFANFIDKVRFLEQQNKVLEAQWRALQERGSGGSNLDSLFEAYINGLKQHLDGLGNEKQRLQGELQHMQGAVEDFKAKYEEEI
NTRTQRENEFVVVKKEVDDAFLNKVELEARLDGLNDEIEFLKKIFAEELRQLESQIKDTSLFVSVDTSRNLDVDGLIADVRAQYEAMARKSQAE
ADDFYKKKITELSSSAGKGDDDVRNSRNEINELNRQMQRIKAEIEALKKQRAQLEAAISEAEGRGEMSIKEAKETIARLEEELHKAKQQMAQHV
REYQELMNVKLALDIEIATYRKLLEGEE
>Pm-krt8.6
QEKDQIKGLNDRFANFIDKVRFLEQQNKVLEAQWRALHERGTVVSGLDGLFDAYTRGLKQQLESLSNNKLGLQNELQAMQGHVEDFKTKYEEEI
NTRTERENEFVVVKKDVDDAFLNKVELEAKLEALQDEIEFLKKIFAEELRQLESQIQDTILTVEIDTSRNLDVDGLIADVRAQYEAIAAKSKAE
SDAFYKSKFADLNAMSGKGDDELRQSRSEIADINRQVQRIKAEIEALKKQRAQLEAAIAEAEGRGELAIKEAKESIARLEEELHQAKQDMAKHV
REYQELMNVKLALDIEIATYRKLLEGEE
>Lr-krt8.3
QEKDQIKGLNDRFANFIDKVRFLEQQNKVLEAQWRALHERGTVVSGLDGLFDAYIRGLKQQLESLSNNKLGLQNELQAMQGHVEDFKTKYEEEI
NTRTERENEFVVVKKDVDDAFLNKVELEAKLEALQDEIEFLKKIFSEELRQLESQIQDTILTVEIDTSRNLDVDGLIADVRAQYEAIAAKSKAE
SDAFYRSKFADLNAMSGKGDDDLRQSRSEIADINRQVQRIKAEIEALKKQRAQLEAAIAEAEGRGELAIKEAKESIARLEEELHQAKQDMAKHV
REYQELMNVKLALDIEIATYRKLLEGEE
```

Figure S12. Multiple sequence alignment used for the phylogeny of type II keratins of four species of cyclostomes and human in Figure S8. The trimmed amino acid sequences are provided in fast_align format. Species abbreviations: Ea*,* *Eptatretus atami*; Eb, *Eptatretus burgeri*; Hs, *Homo sapiens*; Lr, *Lethenteron reissneri*; Pm, *Petromyzon marinus*.

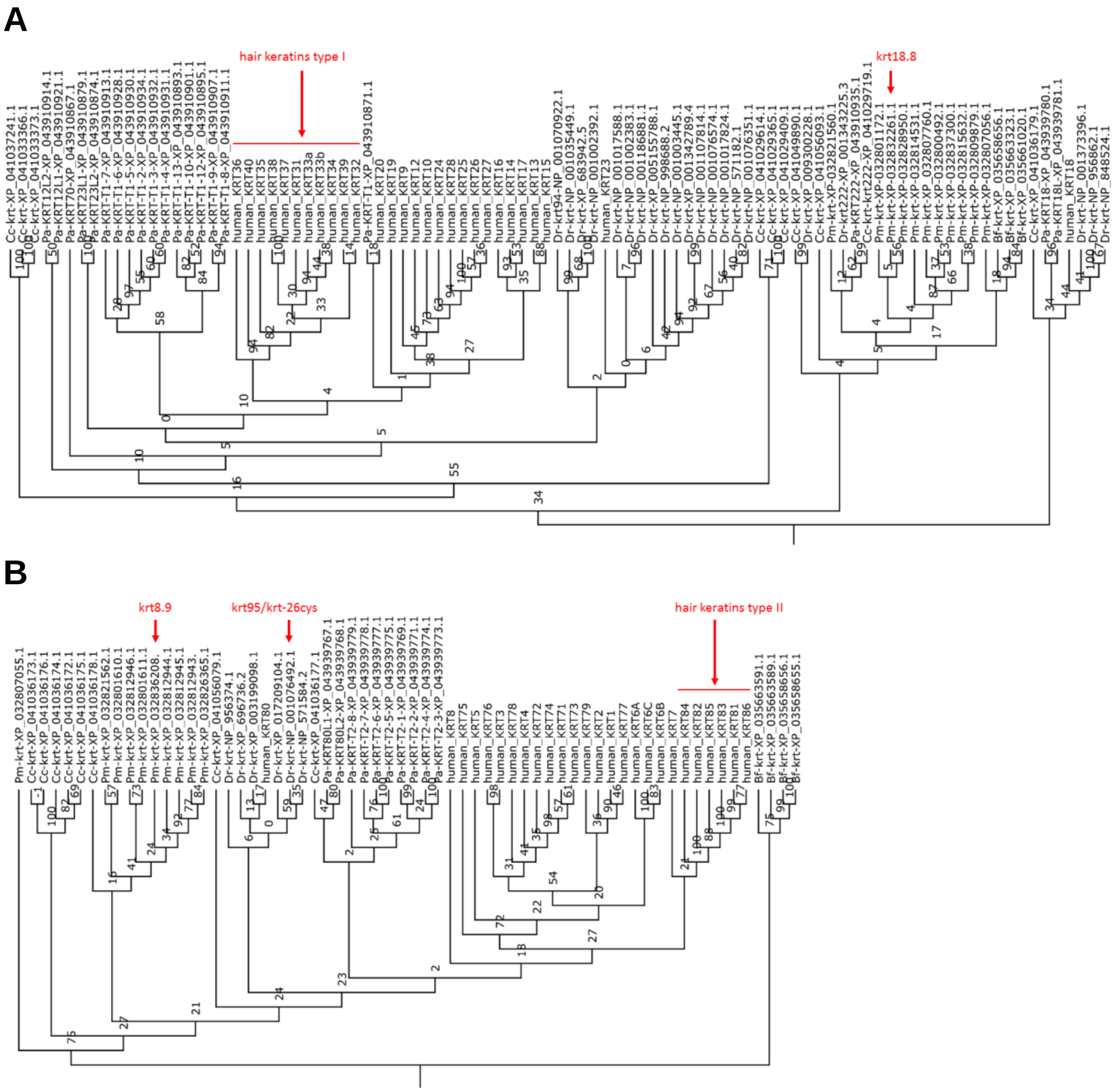

Figure S13. Maximum likelihood analysis of keratins from phylogenetically diverse species of chordates. Phylogenetic trees of type I (A) and type II (B) keratins were inferred using the maximum likelihood method. Bootstrap values are shown above the nodes of the trees. Cysteine-rich keratins are highlighted by red labeling. Note that krt95, which is an as-yet uncharacterized keratin containing 26 cysteine residues (Ehrlich et al. 2020), is not orthologous to the cysteine-rich keratins of the sea lamprey and humans. Species: lancelet (Bf, *Branchiostoma floridae*, *Cephalochordata*), sea lamprey (Pm, *Petromyzon marinus*, *Cyclostomata*), great white shark (Cc, *Carcharodon carcharias*, *Chondrichthyes*), zebrafish (Dr, *Danio rerio*, *Actinopterygii*), lungfish (Pa, *Protopterus annectens*, *Dipnoi*), human (*Homo sapiens*).

>Pm-krt-XP-032801172.1
DAEERRSESEEKLESYMSRVGALRTSCKEDVELLVELIEDLTA-
NPVAWDSAKRKLQEATKQVLPLEIQNARLSLAVQQTQSSADDYKVRMEGECLLRVDVERRLADVHRWVDDFSSSRPELESKVERLREKLSTLQR
EHLQAQAVILSQLLSRDLLSVDPSE-
ALGEALRAGLAYVKSHASGTTTTTQLSVEDRLRQRIEQLNRQSLSSQSAPQSSSQSSSQSSSQPQSSSSSQSAPQSSSQSSSQSSSSSSSAAAP
LIAATEAASSVLEAELRAVRAQAATELREGDLALRLRSLLLREAAAFRRQLQPL
>Bf-krt-XP_035658656.1
NSRQELNTLNTRLSGYINKVRSLEARNAELEMLIAEASKAHAA-
STAPISIDTSELMAMKKEIQATANEVSYLESQKSTLLIEIENLRRQLEDLLRRRAEMESDISGAKGELDSALGALDGLQDEVAQAEDEMSSAAQ
GPAQATTI------EEQIDVVQVER--
LRADLNDSLNALRGEFEQLASQGIADVEGDWEARLRELQAEIERLKALLEELRMQISQLRAELSAILSEAEQIKATNISLERDISTLKDTIRKR
LEDYRIQIADLNGEGQRLDGEIQAVVAQYNDLLNKKLALEKEVIDYEQIVGGE
>Bf-krt-XP_035663323.1
EEKPTMRGLNDRLSTYLARVRALEQANAALQEQINAASGLGG--
SDADAYDWQPDLDAAREALLKANLERARVEIERDSYALEVEQWRGKLEREMDLRADLEADINALKREMDEANMAKVDLEGQIEGAKSELEFMKQ
VHEQEVKELRDRIQGAGAVDIQMGQ-
SSSQDLVAALKAIREEYEEIARKNKEDVERQFEKKAETVKQEASQNVEAASVAKSEVKEMKSQVQGLMAELEALKAMQRSLEEQIADAEMNNAQ
ALESKQLILEQLKQEIARMKGEMSSTLKSYNDMMKTKLALEEEISIYNNLLQGE
>Bf-krt-XP_035661020.1
NEKIEMQSLNNRLGSYITKVRALEEANRNLQIQINEASSVQV--
VSDDGIDWAAELAAAREALLAANLERARVEIERDSVLLEVNQWQDKISGVMEQRAILDDEMNMLRRESEELTMEKVDLEGQIEGAKGELEFLKQ
LHAQEVTDLKGRIAASG--
SIEIKSDFQAVDLNAALKAIREQYDEIAAKNRAEVEASFKGKVAEVKVQQEQSSEALTLVKTEITETRRTVTALTAELESLKAQSFAIQESIAS
AERDNASRLESKMEIINSIKMELGRLKSEMSSMLKQYEDLIKLKLALETEIAQYNTLLSGE
>Dr-krt222-XP_001343225.3
EPKVALRGLNERLKGFLDHVNQLEKANLELEELIGEWGIRNLA-
PPRDWSDKEALAQELRAQIRIILMENAELLIQSDAVNLKAAHLKNRCETEENLRLLQEQDVLQLKTKKNEVETTNRLLEKQILETEHELQQTIE
EHERELDQHQQRGMDLSRTSTQCDHSSPAQSSPDSATPPTQASGSGPAPKRRPRKDAVSPTV-
VFPPQVKASDGALKEARAELAEARKRWHRLQVEIESLHALERSLQSSLRHTQLQYSVQLRDLSRSVKGLESELETVRDGLEVQKESHNQLLNTK
MRLEKEIATYRKLLEHE
>Pa-KRT222-XP_043910935.1

MELSQLLNEIRTHYEEFITSNRIEVNPLSRAQLEEEARRMSKEEEALKMARAELNEARRQWQSLQVEIESLYALEKGLKNSLQATEDHYKAELQ
SLAAIIKELEEELHDVRQNIEKQLTEHQTLLNTNVRLEQEITRYRRILEKE
>Cc-krt-krt222-XP_041029719.1
MKKAQMK---LDMEND--------------
---KATVFGKPVDLVCALQALPACSYPVS----------------------------------AMEDGS-
SRGLELSQLLTDIKKHYEKIIHRSRMELDACISTQLLEVSAEMNEDEDSLQAARAELNDARRQWQSLQVEIESLLALQKGLENSLQVTEQRYEL
QLKGLASVIHELEGELQEVRRDIEHQVWEHELLLNTKMRLEREIAMYRSLLDRE
>Pa-KRT-T1-XP_043910871.1
DQQAMMQNLNERLEDFLGKVRNLEAENVMLEHQIDEWNQKNEP-VKKDYSKYDKEIEELIDMVCQKTVENAQLAYSIESAQIEAYNYQ------
---NSDVES-------------------------
--GAKVSYSFHFAIPEAEVA--------------

>Pm-krt-XP-032807056.1
NEKEELQGLNNRLSGYLQRVRTLEQDNNKIELEIAELMSLRKG-
GPHDWAKWDGIFRALREEIFMLTMDTTKLELGIQNTDLARSDYYEKWETEHAIRMAVEADIQALRKMIDDTNVARLNLESRVEALKEELSVLRY
THAEEVKKLLVEISSFEVGSVEVDSGTKDIDLNKALAEIRRQYEDLCRQNAVQVESSYLETITITNTKVTEKMQALEKYRVELNELRRTTQVLF
LDLENMRSKKLSLENANADMSARYEMEVSSLRDMVIMLDKILVDVNMDLERQRSEYEMLTNAKMRLEQEIAYYSSLLDQE
>Cc-krt-XP_041037241.1
NEKQAMIDLNSRLSIYLQKVRDLEEANTKLELQLKEFQGGKAI-
TGIDYDAYDAVIKPLREQILALHLGNARLALDLDNAALAAKDFKNKFENELYIKQSVEADITDLGAMKDEYIRNYKDLESEIAASKDELAYLKK
NHEEELAGLRQQV-TGTV-SVAVEP-
GPTIDLAKQLQTMRDNYESFSKKTQEDLDNWYKLQVESQTVQTIQVNEAAAGAKLEVTELRKQLQPLEIEYNSLLSGNASLEACIQDINDKYYQ
KLQTLQMTISKMELDLTNIRNEMQQKVKDYDDLLNVKMKLETEIANYKKLLDGS
>Cc-krt-XP_041033366.1
NEKDTMQNLNQRLSSYLDKVRSLELANSTLEKQIQELVSSRTI-
DYFDWSIYKTTVKPLQQQIISSIQLNTRISLEVDNARLAAEDFKNKWESELILRQSVESDINGLHQLKDTYLQLQTDLAHDVAGLEDEIAYLTR
NHNEDLKLLRQQK-TRDI-QVEVDA-
APSVDLAAALEAMRENYTKIADTNQKDLDDWYKQQVQIQVTQTTQSTQALDASKGELIQLRQQMQTLDAEYNSLRGTISALQNALNNTDARYDM
ELRNLLARSTQLEGELGNLLNSITQQSQEYENLLNIKMKLEAEIQKYKTLLEGG
>Cc-krt-XP_041033373.1
NEKDAMQNLNQRLAGYLENVRSLEKSNKELEKEILSY-GTKSI-
EGFDWSVYDKIVRPIQQQILDAIMQNSRIALEIDNAKLAAEDFKNKWESELILRQSVESDINGLHQLKDTYLQLQSDLAHDIAGLEDEIAYLKK

```
NHNEELKLLQQQK-TQDI-QVEIDA-
GPSVDLTAALQDMRENYTKIAEKNQKDLDDWYKQQVQIQVTQTTQSTQALDASKGELTQLRQQIQNLDGEYNALLGTISALQNALNNTDARYDM
ELQNLLTRSTQLEGELGNLLNSITQQGQEYENLLNIKMKLEAEIQKYKTLLEGG
>Pm-krt-XP-032832261.1
NEKEALQDLNERLAGYLDKVRYLEQANQELECKIKEFRANSCI-
AEQQWEPCMGNIEELMGQVEVATLENTRLVLEIDNARLASEDFRVKWEAESALRETVEADAHELRRLSAEYCACRNQLACEQNVLQGEIHTLKS
HHRGEMESLRMDYSSSTT-KVEMDN-
SPGADTAGIISEIRAQYETMIHNNRHEAECMLQSKLEAAEASAVHSHGELAAAKNQAHHLRQQYQTMEVEMESLRSANATLEDNLAETEERFSV
EVRSLAEVLSRLEAEYSDVRANVERQLHEYESLLNIKMGLEMEISTYKCLIEGE
>Pm-krt-XP-032821560.1
DEKAEMQGLNGRLATYIERVRSLDEANRRTELQIKELVEKRPS-
GAVELRQYHEAARELREQILKATMTNARLHVELDNGRLAAEDFRAKLESEVSIHTSVESDITNLRRAIDETNVTRMSLEGQVEQLEEQILHMQK
SHADEKSSLLKEIEESSI-SVEVDS-
VKGHNLNDIIAEIRTQYEALIKSNLQEMEVWYKSKVDAIHPRISQNSEELGTLRQQLSEQRRAMQALYAEAETLRSTVSSLNEAAQDVEAHSAE
GLAGLAGSISRLLSELGSARGDIDRQLREHETLLNTKMRLEEEIETYRRLLEGQ
>Cc-krt-XP_041049890.1
SEKETMQGLNSRLAIYLEKVRSLETSNRDLELKIKALLEERKP-
VDRDIKPMLAQAHALNKQIQDLTMNNAVIMLQMDNAGLSAEDFRMKMESEALVRQSVESDIERLRRVKEEYDHNAITLRSEGEMMTEELLFLKK
NHQEEVNTLRSQITNNQV-TVEMDC-
VDEPDLTKLLAEIRKEYEDMIQKNKMEAELWYKSKLETITTDVNHTNKELDIAKEELNSKRQAVQTLEMELDIIRRQNLGLENILSDTEQRHEM
EFNKLQITISKLEGDLVNIRNEMLQHKERYETLLKTKLTLEAEVAEYRRLLNGE
>Dr-krt-XP_009300228.1
SEKETMQVLNDRLASYLEKVRSLEKSNADLELKIKQFMTERAP-
KGHDIEGLMAQAHAIGQEVRKKTLENARIMLEIDNAKLAAEDFRVKWEAEATLCQSVERDCHALRRAKSDHDQFITTLRGDLDSLKEELYFLKK
NHEEEKNALKSRLSNEQV-NVEVDA-
AQGPDLGAVMAELRVQYENIARKNKEDAEIWYLKKLEAVQSEVKESNEALRCAHSELNERRRFLQALEVELDSLRKQIGVLEGNLEETNQKYTL
EMERLQSSLTQLEDELSQLRLDMQRIKTDYEQLLRIKQNLELEIATYRRLLDGE
>human_KRT23
-GKATMQNLNDRLASYLEKVRALEEANMKLESRILKWHQQRDP-
SKKDYSQYEENITHLQEQIVDGKMTNAQIILLIDNARMAVDDFNLKYENEHSFKKDLEIEVEGLRRTLDNLTIVTTDLEQEVEGMRKELILMKK
HHEQEMEKHHVPS-DFNV-NVKVDT-GPREDLIKVLEDMRQEYELIIKKKHRDLDT-YKEQSAAMSQEAA-
SPATVQSRQGDIHELKRTFQALEIDLQTQYSTKSALENMLSETQSRYSCKLQDMQEIISHYEEELTQLRHELERQNNEYQVLLGIKTHLEKEIT
TYRRLLEGE
>Pm-krt-XP_032807760.1
DEKAEMRGLNERLAEYIAKVHYLEGVNHELEVKIKELLKSGHG-
KIQGSSSRFTAAEELCAKIKSQIVDNARAGVKIENARLAADDFHYKLETERSARSCVEEDIARLYALLEEYGVAGAGAEAEIEALSEELHYIRK
THKQDTATLGARLEASSV-SVEVAS-
TKGSDLSEILAGLRRQYEAMIVKTHEEMEFAYKQKMDTVNVTVSEKKQASQLVKDEISDMNHSTQSLQKELDTLWGLIRCLEEQLRSAEALNAD
SLTGYSSAIGALESELEKLRADTHRQMHEYSLLLNEKMKLEQEISTYRTLLESG
>Pm-krt-XP-032828950.1
DEKLEMQDLNTRLEEYMEKVRYLESVNKALEIKIKESRASNI--
STTNYDPLLVNIEALIEQITAAKLVNAQISLEIDNARLAADDFRTKWETEIVLRQSVEGDIDNLRGLKLDYESESGLLHSQLQLLAEELLYLKK
NHAEEIAALRAQC-GAEM-SVEVDS-
TPGVDLSKIIAQIRAQYEEMIRKNQEEAEAAFKKQAEMVKATASQGQAALNTVKMESKEARQSLQTLMLELEMLRSTNKSLEDALADTENRYGH
ELQLLQAQLQKLEAEIAQVRTGDNAQLQEYQTLLNAKMKLEMEIATYRRLLEGE
>Pm-krt-XP-032815632.1
GEREEMQELNKRLAGYLHKVKELEAANATTEANIQELLRVRGA-
IVQDHGARFAAIADLRSKMLAQVLENARIGLDVDNARLAADDFRCKWETEVALRSSVEADIGNLHMLLDEYTGSRDAMASEAQSMHEELAYMKR
NHRERLALSKAQVEGSSV-SIQVDS-
AKGVDLTKMLGDMREQYESLIARSRGQAEEAFRKQLESVKVQSFQQDQAASAAKAELAEVRRTMQGLTVELESLMALLGSLEEQLRQTEHDNAR
ELSSHGDQIGALQGRLHSVHGATNAQLRDYSELLNMKMKLEQEIGTYRRLLEGE
>Pm-krt-XP-032809879.1
SEKVEMQGLNERLAEYLDKVKYLESANQAIELKIKEMLQGKGS-
TAKDYSVYYSTIEDLREKIFVQILENAKISLEIDNARLAADDFRSKWETEAALRMSVETDIGNLRGLLDEYGMACMGLEGDIEALREELIFMKK
NHENELAALRAQLSGANM-SVEVDS-
TRGQDLHKILDDMRAQYEGLIAVNRANAEMAFNKQAESVVVQGAQQSQAALAAQSEMSETRHAMQSLMTELESLRGLIRSLEDQQYDTEDRNAR
DLASYTVHIQMLEGELGSVRVGINQQLKDYAELLNMKMKLEQEISTYRRLLEGE
>Pm-krt-XP-032814531.1
DEKQELQGLNDRLAGYIEKVRILEQANKEIEIKIKELLKGKGP-
TIKDYSMYYATIEDLRAKILAQTLENARVSLEIDNARLAADDFRSKWETELALRNSVEVDIANLKCLLDEYNMARMGLEGEIEALREELIFMKK
NHEQEVQALRAQIADTSM-SVEVDN-
VKGADLARILADIRAQYEAMIARSRDEAEEAFRKQLETVKQVSVQQNQVTITAKTELQETRRSMQGLQVELDSMRSMIRSLEDTLADTEDRNAR
DLSGYQNILARLEAELTSLRCDINRQLKDYADLLNMKMKLEAEIATYRRLLDGG
>Pm-krt-XP-032837300.1
DEKLEMQGLNDRLAGYIEKVRFLEGANQELELKIKELLKGKGG-
```

```
SSKDYSAYYPIIEDLRAKILAQILENARISLEIDNARLAADDFRSKWETELALRSSVEADINNLRGLLDEYNMARMGLEGEIESLREELIFLKK
NHEEELAALRAQLEGSSM-SVEVDS-
AKGKDLSKILAEIRAQYEAMIARNRVDQEEAFKKQVDTVKVASVQQNQAAQSAKNEVVETRRSMQSLQAELDSLRGLIRSLEQQLQDTEERNAR
ELSGYTNVIQRLEGELNNMRGDINRQLKDYSDLLNMKMKLEEEIATYRRLLEGE
>Pm-krt-XP-032800492.1
DEKLEMQGLNDRLAGYIEKVKFLEGANRELELKIKEMLKGKGG-
SSKDYSGYYKIMDDLRAKILVQILENARISLEIDNARLAADDFRTKWETELALRSSVEADINNLRGLLDEYNMARMGLEGEIESLREELHFMRK
NHEEELAALRAQLEGSSM-SVEVDS-
AKGVDLSKILAEIRAQYEAMIARNRVDQEEAFKKQVETVKVSSVQQSQAANSAKSEVVETRRAMQSLQAELDSLRGLVRSLDDQLQDTEDRNAR
ELSSYTIIIQRLEGELNNMRGDINRQLKDYSDLLNMKMKLEAEIATYRRLLEGE
>Cc-krt-XP_041056093.1
NQQDELQGLNKRLANYLNKVKTLESANKELEGKIQNYLAKRGT-
LARDWSSYEKPVLDICKQVQETNLDNAGLTLQLDNCELASDDFKVKWQSESELKQSVEQDLNGLRKILDDTNVGRMQLESQIEAMNEELIYLRK
NHQEDVNELKMQIANSSV-SVEVEH-
SDDEDLVKVMNQIREEYQALADQNRKDAEECYRKKFDAASFEASKNNEVLQAMKNDLTSLRRQIKNLEVEHRSLVAMINSLEDTLDDTEDRTAA
ELQVLNQNIQNLEQQLADLHKKLKAQNVQYQILLNTKMRLQAEINTYRGLLDSE
>Dr-krt-NP_001373396.1
NEKVTMQILNERLASYLEKVRILEKANGDLEVKIRQVMEKKGP-
DTTDYSHFQVTLDKLRKEILEMIMRNVNVNLQIDNAKLAAEDFKIKFENEFQQRQCVEADINALRKMLDDTNVARLHLENDVEGLKVELIEIKK
QHQLELSALNGQITQSGV-QVDINA-
PKGQDLAKIMEEMRANYEKIVLKNQEELKAWHESKISVVQVQVTENTAALKEASTQLSVSRRRMQTLETDLQTLTGSRASLEEALNETKLRYGM
QVEQFNNIILLRESELKQLRDDIQRQSLDYQVLFNIKMELEAEIATYKRLLDGE
>human_KRT18
-EKETMQSLNDRLASYLDRVRSLETENRRLESKIREHLEKKGP-
QVRDWSHYFKIIEDLRAQIFANTVDNARIVLQIDNARLAADDFRVKYETELAMRQSVENDIHGLRKVIDDTNITRLQLETEIEALKEELLFMKK
NHEEEVKGLQAQIASSGL-TVEVDA-PKSQDLAKIMADIRAQYDELARKNREELDKW-
SQQIEESTTVVTTQSAEVGAAETTLTELRRTVQSLEIDLDSMRNLKASLENSLREVEARYALQMEQLNGILLHLESELAQTRAEGQRQAQEYEA
LLNIKVKLEAEIATYRRLLEDG
>Cc-krt-XP_041036179.1
NQKETMQDLNDRLASYLEKVRSLETGNTNLELQIKEHLDARGP-
SVRDWSLYEKPLNELRKEVYDMTVDNARLILQIDNARLAADDFRVKWESELSIRQSVENDINGLRKVIDDTNIGRLHLEGEIESLKEELIYIRK
NHEEEVKALRSQITESSV-HVEVDS-
PQGPDLAKVIADIRKQYEEVAQKNKEDAENWYKNQMDSYKVEVQHNTDELRSAKGQVTELHRQIQSLEVELESLLSMKNSLEGTLKDTELRYEM
ELQSLNGMISKLEGDLFQIRSDMQSQVREYEILLNIKMKLEAEIATYRRLLDGE
>Pa-KRT18-XP_043939780.1
NEKETMQELNDRLANYLETVRRLENANKQLEIQIREAMEKRGP-
SIRDYSNYEKIIKELRDQIYDTTVDSARLVLAIDNARLAADDFRVKWEAELAIRQSVESDINGLRKVIDDTNMGRLQLESEIESLKEELIFIKK
NHEDEVMALRNQVNSCGV-QVDVDA-
PKGTDLAEIMASLRAEYEALMSRNKDDAERWYQSKVETFQVETSQNTEALQTAKTELSDLRRRIQSLEIELESNRSMKASLEDTLRDTELRYAM
EMEKLGALIGRIEAELAQVRTDMQRQAQDYEVLLNAKMKLEVEIATYRRLLEGQ
>Pa-KRT18L-XP_043939781.1
NEKETMQELNDRLANYLETVRRLENANKQLEIQIREEMEKRGP-
SIRDYSNYEKSIKELRDQVYDTAVDNVRLALAIDNDRIAADDFKVKYETEVTIRQCVELDIDGLRKVIDDNTVIRLMLEDEIESLKEERILLKK
SHEAEVAALRDRANGCGV-QVDVDA-
PKGTGMSEIMATLQAEYAALMSKSKEDRDHWYAIKAESFQVETSYNKESLQTAKTELSDLRCKIQSRQLELESNISMKALLEGTLRDTELHYNM
EMERLGALISRIEAELSQFRSDMQRQAQDYEALLNAKMKLEEEIATYRCLLKGD
>Dr-krt-NP_848524.1
NEKMAMQNLNDRLASYLEKVRILEQANSKLELKIREALEKRGP-
DVHDYSRFQPIVDELRKKIFDATTNNARLVLQIDNARLAADDFRVKYESELSIRQGVEADITGLRKVIDDTNLNRMNLESEIEALKEELIFLKK
NHDNEVMELRNQISQSGV-QVDVDA-
PKGQDLSQIMEEIRAKYEKMALKNQEELKAWHESQITEVQVQVTQNTEALQGARSEVNELRRQIQTLEIELESQKNLKGSLEGTLRDTEMRYNM
EIENLNTIILQLEAELTQLRGNIQHQTQEYEALLNIKMKLEAEIATYRRLLDGG
>Dr-krt-NP_956862.1
NEKGQMQNLNDRLAAYLDTVRRLEQENGKLEQQIREALEKGGP-
ETRDYSKYNAILDDLRRKVFDATVDNARLVLQIDNARLAVDDFRVKFENEMAIRQSVEGDIAGLKKVIDETNIGRLNVEGEIESLKEELLFLKK
NHENEVDELRSQISQSGV-QVDVDA-
PKGQDMSQVMEDMRANYEKQALKNAEELKMWHETQIADVQVQVAQNTEALQGAQMECNDLRRQIQTLEIELASQQNLKASLEDTLRNTEIRSNG
EMEKLNNIIIQLEAELAQLRANITEQGQEYEALLNMKMKLEAEINTYKKLLDGE
>Dr-krt-NP_001017588.1
SEKQTMQNLNDRLASYLERVRSLEQENKKLELQIKEFYDSKSPMQSKDMSAYFKTISELRAQIHGRFLENAELHLKLDNIRLAAEDFHIKYESE
LNMRTIVEADSARLRGVLSEIKLSIGDLQSQFTLLKEEQVYLKKNHEEDLHLLREQH-SGSV-NVEMDC-
ADQSHLDEELREMRAQYEKLIEKNRREAERWFHSKAEVLQTQVDTSSTEIKTSQTQLTDLRRTFQSLEIELQGVLTMKQNLENTLADVGIRYTT
QLSQLQLRIDHLQEELQKLNTNIRQQASEYQILLDIKMRLEMEIAEYRRLLEGE
>Cc-krt-XP_041029365.1
NEKTTMQNLNERLSSYLDRVHSLETSNSKMELEIRQLLEKATP-
```

AVRDWSVYWGTIKDIRDQINDLILDNSRLMLQIDNSKLAAEDFKTKFEAELGIRMAVEGDINGLRTVLDDMTLEKSQLEMQIEGLKEELIYIRK
NHEEELRGLRGQI-SGNV-TVDVTA-
EQSPDLSKMLEEMRQLYDDITKKNKSEAEEWYRQQCATVQQEFVVNTEALQAEKSQVVQLRHTLQGLDMELQTQLSMNASLNATLKDTEVRYSE
EYNRIQLTINKLEAELVEMRLKIDQHVKDYADLLDIKSRLEMEISTYRRLLEGG
>Cc-krt-XP_041029409.1
NEKTTMQNLNERLSSYLDRVHSLETSNSKMELEIRQLLEKATP-
AVRDWSVYWGTIKDIRDQINDLILDNSSLMLQVDNSKLAAEDFKTKFEAELGIRMAVEGDINGLRTVLDDMTLEKSQLEMQIEGLREELIYIGK
NHEEELRGLRGQI-SGNV-TVDVTA-
EQSPDLSKMLEEMRQLYDDIAKKNKSEAEEWYRQQCATVQQEFVVNTEALQAEKSQVVQLRHTLQGLDMELQTQLSMNASLNATLKDTEVRYSE
EYNRIQLTINKLEAELVEMRLKIDQHIKDYADLLDIKSRLEMEISTYRRLLEGG
>Dr-krt-XP_005155788.1
DKKATMQNLNTRLASYLEKVRSLEKANTELEQKISDWYDSHDD-
VTFDHTNFQDTIQDLRNEFNTRSQDNAKLILEVDNAKLAADDFKRKYENELAMRREIEADTGNLRKILDEFSLSRSDLELQIEALKEEFIVLKK
NHKENI-TLTIET-GGQV-NVSVDA-
APSMDLNQAIDEIRQHYETVTQKNREELESWYESKMAPMQQEVSNHNEELQDSRTELKDLTSTLQRLQIELQTHQSMKSNLDGQLEDTEARYGN
QLAGLQTTVSNLEDQLSQFHANIANNKEEYETLLDVKTRLEREIAEYRRLLDGE
>Pa-KRT12L1-XP_043910921.1
NKKETMQNLNKRLASYLEKVRYLEEENIRLEEQICEWYEKRRP-
VNQDWSEYEKMIANIQTEINKATVAKTEVVLQLDNAKLAADDFKVKYNNEVLLKQSTETDVNSLRKLLSDLDLSKHDLEKAVDNVNDEINYLKK
NHKEEMTALRAQT-GPGV-NVEVDS-
SPGMELVKILADVRAQYENMAEKNRRDAEEWYNNKCDVLNQQLTTSTETLETYKSETVNLRRTIQTLEIELQSTQSIKISLDETLEETQNRYGL
QLQQLQQAVNTLEAELGQLRMETERQSIEYAILLDTKTRLEMEINTYRQLMEEK
>Pa-KRT-T1-6-XP_043910928.1
NEKITMQNLNDRLAKYLGKVQCLEEANNELELKIKEWHSQVIP-
AARDYGHLEKEIEDLQNKVNNCRVDTATILLHIDNAKLAADDFRNKYENEQSLRMGVEADINGLKRVLDELGLAKADADMQIESLRDEIDYLKK
NHEEDMKSVSSGI-AGQV-NVELDA-
APGTNLLDEMDACRRDQEAMIDQMRREAERWFNEKAKEVTVKSGEAQESLVSHTSEISDLKRSIQSLEIELQTVLAKKASLNSTLSGIESQYGV
RIQDIQMKINILEGQLADIRAQMEFQNEEYQMLLNVKQQLEGEIKTYRALLEGE
>Pa-KRT-T1-3-XP_043910932.1
NEKITMQNLNDRLGAYLEKVHCLEDENHKLELKIKEWYSQQIG-
PGRDYSPLEKEILELQNKIIALNVDNSSWILKIDNAKLAADDFRTKYENELALRKGVEADILGLQRVLDELIFNRSDLEMQIEGRMEELDYMKK
SHDEDMRTASSEI-TGQV-NVELDA-
APSTNLVEEMNSCRKQYEAMAEQIRQEAEKSFNERVNELKKSVDSDTEVLSTHKNEISNLSRHLQSLEIELQSQLGKKATLERALADTEQQYGM
RIQEIQFTIHALEEQLANIRIQMESQSQDYQLLLNIKQRLEQEIETYRILLEGE
>Pa-KRT-T1-4-XP_043910931.1
NEKITMQNLNDRLATYLQNVRSLDDANRDLELKIRDWHSQQIG-
PGRDYSPFEKEIIDLQNKVISAKMENAHYILEIDNAKLAADDFRMKYENEFTMRNGVEADILGLKKVLDEMTLNKADLEMQIESIREELEYIKK
SHEEDMKSASSGI-SGQV-NVELDA-
PPQINLQDEMDACRQQYNAMMEQIRRDAEKWYNEKANELNKSVSSDTAAMSSFKSEISDLNRILQGLQIELQSQMGRKATLERALADTESQYGL
RIQDIQLKINCLEDQLSGLRLQIEQQNQDYQMLLNIKSRLEQEIDTYRRLLEGE
>Pa-KRT-T1-5-XP_043910930.1
NEKITMQGLNERLANYLDKVRSLEEENRNFEVKIKEWCSQGIG-
AGRDYSPLENEIQELQSKLINATMDNSNLILQIDNAKLAADDFRLKFENELSMRQGVEADIAGLRRVLDELTLSKSDYEMQIESIREELAYLNK
CHDEDMKAASSGI-AGQV-NVELDA-
APGPNLMDEMDACRKEYEAMVDKIRRDAEKWYNEKAKDITQSVASDTEAISSFKSEISDLRRTIQSLEIELQTQIGRKGSLESNLAETEGRYGM
MIQEIQMKISALEDQLANLRSQMEYQSQEYQMLLGVKQQLEQEIETYRILLEGE
>Pa-KRT-T1-2-XP_043910934.1
NEKHTMQGLNDRLAIYLKSVRSLEAENNDLELKIREWYSQQIG-
HGRDYSPLENEILELQNKVVAAKQDNASYIIQIDNAKLAADDFRMKYESEKVLRTGVEADLLGLRRVLDEMTLSRSDLEMEIEGLKEELEFMKK
SHEEDMRTASSGI-SGQV-NVELDA-
APDTNLMDEMDACRKEYEGMVDQIRRDAEKWYNEKAKEVNQAVTSDKEALSSNKSEISDLRRTVQSLEIELQTLLTMKASLERNLAETENQYGV
RLQEIQLKIHALEEQLSNLRTQTENQNQDYQMLLDIKVRLEQEIETYRILLDGE
>human_KRT20
-EKMAMQNLNDRLASYLEKVRTLEQSNSKLEVQIKQWYETNAP-
AGRDYSAYYRQIEELRSQIKDAQLQNARCVLQIDNAKLAAEDFRLKYETERGIRLTVEADLQGLNKVFDDLTLHKTDLEIQIEELNKDLALLKK
EHQEEVDGLHKHL-GNTV-NVEVDA-APGLNLGVIMNEMRQKYEVMAQKNLQEAKE-
FERQTAVLQQQVTVNTEELKGTEVQLTELRRTSQSLEIELQSHLSMKESLEHTLEETKARYSSQLANLQSLLSSLEAQLMQIRSNMERQNNEYH
ILLDIKTRLEQEIATYRRLLEGE
>human_KRT9
-EKSTMQELNSRLASYLDKVQALEEANNDLENKIQDWYDKKGP-
IQKNYSPYYNTIDDLKDQIVDLTVGNNKTLLDIDNTRMTLDDFRIKFEMEQNLRQGVDADINGLRQVLDNLTMEKSDLEMQYETLQEELMALKK
NHKEEMSQLTGQN-SGDV-NVEINV-APGKDLTKTLNDMRQEYEQLIAKNRKDIEN-
YETQITQIEHEVSSSGQEVQSSAKEVTQLRHGVQELEIELQSQLSKKAALEKSLEDTKNRYCGQLQMIQEQISNLEAQITDVRQEIECQNQEYS
LLLSIKMRLEKEIETYHNLLEGG
>Cc-krt-XP_041029614.1

NEKQTMQNLNDRLALYLEKVRSLEKSNNQLERQIREFYEKRTP-
GTKDLGSYWKTISELRAQINDASLANARVLLQIDNAKLAADDFKTKYEAEYSIRCGVEMDIAGLRKVLDELTFTRSDLESQIEQLKEETIYTKK
NHEEELKSLHNQI-RGTV-TVDVDS-
TPGTDLTKALAEIREKYELISAKNQKEAESWYKEQSANLQQTVVTNTEMIQTEKSQLTEQRRKLQALEIELQTMLSVKMSLEDSLQEIEDRYAV
ELNRLQGIMADREAEFMHIQTEMERKTQEYAQLLDVKSRLEMEIATYRHLLEGE
>Pa-KRT23L1-XP_043910879.1
SGKEAMQNLNDRLANYLEKVRSLEERNNELEIKIREWYDKQGS-
KPKDFSHFLKIIADLQNQIHDGNMENARILLKIDNAKLAADDFKQKWEAEQLMRLNVEGDINGLRKILDEMMLARTDLEMQIDGQKEELAYLNK
SHDEEMKALRSQL-GGQV-SVEVDA-
APTEDLTKKMELIRQQYEQLAEKNRKDAEDWFLKASEDLNRNVASSTEAIQTSRTEINELRRTIQGLEIELQSQLSMKDALEGQLADTELRYSS
ALMNLQNVINQKEAELSNIRADIERQANEYKILLDVKNRLENEIKTYRILLEGD
>Pa-KRT23L2-XP_043910874.1
SGKEAMQNLNDRLADYLVKVRGLEDKNKELEIKIREWHEKQGA-
KTKDFTHYFKTIADLQNQVNAGNMENAKILLKIDNAKLAADDFRQKWEAEQALRLSVEGDIHGLRKILDEMTLGRTDLEMQIEGHKEEKAYLIK
SHDEDMKALRSQL-GGQV-NVEVDA-
PPTEDLTKKLELIRQQYEQLAEKNRKETEDWFTKKSEELNKNMVSSTEALQTSKTEINELRRTIQGLEIELQSQQSMKGALEGQLAETEHRYSS
TLMNLQNIINQKEAELSNIRADIERQSSEYKILLDVKTRLEKEIATYRILLEGD
>human_KRT37
-EKETMKFLNDRLANYLEKVRQLEQENAELETTLLERSKCHES-
VCPDYQSYFRTIEELQQKILCSKAENARLIVQIDNAKLAADDFRIKLESERSLHQLVEADKCGTQKLLDDATLAKADLEAQQESLKEEQLSLKS
NHEQEVKILRSQL-GEKF-RIELDI-EPTIDLNRVLGEMRAQYEAMVETNHQDVEQ-
FQAQSEGISLQAMSCSEELQCCQSEILELRCTVNALEVERQAQHTLKDCLQNSLCEAEDRYGTELAQMQSLISNLEEQLSEIRADLERQNQEYQ
VLLDVKARLENEIATYRNLLESE
>human_KRT38
-EKETMQFLNDRLANYLEKVRQLEQENAELEATLLERSKCHES-
VCPDYQSYFHTIEELQQKILCSKAENARLIVQIDNAKLAADDFRIKLESERSLRQLVEADKCGTQKLLDDATLAKADLEAQQESLKEEQLSLKS
NHEQEVKILRSQL-GEKL-RIELDI-EPTIDLNRVLGEMRAQYEAMLETNRQDVEQ-
FQAQSEGISLQDMSCSEELQCCQSEILELRCTVNALEVERQAQHTLKDCLQNSLCEAEDRFGTELAQMQSLISNVEEQLSEIRADLERQNQEYQ
VLLDVKTRLENEIATYRNLLESE
>human_KRT39
-EKETMQILNERLANYLQKVRMLERENAELESKIQEESNKELP-
LCPDYLSYYTTIEELQQKILCTKAENSRLVSQIDNTKLTADDLRAKYEAEVSLRQLVESDANGLKQILNVLTLGKADLEAQVQSLKEELLCLKN
NHKEEINSLQCQL-GERL-DIEVTA-APSADLNQVLQEMRCQYEPIMETNRKDVEQ-
FNTQIEELNQQVVTSSQQQQCCQKEIIELRRSVNTLEVELQAQHRMRDSQECILTETEARYTALLTQIQSLIDNLEAQLAEIRCALERQNQEYE
ILLDVKSRLECEITTYRSLLESS
>human_KRT40
-EKETMQFLNDRLASYLEKVRSLEETNAELESRIQEQCEQDIP-
VCPDYQRYFNTIEDLQQKILCTKAENSRLAVQLDNCKLATDDFKSKYESELSLRQLLEADISSLHGILEELTLCKSDLEAHVESLKEDLLCLKK
NHEEEVNLLREQL-GDRL-SVELDT-APTLDLNRVLDEMRCQCETVLANNRREAEE-
LAVQTEELNQQQLSSAEQLQGCQMEILELKRTASALEIELQAQQSLTESLECTVAETEAQYSSQLAQIQCLIDNLENQLAEIRCDLERQNQEYQ
VLLDVKARLEGEINTYWGLLDSE
>human_KRT32
-EKETMQFLNDRLASYLTRVRQLEQENAELESRIQEASHSQVL-
MTPDYQSHFRTIEELQQKILCTKAENARMVVNIDNAKLAADDFRAKYEAELAMRQLVEADINGLRRILDDLTLCKADLEAQVESLKEELMCLKK
NHEEEVGSLRCQL-GDRL-NIEVDA-APPVDLTRVLEEMRCQYEAMVEANRRDVEE-
FNMQMEELNQQVATSSEQLQNYQSDIIDLRRTVNTLEIELQAQHSLRDSLENTLTESEARYSSQLAQMQCMITNVEAQLAEIRADLERQNQEYQ
VLLDVRARLEGEINTYRSLLENE
>human_KRT34
-EKETMQFLNDRLASYLEKVRQLERDNAELEKLIQERSQQQEP-
LCPSYQSYFKTIEELQQKILCAKAENARLVVNIDNAKLASDDFRSKYQTEQSLRLLVESDINSIRRILDELTLCKSDLESQVESLREELICLKK
NHEEEVNTLRSQL-GDRL-NVEVDT-APTVDLNQVLNETRSQYEALVEINRREVEQ-
FATQTEELNKQVVSSSEQLQSCQAEIIELRRTVNALEIELQAQHNLRDSLENTLTESEAHYSSQLSQVQSLITNVESQLAEIRCDLERQNQEYQ
VLLDVRARLECEINTYRSLLESE
>human_KRT33b
-EKETMQFLNDRLASYLEKVRQLERDNAELENLIRERSQQQEP-
LCPSYQSYFKTIEELQQKILCSKSENARLVVQIDNAKLAADDFRTKYQTEQSLRQLVESDINSLRRILDELTLCRSDLEAQMESLKEELLSLKQ
NHEQEVNTLRCQL-GDRL-NVEVDA-APAVDLNQVLNETRNQYEALVETNRREVEQ-
FATQTEELNKQVVSSSEQLQSYQAEIIELRRTVNALEIELQAQHNLRYSLENTLTESEARYSSQLSQVQSLITNVESQLAEIRSDLERQNQEYQ
VLLDVRARLECEINTYRSLLESE
>human_KRT31
-EKETMQFLNDRLASYLEKVRQLERDNAELENLIRERSQQQEP-
LCPSYQSYFKTIEELQQKILCTKSENARLVVQIDNAKLAADDFRTKYQTELSLRQLVESDINGLRRILDELTLCKSDLEAQVESLKEELLCLKS
NHEQEVNTLRCQL-GDRL-NVEVDA-APTVDLNRVLNETRSQYEALVETNRREVEQ-
FTTQTEELNKQVVSSSEQLQSYQAEIIELRRTVNALEIELQAQHNLRDSLENTLTESEARYSSQLSQVQSLITNVESQLAEIRSDLERQNQEYQ
VLLDVRARLECEINTYRSLLESE

>human_KRT33a
-EKETMQFLNDRLASYLEKVRQLERDNAELENLIRERSQQQEP-
VCASYQSYFKTIEELQQKILCSKSENARLVVQIDNAKLASDDFRTKYETELSLRQLVESDINGLRRILDELTLCRSDLEAQVESLKEELLCLKQ
NHEQEVNTLRCQL-GDRL-NVEVDA-APTVDLNQVLNETRSQYEALVETNRREVEQ-
FATQTEELNKQVVSSSEQLQSYQAEIIELRRTVNALEIELQAQHNLRDSLENTLTESEARYSSQLSQVQRLITNVESQLAEIRSDLERQNQEYQ
VLLDVRARLECEINTYRSLLESE
>human_KRT35
-EKETMQSLNDRLAGYLEKVRQLEQENASLESRIREWCEQQVP-
MCPDYQSYFRTIEELQKKTLCSKAENARLVVEIDNAKLAADDFRTKYETEVSLRQLVESDINGLRRILDDLTLCKSDLEAQVESLKEELLCLKK
NHEEEVNSLRCQL-GDRL-NVEVDA-APPVDLNRVLEEMRCQYETLVENNRRDAED-
LDTQSEELNQQVVSSSEQLQSCQAEIIELRRTVNALEIELQAQHSMRDALESTLAETEARYSSQLAQMQCMITNVEAQLAEIRADLERQNQEYQ
VLLDVRARLECEINTYRGLLESE
>human_KRT36
-EKETMQFLNDRLANYLEKVRQLERENAELESRIQEWYEFQIP-
ICPDYQSYFKTIEDFQQKILLTKSENARLVLQIDNAKLAADDFRTKYETELSLRQLVEADINGLRRILDELTLCKADLEAQVESLKEELMCLKK
NHEEEVSVLRCQL-GDRL-NVEVDA-APPVDLNKILEDMRCQYEALVENNRRDVEA-
FNTQTEELNQQVVSSSEQLQCCQTEIIELRRTVNALEIELQAQHSMRNSLESTLAETEARYSSQLAQMQCLISNVEAQLSEIRCDLERQNQEYQ
VLLDVKARLEGEIATYRHLLEGE
>Pa-KRT-T1-13-XP_043910893.1
NEKVTMQNLNDRLASYMDKVRNLENANFELEKNIKDRYLKQSQ-
TGRDYSQYEKTIDDLRDKIQNANMEIASIMLGFDNAKLAAEDFKSKFEVEQTMRNNVEGDINGMRKLLDELTLCRADLELKIESLKEELAYMTK
SHEEESDALRNQI-TGEI-TVDLEV-
APNLDITKSLEEMRAQYDVLEKKNKADAEAWFKDRAEALNKEVSTDTKLLQTQTSEIADLRRTLQGLEIEKQSQLAMKNSLENTLAETEGRYGA
MLQNIQSSIHDIEDNLAHLRAEIERHSREYQMLFDIKTRLEHEIAVYHKLLEGD
>Pa-KRT-T1-12-XP_043910895.1
NEKATMQNLNDRLASYLDKVRSLENANLELEVKIKEHYLKQSQ-
TGHDYSHYEKTIDDLKDKVVNATVENASIVLGIDNAKLAADDFRQKFENEQAMRLSVEADINGMRKILDDLTLARAELELNIEGVREELAYLKK
NHAEEIDSVRSQM-GGQV-NVEIDA-
APTVDLTKTLEEMRAQYTAVVEKNKAEAEAWFKDKAADLSKEVSTGTELLQTHKTEITELRRTLQGLEIELQSQLAMKFSLENTLSETEGRYVA
MLQNIQSAIHDIEENLSQLRAEIERQSLDYQMLLDVKTRLEKEITQYRILLEGE
>Pa-KRT-T1-10-XP_043910901.1
NEKATMQNLNDRLASYLDKVRDLGKENSELEQKIKEHYLKQTQ-
TGRDYSEFEKIIEELRDKLINVQVDNATILLGIDNAKLAADDFRQKYETEQAMRFGVEADINGMRKILDDLTLAKAELESNIEAFRDDLAYINK
NHREEMDSMRTQI-GGQV-NVEVDA-
APGVDLTKVMEEMRAEYTAMVEKNKAEAEAWFKDKAADLNKEVSTGTELLQTHKTEITDLRRTLQGLEIELQSQLARKNTLQHSLDETEGRYGS
QLQDMQFSIHDIEENIANLRAQIEHQSREYQLLLDVKTRLENEINMYRKLLEGE
>Pa-KRT-T1-8-XP_043910911.1
NEKITMQNLNDRLANYLGNVHMLERKNAELEKNIKEWYLKQSQ-
TTRDYSEYEKIIDDLRNKILNKNVDNAEAVLHIDNAKLAADDLRLKYENELNLRQNVEADINGLHRVMDELSLCRADLESQIEALMEDLNFLKK
NHVEEMAALQAQI-GGDV-NVEIDA-
APGIDLTKAMDEMRCQYETIVEKNQREAEDWYKKKASELSKEVSSSTELLQTHKTEMTNLKHTLQGLEIELQTLLATKCSLENILADTEARYGA
MLQDIQASVNNVEVNLANIRSEIECQSRDYQLLLDTKTRLEREISEYRQLLEGE
>Pa-KRT-T1-9-XP_043910907.1
NEKATMQNLNDRLASYLTKVHDLEKENSALELKIKDWYLKQTH-
TGRDYSEYEKTIDDLRDKIQNITVGNANILLSIDNAKLAADDFRLKYENELNMRQCVEADINGMRRLLDDLTLSRADLESQIECMKEDLAYTKK
NHADEISSLHSHV-TGNI-NVEVDA-
APGTDMMKALAEIRHDYETIIEKNNADAEAWFNKQASELSKEMTVSTEALQTHKTEITDLRRTLQGLEIELQAQLAKKGSLDNTLRDTESRYGT
MLQDIQISINNLEASLANIRSEIECQSREYQLLLDVKTRLEKEINDYRQLLEGE
>Pa-KRT12L2-XP_043910914.1
NGKETMQNLNERLAAYLEKVRSLESKNKQLERQIREWYEKKPP-
VRLDVSGYEKTIATLRSQINAGTLANSRIILQIDNAKLAADDFRIKYESELAIRKSVEADIISLRRVLDELNLSRSGLEIQIDTLKEELAYHKK
NHEEEMESLKGKL-GGQV-NVEVDT-
SPSTDLHKIMEGIRKQYEEIADQNRKDAEAWYKDKFEALNQEVSINTTALNTSKNEVTELKRTIQTLEIELQSLISMKGALEGTLQETQSRYGT
QLQGLQAKINTLEGELSQLKMEMERQGNEHKILLDIKTRLEKEIETYRRLLEGE
>Pa-KRT-T1-7-XP_043910913.1
SEKQTMQNLNDRLANYLKKVHSLEVENGELEKKIREWHMSKSP-
THHDYSSFNETIADLQKKIQDATVENAEILVNLDNAKLATDDFRTKYENELTFRQGVDADIAGMRRMLDDLTLCRSDLEMQIEGLKDELDNLNK
THEEDMKSMSTQL-SGQV-SVEVDA-
VPGVDLTRSLEEMRSQYDGMVEKNRREAKEWFDNKSIELNKEVTTSTETLQIQKTEITDLRRTLQGLEIELQSQLSMRNGLQSNLAETESRYGT
QLQQIQAIIHETEAQLTNLRAQLEGQATEYKLLLDIKAHLEQEINTYRHLLEGE
>Pa-KRT70-XP_043910867.1
NEKETMKNLNDRLAAYLEKVRILEAGNHQLEKKIREYYEKKSP-
AEKDMSGYFATINELRDKISAASIDNARLVLQIDNAKLAADDFRIKYENELALRQGVEMDINGLKKVLDELTLARTDLELQIESLKEELIFLKK
THEEEMNSLHGQV-TGAV-NVEVDS-
APPVDLSKIIAEIREKYETMIEKNRQEMEKWHKEQFDALNKEVATSTEAIQSSKTEMTELRRTIQGLEIELQSLLSMKQALEGTLADTEGRYAN

QLRQYQDMISRLELELQDVRNDIERQGQEYTILLNVKTRLEQEIAEYRRLLEGE
>human_KRT26
-EKVTMQNLNDRLASYLDHVHALEEANADLEQKIKGWYEKCEP-
HDHDYSRYFSVIEDLKRQIISATICNASIVLQNDNARLTADDFRLKYENELALHHSVEADTSGLRRVLDELTLCTTDLEIQCETLSEELTYLKK
SHEEEMEVLQYTA-GGNV-NVEMNA-TPGVDLTVLLNNMRAEYEDLAEQNRKDAEA-
FNERSATLQQQISDHEGAATAARNELTELKRNLQTLEIELQSLMAVKHSYECSLAETEGNYCNQLQQIQDQIGVMEEQLQQIRTETEGQKLEYE
QLLDVKIFLEKEIDIYCNLLDGE
>human_KRT28
-EKVTMQNLNDRLASYLDNVRALEEANAELERKIKGWYEKYGP-
LDHDYSRYHLTIEDLKNKIISSTTTNANVILQIDNARLAADDFRLKYENELTLHQNVEADINGLRRVLDELTLCRTDQELQYESLSEEMTYLKK
NHEEEMKALQCAA-GGNV-NVEMNA-APGVDLAVLLNNMRAEYEALAEQNRKDAEA-
FNEKSASLQQQISHDSGAATFARSQLTEMRRTLQTLEIQLQSLMATKHSLECSLTETESNYCTQLAQIQAQIGALEEQLHQVRTETEGQKLEYE
HLLDVKVHLEKEIETYCRLIDGD
>human_KRT25
-EKVTMQNLNDRLASYLDSVHALEEANADLEQKIKGWYEKFGP-
LDHDYSRYFPIIDDLKNQIIASTTSNANAVLQIDNARLTADDFRLKYENELALHQSVEADVNGLRRVLDEITLCRTDLEIQYETLSEEMTYLKK
NHKEEMQVLQCAA-GGNV-NVEMNA-APGVDLTVLLNNMRAEYEALAEQNRRDAEA-
FNEKSASLQQQISEDVGATTSARNELTEMKRTLQTLEIELQSLLATKHSLECSLTETESNYCAQLAQIQAQIGALEEQLHQVRTETEGQKLEYE
QLLDIKLHLEKEIETYCLLIGGD
>human_KRT27
-EKVTMQNLNDRLASYLENVRALEEANADLEQKIKGWYEKFGP-
LDHDYSRYFPIIDELKNQIISATTSNAHVVLQNDNARLTADDFRLKFENELALHQSVEADINGLRRVLDELTLCRTDLEIQLETLSEELAYLKK
NHEEEMKALQCAA-GGNV-NVEMNA-APGVDLTVLLNNMRAEYEALAEQNRRDAEA-
FNEKSASLQQQISDDAGATTSARNELIEMKRTLQTLEIELQSLLATKHSLECSLTETESNYCAQLAQIQAQIGALEEQLHQVRTETEGQKLEYE
QLLDIKVHLEKEIETYCLLIDGE
>human_KRT24
-EKQTMQNLNDRLANYLDKVRALEEANTDLENKIKEWYDKYGP-
SGRDYSKYYSIIEDLRNQIIAATVENAGIILHIDNARLAADDFRLKYENELCLRQSVEADINGLRKVLDDLTMTRSDLEMQIESFTEELAYLRK
NHEEEMKNMQGSS-GGEV-TVEMNA-APGTDLTKLLNDMRAQYEELAEQNRREAEE-
FNKQSASLQAQISTDAGAATSAKNEITELKRTLQALEIELQSQLAMKSSLEGTLADTEAGYVAQLSEIQTQISALEEEICQIWGETKCQNAEYK
QLLDIKTRLEVEIETYRRLLDGE
>human_KRT12
-EKETMQNLNDRLASYLDKVRALEEANTELENKIREWYETRGT-
SQSDYSKYYPLIEDLRNKIISASIGNAQLLLQIDNARLAAEDFRMKYENELALRQGVEADINGLRRVLDELTLTRTDLEMQIESLNEELAYMKK
NHEDELQSFRVGG-PGEV-SVEMDA-APGVDLTRLLNDMRAQYETIAEQNRKDAEA-
FIEKSGELRKEISTNTEQLQSSKSEVTDLRRAFQNLEIELQSQLAMKKSLEDSLAEAEGDYCAQLSQVQQLISNLEAQLLQVRADAERQNVDHQ
RLLNVKARLELEIETYRRLLDGE
>human_KRT10
-EKVTMQNLNDRLASYLDKVRALEESNYELEGKIKEWYEKHGN-
EPRDYSKYYKTIDDLKNQILNLTTDNANILLQIDNARLAADDFRLKYENEVALRQSVEADINGLRRVLDELTLTKADLEMQIESLTEELAYLKK
NHEEEMKDLRNVS-TGDV-NVEMNA-APGVDLTQLLNNMRSQYEQLAEQNRKDAEA-
FNEKSKELTTEIDNNIEQISSYKSEITELRRNVQALEIELQSQLALKQSLEASLAETEGRYCVQLSQIQAQISALEEQLQQIRAETECQNTEYQ
QLLDIKIRLENEIQTYRSLLEGE
>human_KRT19
-EKLTMQNLNDRLASYLDKVRALEAANGELEVKIRDWYQKQGP-
PSRDYSHYYTTIQDLRDKILGATIENSRIVLQIDNARLAADDFRTKFETEQALRMSVEADINGLRRVLDELTLARTDLEMQIEGLKEELAYLKK
NHEEEISTLRGQV-GGQV-SVEVDS-APGTDLAKILSDMRSQYEVMAEQNRKDAEA-
FTSRTEELNREVAGHTEQLQMSRSEVTDLRRTLQGLEIELQSQLSMKAALEDTLAETEARFGAQLAHIQALISGIEAQLGDVRADSERQNQEYQ
RLMDIKSRLEQEIATYRSLLEGQ
>human_KRT16
-EKVTMQNLNDRLASYLDKVRALEEANADLEVKIRDWYQRQRP-
EIKDYSPYFKTIEDLRNKIIAATIENAQPILQIDNARLAADDFRTKYEHELALRQTVEADVNGLRRVLDELTLARTDLEMQIEGLKEELAYLRK
NHEEEMLALRGQT-GGDV-NVEMDA-APGVDLSRILNEMRDQYEQMAEKNRRDAET-
FLSKTEELNKEVASNSELVQSSRSEVTELRRVLQGLEIELQSQLSMKASLENSLEETKGRYCMQLSQIQGLIGSVEEQLAQLRCEMEQQSQEYQ
ILLDVKTRLEQEIATYRRLLEGE
>human_KRT14
-EKVTMQNLNDRLASYLDKVRALEEANADLEVKIRDWYQRQRP-
EIKDYSPYFKTIEDLRNKILTATVDNANVLLQIDNARLAADDFRTKYETELNLRMSVEADINGLRRVLDELTLARADLEMQIESLKEELAYLKK
NHEEEMNALRGQV-GGDV-NVEMDA-APGVDLSRILNEMRDQYEKMAEKNRKDAEEFF-
TKTEELNREVATNSELVQSGKSEISELRRTMQNLEIELQSQLSMKASLENSLEETKGRYCMQLAQIQEMIGSVEEQLAQLRCEMEQQNQEYKIL
LDVKTRLEQEIATYRRLLEGE
>human_KRT17
-EKATMQNLNDRLASYLDKVRALEEANTELEVKIRDWYQRQAP-
PARDYSQYYRTIEELQNKILTATVDNANILLQIDNARLAADDFRTKFETEQALRLSVEADINGLRRVLDELTLARADLEMQIENLKEELAYLKK
NHEEEMNALRGQV-GGEI-NVEMDA-APGVDLSRILNEMRDQYEKMAEKNRKDAEDFF-

```
SKTEELNREVATNSELVQSGKSEISELRRTMQALEIELQSQLSMKASLEGNLAETENRYCVQLSQIQGLIGSVEEQLAQLRCEMEQQNQEYKIL
LDVKTRLEQEIATYRRLLEGE
>human_KRT13
-EKITMQNLNDRLASYLEKVRALEEANADLEVKIRDWHLKQSP-
PERDYSPYYKTIEELRDKILTATIENNRVILEIDNARLAADDFRLKYENELALRQSVEADINGLRRVLDELTLSKTDLEMQIESLNEELAYMKK
NHEEEMKEFSNQV-VGQV-NVEMDA-TPGIDLTRVLAEMREQYEAMAERNRRDAEE-
FHAKSAELNKEVSTNTAMIQTSKTEITELRRTLQGLEIELQSQLSMKAGLENTVAETECRYALQLQQIQGLISSIEAQLSELRSEMECQNQEYK
MLLDIKTRLEQEIATYRSLLEGQ
>human_KRT15
-EKITMQNLNDRLASYLDKVRALEEANADLEVKIHDWYQKQTP-
PECDYSQYFKTIEELRDKIMATTIDNSRVILEIDNARLAADDFRLKYENELALRQGVEADINGLRRVLDELTLARTDLEMQIEGLNEELAYLKK
NHEEEMKEFSSQL-AGQV-NVEMDA-APGVDLTRVLAEMREQYEAMAEKNRRDVEAFF-
SKTEELNKEVASNTEMIQTSKTEITDLRRTMQELEIELQSQLSMKAGLENSLAETECRYATQLQQIQGLIGGLEAQLSELRCEMEAQNQEYKML
LDIKTRLEQEIATYRSLLEGQ
>Dr-krt-NP_001035449.1
NEKATMQNLNDRLASYLDKVRSLEAANATLERQIREYYEKKGPVAQRDYSQYWNTIKDLKDKIKNATTHNANILLQIDNSKLAADDFRLKFEHE
VVMRQCVESDIANLRRLLDQTNLAKNDLQMQIKGLEEELVFLKKNHQEEMAALRCQL-TGTV-NVEVDA-
APQQDLNKVLEEIRTHYENIIQKHRREQEAWFKDKTAPLNKEVAHSTETIQTSRSQITELRNTLQSLEIELQSQLSMKAALEHSLAETEARYSA
MLAGFQNQINNLEAELAQLRASIEQQGRDYALLLDIKTRLEQEIATYRNLLENQ
>Dr-krt94-NP_001070922.1
NEKATMQNLNDRLASYLDKVRSLEAANATLERQIREYYEKKGPVAERDYSHFWNTIKDLKDKIKNATIHNANILLQIDNSKLAADDFRIKFEHE
LVMRQSVEADIANLRRLLDQTTLTKADLEMQIEGMQDELAFMKKNHQEDLAALRSQL-TGTV-NVEVDA-
APQQDLSKVLEEIRAHYENIIQKHRREQEDWFKDKSAGLSKEVAISTETIQTTKTQITDLRRTLQGLEIELQSQLSMKAALENSLGDTEARYSA
MLAGYQNQINMLEAELAQLRASIEQQSRDYILLLDIKTRLEQEIATYRSLLENQ
>Dr-krt-XP_683942.5
NEKATMQNLNDRLASYLDKVHFLEAANATLEKQICDYFEKKDP-
CQKDYSCYLKTIDCLQKKIKDATITNGKILLRIDNSKLAADDFRIKYENELSIHQCVKADIDNLRCILDKTCLAKADLELQICTLEEELVYLRK
THQEDVAALMCQLTNSKV-CVEVDA-
APQQDLKKVLDDIRCYYDTIIDKHCTEQECWFKEKMAGLCKDTAINTECLETSMSRISDLRRTLQSLEIELQSQISLKGALECSLLETEARYST
MLAGYQKHINAYEAELCQVRAGIEQQGRDYDALLDIKSRLEQEIATYRCLLENQ
>Dr-krt-NP_001002392.1
NEKTTMQNLNDRLASYIEKVRCLEAANATLEQQIRDYYEKKGP-
CQRDYSCYWNTINCLKEQIAAANVNNANILLQIDNSKLAADDFRIKYEHEIAVRQCVESDIANLRRLLDQATLSKAELENQIETLIGDLECLKK
NHQEDVAALMCQLTNSKV-CVEVDA-
APQQDLNKILDDIRCHYENIIDKHRREQECWFKEKTAQLCKDVAGDTECLETSRSQISDLRRTLQCLEIELQSQLSMKCALEASLRETEARYST
MLAGYQKHINTYEAELSQVRAGIEQQGRDYDALLDIKSRLEQEIATYRCLLENQ
>Dr-krt-NP_001002383.1
NEKTTMQNLNDRLASYLEKVRSLEKANAELERQIREWYDKRTP-
VSRDYSHYYVTIEDLRKKISVASQDNARIILQIDNAKLAAEDFRVKYENELALRLSVEADIAGLRKVLDDLTMTRSDLEMQIEGLKEELVYLKK
NHAEELAALRAQMTSSSV-NVEVDA-
APQQDLARIMEEMRQQYEGITEKNKREMEAWYKGKFDELNKQVSTRQEDLSMSRNEINELRRTLQALEIELQSQLSLKSALEGTLGETESRYSI
QLNQLQAVINSLEQELTQMRMDIERQASEYKLLLDIKTRLEMEIAEYRRLLDGE
>Dr-krt-NP_001186881.1
FEKTTMQNLNDRLASYLEKVRSLEKANAQLERQIREWYDKRTP-
VSRDYSHYYVTIEDLRKKISLASQDNARIILQIDNANLSAEDFRVKYENELALRLSVEADIAGLRKVLDDLTMTRSDLEMQIEGLKEELVYLKK
NHAEELAALRAQMTSSSV-NVEVDA-
APQQDLARIMEEMRQQYEGITEKNKREMEAWYKGKFDELNKQVSTQQEDLSMSRNEINELGRTLQALEIELQSQLSLKSALEGTLGETESRYSI
QLNQLQAVINSLEQELTQMRMDIERQASEYKLLLDIKTRLEMEIAEYRRLLDGE
>Dr-krt-NP_001107814.1
NEKFTLQNLNDRLASYLDKVRSLEKANAELELKIREFLENKIS-
AARNYSAFETVVSDLQAKILAAIQLNNTVHLSIDNSSLAADDFRIKYENELSMRQSVEADIAGLKNVLSDLNMGQKDLNLQIEALTEELAYMKA
NHQEDLSTSRDQM-SGQV-NVEVDA-
APQEDLTKVLADIREHYETVVSKSQRELEGWFQQKSETLKQEVVASTETLQTSKTEVNSVKSSVQSLEIELQSLLAMKSSMEGTLNDTQNRYSM
QLSGYQAQVSGMEGQLVQLRADLERQGQEYQMLLDIKTRLEMEIAEYRRLLDAE
>Dr-krt-XP_001342789.4
NEKITMQSLNDRLATYLAKVRALEKANAELELKIRQFLDGKAS-
KTRDYSAYYVTIEDLQTKIMAAIHLKGGLHLSIDNTSLAMNDFKTKFEMEHVMRQSVEADIVGLKRVLDELNMSRKDITMQIEGLKEELVFLKK
NHEEDLLAARAQM-SGQV-HVEVDA-
APHQDLTKILAEIREHYEAVTAKNQRELEQWFKTKSESLKQEVVTSTTDLKTSRSEINAVKSRLQALEIELQSLLALKASLETTLSDTKNRYSM
KLSGYQHQVTVLEEQIVQLRADLERQRHDYQMLLDIKARLEMEIAEYRRLLDGE
>Dr-krt-NP_998688.2
NEKATMQNLNDRLATYLDKVRSLEKANADLELKIRQFLESKTSPAARDYSAYQATIKDLQDKIQDATRVNGGVYLAVDNAKLAADDFKTKYENE
LTMRQSVEADIAGLKRLLDELTLARSDLEMQIEGLKEELIFLKKNHEEELAAMRSQM-SGSV-NVEVDA-
APQVDLNHVMAEIREQYENVAAKNQRDLENWFQTKSETLTKEVAASTETIQVSKTELTELRRTLQGLEIELQSELSKKASLEGTLADTEARFSR
QLMNLQTQVTNLEEQIVQLRADMDRQGQEYKMLLDIKTRLEMEIAEYRRLLDGG
```

```
>Dr-krt-NP_001003445.1
NDKATMQNLNDRLASYLEKVRSLEKANADLELKIRQFLDSKTS-
SARDYSAYYATISDLQNKIQDATRINGGIYLSIDNAKLAADDFRVKYENELSMRQSVEADIAGLRKVLDELTMTRSDLEMQIEGLKEELIFLKK
NHEEELLAARGQM-SGQV-HVEVDA-
APQEDLTKVMADIREHYEQVAAKNQRDLEHWFQSKSESLNKEVAASTETLQSTKSEITELKRTLQGLEIELQSQLSMKASLEGTLADTQARYGN
MLNGYQMQVGNMEEQLMQLRADLERQGQEYQMLLDIKTRLEMEIAEYRRLLDGE
>Dr-krt-NP_001076574.1
NEKATMQNLNDRLASYLEKVRILEKENADLELKIRQFLDSKAT-
NARDYSAYYATISDLQAKILHATGVNAGIYLHIDNAKLAADDFKVKFDNEQSMRHAVEADISGLRKVLDELTMTRSDLEMQIEGLKEELVFLKK
NHEEELLAARTQM-GGQV-NVEVDA-
APQEDLTKIIADIREHYEAVSAKYQKDFEQWFQSKSESLNKEVAASTETLQSSRSEFTELKRTLQSLQIELQSQLSMKASLEGTLADTQARYAN
MLNGYQLQVGSLEEQLVHLRGDLERQRQEYQMLLDIKTRLEMEIAEYRRLLDGE
>Dr-krt-NP_001017824.1
NEKATMQNLNDRLASYLEKVRTLERANADLELKIRQFLDSKTA-
SARDYSAYYATISDLQVKIQHATGLNGGIYLSIDNAKLAADDFRVKYENELSMRQSVEADIAGLRRVLDELTMTRSDLELQIEGLREELIFLKK
NHEEELLAVRAQM-TGQV-NVEVDA-
APQEDLTKIMAEIREHYESVAAKNRKDLEAWFQAKTETLTKEVAVSTETLQISRSEITEVKRTLQSLEIELQSQLSMKASLEGTLADTQARYAA
MLSGYRFQVSSLEEQLVQLRADLERQSQEYKMLLDIKTRLEMEIAEYRRLLDGE
>Dr-krt-NP_001076351.1
NEKATMQNLNDRLASYLEKVRSLEKANADLELKIRQFLDSKAT-
NARDYSAYYATISDLQAKILHATGVNGGIYLSIDNAKLAADDFRVKYENELTMRQSVEADIVGLRKVLDELTMTRSDLELQIEGLKEELIFLKK
NHEEELLAARTQM-SGQV-NVEVDA-
APQEDLTKIMADIREHYEAVSAKNRKDLESWFQAKSESLNKEVAVSTETLQTSRSEITEVKRTLQSLEIELQSQLSMKASLEGTLADTQARYTN
MLNGYQFQVSSLEEQLIQLRADLERQGQEYQMLLDIKTRLEMEIAEYRRLLDGE
>Dr-krt-NP_571182.1
NEKATMQNLNDRLASYLEKVRSLEKANADLELKIRQFLDSKAT-
NARDYSAYYATISDLQAKILHATGVNGGIYLSIDNAKLAADDFRVKYENELSMRQSVEADIVGLRKVLDELTMTRSDLELQIEGLKEELIFLKK
NHEEELLAARTQM-SGQV-NVEVDA-
APQEDLTKILADIREHYEAVSAKNRKDLESWFQAKSESLNKEVAVSTETLQTSRSEITEVKRTLQSLEIELQSQLSMKASLEGTLADTQARYTN
MLNGYQFQVSSLEEQLIQLRADLERQGQEYQMLLDIKTRLEMEIAEYRRLLDGE
```

Figure S14. Multiple sequence alignment of type I keratins used to generate the phylogeny shown in Figure S13A. The protein sequences are provided in fasta_aln format. Species: lancelet (Bf, *Branchiostoma floridae*), sea lamprey (Pm, *Petromyzon marinus*), great white shark (Cc, *Carcharodon carcharias*), zebrafish (Dr, *Danio rerio*), lungfish (Pa, *Protopterus annectens*), human (*Homo sapiens*).

```
>Bf-krt-XP_035663591.1
ELQGLRVHNDKLASYIEKVRVLEDQNRDLELQYAVLSRA--
PMSASGGADQGQAQMDAYQEQLRKARDMLQQLQGRLEQEVAGYEVEVAELTAQIQAEMAALAAMEEELAVLRKSVAEANAEGKRLQGAMEMLSV
NLDF----------
QIQANVQEERVTLEVQQQAEAEFQVSVESMRKEFEVNAGARKAQLEEMYARRLAALQTQKQRLEAQISEAKREIKTVEREIQQALRELDKLRET
TARLQAELEATDIEGQRQFEMMEVTIAELEAVLVELRDRLQAQRDAYSELQRVKMKMDMEITCYKQLLEGE
>Bf-krt-XP_035663589.1
DKDELVGLNDRFASFIEKVRFLENQNRKLEMKLKMVQQK--G-----
SGPDLGAMWEAELRQIRQLIEVVNTERGSLEAERDGLSGEVKELKTRYEDEVGTREGLEEEIKKIRADFDEASLTRVDLEARLDSIKSEIEFLK
EVYAAEIEALNSQILDTMIELEPTAGPDVDLDSCLAEVKAQYEQLTRMSRAEAESWYATKFDDLQRSSGKNTNDLADARAELSKYQNQIARLQS
EIESMKNRNRQLEGQLKNVEESGATKLAEKQAEIEALEAELQRLRGEINKQMKEYAELQNVKMALDVEIAAYRKLLEGE
>Bf-krt-XP_035658655.1
DKDELAGLNDRFATFINKVRYLEEMNRKLTLQLEMVQKK--AG---
VGGPDIGKMWEAELNNIKRLTEVVINEKNVLQSESDGLQAEVTTMKKSYEDEHTTVETLQAEVTKLRPEMDGVSVERVDLEARLDTIKAEIDFL
KEVYAAELEALQGQVLDGTLELQTVASPDIDFDSIVEEVRVQYEALALQSKQEAQSWYETKFESLQQQSGSASGGLSAARAELSKYGSEMKKIQ
SQLDAAKSRNARLQEQLAAVEAKGVSQLEAKQLEITALETELQTLRGQIKAQMTEYNSLLSVKMALDIEISAYRKLLEGE
>Bf-krt-XP_035658656.1
DKDELAGLNDRFATFINKVRYLEEMNRKLTLQLEMVQKK--AG---
AGGPDINKMWEAELNNIRKLIEVVNNEKNALNSEKDGLQGEAATLKASYEEEKTRNEGLQDEITALRPDVDNVSVERVDLEARLDTIKAEIDFL
KEVYAAELDALRGQIIDGSMTVDLGGAPDIDFQSILDEVRAQYEALALQSKQETQGWYDTKVQGLAQQQGQAGRGLDDARAELSKMTSELKRLQ
AQIEAAKARNAQLQDQLTAVEERGVKQLEAKQEEITKLEEELTTLRSKIKEQMVEYQALMAVKMALDVEIGAYRKLLEGE
>Pm-krt-XP_032807055.1
EKNQLMGLNDRFANYIQNVRILERKNKELQVSLKRMRIH--S----
GQDSQLDNLCAQYEVLLKRQIQALIEEKRRLANDQEQAHINMEQMKIKYETEIRERTQIENEFVVLKQEADQVYMQKVELEARLTGLTDDIDFY
RKVYEREIRELEARMVKVDVVVEVDSSPGLDLDTYLAEVREQYMKQAGRIREELKISFETKLDANRNNEVKINDDMRLVTVEISEVRRNMQRYK
AELEALKQQCLALERAIALAEEKGKESMRQLTEKRQLLEVTIMEQKNKLTGHHRSYQELMNVKLALDMEIIAYRKLLEGE
>human_KRT80
EKEEMKALNDKFASLIGKVQALEQRNQLLETRWSFLQGQ--D----
SAIFDLGHLYEEYQGRLQEELRKVSQERGQLEANLLQVLEKVEEFRIRYEDEISKRTDMEFTFVQLKKDLDAECLHRTELETKLKSLESFVELM
KTIYEQELKDLAAQVKDVSVTVGMDSRCHIDLSGIVEEVKAQYDAVAARSLEEAEA-
SRSQLEEQAARSAEYGSSLQSSRSEIADLNVRIQKLRSQILSVKSHCLKLEENIKTAEEQGELAFQDAK-
KLAQLEAALQQAKQDMARQLRKYQELMNVKLALDIEIATYRKLVEGE
>Cc-krt-XP_041056079.1
EKEEIKGLNNKFSDVISRVRKLEQENQVLETRWELLQQT--D----
TYKSNADKIINMFCNKLKQQLTDLERERDRLKGQITQTEQMVEDFKGKYEDEINSRTSLENEFVILKKEVDDAYFQKVELESKLEYLTNLIEFL
KLLYAEEIQELQSQVQSTAVTLKVNNSRQLDMKQMIEDMKRQHAEIAARNKAEAETWYQNKLLELENDKSRQNAELRNAKNEFADLTRYLQKMR
SDADGLQNQRASMESNIGEAEERGQIAINEAKNRINQLQESLRKANTDMAEQMREHQELLNSTMALDMEIATYRKLLDGE
>Pm-krt-XP_032836208.
EKNAISHLNDRFASFIDKVRYLEQENKVLEAQWGCLQAR-------
VCTSDLDSMFETYAQAIKRQLDCVLADRPRLETELHQTRALADEHKLKYEAEVTGREAAEANFVAIKREADDNYMGKVQMETRVGQLSDELHFI
KELFACEMQEMEERIRDMNVTIDLDTGCNFDLSSLIAEVRANYEMVAARSREEVECWYKSKMDDMAEASERHCIELRCTKNEILELSSMLQRLA
CEIDGLKTQRYQLESSIQEAETRGELNAHEARDATARVEMELSQAKADMARHVRDYQELMNVKLALDIEIATYRKLLEGE
>Cc-krt-XP_041036177.1
EKDQLKTLNNRFAGFINKVRHLEQQNKILETKWSLLQQQ--G----
HYSSSNEELYKAYINALSRQLESFKQDKMRLDSELQQMHGTVTGLKMQYEDELNNRAGMENDFVTLKKTVDDAYLTKHQLGDKLDGFVSELDFL
RQVYDEDIRELQAQVKDVAVTVELDNTRELDLNELVANMKEQYEILAAKGRDEAQAEYRTKFNQLSQSTGKYDDDLRLMKNDITETSRRIAKMN
SEIDSLKGQRIGLEAAITEAEERGDISVKNIRAKIAGLEDSIFKAKQDIARKLREYQDLLNVKMGLDFEIATYRKMLEGE
>Pa-KRT-T2-4-XP_043939774.1
EKEQIKTLNNTFANFIDKVRILEQKNAVLRTKWELLQQQDGGAAGRGGRNDLTPIYKAHIQNLQKQLDFQNNERQRLENENNAMQQTIDNFRNR
YEDEINLHFDAENEFVVLKKEVDAAYIDKINLEANAEKLTDEMEFLRNLHALEKQAMHSNIEDIKAIVQINNNREFDLQGILDIYKAQYDEMGK
RTRGDSEQYYQKKYRELEQSAGKSGDDIKNTKHEIAELQRMIRKLRNEIEKVNQQCATLESQIAHAEEHGEQALQDARAKLAELQTALQKAKED
MAFKLQQYQELMNIKMQLDMEIATYNKLLCGE
>Pa-KRT-T2-3-XP_043939773.1
EKEEIKTLNNKFAGFINKVRSLEQTNAVLRTKWELLQQQCSGGSGTGGRNDLTPVYQAYISNMKRNIDTLMSDKGRLENELQNMQGCVEDFKSK
YEEEINLRTNAENEFVKLKKDVDAAFMDKVRLEAQADSLRDELEFRKTLCAQELQVVQSSVEDINAIIQMNNNRELDMKDMIANYKAQLDVITQ
NNRADIDNQFKQKLDDLTFQASRQGDDVKSTKEEISNLNRTIKQIQNEIENVKRQCGNLETGIASAEERGEIAVQDARAKLADMEAALQKAKQD
LANMLRQYQELMNVKLGLDIEIATYMKLLTGE
>Pm-krt-XP_032821562.1
EKDQIRGLNDRFANFIDKVRSLEQQRSVLDAQWKVLQAK--G----
EDKSNLEDIYQEYIRGLRRQLEMLQENKEHLQSDVGHMQGVVEEFKNKYETELNNRNHAENEFVLIKKDFDDAHLNKVELEARLEGLTDEIDFL
RRIYEEELRELHAQMNNISLTVEVDTNRHFNMDDIVASVRSQYEALAQQSRQEAEDFYRVKFEDINASADKSNEDIRNSKQELNDLLRTIKNLT
SEVQRLKQQRGQAERAVAEAEDLGEQALKDAKKRIADLEQELADSRRQMAQHVRDYQELMNVKLALDIEIATYGKLLEGE
>Pm-krt-XP_032826365.1
EKEEIKMLNNRFANFIDKVRILEQQNKVLEAQWYVLQDK--G----
KVDCNLDELFEVYIEELRRQLQNLGHNGARMNSDLQMIKHAGKDFSSKYEVEVAAHKKKVEEHELVKKEAVEASFVKIELEAKFQGLIEDIHFL
```

REAFAQELRELEAQIKNTNVLVEIDNRRSLDMDGIISEVRKQYEFIMSQSQAEAEAIYKQKFDKLRSESDRSDEELRKAKAEINDIKRQIHQIN
SELDILSKQREKLEAAISEREEGGAKNIGEAKAAVAQLEEALAKAKHDMVKHVRDFTDLMNIKLALDTEILTYRKLLEGE
>Pm-krt-XP_032801610.1
EKEQIKKVNDRFVRFIEKVGILEQQNKVLEVQWLLLQDR--GAR--
GSKVEASVFFQSQIHGLQRQLDTLERDKQRRQGELRLTQGLVEDFKNKYEEDKSIRNKAENEFVAVKQDFDDSCLVKAELEARLEGLKDEINFL
RGIFEKELRDLETQIKDTLVSAGVDTSRSIDVQGMISDVHAQYELIAAKSQAEANEFYRKKYDLLSLSADPTDQEIRSNRNEMNNLHRQIQRVR
GESDALKKQRASLQAAIAGAVDRGGLSVCEAKERIARLEEELHKDKQQKVQRVREYQELMNVKLALDIEIATYGRLLEGE
>Pm-krt-XP_032812943.
EKEAIKHLNDRFANFIDKVRFLEQQNKVLEAQWYALQEK--T----
TTGSSVDEMFEAYINGLRRQLDGLGHENGRANGDLGQMQAVVEDFMSKYEAEITARIHKENEYVVIKKDVDAANVIKIELEAKMQGLVEDINFL
REIFAQELSELEAQIKHTNVLVEIDNSRNLDVDGIIADVRHQYETIAARSRAEADAFYQDKFDRLHADSGRNDEEMRVIRTEINDNNRHIHRMR
AEIDALKKQRAKLEAAIAEAEGRGEADIREAKAAIAQLEEEIHKAKQEMARHVREYQELMNVKLALDIEIVTYRKLLEGE
>Pm-krt-XP_032812945.1
EKEAIKHLNDRFANFIDKVRFLEQQNKVLEAQWYALQEK--T----
TTGSSVDEMFEAYINGLRRQLDGLGHENGRANGDLGQMQAVVEDFRAKYEAEISLRTEREDEFVFAKKDVDGAYLSKVELEAKLDGLQEDINFM
RSIFAQELSELEAQIKHTNVLVEIDNSRNLDVDGIIADVRHQYETIAARSRAEADAFYQDKFQSLHSDSGRNDEELRVTRNEITELNRQMQRFR
AEMEALKKQRAKLESAIAEAEGRGEADIREAKAAIAQLEEEIHKAKQEMARHVREYQELMNVKLALDIEIVTYRKLLEGE
>Pm-krt-XP_032812944.1
EKEAIKHLNDRFANFIDKVRFLEQQNKVLEAQWYALQEK--T----
TTGSSIDEMFEAYINGLRRQLDGLGHDKGQLNGNLGQMQAVVEDFRAKYEAEINNRNAAEGEFVVVKKDVDGAYLSKVELETRLQGLVDEINFL
RSIFTQELSELEAQIKHTNVLVEIDNSRNLDVDGIIADVRQQYETIAARSRAEADAFYQDKFQSLHSDSGRNDEELRMTRNEITELNRQMQRFR
AEMEALKKQRAKLESAIAEAEGRGEADIREAKAAIAQLEEEIHKAKQEMARHVREYQELMNVKLALDIEIVTYRKLLEGE
>Pm-krt-XP_032812946.1
EKDQIKGLNDRFANFIDKVRFLEQQNKVLEAQWRALHER--G----
TVVSGLDGLFDAYTRGLKQQLESLSNNKLGLQNELQAMQGHVEDFKTKYEEEINTRTERENEFVVVKKDVDDAFLNKVELEAKLEALQDEIEFL
KKIFAEELRQLESQIQDTILTVEIDTSRNLDVDGLIADVRAQYEAIAAKSKAESDAFYKSKFADLNAMSGKGDDELRQSRSEIADINRQVQRIK
AEIEALKKQRAQLEAAIAEAEGRGELAIKEAKESIARLEEELHQAKQDMAKHVREYQELMNVKLALDIEIATYRKLLEGE
>Pm-krt-XP_032801611.1
EKEQIKGLNDRFANFIEKVRFLEQQNKVLEAQWRALQER--G----
SGGSNLDSLFEAYINGLKQHLDGLGNEKQRLQGELQHMQGAVEDFKAKYEEEINTRTQRENEFVVVKKEVDDAFLNKVELEARLDGLNDEIEFL
KKIFAEELRQLESQIKDTSLFVTVDTSRNLDVDGLIADVRAQYEAMARKSQAEADDFYKKKISELSSSAGKGDDDVRNSRNEINELNRQMQRIK
AEIEALKKQRAQLEAAISEAEGRGEMSIKEAKETIARLEEELHKAKQQMAQHVREYQELMNVKLALDIEIATYRKLLEGE
>Dr-krt-XP_003199098.1
ETQQMKTLNNRFASFIDKVRKLEQENKLLETKWRLLQKE--T----
KAESKLEPMLKNYSTSLQMQLERVKKDKEQLDNELRKAHAQVQEQKQRYEDEIGNRNKAENAFVLLKKDVDTTYLGKIALEEKLETIQEELNFF
KSFYEQELEELRDEVKDTSVVVQMDNSRNLNMEKILADVKSQYEEISACSRREAEAWYKNKFDLVSSQANQCSTELKNNKGAIDELKRKIQRLQ
NDITSAKSQCDNVEEKIKEAERDGEEAVLDATEQIRLLEEALQKAKKEMARQLRDYQELMNLKLALDIEIATYKKLLEGE
>Cc-krt-XP_041036178.1
EKEQIKGLNNQFARFIDKVRTLEQRNKQLEMHWKLLQEK--G----
VRSSNIDSVFQVYSNNLRQQIDALAQEKIRLQAELEGMQGLVEDYKTKYEDEINLRTERENEFVMIKKDVDESYLNKVDLEAKLESLTDEINFL
KDIFEEEIHELQAQMKNTAITLEVDTSRKLDLSSILDDVRSQYEGMVVKIRQDTMSSWEGKFNDMKKNSGKHADELRVRKTEISDMQRQAGRLN
AEIQALNKQKEQLEAAIAEAEERGELALMEAKKRIAELQEVVDNANKEMIRQVREYQDLMNTKLALDIEIATYKKLLEGE
>Cc-krt-XP_041036172.1
ESRQIKGLNNQFVKFIDQVRYLEQINKKLEVKLNLLKEQ--G----
EYKSNIDSMFQTYIDNLKRQLETLGQEKLRFEADLVQMQGLVEDFKGKYEDEINKRTEMENEFVLVKKDVDDSYMSKVELEAKLESLTDEIDFL
RSIFEEEIRELQAQIQNTSVSVQLDSGPKLDIEQLITEAKIQYENMANQSRKEAEIWKQTKMQELSMSSGQYGDELRVTKDEVKQMNQRIRALN
NDIESLRNERLKLEALIKEAEERGELSLRGAKDRIEDLKTALSNAQQQLMKQVQEYEALLQVKLYLDVEISTYKSLLEGE
>Cc-krt-XP_041036175.1
ESRQIKGLNNQFVKFIDQVRYLEQSNKKLEVKLNLLKEQ--G----
EYKSNIDSMFQTYIDNLKRQLETLGQEKLRFEADLVQMQGLVEDFKGKYEDEINKRTEMENEFVLVKKDVDDSYMSKVELEAKLESLTDEIDFL
RSIFEEEIRELQAQIQNTSVSVQLDSGPKLDIEQLITEAKIQYENMANQSRKEAEIWKQTKMQELSMSSGQYGDELRVTKDEVKQMNQRIRALN
NDIESLRNERLKLEALIKEAEERGELSLRGAKDRIEDLKTALANAQQQLMKQVQEYEALLQVKLYLDVEISTYKSLLEGE
>Cc-krt-XP_041036173.1
ESRQIKGLNNQFVKFIDQVRYLEQINKKLEVKLNLLKEQ--G----
EYKSNIDSMFQTYIDNLKRQLETLGQEKLRFEADLVQMQGLVEDFKGKYEDEINKRTEMENEFVLVKKDVDDSYMSKVELEAKLESLTDEIDFL
RSIFEEEIRELQAQIQNTSVSVQLDSGPKLDIEQLIAEAKIQYENMANQSRKEAEIWKQTKMQELSMSSGQYGDELRVTKDEIKQMNQRIRALN
NDIESLRNERLKLEALIKEAEERGELSLRGAKDRIEDLKTALANAQQQLMKQVQEYEALLQVKLYLDVEISTYRSLLEGE
>Cc-krt-XP_041036176.1
ESRQIKGLNNQFVKFIDQVRYLEQINKKLEVKLNLLKEQ--G----
EYKSNIDSMFQTYIDNLKRQLETLGQEKLRFEADLVQMQGLVEDFKGKYEDEINKRTEMENEFVLVKKDVDDSYMSKVELEAKLESLTDEIDFL
RSIFEEEIRELQAQIQNTSVSVQLDSGPKLDIEQLIAEAKIQYENMANQSRKEAEIWKQTKMQELSMSSGQYGDELRVTKDEIKQMNQRIRALN
NDIESLRNERLKLEALIKEAEERGELSLRGAKDRIEDLKTALANAQQQLMKQVQEYEALLQVKLYLDVEISTYRSLLEGE
>Cc-krt-XP_041036174.1
ESRQIKGLNNQFVKFIDQVRYLEQINKKLEVKLNLLKEQ--G----
EYKSNIDSMFQTYIDNLKRQLETLGQEKLRFEADLVQMQGLVEDFKGKYEDEINKRTEMENEFVLVKKDVDDSYMSKVELEAKLESLTDEIDFL

RSIFEEEIRELQAQIQNTSVSVQLDSGPKLDIEQLIAEAKIQYENMANQSRKEAEIWKQTKMQELSMSSGQYGDELRMTKDEVKQMNQRIRALN
NDIESLRNERLKLEALIKEAEERGELSLRGAKDRIEDLKTALANAQQQLMKQVQEYEALLQVKLYLDVEISTYKSLLEGE
>human_KRT78
ETQEIRTLNNQFASFIDKVRFLEQQNKVLETKWHLLQQQ--GLS--
GSQQGLEPVFEACLDQLRKQLEQLQGERGALDAELKACRDQEEEYKSKYEEEAHRRATLENDFVVLKKDVDGVFLSKMELEGKLEALREYLYFL
KHLNEEELGQLQTQASDTSVVLSMDNNRYLDFSSIITEVRARYEEIARSSKAEAEA-
YQTKYQELQVSAQLHGDRMQETKVQISQLHQEIQRLQSQTENLKKQNASLQAAITDAEQRGELALKDAQAKVDELEAALRMAKQNLARLLCEYQ
ELTSTKLSLDVEIATYRRLLEGE
>Pa-KRT80L2-XP_043939768.1
EKDQIMDLNNKFAARIDKVRFLEQQNKMLETKWSLLQEQ--K----
TQKCNVEPMFEAFINGLRRQVDNINSDRDKLNSELRNMQDLVEDFKNKYEDEINRRTGLENEFVTIKKDVDAAYLTKADLDDKNNSLADEINFL
RAVHDEEIRCMQSQIQDVSVIVELNNNRGLDMDSIIADVKSQYEAYLSKSRQEAEDTYKSKYDELAKQAGQFGDDLRLTKNEIAELNRYISKLN
LEIDSLRNQNSGLESAIVDAEGRGDKAVNDAKAKIADLDAALQKAKQDMANQVRMYNDIMNAKLRLDMEIATYRKLLEGE
>Pa-KRT80L1-XP_043939767.1
EKDEIKTLNNKFASFIDKVQVLEKQNKLLETKWALLQEQ--EQK--
KVKSNIEPMLESFINSLKKQLDVINDEKEKLEKECKQMQDVVEDYKTRYEDEICKRTSGENEFVMMKKDVDSAFMVKSELTDKVDRLTDEINFL
RQLYDEQLRELQNDINDVSVIVEVDNRRNLDLDGIVADVTSQYEAMLAKSRDEVEACHKAKITEISSAIGKYDDELRNTKSEIHELNRYIQRLN
AEIESLKNQRASLELAIGEAEGHGEMAVADAKSRISELEAALQKAKQDMATQIREYQDLMNIKLSLDIEIATYRKLLEGE
>Pa-KRT-T2-1-XP_043939769.1
EKEQIKTLNNKFAGFIEKVRRLEQTNKMLETKWALLQQQ--QQGG-
PDTASLEQLYQAFINNLKRQMDNLCHEKDRLTSDLRNMENVAEDFKKKYEDEINSRTTAENEFVTLKKDVDAAFMQKCGLDAKISALEEEIAFL
KELHNAELAQMNTCVKDITAIIEMDNRRDFDMKGMLETVKAQYDDMVKNSRAEAEAFYQSKYEELVNSTNQYENDIRNTKTEIAELNRNITRLR
NEIESLKRQNAHLDASIAEAEERGEMTLKDAKLKLTNLEAALQKAREDMAHLTRDYQELMNIKLALDMEIACYRKLLEGE
>Pa-KRT-T2-2-XP_043939771.1
EKNEIKTLNNKFAGFIEKVRRLEQTNKMLETKWALLQQQ--QQGG-
PDSANLDATYQSYIHNLRRQLDNLNHDKDRLNSELHNMENIVDDFKKKYEDEINNRTAAENEFVTLKKDVDAAFLQKCELEAKLNGLQDEINFT
RELHNAELEQMNLYVKDITAVIEMDNRRDFDMTAITEVIKAQYDDMVKNSRAEAEAFYQSKYDELVTSTNRCGDDLRNTKTEIQEINRNITRVR
NEIENIKRQNANLETAIAEAEERGETAVKDARLKLAELEAALQKAKEDMARLTRDYQELMNIKLALDMEIACYRKLLEGE
>human_KRT82
EKEQIKCLNNRFASFINKVRFLEQKNKLLETKWNFMQQQ--R----
CCQTNIEPIFEGYISALRRQLDCVSGDRVRLESELCSLQAALEGYKKKYEEELSLRPCVENEFVALKKDVDTAFLMKADLETNAEALVQEIDFL
KSLYEEEICLLQSQISETSVIVKMDNSRELDVDGIIAEIKAQYDDIASRSKAEAEA-
YQCRYEELRVTAGNHCDNLRNRKNEILEMNKLIQRLQQETENVKAQRCKLEGAIAEAEQQGEAALNDAKCKLAGLEEALQKAKQDMACLLKEYQ
EVMNSKLGLDIEIATYRRLLEGE
>human_KRT84
EKEQIKTLNNKFASFIDKVRFLEQQNKLLETKWSFLQEQ--K----
CIRSNLEPLFESYITNLRRQLEVLVSDQARLQAERNHLQDVLEGFKKKYEEEVVCRANAENEFVALKKDVDAAFMNKSDLEANVDTLTQEIDFL
KTLYMEEIQLLQSHISETSVIVKMDNSRDLNLDGIIAEVKAQYEEVARRSRADAEA-
YQTKYEEMQVTAGQHCDNLRNIRNEINELTRLIQRLKAEIEHAKAQRAKLEAAVAEAEQQGEATLSDAKCKLADLECALQQAKQDMARQLCEYQ
ELMNAKLGLDIEIATYRRLLEGE
>human_KRT85
EKEQIKSLNSRFAAFIDKVRFLEQQNKLLETKWQFYQNQ--R----
CCESNLEPLFSGYIETLRREAECVEADSGRLASELNHVQEVLEGYKKKYEEEVALRATAENEFVVLKKDVDCAYLRKSDLEANVEALVEESSFL
RRLYEEEIRVLQAHISDTSVIVKMDNSRDLNMDCIIAEIKAQYDDVASRSRAEAES-
YRSKCEEMKATVIRHGETLRRTKEEINELNRMIQRLTAEIENAKCQRAKLEAAVAEAEQQGEAALSDARCKLAELEGALQKAKQDMACLLKEYQ
EVMNSKLGLDIEIATYRRLLEGE
>human_KRT83
EKEQIKSLNSRFAAFIDKVRFLEQQNKLLETKLQFYQNR--E----
CCQSNLEPLFAGYIETLRREAECVEADSGRLASELNHVQEVLEGYKKKYEEEVALRATAENEFVALKKDVDCAYLRKSDLEANVEALIQEIDFL
RRLYEEEIRILQSHISDTSVVVKLDNSRDLNMDCIVAEIKAQYDDIATRSRAEAES-
YRSKCEEMKATVIRHGETLRRTKEEINELNRMIQRLTAEVENAKCQNSKLEAAVAQSEQQGEAALSDARCKLAELEGALQKAKQDMACLIREYQ
EVMNSKLGLDIEIATYRRLLEGE
>human_KRT81
EKEQIKSLNSRFAAFIDKVRFLEQQNKLLETKLQFYQNR--E----
CCQSNLEPLFEGYIETLRREAECVEADSGRLASELNHVQEVLEGYKKKYEEEVSLRATAENEFVALKKDVDCAYLRKSDLEANVEALIQEIDFL
RRLYEEEILILQSHISDTSVVVKLDNSRDLNMDCIIAEIKAQYDDIVTRSRAEAES-
YRSKCEEMKATVIRHGETLRRTKEEINELNRMIQRLTAEVENAKCQNSKLEAAVAQSEQQGEAALSDARCKLAELEGALQKAKQDMACLIREYQ
EVMNSKLGLDIEIATYRRLLEGE
>human_KRT86
EKEQIKSLNSRFAAFIDKVRFLEQQNKLLETKLQFYQNR--E----
CCQSNLEPLFEGYIETLRREAECVEADSGRLASELNHVQEVLEGYKKKYEEEVSLRATAENEFVALKKDVDCAYLRKSDLEANVEALIQEIDFL
RRLYEEEIRVLQSHISDTSVVVKLDNSRDLNMDCIIAEIKAQYDDIVTRSRAEAES-
YRSKCEEMKATVIRHGETLRRTKEEINELNRMIQRLTAEVENAKCQNSKLEAAVAQSEQQGEAALSDARCKLAELEGALQKAKQDMACLIREYQ
EVMNSKLGLDIEIATYRRLLEGE
>Pa-KRT-T2-7-XP_043939778.1

EKEQIKTLNNKFAGYIDKVRTLEQQNKLLQVKWELLQKQQQGG---
CSDVNLNAMYEAYIGNLKRQLDSLQNDKYRLESELKNMQNIVEDYKCKYEDEINKRNGAENDFVMLKKDVDAAYMNKVELEAKLDGLNDYMKFL
RAIHEAELQEMQTQVQDISAIIQMDNNRQLDLDNVIAEAKTCFANMAQQSRAEAESYYQAKYDQLASTAGNYGDELRTTKAEIQDLQRMITRLT
NEIESIKNQRATLETTIAEAEERGEMALKEAREKKAELDAALQKAKQDLAKLLHDVQEVMNIKLGLDIEIATYRKLLEGE
>Pa-KRT-T2-5-XP_043939775.1
EKEQIKTLNNKFVGFIEKVRLLEQQNKMLTVKWELLQNQ--
QQGGAPGGMNLNNLYEVYISNLRRQMDALSNDKSRLESDLKNMQGQVEDFKNRYEDEINKRTALENEFVVIKKDVDAAFMIKVELEEKLRALRE
EIDFLRAIYEAELQQLHTQVQDITAIVQMDNNRQLDLDNIISEVRLQYEEMANNSRAEAESFYQQKYEELASSAGKYGDDLRVTKSEIAEMTRM
ITRLTNEIESLKNQRASLEAAITEAEERGELALKDARDKKADLEAALQRAKQDMARLVHDYQELMNAKLGLDIEIACYRKLLEGE
>Pa-KRT-T2-6-XP_043939777.1
EKEQIKTLNNKFAGFIEKVRLLEQQNKMLTVKWELLQNQ--
QHGGAPGGMNLNSMYEAYISNLRRQLDGHGNDKSRLESELKNMQGQVEDFKNRYEDEINRRTTLENEFVVLKKDVDAAYLAKVELEAKRDALQD
ELNFLRTLYEAELQQFHTQVQDVTAIVQMDNNRQLDLDNLISEVKLQYEEMANKSRAEAEAFYQQKYEELASSAGQYGEDLRVTKTEIAEMTRM
ISRLTNEIESLKNQRASLEAAITEAEERGELSLKDARDKKAELEAALQRAKQDMARLVHDYQELMNAKLGLDIEIACYRKLLEGE
>human_KRT77
EREQIMVLNNKFASFIDKVRFLEQQNQVLQTKWELLQQV--NTS--
TGTNNLEPLLENYIGDLRRQVDLLSAEQMRQNAEVRSMQDVVEDYKSKYEDEINKRTGSENDFVVLKKDVDAAYVSKVDLESRVDTLTGEVNFL
KYLFLTELSQVQTHISDTNVILSMDNNRSLDLDSIIDAVRTQYELIAQRSKDEAEA-
YQTKYQELQITAGRHGDDLKNSKMEIAELNRTVQRLQAEISNVKKQIEQMQSLISDAEERGEQALQDAWQKLQDLEEALQQSKEELARLLRDYQ
AMLGVKLSLDVEIATYRQLLEGE
>human_KRT7
ESEQIKTLNNKFASFIDKVRFLEQQNKLLETKWTLLQEQ--KS---
AKSSRLPDIFEAQIAGLRGQLEALQVDGGRLEAELRSMQDVVEDFKNKYEDEINHRTAAENEFVVLKKDVDAAYMSKVELEAKVDALNDEINFL
RTLNETELTELQSQISDTSVVLSMDNSRSLDLDGIIAEVKAQYEEMAKCSRAEAEA-
YQTKFETLQAQAGKHGDDLRNTRNEISEMNRAIQRLQAEIDNIKNQRAKLEAAIAEAEERGELALKDARAKQEELEAALQRGKQDMARQLREYQ
ELMSVKLALDIEIATYRKLLEGE
>Dr-krt-XP_696736.2
EKEQIKSLNNRFATFIDKVRFLEQQNKMLETKWELLQTQ--T----
PGRSNVEPMFEAYMANLRRQMDVVNNDRTKLDGELRNMQGLVEDFKHKYEDEINKRNNLENDFVILKKDVDSAYLVKADLEDKVGALTDEINFL
RTIYDEELRELQASIQDTSVVVQMDNSRNLNMDHIVAEVKSQYEEIAARSREEAESWYKSKFDQMASQANQYNDELRNTRGEIGDINRMISRLQ
SEIEVIKAQRANLENQIAEAEDRGEMTVKEAKGRIKDLEEALQRAKQDMARQLREYQELMNVKLALDIEIATYRKLLEGE
>Dr-krt-NP_001076492.1
EKEQIKTLNNRFASFIDKVRYLEQQNKMLETKWSVLQEQ--T----
SRQSNIDMMFEGYIAGLRRQLDSLGNEKQRLESGLRDMNTLVEDFKSKYEDEINKRSDSDNEFVLLKKNADDSYLSKVELDCKLDALSEEINFL
WQIYEAELHELQTQIKDTSVVVEMDNSRNLDMESIVAEVRAQYEEIASRSRAEAESWYQQKYEEMQLTATKHGDDLKNSKAEISEYNRRIARIH
SEMELIKGQFSNHEAQIKEAEERGELAVKDARLRLQELEAALLRAKQDMARQVREYQSLMNIKLALDIEIATYRKLLEGE
>Pa-KRT-T2-8-XP_043939779.1
EKEQIKTLNNQFAGFIDKVRYLEQQNKLLETKWQLLQNQ--AT---
PSRSNLDSMFEAYISNLRRQLDTLGQEKGKLEAELHNMQGLVEDFKNKYEDEINKRTDTENEFVLIKKDVDEAYMNKVELEAKLEALTDEINFL
RQIYDEEIRELQTQIQDTSVIVQMDNNRQLDLDNIIAEVKAQYEDMAKNSRAEAETYYQQKYEELASSAGRYGDDLRNTKNEIAELTRYINRLN
SDIDALKGQRTNLEAAIAEAEERGEQAVKNAQAQLQELQNALTQAKQDMAHQLREYQDLMNVKLALDIEIATYRKLLEGE
>human_KRT8
EKEQIKTLNNKFASFIDKVRFLEQQNKMLETKWSLLQQQ--K----
TARSNMDNMFESYINNLRRQLETLGQEKLKLEAELGNMQGLVEDFKNKYEDEINKRTEMENEFVLIKKDVDEAYMNKVELESRLEGLTDEINFL
RQLYEEEIRELQSQISDTSVVLSMDNSRSLDMDSIIAEVKAQYEDIANRSRAEAES-
YQIKYEELQSLAGKHGDDLRRTKTEISEMNRNISRLQAEIEGLKGQRASLEAAIADAEQRGELAIKDANAKLSELEAALQRAKQDMARQLREYQ
ELMNVKLALDIEIATYRKLLEGE
>Dr-krt-NP_956374.1
EKEQIKTLNNRFASFIDKVRFLEQQNKMLETKWSLLQNQ--T----
ATRSNIDAMFEAYIANLRRQLDSLGNDKMKLEADLHNMQGLVEDFKNKYEDEINKRTECENEFVLIKKDVDEAYMNKVELEAKLESLTDEINFL
RQIFEEEIRELQSQIKDTSVVVEMDNSRNLDMDAIVAEVRAQYEDIANRSRAEAEMWYKSKYEEMQTSANKYGDDLRSTKTEIADLNRMIQRLQ
SEIDAVKGQRANLENQIAEAEERGEMAVRDAKGRIKDLEDALQRAKQDMARQIREYQDLMNVKLALDIEIATYRKLLEGE
>Dr-krt-NP_571584.2
EKEQIKTLNNRFASFIDKVRFLEQQNKMLETKWSLLQEQ--T----
TTRSNIDAMFEAYISNLRRQLDGLGNEKMKLEGELKNMQGLVEDFKNKYEDEINKRASVENEFVLLKKDVDAAYMNKVELEAKVDALQDEINFL
RAVYEAELRELQSQIKDTSVVVEMDNSRNLDMDSIVAEVRAQYEDIANRSRAEAESWYKQKFEEMQSTAGQYGDDLRSTKAEIAELNRMIARLQ
NEIDAVKAQRANLEAQIAEAEERGELAVKDAKLRIRELEEALQRAKQDMARQVREYQELMNVKLALDIEIATYRKLLEGE
>Dr-krt-XP_017209104.1
EKEQIKTLNNRFASFIDKVRFLEQQNKVLETKWSLLQEQ--T----
TTRSNIDAMFEAYIANLRRQLDGLGNEKMKLEGELKNMQNLVEDFKNKYEDEINKRAAVENEFVLLKKDVDAAYMNKVELEAKVDSLQDEINFL
RAIFEEELRELQSQIKDTSVVVEMDNSRNLDMDAIVAEVRAQYEDIANRSRAEAESWYKQKFEEMQSSAGKYGDDLRNTKAEIADLNRMISRLQ
NEIEAVKGQRANLEAQIAEAEERGELAVKDAKLRIKDLEDALQRAKQDMARQVREYQELMNVKLALDIEIATYRKLLEGE
>human_KRT74
EREQIKVLNDKFASFIDKVRFLEQQNQVLETKWELLQQL--DLN--
NCKKNLEPILEGYISNLRKQLETLSGDRVRLDSELRSMRDLVEDYKKRYEVEINRRTTAENEFVVLKKDADAAYAVKVELQAKVDSLDKEIKFL

```
KCLYDAEIAQIQTHASETSVILSMDNNRDLDLDSIIAEVRMHYEEIALKSKAEAEA-
YQTKIQELQLAASRHGDDLKHTRSEMVELNRLIQRIRCEIGNVKKQRASLETAIADAEQRGDNALKDAQAKLDELEGALHQAKEELARMLREYQ
ELMSLKLALDMEIATYRKLLEGE
>human_KRT72
EREQIKALNNKFASFIDKVRFLEQQNQVLETKWNLLQQL--DLN--
NCRKNLEPIYEGYISNLQKQLEMLSGDGVRLDSELRNMQDLVEDYKKRYEVEINRRTAAENEFVVLKKDVDAAYMNKVELQAKVDSLTDEIKFF
KCLYEGEITQIQSHISDTSIVLSMDNNRDLDLDSIIAEVRAQYEEIALKSKAEAET-
YQTKIQELQVTAGQHGDDLKLTKAEISELNRLIQRIRSEIGNVKKQCADLETAIADAEQRGDCALKDARAKLDELEGALHQAKEELARMLREYQ
ELVSLKLALDMEIATYRKLLESE
>human_KRT71
EREQIKALNNKFASFIDKVRFLEQQNQVLETKWELLQQL--DLN--
NCKNNLEPILEGYISNLRKQLETLSGDRVRLDSELRNVRDVVEDYKKRYEEEINKRTAAENEFVLLKKDVDAAYANKVELQAKVESMDQEIKFF
RCLFEAEITQIQSHISDMSVILSMDNNRNLDLDSIIDEVRTQYEEIALKSKAEAEA-
YQTKFQELQLAAGRHGDDLKNTKNEISELTRLIQRIRSEIENVKKQASNLETAIADAEQRGDNALKDARAKLDELEGALHQAKEELARMLREYQ
ELMSLKLALDMEIATYRKLLESE
>human_KRT73
EREQIKVLNNKFASFIDKVRFLEQQNQVLETKWELLQQL--DLN--
NCKNNLEPILEGYISNLRKQLETLSGDRVRLDSELRSVREVVEDYKKRYEEEINKRTTAENEFVVLKKDVDAAYTSKVELQAKVDALDGEIKFF
KCLYEGETAQIQSHISDTSIILSMDNNRNLDLDSIIAEVRAQYEEIARKSKAEAEA-
YQTKFQELQLAAGRHGDDLKHTKNEISELTRLIQRLRSEIESVKKQCANLETAIADAEQRGDCALKDARAKLDELEGALQQAKEELARMLREYQ
ELLSVKLSLDIEIATYRKLLEGE
>human_KRT2
EREQIKTLNNKFASFIDKVRFLEQQNQVLQTKWELLQQM--NVG--
TRPINLEPIFQGYIDSLKRYLDGLTAERTSQNSELNNMQDLVEDYKKKYEDEINKRTAAENDFVTLKKDVDNAYMIKVELQSKVDLLNQEIEFL
KVLYDAEISQIHQSVTDTNVILSMDNSRNLDLDSIIAEVKAQYEEIAQRSKEEAEA-
YHSKYEELQVTVGRHGDSLKEIKIEISELNRVIQRLQGEIAHVKKQCKNVQDAIADAEQRGEHALKDARNKLNDLEEALQQAKEDLARLLRDYQ
ELMNVKLALDVEIATYRKLLEGE
>human_KRT4
EREQIKLLNNKFASFIDKVQFLEQQNKVLETKWNLLQQQ--TTT--
TSSKNLEPLFETYLSVLRKQLDTLGNDKGRLQSELKTMQDSVEDFKTKYEEEINKRTAAENDFVVLKKDVDAAYLNKVELEAKVDSLNDEINFL
KVLYDAELSQMQTHVSDTSVVLSMDNNRNLDLDSIIAEVRAQYEEIAQRSKAEAEA-
YQTKVQQLQISVDQHGDNLKNTKSEIAELNRMIQRLRAEIENIKKQCQTLQVSVADAEQRGENALKDAHSKRVELEAALQQAKEELARMLREYQ
ELMSVKLALDIEIATYRKLLEGE
>human_KRT1
EREQIKSLNNQFASFIDKVRFLEQQNQVLQTKWELLQQV--DTS--
TRTHNLEPYFESFINNLRRRVDQLKSDQSRLDSELKNMQDMVEDYRNKYEDEINKRTNAENEFVTIKKDVDGAYMTKVDLQAKLDNLQQEIDFL
TALYQAELSQMQTQISETNVILSMDNNRSLDLDSIIAEVKAQYEDIAQKSKAEAES-
YQSKYEELQITAGRHGDSVRNSKIEISELNRVIQRLRSEIDNVKKQISNLQQSISDAEQRGENALKDAKNKLNDLEDALQQAKEDLARLLRDYQ
ELMNTKLALDLEIATYRTLLEGE
>human_KRT79
EREQIKTLNNKFASFIDKVRFLEQQNKVLETKWALLQEQ--
GQNLGVTRNNLEPLFEAYLGSMRSTLDRLQSERGRLDSELRNVQDLVEDFKNKYEDEINKHTAAENEFVVLKKDVDAAYMGRMDLHGKVGTLTQ
EIDFLQQLYEMELSQVQTHVSNTNVVLSMDNNRNLDLDSIIAEVKAQYELIAQRSRAEAEA-
YQTKYEELQVTAGKHGDNLRDTKNEIAELTRTIQRLQGEADAAKKQCQQLQTAIAEAEQRGELALKDAQKKLGDLDVALHQAKEDLTRLLRDYQ
ELMNVKLALDVEIATYRKLLESE
>human_KRT3
EREQIKTLNNKFASFIDKVRFLEQQNKVLETKWNLLQQQ--
GTSSISGTNNLEPLFENHINYLRSYLDNILGERGRLDSELKNMEDLVEDFKKKYEDEINKRTAAENEFVTLKKDVDSAYMNKVELQAKVDALID
EIDFLRTLYDAELSQMQSHISDTSVVLSMDNNRSLDLDSIIAEVRAQYEDIAQRSKAEAEA-
YQTKLGELQTTAGRHGDDLRNTKSEIIELNRMIQRLRAEIEGVKKQNANLQTAIAEAEQHGEMALKDANAKLQELQAALQQAKDDLARLLRDYQ
ELMNVKLALDVEIATYRKLLEGE
>human_KRT76
EREQIKTLNNKFASFIDKVRFLEQQNKVLETKWELLQQQ--TTG--
SGPSSLEPCFESYISFLCKQLDSLLGERGNLEGELKSMQDLVEDFKKKYEDEINKRTAAENEFVGLKKDVDAAFMNKVELQAKVDSLTDEVSFL
RTLYEMELSQMQSHASDTSVVLSMDNNRCLDLGSIIAEVRAQYEEIAQRSKSEAEA-
YQTKLGELQTTAGRHGDDLRNTKSEIMELNRMIQRLRAEIENVKKQNANLQTAIAEAEQRGEMALKDANAKLQDLQTALQKAKDDLARLLRDYQ
ELMNVKLALDVEIATYRKLLEGE
>human_KRT75
EREQIKTLNNKFASFIDKVRFLEQQNKVLETKWALLQEQ--GSR--
TVRQNLEPLFDSYTSELRRQLESITTERGRLEAELRNMQDVVEDFKVRYEDEINKRTAAENEFVALKKDVDAAYMNKVELEAKVKSLPEEINFI
HSVFDAELSQLQTQVGDTSVVLSMDNNRNLDLDSIIAEVKAQYEDIANRSRAEAES-
YQTKYEELQVTAGRHGDDLRNTKQEISEMNRMIQRLRAEIDSVKKQCSSLQTAIADAEQRGELALKDARAKLVDLEEALQKAKQDMARLLREYQ
ELMNIKLALDVEIATYRKLLEGE
>human_KRT5
EREQIKTLNNKFASFIDKVRFLEQQNKVLDTKWTLLQEQ--GTK--
```

```
TVRQNLEPLFEQYINNLRRQLDSIVGERGRLDSELRNMQDLVEDFKNKYEDEINKRTTAENEFVMLKKDVDAAYMNKVELEAKVDALMDEINFM
KMFFDAELSQMQTHVSDTSVVLSMDNNRNLDLDSIIAEVKAQYEEIANRSRTEAES-
YQTKYEELQQTAGRHGDDLRNTKHEISEMNRMIQRLRAEIDNVKKQCANLQNAIADAEQRGELALKDARNKLAELEEALQKAKQDMARLLREYQ
ELMNTKLALDVEIATYRKLLEGE
>human_KRT6A
EREQIKTLNNKFASFIDKVRFLEQQNKVLETKWTLLQEQ--GTK--
TVRQNLEPLFEQYINNLRRQLDSIVGERGRLDSELRGMQDLVEDFKNKYEDEINKRTAAENEFVTLKKDVDAAYMNKVELQAKADTLTDEINFL
RALYDAELSQMQTHISDTSVVLSMDNNRNLDLDSIIAEVKAQYEEIAQRSRAEAES-
YQTKYEELQVTAGRHGDDLRNTKQEIAEINRMIQRLRSEIDHVKKQCANLQAAIADAEQRGEMALKDAKNKLEGLEDALQKAKQDLARLLKEYQ
ELMNVKLALDVEIATYRKLLEGE
>human_KRT6B
EREQIKTLNNKFASFIDKVRFLEQQNKVLDTKWTLLQEQ--GTK--
TVRQNLEPLFEQYINNLRRQLDNIVGERGRLDSELRNMQDLVEDLKNKYEDEINKRTAAENEFVTLKKDVDAAYMNKVELQAKADTLTDEINFL
RALYDAELSQMQTHISDTSVVLSMDNNRNLDLDSIIAEVKAQYEEIAQRSRAEAES-
YQTKYEELQITAGRHGDDLRNTKQEIAEINRMIQRLRSEIDHVKKQCANLQAAIADAEQRGEMALKDAKNKLEGLEDALQKAKQDLARLLKEYQ
ELMNVKLALDVEIATYRKLLEGE
>human_KRT6C
EREQIKTLNNKFASFIDKVRFLEQQNKVLDTKWTLLQEQ--GTK--
TVRQNLEPLFEQYINNLRRQLDSIVGERGRLDSELRNMQDLVEDLKNKYEDEINKRTAAENEFVTLKKDVDAAYMNKVELQAKADTLTDEINFL
RALYDAELSQMQTHISDTSVVLSMDNNRNLDLDSIIAEVKAQYEEIAQRSRAEAES-
YQTKYEELQVTAGRHGDDLRNTKQEIAEINRMIQRLRSEIDHVKKQCASLQAAIADAEQRGEMALKDAKNKLEGLEDALQKAKQDLARLLKEYQ
ELMNVKLALDVEIATYRKLLEGE
```

Figure S15. Multiple sequence alignment of type II keratins used to generate the phylogeny shown in Figure S13B. The protein sequences are provided in fasta_aln format. Species: lancelet (Bf, *Branchiostoma floridae*), sea lamprey (Pm, *Petromyzon marinus*), great white shark (Cc, *Carcharodon carcharias*), zebrafish (Dr, *Danio rerio*), lungfish (Pa, *Protopterus annectens*), human (*Homo sapiens*).

Supplementary Table S1. Accession numbers of type I keratins investigated in this study

Species	Species name	Short name	Protein	Accession number	Database
Homo sapiens	Human	Human	KRT24	NP_061889	GenBank
			KRT25	XP_011522716	GenBank
			KRT26	NP_853517	GenBank
			KRT27	NP_853515	GenBank
			KRT28	NP_853513	GenBank
			KRT10	NP_001366295	GenBank
			KRT12	NP_000214	GenBank
			KRT20	NP_061883	GenBank
			KRT23	XP_047291682	GenBank
			KRT39	NP_998821	GenBank
			KRT40	NP_872303	GenBank
			KRT33A	NP_004129	GenBank
			KRT33B	NP_002270	GenBank
			KRT34	NP_001372943	GenBank
			KRT31	NP_002268	GenBank
			KRT37	NP_003761	GenBank
			KRT38	NP_006762	GenBank
			KRT32	NP_002269	GenBank
			KRT35	NP_002271	GenBank
			KRT36	NP_003762	GenBank
			KRT13	NP_705694	GenBank
			KRT15	NP_002266	GenBank
			KRT19	NP_002267	GenBank
			KRT9	NP_000217	GenBank
			KRT14	NP_000517	GenBank
			KRT16	NP_005548	GenBank
			KRT17	NP_000413	GenBank
Alligator sinensis	Chinese alligator	Alligator	HAS1	XP_006032843	GenBank
			HAS2	XP_006032844	GenBank
			KRT10L	XP_025058719	GenBank
			KRT117	XP_025070287	GenBank
			KRT12	XP_006022639	GenBank
			KRT14L1	XP_006032841	GenBank
			KRT14L2	XP_006032839	GenBank
			KRT15	XP_006022586	GenBank
			KRT18	XP_006030669	GenBank
			KRT19	XP_006022585	GenBank
			KRT20	XP_006022587	GenBank
			KRT23	XP_006022637	GenBank
			KRT24	XP_025058718	GenBank
			KRT36L1	XP_006022636	GenBank
			KRT36L2	XP_006022635	GenBank
			KRT36L3	XP_025058710	GenBank
			KRT9L1	XP_006022584	GenBank

			KRT9L2	XP_006039283	GenBank
			KRT9L4	XP_025070308	GenBank
			KRT9L5	XP_025070304	GenBank
Anolis carolinensis	Green anole lizard	Lizard	HAS1	XP_016850077	GenBank
			HAS2	NP_001280049	GenBank
			HAS3	XP_003222627	GenBank
			HAS4	XP_016850078	GenBank
			KRT10L	XP_016850071	GenBank
			KRT117	XP_008111601	GenBank
			KRT12	XP_003222621	GenBank
			KRT14L1	XP_003222513	GenBank
			KRT14L2	XP_003222514	GenBank
			KRT15	XP_008111595	GenBank
			KRT18	XP_003224490	GenBank
			KRT19	XP_008111599	GenBank
			KRT23	XP_008111584	GenBank
			KRT24	XP_008111591	GenBank
			KRT24L	XP_008111590	GenBank
			KRT36L1	NP_001280052	GenBank
			KRT36L2	NP_001280048	GenBank
			KRT9L1	XP_008111598	GenBank
			KRT9L2	XP_016850073	GenBank
Xenopus tropicalis	Western clawed frog	Frog	krt24	XP_002940698	GenBank
			krt50	XP_031750197	GenBank
			krt51	XP_031750195	GenBank
			krt53	NP_001121485	GenBank
			krt54	XP_002940703	GenBank
			krt55	NP_001011224	GenBank
			krt56	XP_004918844	GenBank
			krt57	XP_031750559	GenBank
			krt12.6	XP_002940696	GenBank
			krt12.5	NP_001116496	GenBank
			krt12.4	NP_001120056	GenBank
			krt12.3	NP_001016659	GenBank
			krt20	XP_031750289	GenBank
			krt12.2	XP_004918756	GenBank
			krt12.1	XP_031750293	GenBank
			krt12.7	XP_031750291	GenBank
			krt23	XP_031750290	GenBank
			krt34	XP_002939082	GenBank
			krt15.2	XP_002939083	GenBank
			krt15.1	XP_004918754	GenBank
			krt19	XP_012827034	GenBank
			krt9.2	XP_012808156	GenBank
			krt9.1	XP_002939076	GenBank
			krt17	XP_002939077	GenBank
			krt70	NP_001006687	GenBank

Petromyzon marinus	Sea lamprey	Lamprey	krt18.1	XP_032807760	GenBank
			krt18.2	XP_032807056	GenBank
			krt18.3	XP_032809879	GenBank
			krt18.4	XP_032814531	GenBank
			krt18.5	XP_032815632	GenBank
			krt18.6	XP_032821560	GenBank
			krt18.7	XP_032828950	GenBank
			krt18.8	XP_032832261	GenBank
			krt18.9	XP_032837300	GenBank
			krt18.10	XP_032800492	GenBank
Lethenteron reissneri	Far Eastern brook lamprey	n.a.	krt18.1	n.a. (related to XP_061434683)[1]	GenBank
			krt18.2	XP_061434523	GenBank
			krt18.3	XP_061427718	GenBank
			krt18.4	XP_061419285	GenBank
			krt18.5	XP_061419111	GenBank
			krt18.6	XP_061410630	GenBank
			krt18.7	XP_061410422	GenBank
			krt18.8	XP_061406340	GenBank
Eptatretus burgeri	Inshore hagfish	Hagfish	krt18.1	ENSEBUP00000013892	Ensembl
			krt18.2	ENSEBUP00000011392	Ensembl
			krt18.4	ENSEBUP00000017668	Ensembl
			krt18.8	n.a. (related to ENSEBUP00000010460)[2]	Ensembl
			krt18.9	n.a. (related to ENSEBUP00000019181)[2]	Ensembl
			krt18.12	ENSEBUP00000014123	Ensembl
			krt18.13	ENSEBUP00000006941	Ensembl
			krt18.14	ENSEBUP00000005546	Ensembl
			krt18.15	ENSEBUP00000005523	Ensembl
Eptatretus atami	Brown hagfish	n.a.	krt18.1	EA83790	Zenodo[4]
			krt18.2	EA01219	Zenodo[4]
			krt18.3	EA00164	Zenodo[4]
			krt18.5	EA85498[3]	Zenodo[4]
			krt18.6	EA36694	Zenodo[4]
			krt18.7	EA39268	Zenodo[4]
			krt18.8	EA02654	Zenodo[4]
			krt18.9	EA60320	Zenodo[4]
			krt18.10	EA25574	Zenodo[4]

Note:
[1] The prediction of coding sequence (cds) of the corresponding gene was corrected.
[2] The complete cds obtained from juvenile head trancriptome (SRX2541845).
[3] The complete cds was corrected with tBLASTn against the published genome (GCA_035128595.1). See Table S4.
[4] The peptide file (Pata_ah2p.pep.fa) is available at https://zenodo.org/records/10227719.

Supplementary Table S2. Accession numbers of type II keratins investigated in this study

Species	Species name	Short name	Protein	Accession number	Database
Homo sapiens	Human	Human	KRT80	NP_872313	GenBank
			KRT7	XP_011536627	GenBank
			KRT81	NP_002272	GenBank
			KRT86	XP_005268923	GenBank
			KRT83	NP_002273	GenBank
			KRT85	NP_002274	GenBank
			KRT84	NP_149034	GenBank
			KRT82	NP_149022	GenBank
			KRT75	NP_004684	GenBank
			KRT6B	NP_005546	GenBank
			KRT6C	NP_775109	GenBank
			KRT6A	NP_005545	GenBank
			KRT5	NP_000415	GenBank
			KRT71	XP_047284152	GenBank
			KRT74	NP_778223.2	GenBank
			KRT72	XP_047284279	GenBank
			KRT73	XP_047284717	GenBank
			KRT2	NP_000414	GenBank
			KRT1	NP_006112	GenBank
			KRT77	NP_778253	GenBank
			KRT76	NP_056932	GenBank
			KRT3	NP_476429	GenBank
			KRT4	NP_002263	GenBank
			KRT79	NP_787028	GenBank
			KRT78	XP_011536312	GenBank
			KRT8	NP_001243211	GenBank
			KRT18	NP_954657	GenBank
Alligator sinensis	Chinese alligator	Alligator	HBS1	XP_006031800	GenBank
			HBS2	XP_025069184	GenBank
			HBS3	XP_025069194	GenBank
			KRT5L1	XP_006031817	GenBank
			KRT5L2	XP_006031796	GenBank
			KRT5L3	XP_006031794	GenBank
			KRT5L4	XP_006031793	GenBank
			KRT7	XP_006031791	GenBank
			KRT78L1	XP_014378640	GenBank
			KRT78L2	XP_014378646	GenBank
			KRT78L3	XP_006030688	GenBank
			KRT78L4	XP_014379196	GenBank
			KRT78L5	XP_025069173	GenBank
			KRT78L6	XP_014379207	GenBank
			KRT78LT	XP_006031801	GenBank
			KRT80	XP_006031790	GenBank
			KRT84L1	XP_006031815	GenBank

			KRT84L2	XP_025069161	GenBank
			KRT8L1	XP_006030670	GenBank
			KRT8L2	XP_006030686	GenBank
Anolis carolinensis	Green anole lizard	Lizard	HBS1	XP_003217077	GenBank
			HBS2	XP_003217076	GenBank
			HBS3	XP_003217075	GenBank
			KRT5L1	XP_003217078	GenBank
			KRT5L2	XP_003217018	GenBank
			KRT5L3	XP_003217019	GenBank
			KRT5L4	XP_008102284	GenBank
			KRT7	XP_003217023	GenBank
			KRT78L1	XP_003217017	GenBank
			KRT78L2	XP_003217070	GenBank
			KRT78L3	XP_008102271	GenBank
			KRT78L4	XP_003217072	GenBank
			KRT78L5	XP_016846727	GenBank
			KRT78LT	XP_016846726	GenBank
			KRT8	XP_003224483	GenBank
			KRT80	XP_008102289	GenBank
			KRT84L1	XP_016846291	GenBank
			KRT84L2	NP_001280051	GenBank
			KRT84L3	XP_003217080	GenBank
			KRT84L4	NP_001280050	GenBank
Xenopus tropicalis	Western clawed frog	Frog	krt80	NP_001163982	GenBank
			krt7	XP_002935788	GenBank
			krt59	XP_012813652	GenBank
			krt6b	XP_031753073	GenBank
			krt61	NP_001072377	GenBank
			krt62	XP_002935776	GenBank
			krt78.1	XP_002935786	GenBank
			krt78.2	XP_002935775	GenBank
			krt78.3	XP_002935785	GenBank
			krt78.4	XP_002935784	GenBank
			krt78.5	XP_031753072	GenBank
			krt78.10	XP_031752089	GenBank
			krt78.6	XP_002935774	GenBank
			krt78.7	NP_001006716	GenBank
			krt78.8	XP_031753071	GenBank
			krt78.9	NP_001166034	GenBank
			krt8	NP_001002797	GenBank
			krt18	NP_988944	GenBank
Petromyzon marinus	Sea lamprey	Lamprey	krt8.1	XP_032801610	GenBank
			krt8.2	XP_032807055	GenBank
			krt8.3	XP_032812943	GenBank
			krt8.4	XP_032812945	GenBank
			krt8.5	XP_032812944	GenBank
			krt8.6	XP_032812946	GenBank

			krt8.7	XP_032821562	GenBank
			krt8.8	XP_032826365	GenBank
			krt8.9	XP_032836208	GenBank
			krt8.10	XP_032801611	GenBank
Lethenteron reissneri	far eastern brook lamprey	n.a.	krt8.1	n.a. (related to XP_061433315)[1]	GenBank
			krt8.2	n.a. (related to XP_061433315)[1]	GenBank
			krt8.3	XP_061431777	GenBank
			krt8.4	XP_061431767	GenBank
			krt8.5	XP_061419282	GenBank
			krt8.6	XP_061431170	GenBank
			krt8.7	XP_061431169	GenBank
			krt8.8	XP_061413498	GenBank
			krt8.9	XP_061413497	GenBank
			krt8.10	XP_061410631	GenBank
			krt8.11	XP_061405481	GenBank
Eptatretus burgeri	Inshore hagfish	Hagfish	krt8.1	ENSEBUP00000016891	Ensembl
			krt8.3	ENSEBUP00000019876	Ensembl
			krt8.4	ENSEBUP00000009095	Ensembl
			krt8.7	ENSEBUP00000009209	Ensembl
			krt8.8	n.a. (related to ENSEBUP00000018890)[2]	Ensembl
			krt8.10	ENSEBUP00000015898	Ensembl
Eptatretus atami	Brown hagfish	n.a.	krt8.1	EA37681	Zenodo[3]
			krt8.2	EA38814	Zenodo[3]
			krt8.3	EA38903	Zenodo[3]
			krt8.4	EA29089	Zenodo[3]
			krt8.5	EA37381	Zenodo[3]
			krt8.6	EA24614	Zenodo[3]

Notes:
[1] The prediction of coding sequence (cds) of the corresponding gene was corrected.
[2] The complete cds was obtained from juvenile head trancriptome (SRX2541845)
[3] The peptide file (Pata_ah2p.pep.fa) is available at https://zenodo.org/records/10227719.

Supplementary Table S3. Keratins of jawed fish

Species	Species (binomial name)	Taxon	Gene symbol	Keratin type	Protein (GenBank accession number)	Number of cysteine residues
Great white shark	*Carcharodon carcharias*	Chondrichthyes, Selachimorpha	krt222	type I	XP_041029719.1	6
			LOC121269163	type I	XP_041029614.1	1
			LOC121269037	type I	XP_041029365.1	1
			LOC121269056	type I	XP_041029409.1	1
			LOC121273230	type I	XP_041036179.1	0
			LOC121284630	type I	XP_041056093.1	3
			si:ch211-243g18.2	type I	XP_041049890.1	1
			LOC121271425	type I	XP_041033366.1	0
			LOC121271433	type I	XP_041033373.1	0
			LOC121274134	type I	XP_041037241.1	2
			LOC121273229	type II	XP_041036178.1	0
			LOC121273223	type II	XP_041036173.1	0
			LOC121273226	type II	XP_041036176.1	0
			LOC121273224	type II	XP_041036174.1	0
			LOC121273225	type II	XP_041036175.1	0
			LOC121273222	type II	XP_041036172.1	0
			LOC121273227	type II	XP_041036177.1	0
			LOC121284626	type II	XP_041056079.1	1
Smalltooth sawfish	*Pristis pectinata*	Chondrichthyes, Batoidea (rays)	krt222	type I	XP_051894676.1	7
			LOC127582940	type I	XP_051894556.1	1
			LOC127583009	type I	XP_051894658.1	1
			si:ch211-243g18.2	type I	XP_051893282.1	2
			LOC127584977	type I	XP_051898021.1	0
			LOC127584979	type I	XP_051898026.1	0
			LOC127566939	type I	XP_051865435.1	0
			LOC127574698	type I	XP_051879945.1	9
			LOC127566938	type II	XP_051865434.1	2
			LOC127566957	type II	XP_051865480.1	2
			LOC127566956	type II	XP_051865479.1	2
			LOC127566958	type II	XP_051865481.1	0
			LOC127569718	type II	XP_051870493.1	1
Grey bichir	*Polyterus senegalus*	Actinopterygii, Polypteriformes	krt222	type I	XP_039596227.1	11
			LOC120517581	type I	XP_039595920.1	1
			LOC120517582	type I	XP_039595921.1	0
			LOC120518025	type I	XP_039596510.1	1
			LOC120517580	type I	XP_039595919.1	0
			LOC120517986	type I	XP_039596465.1	1
			LOC120518116	type I	XP_039596653.1	0
			LOC120518115	type I	XP_039596652.1	0
			zgc:92380	type I	XP_039612352.1	1
			LOC120541364	type I	XP_039628852.1	0
			LOC120541363	type I	XP_039628852.1	0

			LOC120541362	type I	XP_039628846.1	0
			LOC120541365	type I	XP_039628854.1	0
			LOC120541370	type I	XP_039628860.1	0
			LOC120541371	type I	XP_039628862.1	1
			LOC120524911	type I	XP_039602709.1	1
			LOC120531211	type II	XP_039612354.1	0
			LOC120524912	type II	XP_039602711.1	2
			LOC120524913	type II	XP_039602712.1	0
			LOC120524915	type II	XP_039602713.1	2
			LOC120526748	type II	XP_039605833.1	0
Zebrafish	*Danio rerio*	Actinopterygii, Teleostei	cyt1	type I	NP_571182.1	0
			cyt1l	type I	NP_001076351.1	0
			krt1-19d	type I	XP_001342789.4	0
			krt15	type I	NP_998688.2	0
			krt17	type I	NP_001076574.1	0
			krt18	type I	NP_848524.1	0
			krt18b	type I	NP_956862.1	1
			krt1-c5	type I	XP_009300228.1	4
			krt222	type I	XP_001343225.3	6
			krt91	type I	NP_001003445.1	0
			krt92	type I	NP_001017824.1	0
			krt93	type I	NP_001035449.1	4
			krt94	type I	NP_001070922.1	0
			krt95	type I	NP_001002392.1	26
			krt96	type I	NP_001186881.1	0
			krt97	type I	NP_001002383.1	0
			krt98	type I	XP_005155788.1	0
			krt99	type I	NP_001017588.1	2
			krtt1c19e	type I	NP_001107814.1	0
			si:ch211-133j6.3	type I	NP_001373396.1	1
			si:ch211-156l18.7	type I	XP_683942.5	20
			krt4	type II	NP_571584.2	0
			krt5	type II	XP_017209104.1	0
			krt8	type II	NP_956374.1	1
			si:dkey-222f2.1	type II	XP_696736.2	0
			si:dkey-222n6.2	type II	XP_003199098.1	3
			zgC:158846	type II	NP_001076492.1	1

Table S4. Positions of the protein-coding exonic sequences of *krt18.5* (Accession: EA85498) of *Eptatretus atami* (GCA_035128595.1)

Exon	GenBank accession number	cds start	cds end
1	CM068966.1	33060942	33061409
2	CM068966.1	33061625	33061711
3	CM068966.1	33061943	33062098
4	CM068966.1	33062357	33062521
5	CM068966.1	33062693	33062818
6	CM068966.1	33063119	33063337
7	CM068966.1	33063733	33063801
8	CM068966.1	33065210	33065269

Note: cds, coding sequence
